# *anti*-Selective synthesis of β-boryl-α-amino acid derivatives by Cu-catalysed borylamination of α,β-unsaturated esters†

**DOI:** 10.1039/d2sc06003e

**Published:** 2022-11-24

**Authors:** Soshi Nishino, Yuji Nishii, Koji Hirano

**Affiliations:** Department of Applied Chemistry, Graduate School of Engineering, Osaka University Suita Osaka 565-0871 Japan k_hirano@chem.eng.osaka-u.ac.jp; Innovative Catalysis Science Division, Institute for Open and Transdisciplinary Research Initiatives (ICS-OTRI), Osaka University Suita Osaka 565-0871 Japan

## Abstract

A copper-catalysed regio- and diastereoselective borylamination of α,β-unsaturated esters with B_2_pin_2_ and hydroxylamines has been developed to deliver acyclic β-boryl-α-amino acid derivatives with high *anti*-diastereoselectivity (up to >99 : 1), which is difficult to obtain by the established methods. A chiral phosphoramidite ligand also successfully induces the enantioselectivity, giving the optically active β-borylated α-amino acids. The products can be stereospecifically transformed into β-functionalised α-amino acids, which are of potent interest in medicinal chemistry.

## Abstract 2

A Cu-catalysed regio-, diastereo-, and enantioselective borylamination of α,β-unsaturated esters with B_2_pin_2_ and hydroxylamines has been developed. The products can be converted into functionalised α-amino acids with two adjacent stereocentres.

## Graphical abstract

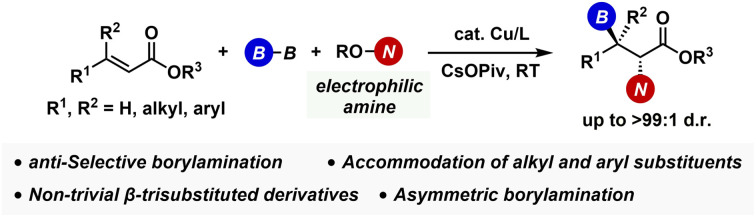

## Introduction

**Scheme 1:**
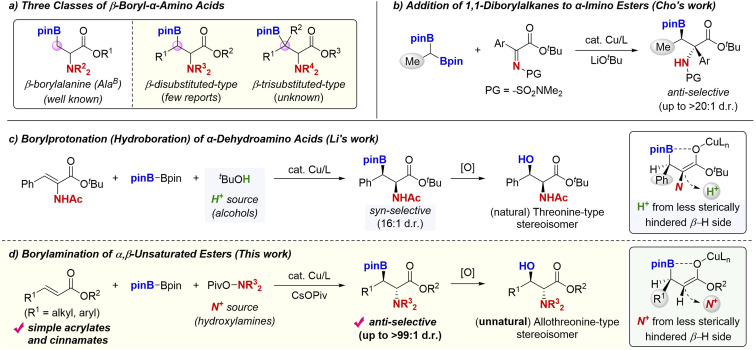
β-Boryl-α-amino acids: classification and their synthetic strategies.

Unnatural α-amino acids are key structures for the synthesis of modified peptide drugs to improve their activities and stabilities.^1^ In this context, the β-boryl-α-amino acids have received considerable attention since these compounds can be of high potential in the peptidemimetic strategy^2^ and easily transformed to natural/unnatural β-functionalised α-amino acids such as β-hydroxy-α-amino acids, which are frequently found in drugs and bioactive molecules.^3^ Thus, the development of efficient and stereoselective synthetic methods for their preparation is of importance not only in synthetic chemistry but also in biological and pharmaceutical research fields. The synthesis of the most simple β-borylalanine (*Ala*^B^; Scheme 1a, left) has been well developed by Curtius rearrangement,^4*a*^ substitution reaction with boron electrophile,^4*b*^ boron conjugate addition,^4*c*^ C–H borylation,^4*d*^ and decarboxylative borylation.^4*e*^ On the other hand, the more sterically hindered β-disubstituted derivatives^5^ are still challenging synthetic targets despite the fact that such sterically congested α-amino acid derivatives are promising building blocks in the preparation of modified peptides (Scheme 1a, middle, β-disubstituted-type).^1^ In particular, there are a few examples of the diastereoselective synthesis of acyclic derivatives. Cho developed the copper-catalysed stereoselective addition of 1,1-diborylalkanes to α-imino esters to form the corresponding *anti*-β-boryl-α-amino acids (Scheme 1b).^5*h*^ While a variety of α-imino esters could be employed, only the Me-substituted 1,1-diborylalkane was used in almost cases, which largely limited the substituent pattern at the β-position. On the other hand, Li reported the borylcopper-mediated borylprotonation of the α-dehydroalanine with B_2_pin_2_ and proton sources (alcohols) for preparation of the disubstituted-type β-boryl-α-amino acids (Scheme 1c).^5*b*^ This method showed the remarkably high *syn*-diastereoselectivity. Such a stereochemical control is proposed to be induced by a strong interaction between the boron and oxygen in the β-borylated *O*-bound copper enolate intermediate, which regulates the molecular conformation.^6^ Accordingly, the subsequent protonation with alcohols proceeds on the less sterically hindered H side, giving the *syn*-product selectively. The obtained *syn*-isomer can be delivered to the β-hydroxy-α-amino acid with natural threonine-type relative stereochemistry. However, just one example was demonstrated, and the generality of this process thus still remains unclear. Furthermore, the synthesis of the most sterically demanding trisubstituted β-boryl-α-amino acids (Scheme 1a, right, β-trisubstituted-type) has not been reported yet, except for a somewhat specialised cyclic derivative.^7^

Herein, we report a general and catalytic way to the acyclic *anti*-β-boryl-α-amino acids; a copper-catalysed borylamination^8,9^ of the α,β-unsaturated carboxylic acid derivatives with B_2_pin_2_ and hydroxylamines (Scheme 1d).^10^ Analogous to Li's proposal (Scheme 1c), the conformationally regulated β-borylated *O*-bound copper enolate intermediate undergoes the face-selective C–N bond formation with the hydroxylamine^11^ on the more sterically accessible H side, en route to the *anti*-β-boryl-α-amino acid (up to >99 : 1 d.r). The *anti*-isomer can be transformed to the unnatural allothreonine-type β-hydroxy-α-amino acids of higher value. Additional synthetic advantages of this method include (1) the ready availability of starting materials, α,β-unsaturated esters, (2) accommodation of versatile aromatic and aliphatic substituents at the β-position, and (3) the successful use of β,β-disubstituted acrylates, thus leading to the most bulky trisubstituted β-boryl-α-amino acids. Moreover, an appropriate chiral phosphine ligand makes the reaction enantioselective, affording optically active β-borylated α-amino acid derivatives. The follow-up stereospecific transformations of the Bpin moiety delivered the enantioenriched unnatural α-amino acids with versatile functionalities at the β-position. Although the related copper-catalysed boron conjugate addition of α,β-unsaturated carbonyls was well studied,^12^ the tandem α-functionalisation of the copper enolate intermediate still remains underdeveloped, except for the classical aldol-type processes.^13^

## Results and discussion

Our optimisation studies commenced with β-monosubstituted unsaturated ester 1a, B_2_pin_2_ (2.5 equiv.), and *O*-pivaloyl-*N*,*N*-dibenzylhydroxylamine (2a-Piv; 1.5 equiv.) as model substrates (Table 1). The initial screening of ligands in 1,4-dioxane at room temperature in the presence of a Cu(OAc)_2_·H_2_O catalyst (12 mol%) and a CsOPiv base (3.0 equiv.) revealed that the monodentate phosphine ligands were more effective than bidentate ones: PPh_3_ showed better performance than dppbz, dppe, and Xantphos (entries 1–4). On the other hand, the diastereomeric ratio (d.r.) was uniformly high (94 : 6 to 97 : 3 *anti*/*syn*), thus suggesting that the nature of ligands on the copper gives negligible impact on the face selection in the amination step (Scheme 1d). More electron-withdrawing P(3,4,5-F_3_C_6_H_2_)_3_ slightly improved the yield (entry 6). The CsOPiv base was critical to suppress the competitive protonation of the copper enolate intermediate: Cs_2_CO_3_ and NaO^*t*^Bu afforded the hydroborylated 4a as the major product (entries 7 and 8). The absence of base also resulted in no product formation, and only the undesired 4a was formed in 23% yield along with 68% recovery of 1a (entry 9). Additional investigation of ligands identified P(3,5-F_2_C_6_H_3_)_3_ to be best (entry 10). Among copper catalyst precursors we tested, Cu(OAc)_2_ anhydrate further increased the yield (entries 11 and 12). Finally, with a reduced catalyst loading (10 mol%) and toluene solvent instead of 1,4-dioxane, the desired product was isolated in 74% yield as the single *anti*-isomer (entry 13; see the ESI for more detailed optimisation studies†).^14,15^

**Table 1:** Optimisation studies for copper-catalysed borylamination of α,β-unsaturated ester 1a with B_2_pin_2_ and hydroxylamine 2a-Piv^a^

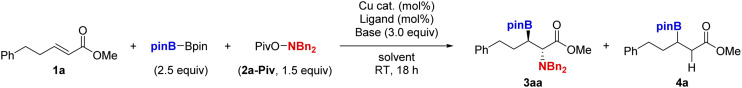						
Entry	Cu cat. (mol%)	Ligand (mol%)	Base	Solvent	Yield of 3aa (%), d.r^b^	Yield of 4a (%)^b^
1	Cu(OAc)_2_·H_2_O (12)	dppbz (12)	CsOPiv	1,4-dioxane	16, 94 : 6	49
2	Cu(OAc)_2_·H_2_O (12)	dppe (12)	CsOPiv	1,4-dioxane	31, 96 : 4	54
3	Cu(OAc)_2_·H_2_O (12)	Xantphos (12)	CsOPiv	1,4-dioxane	0, —	18
4	Cu(OAc)_2_·H_2_O (12)	PPh_3_ (24)	CsOPiv	1,4-dioxane	65, 97 : 3	29
5	Cu(OAc)_2_·H_2_O (12)	P(4-MeOC_6_H_4_)_3_ (24)	CsOPiv	1,4-dioxane	51, 97 : 3	38
6	Cu(OAc)_2_·H_2_O (12)	P(3,4,5-F_3_C_6_H_2_)_3_ (24)	CsOPiv	1,4-dioxane	68, 98 : 2	25
7	Cu(OAc)_2_·H_2_O (12)	P(3,4,5-F_3_C_6_H_2_)_3_ (24)	Cs_2_CO_3_	1,4-dioxane	40, 97 : 3	59
8	Cu(OAc)_2_·H_2_O (12)	P(3,4,5-F_3_C_6_H_2_)_3_ (24)	NaO^*t*^Bu	1,4-dioxane	22, 97 : 3	78
9	Cu(OAc)_2_·H_2_O (12)	P(3,4,5-F_3_C_6_H_2_)_3_ (24)	None	1,4-dioxane	0, —	23
10	Cu(OAc)_2_·H_2_O (12)	P(3,5-F_2_C_6_H_3_)_3_ (24)	CsOPiv	1,4-dioxane	76, 96 : 4	21
11	Cu(OAc)_2_ (12)	P(3,5-F_2_C_6_H_3_)_3_ (24)	CsOPiv	1,4-dioxane	80, 97 : 3	18
12	CuCl (12)	P(3,5-F_2_C_6_H_3_)_3_ (24)	CsOPiv	1,4-dioxane	78, 97 : 3	21
13	Cu(OAc)_2_ (10)	P(3,5-F_2_C_6_H_3_)_3_ (20)	CsOPiv	Toluene	80 (74), >99 : 1	20

a Conditions: 1a (0.25 mmol), B_2_pin_2_ (0.63 mmol), 2a-Piv (0.38 mmol), Cu(OAc)_2_·H_2_O, ligand, base (0.75 mmol), solvent (1.0 mL), RT, 18 h, N_2_.

b Estimated by ^1^H NMR based on 0.25 mmol with 1-methylnaphthalene as the internal standard. The diastereomeric ratio (d.r.) is determined in the crude mixture. Isolated yield is in parentheses.

**Scheme 2:**
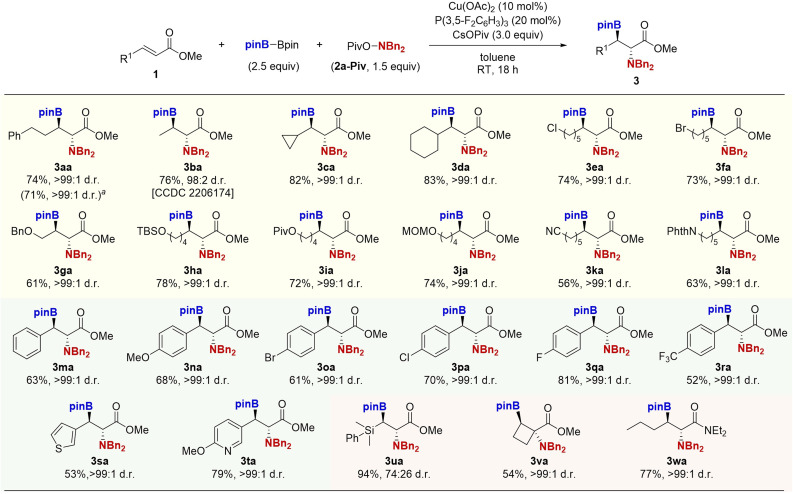
Copper-catalysed borylamination of various α,β-unsaturated esters 1 with B_2_pin_2_ and *N*,*N*-dibenzylhydroxylamine 2a-Piv. Conditions: 1 (0.25 mmol), B_2_pin_2_ (0.63 mmol), 2a-Piv (0.38 mmol), Cu(OAc)_2_ (0.025 mmol), P(3,5-F_2_C_6_H_3_)_3_ (0.050 mmol), CsOPiv (0.75 mmol), toluene (1.0 mL), RT, 18 h, N_2_. Isolated yields are given. ^*a*^On a 1.0 mmol scale.

With conditions of entry 13 in Table 1, we examined the generality of the copper-catalysed borylamination reaction (Scheme 2). Both simple crotonate and *γ*-branched acrylates underwent the reaction smoothly to form the desired products 3ba–da in good yields with high *anti*-diastereoselectivity. The reaction was tolerant of versatile functional groups including alkyl halide, ether, ester, acetal, nitrile, and phthalimide moieties to afford the corresponding β-boryl-α-amino acids 3ea–la in moderate to good yields as the single *anti*-diastereomers. Additionally, the electronically diverse cinnamates could also be employed; the electron-donating methoxy and electron-withdrawing bromo, chloro, fluoro, and trifluoromethyl groups all were tolerated to afford the corresponding β-boryl-α-amino acid derivatives 3ma–ra in good yields with the exclusive *anti*-selectivity. The heteroaromatic thiophene and pyridine substrates were also adopted to deliver the targeted α-amino acids 3sa–ta. Notably, the α-amino acid containing the *gem*-boryl-silyl structure was also prepared from the β-silyl acrylate (3ua). The cyclobutenecarboxylate ester was successfully converted to the α,α-disubstituted β-boryl-α-amino acid 3va. Furthermore, the copper catalyst was applicable to the α,β-unsaturated amide to give the β-boryl-α-amino amide 3wa. The relative stereochemistry of 3ba was confirmed by X-ray analysis (CCDC 2206174),† and others were assigned by analogy.

**Scheme 3:**
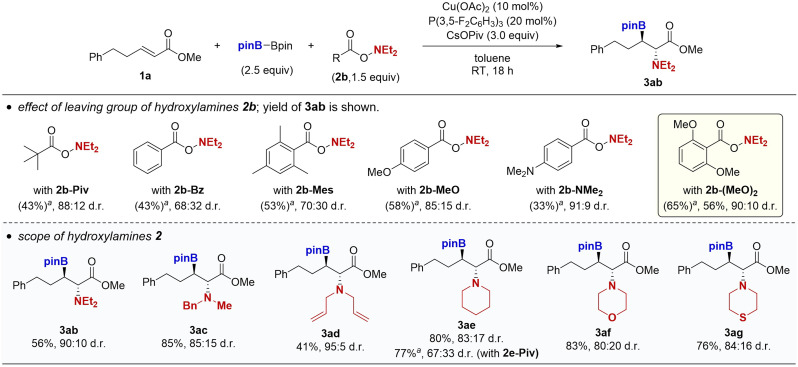
Copper-catalysed borylamination of α,β-unsaturated esters 1a with B_2_pin_2_ and various hydroxylamines 2. Conditions: 1a (0.25 mmol), B_2_pin_2_ (0.63 mmol), 2 (0.38 mmol), Cu(OAc)_2_ (0.025 mmol), P(3,5-F_2_C_6_H_3_)_3_ (0.050 mmol), CsOPiv (0.75 mmol), toluene (1.0 mL), RT, 18 h, N_2_. Isolated yields are given. ^*a*^NMR yield.

We next investigated the scope of the hydroxylamines (Scheme 3). While the pivalate leaving group was optimal in the case of the *N*,*N*-dibenzylamine, other acyclic and cyclic amines required the more sterically hindered and strongly electron-donating *o*,*o*-dimethoxybenzoyloxy leaving group for the acceptable reaction efficiency and diastereoselectivity. For example, the borylamination of 1a with *O*-pivaloyl-*N*,*N*-diethylhydroxylamine (2b-Piv) resulted in 43% yield of 3ba with 88 : 12 d.r. Less sterically hindered benzoyl-type 2b-Bz largely dropped the diastereomeric ratio. Introduction of electron-donating groups improved the stereoselectivity (2b-Mes, 2b-MeO, and 2b-NMe_2_), with 2b-(MeO)_2_ proving to be best (65% ^1^H NMR yield, 90 : 10 d.r). The observed trend was consistent with our proposal in Scheme 1d, where the *anti*-selectivity is generally induced by the steric repulsion between the substituent at the β-position and amino electrophile. Moreover, the electron-donating substituent can suppress the direct reaction of the borylcopper with the hydroxylamine to avoid its unproductive decomposition. The modified leaving group was also effective for *N*-benzyl-*N*-methylamine, *N*,*N*-diallylamine, piperidine, morpholine, and thiomorpholine to deliver the targeted β-boryl-α-amino acids 3ac–ag in 41–85% yields with synthetically useful diastereomeric ratios (80 : 20–95 : 5 *anti*/*syn*). The positive effects of *o*,*o*-dimethoxybenzoyl group were more remarkable in the reaction with relatively small cyclic amines such as piperidine (3ae). A similar beneficial effect of the *o*,*o*-dimethoxy substitution was reported in the nickel-catalysed carboamination reaction of alkenes developed by Engle.^16^

**Scheme 4:**
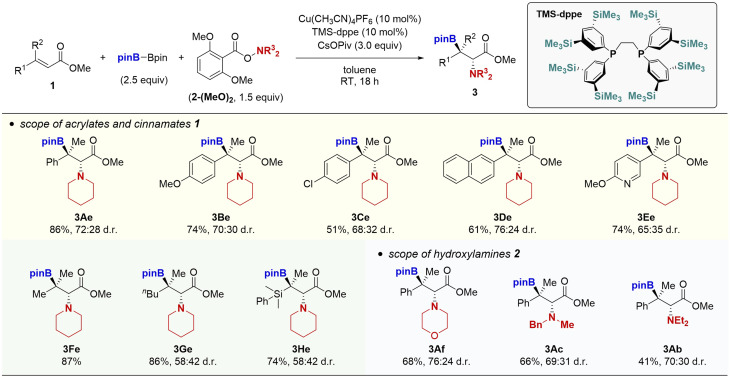
Copper-catalysed borylamination of β,β-disubstituted cinnamates and acrylates. Conditions: 1 (0.25 mmol), B_2_pin_2_ (0.63 mmol), 2-(MeO)_2_ (0.38 mmol), Cu(CH_3_CN)_4_PF_6_ (0.025 mmol), TMS-dppe (0.025 mmol), CsOPiv (0.75 mmol), toluene (1.0 mL), RT, 18 h, N_2_. Isolated yields are given.

The aforementioned success prompted us to attempt the borylamination of β,β-disubstituted α,β-unsaturated esters (Scheme 4). This is highly challenging because the rate of 1,4-addition of borylcopper species to the sterically congested β,β-disubstituted unsaturated esters is much slower than that of β-monosubstituted ones to predominantly decompose the hydroxylamine by the direct reaction.^17^ Actually, in the reaction of the β-methylcinnamate 1A and piperidine derivative 2e-(MeO)_2_, the Cu(OAc)_2_/P(3,5-F_2_C_6_H_3_)_3_ catalyst system did not provide the target product 3Ae at all, even with the assistance of the modified *o*,*o*-dimethoxybenzoyloxy leaving group. Thus, we again performed optimisation studies. After extensive re-screening of various catalysts and ligands, the combination of a cationic copper salt, Cu(CH_3_CN)_4_PF_6_ and TMS-modified dppe ligand^18^ was found to dramatically promote the reaction to give the trisubstituted β-boryl-α-amino acids 3Ae in 86% yield (see the ESI† for details). Although the diastereomeric ratio was moderate (72 : 28 *anti*/*syn*),^19^ to the best of our knowledge, this is the first successful example of preparation of acyclic β-boryl-α-amino acid derivatives with the *tetra*-substituted carbon centre at the β-position. The newly developed copper catalysis was applicable to other β-methylcinnamates (3Be–Ee) and acrylates (3Fe–He). As the hydroxylamine coupling partner, morpholine (3Af), *N*-benzyl-*N*-methylamine (3Ac), and *N*,*N*-diethylamine (3Ab) also worked well to furnish the corresponding trisubstituted β-boryl-α-amino acids in moderate to good yields.

**Scheme 5:**
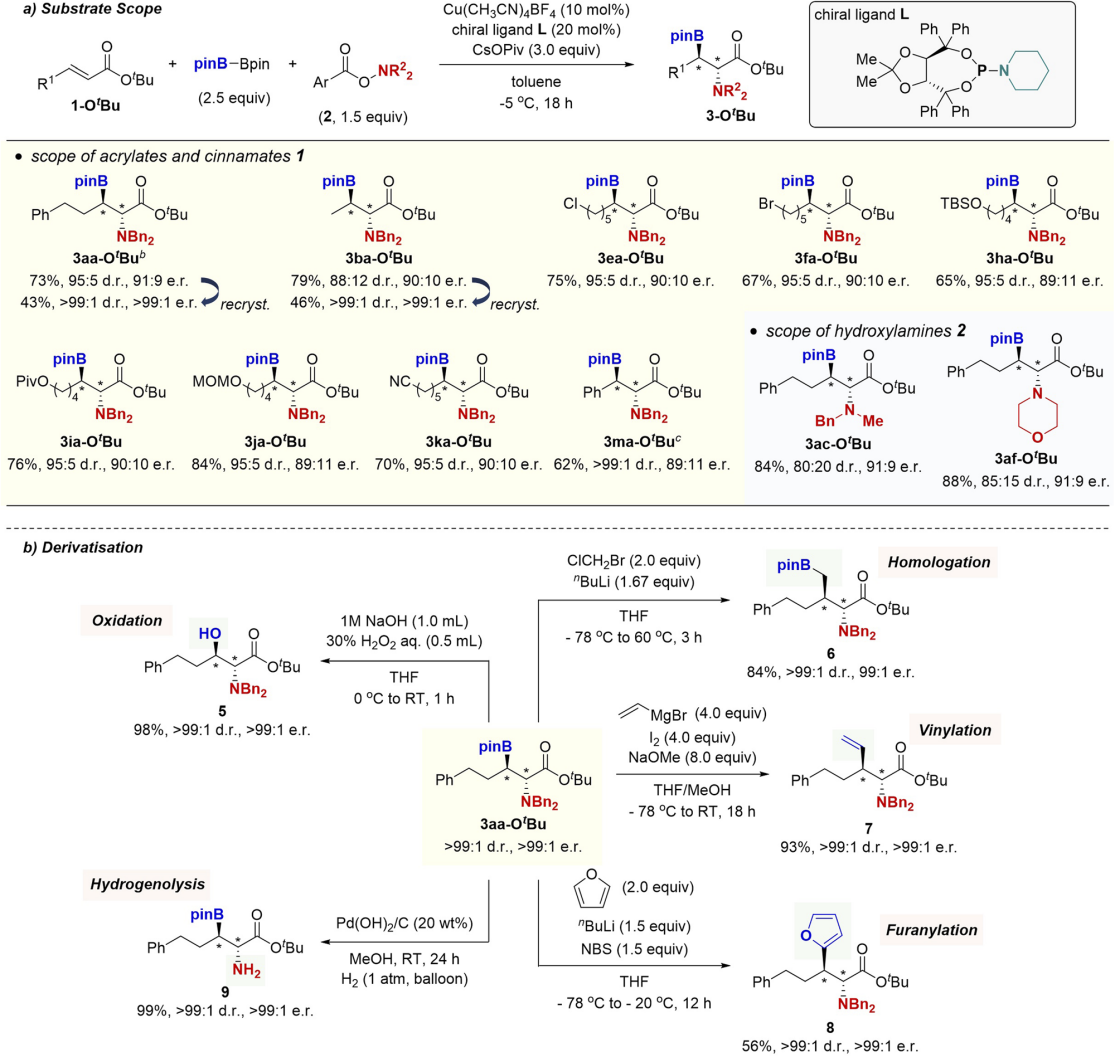
(a) Copper-catalysed enantioselective borylamination of α,β-unsaturated esters^*a*^ and (b) derivatisations of 3aa-O*^t^*Bu. ^*a*^Conditions: 1-O*^t^*Bu (0.25 mmol), B_2_pin_2_ (0.63 mmol), 2 (0.38 mmol), Cu(CH_3_CN)_4_BF_4_ (0.025 mmol), L (0.050 mmol), CsOPiv (0.75 mmol), toluene (1.0 mL), −5 °C, 18 h, N_2_. Ar = 4-MeOC_6_H_4_- (2a-OMe) or 2,6-(MeO)_2_C_6_H_3_- (2c-(OMe)_2_ and 2f-(OMe)_2_). Isolated yields are given. ^*b*^On a 1.0 mmol scale. ^*c*^On a 0.50 mmol scale.

We next turned attention to the diastereo- and enantioselective borylamination. Our initial attempts of common chiral bidentate ligands, such as Quinox P*, Ph-BPE, SEGPHOS, BINAP, and Josiphos led to no product formation or low enantioselectivity. On the other hand, several chiral monodentate phosphoramidite ligands were found to be good candidates. In particular, TADDOL-based piperidine phosphoramidite L^20^ successfully induced the high enantioselectivity as well as diastereoselectivity in the reaction of ^*t*^Bu ester 1a-O*^t^*Bu and *O*-(4-MeO)benzoyl-*N*,*N*-dibenzylhydroxylamine (2a-OMe) to furnish 3aa-O*^t^*Bu in 74% yield with 95 : 5 d.r. and 91 : 9 e.r. (Scheme 5a). The (2*R*,3*R*) absolute configuration was determined by comparison of retention time in chiral HPLC analysis with the known compound after the oxidative derivatisation (see the ESI† for details). The asymmetric catalysis was compatible with the alkyl chloride, alkyl bromide, silyl ether, pivaloyl ester, acetal, and nitrile functionalities, and the functionalised β-boryl-α-amino acids 3ea-O*^t^*Bu, 3fa-O*^t^*Bu, 3ha-O*^t^*Bu, 3ia-O*^t^*Bu, 3ja-O*^t^*Bu, and 3ka-O*^t^*Bu were prepared in good yields with 89 : 11 to 90 : 10 e.r. Cinnamate 1m-O*^t^*Bu was also applicable to the enantioselective borylamination with synthetically acceptable enantioselectivity. In addition to 2a-OMe, *N*-benzyl-*N*-methylamine 2c-(OMe)_2_ and morpholine 2f-(OMe)_2_ were viable to produce 3ac-O*^t^*Bu and 3af-O*^t^*Bu with good enantiomeric ratios. Moreover, single recrystallisation from Et_2_O/hexane afforded the optically pure β-boryl-α-amino acids (3aa-O*^t^*Bu and 3ba-O*^t^*Bu) with adjacent two stereocentres (>99 : 1 d.r. and >99 : 1 e.r).

To further demonstrate the synthetic utility of the copper-catalysed borylamination, we converted the stereochemically pure β-boryl-α-amino acid 3aa-O*^t^*Bu into functionalised α-amino acid derivatives based on the established organoboron chemistry (Scheme 5b). The enantioenriched β-hydroxy-α-amino acid 5 with the unnatural *anti*-configuration could be easily accessed by oxygenation with H_2_O_2_. Matteson homologation^21^ with the *in situ*-generated LiCH_2_Cl was also possible to afford optically active 6. Additionally, Zweifel-type olefination^22^ delivered the vinylation product 7 in 93% yield with complete stereoretention. The coupling with furan could also be conducted under conditions developed by Aggarwal^23^ to furnish the cross-coupling product 8 in 88% yield without any erosion of the stereochemistry. Furthermore, the hydrogenolysis of *N*-benzyl groups proceeded without any detectable deborylation to give the primary amine 9 in 78% yield.

## Conclusions

We have developed an *anti*-selective copper-catalysed borylamination of α,β-unsaturated esters with B_2_pin_2_ and hydroxylamines to give the corresponding acyclic β-boryl-α-amino acid derivatives with high diastereoselectivity (up to >99 : 1 *anti*/*syn*). The use of the amino electrophile is critical to induce the *anti*-stereochemistry in the acyclic system, which is otherwise difficult to obtain by the reported procedures. Additionally, the originally developed modified dppe-type ligand accommodates the more sterically congested β,β-disubstituted cinnamates and acrylates to form the non-trivial β-trisubstituted derivatives. Furthermore, the enantioselectivity is successfully induced by the appropriate chiral phosphoramidite ligand. The obtained optically active β-boryl-α-amino acid with adjacent two stereocentres can be easily transformed into highly functionalised α-amino acids with the *anti*-stereochemistry, which demonstrates the synthetic value of our protocol. More detailed mechanistic studies and asymmetric synthesis of the most congested β-trisubstituted-type β-boryl-α-amino acids^24^ are ongoing in our laboratory.

## Data availability

All experimental procedures and spectroscopic data can be found in the ESI.†

## Author contributions

S. N. and K. H. conceived the idea. S. N. performed all experiments. K. H. supervised the project. Y. N. supported X-ray analysis. The paper was written by S. N. and K. H. All the authors discussed the results and commented on the manuscript.

## Conflicts of interest

There are no conflicts to declare.

## Supplementary Material

SC-013-D2SC06003E-s001

SC-013-D2SC06003E-s002
