# Supplementary material for: anti-Selective synthesis of β-boryl-α-amino acid derivatives by Cu-catalysed borylamination of α,β-unsaturated esters
Source: Chem Sci. 2022 Nov 24;13(48):14387–94. doi: 10.1039/d2sc06003e (PMC9749109; doi:10.1039/d2sc06003e)
Supplement: SC-013-D2SC06003E-s001 [file SC-013-D2SC06003E-s001.pdf]

Electronic Supplementary Material (ESI) for Chemical Science.
This journal is © The Royal Society of Chemistry 2022

# Supplementary Information

**_anti_-Selective synthesis of _β_-boryl-_α_-amino acid derivatives**

**by Cu-catalysed borylamination of _α,β_-unsaturated esters**

Soshi Nishino,[†] Yuji Nishii,[†] and Koji Hirano*[,†,‡]

[†]*Department of Applied Chemistry, Graduate School of Engineering, Osaka University, Suita, Osaka 565-0871, Japan*

[‡]*Innovative Catalysis Science Division, Institute for Open and Transdisciplinary Research Initiatives (ICS-OTRI), Osaka University, Suita, Osaka 565-0871, Japan*

*E-mail: k_hirano@chem.eng.osaka-u.ac.jp (K.H.)*

**Contents**

**Instrumentation and Chemicals** S2-S3
**Experimental Procedures** S4–S8
**Detailed Optimisation Studies** S9–S21
**Substrate Limitation** S22
**Stereochemical Assignment** S23
**X-Ray Analysis** S24–S25
**Chiral HPLC Charts of Enantioenriched Products** S26–S45
**Characterisation Data for Products** S46–S187
**References** S188

## Instrumentation and Chemicals

$^{1}H$, $^{13}C\{^{1}H\}$, $^{19}F\{^{1}H\}$, and $^{11}B$ NMR spectra were recorded at 400, 100, 376, and 128 MHz, respectively, for $CDCl_3$ solutions. HRMS data were obtained by APCI using TOF. TLC analyses were performed on commercial glass plates bearing 0.25-mm layer of Merck silica gel $60F_{254}$ or Wako $NH_2$ Silica Gel $60F_{254}$. Silica gel 60 N (spherical, neutral) or $NH_2$ silica gel (Wakogel, $50NH_2$) were used for column chromatography. Toluene was dried on a Glass Contour Solvent dispensing system (Nikko Hansen & Co., Ltd.) prior to use. $Cu(OAc)_2$ was purchased from FUJIFILM Wako Pure Chemical Co. $Cu(CH_3CN)_4PF_6$, $Cu(CH_3CN)_4BF_4$, and bis(pinacolato)diboron were commercial sources from TCI. $P(3,5\text{-}F_2C_6H_3)_3$ was prepared from 3,5-difluorophenylmagnesium bromide and trichlorophosphine.[S1] TMS-dppe was synthesised from 3,5-bis(trimethylsilyl)phenylmagnesium bromide and 1,2-bis(dichlorophosphino)ethane.[S2] TADDOL-based piperidine phosphoramidite **L** was prepared from TADDOL, trichlorophosphine, and piperidine.[S3] CsOPiv (purchased from Aldrich) should be crushed to pieces with a mortar and a pestle in a glovebox filled with nitrogen and then dried at 100 °C under high vacuum overnight (note: this preactivation was essential for reproducibility). The acrylates and cinnamates **1a**, **c-g**, **k, l**, **o-t**, **A, C-E**, **G**, **1a-O*t*Bu**, **1e-O*t*Bu**, **1f-O*t*Bu**, and **1k-O*t*Bu** were prepared by the standard HWE reaction.[S4] The acrylates **1h-j**, **1h-O*t*Bu**, **1i-O*t*Bu**, and **1j-O*t*Bu** were synthesised from the ζ-hydroxy-*α*,*β*-unsaturated ester, which was prepared according to the literature.[S5] (*E*)-Methyl 4-methoxycinnamate (**1n**) and dimethyl mesaconate (**1B**) were produced from the commercially available corresponding cinnamic acids under conditions of classical Fischer esterification.[S6] The *β*-silyl-substituted acrylates **1u** and **1H** were obtained via conjugate silylation of the corresponding alkynoates.[S7] The cyclobutenecarboxylate ester **1v** was prepared according to the

literature.[S8] The *α*,*β*-unsaturated amide **1w** was synthesised by the condensation reaction of (*E*)-2-hexenoic acid (purchased from FUJIFILM Wako Pure Chemical Co.) with diethylamine. The methyl crotonate (**1b**), methyl cinnamate (**1m**), methyl 3-methyl-2-butenoate (**1F**), *tert*-butyl crotonate (**1b-O$^t$Bu**), *tert*-butyl cinnamate (**1m-O$^t$Bu**), were commercial sources. *O*-Pivaloyl-*N*,*N*-dibenzylhydroxylamine (**2a-Piv**) and *O*-4-methoxybenzoyl-*N*,*N*-dibenzylhydroxylamine (**2a-OMe**) were obtained by the reaction of *N*,*N*-dibenzylhydroxylamine with pivaloyl chloride and 4-methoxybenzoyl chloride, respectively. *O*-2,6-Dimethoxybenzoyl-*N*,*N*-dialkylhydroxylamines (**2-(OMe)$_2$**) was prepared by the reaction of 2,6-dimethoxybenzoyl chloride with *N*,*N*-alkylhydroxylamines, which were synthesised from the corresponding *O*-benzoyl-*N*,*N*-dialkylhydroxylamines.[S9] All reactions were carried out under nitrogen atmosphere unless otherwise noted.

**Experimental Procedures**

**Copper-Catalysed Regio- and Diastereoselective Borylamination of *α,β*-Unsaturated Esters**

Synthesis of **3aa** (Table 1, entry 13, 0.25 mmol scale): $Cu(OAc)_2$ (4.5 mg, 0.025 mmol), $P(3,5\text{-}F_2C_6H_3)_3$ (18.5 mg, 0.050 mmol), and CsOPiv (175.5 mg, 0.75 mmol) were placed in a 20 mL Schlenk tube, which was filled with nitrogen by using the Schlenk technique. Toluene (1.0 mL) was then added to the tube, and the suspension was stirred for 15 min at ambient temperature. Bis(pinacolato)diboron (158.7 mg, 0.63 mmol) was then added in one portion, and the resulting solution was stirred at the same temperature. After 5 min, *O*-pivaloyl-*N*,*N*-dibenzylhydroxylamine (**2a-Piv**, 111.5 mg, 0.38 mmol) was then added in one portion, and methyl (*E*)-5-phenylpent-2-enoate (**1a**, 47.5 mg, 0.25 mmol) was finally added dropwise. The reaction solution was stirred at room temperature for additional 18 h. The resulting mixture was directly filtered through a short pad of neutral alumina and $Na_2SO_4$. The filtrate was evaporated in vacuo and purified by silica gel column chromatography on neutral silica gel with hexane/ethyl acetate (5/1, v/v) and GPC ($CHCl_3$) to give methyl 2-(dibenzylamino)-5-phenyl-3-(4,4,5,5-tetramethyl-1,3,2-dioxaborolan-2-yl)pentanoate (**3aa**, 95.0 mg, 0.18 mmol) in 74% yield with >99:1 *anti*/*syn* ratio.

Synthesis of **3aa** (Scheme 2, 1.0 mmol scale): $Cu(OAc)_2$ (18.2 mg, 0.10 mmol), $P(3,5\text{-}F_2C_6H_3)_3$ (74.0 mg, 0.20 mmol), and CsOPiv (702.1 mg, 3.0 mmol) were placed in a two-necked 20 mL reaction flask, which was filled with nitrogen by using the Schlenk technique. Toluene (4.0 mL) was then added to the tube, and the suspension was stirred for 15 min at ambient temperature. Bis(pinacolato)diboron (634.9 mg, 2.5 mmol) was then added in one portion, and the resulting solution was stirred at the same temperature. After 5 min, *O*-pivaloyl-*N*,*N*-dibenzylhydroxylamine (**2a-Piv**, 446.2 mg, 1.5 mmol) was added in one portion, and methyl (*E*)-5-phenylpent-2-enoate (**1a**, 190.1 mg, 1.0 mmol) was finally added dropwise. The reaction solution was stirred at room temperature for additional 18 h. The resulting mixture was directly filtered through a short pad of neutral alumina and $Na_2SO_4$. The filtrate was evaporated in vacuo and purified by silica gel column chromatography on neutral silica gel with hexane/ethyl acetate (5/1, v/v) and GPC ($CHCl_3$) to give methyl 2-(dibenzylamino)-5-phenyl-3-(4,4,5,5-tetramethyl-1,3,2-dioxaborolan-2-yl)pentanoate (**3aa**, 365 mg, 0.71 mmol) in 71% yield with >99:1 *anti*/*syn* ratio.

**Copper-Catalysed Regioselective Borylamination of *β,β*-Disubstituted *α,β*-Unsaturated Esters**

Synthesis of **3Ae** (Scheme 4, 0.25 mmol scale): $Cu(CH_3CN)_4PF_6$ (9.3 mg, 0.025 mmol), TMS-dppe (24.4 mg, 0.025 mmol), and CsOPiv (175.5 mg, 0.75 mmol) were placed in a 20 mL Schlenk tube, which was filled with nitrogen by using the Schlenk technique. Toluene (1.0 mL) was then added to the tube, and the suspension was stirred for 15 min at ambient temperature. Bis(pinacolato)diboron (158.7 mg, 0.63 mmol) was then added in one portion, and the resulting solution was stirred at the same temperature. After 5 min, *O*-2,6-dimethoxybenzoyl-hydroxypiperidine (**2e-(OMe)$_2$**, 99.5 mg, 0.38 mmol) was added in one portion, and methyl (*E*)-*β*-methylcinnamate (**1A**, 44.0 mg, 0.25 mmol) was finally added dropwise. The reaction solution was stirred at room temperature for additional 18 h. The resulting mixture was directly filtered through a short pad of neutral alumina and $Na_2SO_4$. The filtrate was evaporated in vacuo and purified by silica gel column chromatography on $NH_2$ silica gel with hexane/ethyl acetate (5/1, v/v) and GPC ($CHCl_3$) to give methyl 3-phenyl-2-(piperidin-1-yl)-3-(4,4,5,5-tetramethyl-1,3,2-dioxaborolan-2-yl)butanoate (**3Ae**, 83.3 mg, 0.22 mmol) in 86% yield with 72:28 *anti*/*syn* ratio.

**Copper-Catalysed Regio-, Diastereo-, and Enantioselective Borylamination of *α,β*-Unsaturated Esters**

Synthesis of **3aa-O$^t$Bu** (Table S9, entry 27, 0.25 mmol scale): $Cu(CH_3CN)_4BF_4$ (7.9 mg, 0.025 mmol), TADDOL-based piperidine phosphoramidite **L** (29.0 mg, 0.050 mmol), and CsOPiv (175.5 mg, 0.75 mmol) were placed in a 20 mL Schlenk tube, which was filled with nitrogen by using the Schlenk technique. Toluene (1.0 mL) was then added to the tube, and the suspension was stirred for 15 min at -5 °C. Bis(pinacolato)diboron (158.7 mg, 0.63 mmol) was then added in one portion, and the resulting solution was stirred at the same temperature. After 5 min, *O*-4-methoxybenzoly-*N*,*N*-dibenzylhydroxylamine (**2a-OMe**, 130.3 mg, 0.38 mmol) was added in one portion, and *tert*-butyl (*E*)-5-phenylpent-2-enoate (**1a-O$^t$Bu**, 58.1 mg, 0.25 mmol) was finally added dropwise. The reaction solution was stirred at -5 °C for additional 18 h. The resulting mixture was directly filtered through a short pad of neutral alumina and $Na_2SO_4$. The filtrate was evaporated in vacuo and purified by silica gel column chromatography on neutral silica gel with hexane/ethyl acetate (5/1, v/v) and GPC ($CHCl_3$) to give *tert*-butyl 2-(dibenzylamino)-5-phenyl-3-(4,4,5,5-tetramethyl-1,3,2-dioxaborolan-2-yl)pentanoate (**3aa-O$^t$Bu**, 102.7 mg, 0.19 mmol) in 74% yield with 95:5 *anti*/*syn* ratio. The enantiomeric ratio (er) of each diastereomer was determined to be 91:9 by chiral HPLC analysis on a chiral stationary phase.

Synthesis of **3aa-O$^t$Bu** (Scheme 5, 1.0 mmol scale): $Cu(CH_3CN)_4BF_4$ (31.5 mg, 0.10 mmol), TADDOL-based piperidine phosphoramidite **L** (115.9 mg, 0.20 mmol), and CsOPiv (702.1 mg, 3.0 mmol) were placed in a two-necked 20 mL reaction flask, which was filled with nitrogen by using the Schlenk technique. Toluene (4.0 mL) was then added to the tube, and the suspension was stirred for 15 min at -5 °C. Bis(pinacolato)diboron (634.9 mg, 2.5 mmol) was then added in one portion, and the resulting solution was stirred at the same temperature. After 5 min, *O*-4-methoxybenzoly-*N*,*N*-dibenzylhydroxylamine (**2a-OMe**, 521.1 mg, 1.5 mmol) was added in one portion, and *tert*-butyl (*E*)-5-phenylpent-2-enoate (**1a-O$^t$Bu**, 232.3 mg, 1.0 mmol) was finally added dropwise. The reaction solution was stirred at -5 °C for additional 18 h. The resulting mixture was directly filtered through a short pad of neutral alumina and $Na_2SO_4$. The filtrate was evaporated in vacuo and purified by silica gel column chromatography on neutral silica gel with hexane/ethyl acetate (5/1, v/v) and GPC ($CHCl_3$) to give *tert*-butyl 2-(dibenzylamino)-5-phenyl-3-(4,4,5,5-tetramethyl-1,3,2-dioxaborolan-2-yl)pentanoate (**3aa-O$^t$Bu**, 405.6 mg, 0.73 mmol) in 73% yield with 95:5 *anti*/*syn* ratio. The enantiomeric ratio (er) of each diastereomer was determined to be 91:9 by chiral HPLC analysis on a chiral stationary phase. Recrystallisation from $Et_2O$/hexane afforded stereochemically pure **3aa-O$^t$Bu** (238.9 mg, 0.43 mmol) in 43% yield, which was confirmed by chiral HPLC.

**Oxidation of 3aa-O$^t$Bu (Scheme 5)**

A 20 mL Schlenk tube equipped with a stir bar was charged with *tert*-butyl 2-(dibenzylamino)-5-phenyl-3-(4,4,5,5-tetramethyl-1,3,2-dioxaborolan-2-yl)pentanoate (**3aa-O$^t$Bu**, 55.6 mg, 0.10 mmol, >99:1 *anti*/*syn*, >99:1 er) and THF (1.0 mL). Sodium hydroxide aqueous solution (1 M, 1.0 mL) and hydrogen peroxide (30 wt%, 0.5 mL) were added to the reaction mixture, and the solution was stirred for 1 h. The reaction was quenched with saturated aqueous sodium thiosulfate and ammonium chloride aqueous solution. Extraction was repeated a total of 3 times with ethyl acetate, and combined organic phase was then evaporated in vacuo. The residue was purified by silica gel column chromatography on neutral silica gel with hexane/ethyl acetate (10/1 → 5/1, v/v) to give *tert*-butyl 2-(dibenzylamino)-3-hydroxy-5-phenylpentanoate (**5**, 43.5 mg, 0.098 mmol) in 98% yield with >99:1 *anti/syn* ratio. The enantiomeric ratio (er) was determined to be >99:1 by chiral HPLC analysis on a chiral stationary phase.

**Homologation of 3aa-O$^t$Bu (Scheme 5)**

To a solution of *tert*-butyl 2-(dibenzylamino)-5-phenyl-3-(4,4,5,5-tetramethyl-1,3,2-dioxaborolan-2-yl)pentanoate (**3aa-O$^t$Bu**, 55.6 mg, 0.10 mmol, >99:1 *anti*/*syn*, >99:1 er) and

bromochloromethane (25.9 mg, 0.20 mmol) in THF (1.0 mL) at -78 °C was added *n*-BuLi (1.56 M hexane solution, 0.11 mL, 0.17 mmol), and the solution was stirred at the same temperature for 30 min. The mixture was allowed to warm to room temperature over 30 min and then heated at 60 °C for additional 3 h. The resulting mixture was quenched with $NH_4Cl$ and extracted with ethyl acetate. The combined organic layer was dried over $Na_2SO_4$, and the solvent was removed under reduced pressure. The residue was purified by silica gel column chromatography on neutral silica gel with hexane/ethyl acetate (10/1, v/v) to give *tert*-butyl 2-(dibenzylamino)-5-phenyl-3-((4,4,5,5-tetramethyl-1,3,2-dioxaborolan-2-yl)methyl)pentanoate (**6**, 47.6 mg, 0.084 mmol) in 84% yield with >99:1 *anti/syn* ratio. The enantiomeric ratio (er) was determined to be 99:1 by chiral HPLC analysis on a chiral stationary phase.

**Vinylation of 3aa-O$^t$Bu (Scheme 5)**

To a solution of *tert*-butyl 2-(dibenzylamino)-5-phenyl-3-(4,4,5,5-tetramethyl-1,3,2-dioxaborolan-2-yl)pentanoate (**3aa-O$^t$Bu**, 55.6 mg, 0.10 mmol, >99:1 *anti*/*syn*, >99:1 er) in THF (1.0 mL) at -78 °C was added vinylmagnesium bromide (1.0 M in THF, 0.40 mmol) dropwise. The resulting mixture was stirred at room temperature for 30 min and cooled down to -78 °C. A solution of iodine (101.5 mg, 0.40 mmol) in MeOH (0.4 mL) was added dropwise to the reaction mixture, followed 1 h later by a solution of NaOMe (43.2 mg, 0.80 mmol) in MeOH (0.8 mL). The reaction mixture was then allowed to warm to room temperature and stirred for an additional 18 h, and the reaction was quenched with saturated aqueous sodium thiosulfate. Extraction was repeated a total of 3 times with ethyl acetate, and combined organic phase was then evaporated in vacuo. The residue was purified by silica gel column chromatography on neutral silica gel with hexane/ethyl acetate (20/1, v/v) to give *tert*-butyl 2-(dibenzylamino)-3-phenethylpent-4-enoate (**7**, 42.2 mg, 0.093 mmol) in 93% yield with >99:1 *anti/syn* ratio. The enantiomeric ratio (er) was determined to be >99:1 by chiral HPLC analysis on a chiral stationary phase.

**Furanylation of 3aa-O$^t$Bu (Scheme 5)**

A solution of furan (13.6 mg, 0.20 mmol) in THF (0.6 mL) was cooled to -78 °C and treated with *n*-BuLi (1.56 M hexane solution, 0.096 mL, 0.15 mmol). The cooling bath was removed and the mixture was stirred at room temperature for 1 h. The mixture was again cooled to -78 °C, and a solution of *tert*-butyl 2-(dibenzylamino)-5-phenyl-3-(4,4,5,5-tetramethyl-1,3,2-dioxaborolan-2-yl)pentanoate (**3aa-O$^t$Bu**, 55.6 mg, 0.10 mmol, >99:1 *anti*/*syn*, >99:1 er) in THF (0.4 mL) was added dropwise. The mixture was stirred at -78 °C for 1 h. A solution of NBS (26.7 mg, 0.15 mmol) in THF (0.6 mL) was

added dropwise. After 3 h at –78 °C, the reaction mixture was then allowed to warm to –20 °C and stirred for an additional 12 h. The reaction was quenched with saturated aqueous sodium thiosulfate. Extraction was repeated a total of 3 times with ethyl acetate, and combined organic phase was then evaporated in vacuo. The residue was purified by silica gel column chromatography on neutral silica gel with hexane/ethyl acetate (40/1 → 20/1, v/v) to give *tert*-butyl 2-(dibenzylamino)-3-(furan-2-yl)-5-phenylpentanoate (**8**, 27.8 mg, 0.056 mmol) in 56% yield with >99:1 *anti/syn* ratio. The enantiomeric ratio (er) was determined to be >99:1 by chiral HPLC analysis on a chiral stationary phase.

**Hydrogenolysis of 3aa-O$^t$Bu (Scheme 5)**

A 20 mL two-necked reaction flask, equipped with a stir bar was charged with *tert*-butyl 2-(dibenzylamino)-5-phenyl-3-(4,4,5,5-tetramethyl-1,3,2-dioxaborolan-2-yl)pentanoate (**3aa-O$^t$Bu**, 55.6 mg, 0.10 mmol, >99:1 *anti*/*syn*, >99:1 er), $Pd(OH)_2$ on carbon (20 wt%, 11.1 mg), and MeOH (1.0 mL). The flask was evacuated and backfilled with hydrogen (this process was repeated a total of 3 times), and the suspension was stirred at room temperature for 24 h under hydrogen atmosphere (1 atm, balloon). The reaction flask was then evacuated and backfilled with $N_2$. The resulting mixture was filtered through a pad of Celite, and then evaporated in vacuo to give *tert*-butyl 2-amino-5-phenyl-3-(4,4,5,5-tetramethyl-1,3,2-dioxaborolan-2-yl)pentanoate (**9**, 37.0 mg, 0.099 mmol) in 99% yield with >99:1 *anti/syn* ratio. The enantiomeric ratio (er) was determined to be >99:1 by chiral HPLC analysis on a chiral stationary phase.

## Detailed Optimisation Studies

***Table S1.*** Optimisation studies for Cu-catalysed regio- and diastereoselective borylamination of *α*,*β*-unsaturated ester **1a** with B$_2$pin$_2$ and *N*,*N*-dibenzylhydroxylamine **2a**[a]

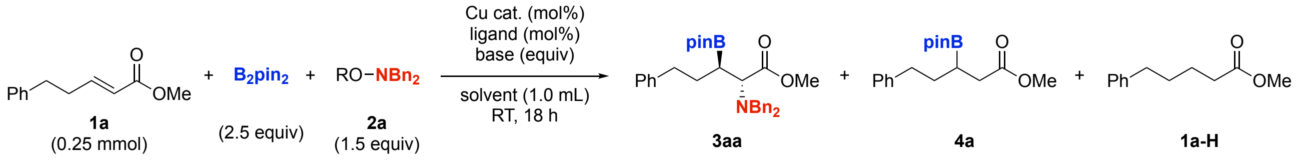


| entry | 2a | Cu cat. (mol%) | ligand (mol%) | base (equiv) | solvent | NMR yield (%) | | | | | d.r. |
|---|---|---|---|---|---|---|---|---|---|---|---|
| | | | | | | **3aa** | **4a** | **1a-H** | **1a** | **2a** | |
| 1 | **2a-Piv** | Cu(OAc)$_2$ · H$_2$O (12) | PPh$_3$ (24) | CsOPiv (3.0) | 1,4-dioxane | 65 | 29 | 4 | 0 | 3 | 97:3 |
| 2 | **2a-Piv** | Cu(OAc)$_2$ · H$_2$O (12) | PPh$_3$ (24) | CsOPiv (3.0) | 1,4-dioxane | 50 | 34 | 6 | 0 | 17 | 95:5 |
| 3 | **2a-Piv** | Cu(OAc)$_2$ · H$_2$O (12) | dppbz (12) | CsOPiv (3.0) | 1,4-dioxane | 16 | 49 | 5 | 0 | 34 | 94:6 |
| 4 | **2a-Piv** | Cu(OAc)$_2$ · H$_2$O (12) | dppe (12) | CsOPiv (3.0) | 1,4-dioxane | 31 | 54 | 5 | 0 | 27 | 96:4 |
| 5 | **2a-Piv** | Cu(OAc)$_2$ · H$_2$O (12) | Xantphos (12) | CsOPiv (3.0) | 1,4-dioxane | 0 | 18 | 0 | 78 | 139 | - |
| 6 | **2a-Piv** | Cu(OAc)$_2$ · H$_2$O (12) | IPr · HCl (12) | CsOPiv (3.0) | 1,4-dioxane | 0 | 58 | 0 | 38 | 140 | - |
| 7 | **2a-Piv** | Cu(OAc)$_2$ · H$_2$O (12) | bpy (12) | CsOPiv (3.0) | 1,4-dioxane | 0 | 63 | 2 | 27 | 0 | - |
| 8 | **2a-Piv** | Cu(OAc)$_2$ · H$_2$O (12) | P(4-MeC$_6$H$_4$)$_3$ (24) | CsOPiv (3.0) | 1,4-dioxane | 55 | 34 | 6 | 0 | 12 | 97:3 |
| 9 | **2a-Piv** | Cu(OAc)$_2$ · H$_2$O (12) | P(4-$^t$BuC$_6$H$_4$)$_3$ (24) | CsOPiv (3.0) | 1,4-dioxane | 47 | 37 | 6 | 0 | 15 | 96:4 |
| 10 | **2a-Piv** | Cu(OAc)$_2$ · H$_2$O (12) | P(4-MeOC$_6$H$_4$)$_3$ (24) | CsOPiv (3.0) | 1,4-dioxane | 51 | 38 | 5 | 0 | 10 | 97:3 |
| 11 | **2a-Piv** | Cu(OAc)$_2$ · H$_2$O (12) | P(4-Me$_2$NC$_6$H$_4$)Ph$_2$ (24) | CsOPiv (3.0) | 1,4-dioxane | 57 | 33 | 8 | 0 | 4 | 97:3 |
| 12 | **2a-Piv** | Cu(OAc)$_2$ · H$_2$O (12) | P(4-FC$_6$H$_4$)$_3$ (24) | CsOPiv (3.0) | 1,4-dioxane | 63 | 23 | 5 | 0 | 18 | 98:2 |
| 13 | **2a-Piv** | Cu(OAc)$_2$ · H$_2$O (12) | P(4-ClC$_6$H$_4$)$_3$ (24) | CsOPiv (3.0) | 1,4-dioxane | 65 | 30 | 5 | 0 | 21 | 98:2 |
| 14 | **2a-Piv** | Cu(OAc)$_2$ · H$_2$O (12) | P(4-CF$_3$C$_6$H$_4$)$_3$ (24) | CsOPiv (3.0) | 1,4-dioxane | 52 | 32 | 5 | 0 | 4 | 97:3 |
| 15 | **2a-Piv** | Cu(OAc)$_2$ · H$_2$O (12) | P(3-MeC$_6$H$_4$)$_3$ (24) | CsOPiv (3.0) | 1,4-dioxane | 64 | 36 | 0 | 0 | 0 | 97:3 |
| 16 | **2a-Piv** | Cu(OAc)$_2$ · H$_2$O (12) | P(3,5-$^t$Bu$_2$C$_6$H$_3$)$_3$ (24) | CsOPiv (3.0) | 1,4-dioxane | 50 | 39 | 7 | 0 | 0 | 96:4 |
| 17 | **2a-Piv** | Cu(OAc)$_2$ · H$_2$O (12) | P(3,5-(CF$_3$)$_2$C$_6$H$_3$)$_3$ (24) | CsOPiv (3.0) | 1,4-dioxane | 67 | 26 | 2 | 0 | 2 | 97:3 |
| 18 | **2a-Piv** | Cu(OAc)$_2$ · H$_2$O (12) | P(2-MeC$_6$H$_4$)$_3$ (24) | CsOPiv (3.0) | 1,4-dioxane | 22 | 50 | 6 | 0 | 0 | 93:7 |
| 19 | **2a-Piv** | Cu(OAc)$_2$ · H$_2$O (12) | P(2-furyl)$_3$ (24) | CsOPiv (3.0) | 1,4-dioxane | 37 | 44 | 7 | 0 | 0 | 97:3 |
| 20 | **2a-Piv** | Cu(OAc)$_2$ · H$_2$O (12) | P(3,4,5-F$_3$C$_6$H$_2$)$_3$ (24) | CsOPiv (3.0) | 1,4-dioxane | 68 | 25 | 3 | 0 | 0 | 98:2 |
| 21 | **2a-Piv** | Cu(OAc)$_2$ · H$_2$O (12) | P(C$_6$F$_5$)$_3$ (24) | CsOPiv (3.0) | 1,4-dioxane | 0 | 12 | 0 | 87 | 123 | - |
| 22 | **2a-Piv** | Cu(OAc)$_2$ · H$_2$O (12) | PCyPh$_2$ (24) | CsOPiv (3.0) | 1,4-dioxane | 46 | 37 | 7 | 0 | 17 | 96:4 |
| 23 | **2a-Piv** | Cu(OAc)$_2$ · H$_2$O (12) | P(2-biphenyl)Ph$_2$ (24) | CsOPiv (3.0) | 1,4-dioxane | 64 | 26 | 5 | 0 | 0 | 96:4 |
| 24 | **2a-Piv** | Cu(OAc)$_2$ · H$_2$O (12) | Xphos (24) | CsOPiv (3.0) | 1,4-dioxane | 21 | 50 | 2 | 10 | 9 | 95:5 |
| 25 | **2a-Piv** | Cu(OAc)$_2$ · H$_2$O (12) | Sphos (24) | CsOPiv (3.0) | 1,4-dioxane | 28 | 45 | 0 | 0 | 6 | 94:6 |
| 26 | **2a-Piv** | Cu(OAc)$_2$ · H$_2$O (12) | P(OEt)$_3$ (24) | CsOPiv (3.0) | 1,4-dioxane | 46 | 48 | 3 | 0 | 14 | 96:4 |
| 27 | **2a-Piv** | Cu(OAc)$_2$ · H$_2$O (12) | P(O-2,4-$^t$Bu$_2$C$_6$H$_3$)$_3$ (24) | CsOPiv (3.0) | 1,4-dioxane | 0 | 18 | 0 | 82 | 126 | - |
| 28 | **2a-Piv** | Cu(OAc)$_2$ · H$_2$O (12) | AsPh$_3$ (24) | CsOPiv (3.0) | 1,4-dioxane | 0 | 39 | 2 | 51 | 0 | - |
| 29 | **2a-Piv** | Cu(OAc)$_2$ · H$_2$O (12) | P(O-2,4-$^t$Bu$_2$C$_6$H$_3$)$_3$ (24) | CsOPiv (3.0) | 1,4-dioxane | 0 | 18 | 0 | 82 | 126 | - |
| 30 | **2a-Piv** | Cu(OAc)$_2$ · H$_2$O (12) | CF$_3$-dppbz (12) | CsOPiv (3.0) | 1,4-dioxane | 44 | 49 | 3 | 0 | 28 | 93:7 |
| 31 | **2a-Piv** | Cu(OAc)$_2$ · H$_2$O (12) | F$_3$-dppbz (12) | CsOPiv (3.0) | 1,4-dioxane | 31 | 54 | 4 | 0 | 54 | 97:3 |
| 32 | **2a-Piv** | Cu(OAc)$_2$ · H$_2$O (12) | P(3,4,5-F$_3$C$_6$H$_2$)$_3$ (24) | KOPiv (3.0) | 1,4-dioxane | 66 | 32 | 2 | 0 | 0 | 96:4 |
| 33 | **2a-Piv** | Cu(OAc)$_2$ · H$_2$O (12) | P(3,4,5-F$_3$C$_6$H$_2$)$_3$ (24) | CsOAc (3.0) | 1,4-dioxane | 73 | 24 | 2 | 0 | 0 | 97:3 |

| | | | | | | | | | | | |
|---|---|---|---|---|---|---|---|---|---|---|---|
| 34 | **2a-Piv** | $Cu(OAc)_2 \cdot H_2O$ (12) | P(3,4,5-$F_3C_6H_2$)$_3$ (24) | KOAc (3.0) | 1,4-dioxane | 71 | 27 | 2 | 0 | 0 | 97:3 |
| 35 | **2a-Piv** | $Cu(OAc)_2 \cdot H_2O$ (12) | P(3,4,5-$F_3C_6H_2$)$_3$ (24) | CsF (3.0) | 1,4-dioxane | 45 | 44 | 3 | 0 | 0 | 96:4 |
| 36 | **2a-Piv** | $Cu(OAc)_2 \cdot H_2O$ (12) | P(3,4,5-$F_3C_6H_2$)$_3$ (24) | $Cs_2CO_3$ (3.0) | 1,4-dioxane | 40 | 59 | 0 | 0 | 11 | 97:3 |
| 37 | **2a-Piv** | $Cu(OAc)_2 \cdot H_2O$ (12) | P(3,4,5-$F_3C_6H_2$)$_3$ (24) | $LiO^tBu$ (3.0) | 1,4-dioxane | 53 | 48 | 0 | 0 | 0 | 96:4 |
| 38 | **2a-Piv** | $Cu(OAc)_2 \cdot H_2O$ (12) | P(3,4,5-$F_3C_6H_2$)$_3$ (24) | $NaO^tBu$ (3.0) | 1,4-dioxane | 22 | 78 | 0 | 0 | 0 | 97:3 |
| 39 | **2a-Piv** | $Cu(OAc)_2 \cdot H_2O$ (12) | P(3,4,5-$F_3C_6H_2$)$_3$ (24) | LiOMe (3.0) | 1,4-dioxane | 49 | 38 | 2 | 0 | 0 | 96:4 |
| 40 | **2a-Piv** | $Cu(OAc)_2 \cdot H_2O$ (12) | P(3,4,5-$F_3C_6H_2$)$_3$ (24) | 4-picoline (3.0) | 1,4-dioxane | 65 | 34 | 0 | 0 | 29 | 97:3 |
| 41 | **2a-Piv** | $Cu(OAc)_2 \cdot H_2O$ (12) | P(3,4,5-$F_3C_6H_2$)$_3$ (24) | 2-picoline (3.0) | 1,4-dioxane | 6 | 57 | 0 | 27 | 72 | - |
| 42 | **2a-Piv** | $Cu(OAc)_2 \cdot H_2O$ (12) | P(3,4,5-$F_3C_6H_2$)$_3$ (24) | DMAP (3.0) | 1,4-dioxane | 16 | 79 | 3 | 0 | 0 | 87:13 |
| 43 | **2a-Piv** | $Cu(OAc)_2 \cdot H_2O$ (12) | P(3,4,5-$F_3C_6H_2$)$_3$ (24) | 4-CN-pyridine (3.0) | 1,4-dioxane | 0 | 0 | 0 | 99 | 139 | - |
| 44 | **2a-Piv** | $Cu(OAc)_2 \cdot H_2O$ (12) | P(3,4,5-$F_3C_6H_2$)$_3$ (24) | pyridine (3.0) | 1,4-dioxane | 54 | 44 | 0 | 0 | 46 | 97:3 |
| 45 | **2a-Piv** | $Cu(OAc)_2 \cdot H_2O$ (12) | P(3,4,5-$F_3C_6H_2$)$_3$ (24) | $Et_3N$ (3.0) | 1,4-dioxane | 0 | 74 | 0 | 22 | 102 | - |
| 46 | **2a-Piv** | $Cu(OAc)_2 \cdot H_2O$ (12) | P(3,4,5-$F_3C_6H_2$)$_3$ (24) | - | 1,4-dioxane | 0 | 23 | 0 | 68 | 83 | - |
| 47 | **2a-Piv** | $Cu(OAc)_2 \cdot H_2O$ (12) | P(3,4,5-$F_3C_6H_2$)$_3$ (24) | CsOPiv (4.0) | 1,4-dioxane | 74 | 25 | 2 | 0 | 0 | 98:2 |
| 48 | **2a-Piv** | $Cu(OAc)_2 \cdot H_2O$ (12) | P(3,5-$(CF_3)_2C_6H_3$)$_3$ (24) | CsOPiv (4.0) | 1,4-dioxane | 71 | 27 | 3 | 0 | 0 | 96:4 |
| 49 | **2a-Piv** | $Cu(OAc)_2 \cdot H_2O$ (12) | P(3,4,5-$F_3C_6H_2$)$_3$ (24) | CsOPiv (5.0) | 1,4-dioxane | 61 | 30 | 2 | 0 | 0 | 97:3 |
| 50 | **2a-Piv** | $Cu(OAc)_2 \cdot H_2O$ (12) | P(3,5-$F_2C_6H_3$)$_3$ (24) | CsOPiv (3.0) | 1,4-dioxane | 76 | 21 | 2 | 0 | 0 | 96:4 |
| 51 | **2a-Piv** | $Cu(OAc)_2 \cdot H_2O$ (12) | P(3,5-$F_2C_6H_3$)$_3$ (24) | CsOAc (3.0) | 1,4-dioxane | 71 | 26 | 2 | 0 | 0 | 98:2 |
| 52 | **2a-Piv** | $Cu(OAc)_2 \cdot H_2O$ (12) | P(3,5-$F_2C_6H_3$)$_3$ (24) | 4-picoline (3.0) | 1,4-dioxane | 65 | 34 | 2 | 0 | 28 | 98:2 |
| 53 | **2a-Piv** | $Cu(OAc)_2 \cdot H_2O$ (12) | P(3,5-$F_2C_6H_3$)$_3$ (24) | CsOPiv (4.0) | 1,4-dioxane | 76 | 21 | 2 | 0 | 0 | 97:3 |
| 54 | **2a-Piv** | $Cu(OAc)_2 \cdot H_2O$ (12) | P(3,5-$F_2C_6H_3$)$_3$ (24) | CsOAc (4.0) | 1,4-dioxane | 71 | 27 | 3 | 0 | 0 | 97:3 |
| 55 | **2a-Piv** | $Cu(OAc)_2 \cdot H_2O$ (12) | P(3,5-$F_2C_6H_3$)$_3$ (24) | CsOPiv (2.0)<br>4-picoline (2.0) | 1,4-dioxane | 75 | 21 | 3 | 0 | 0 | 98:2 |
| 56 | **2a-Bz** | $Cu(OAc)_2 \cdot H_2O$ (12) | P(3,5-$F_2C_6H_3$)$_3$ (24) | CsOPiv (3.0) | 1,4-dioxane | 50 | 45 | 5 | 0 | 7 | 96:4 |
| 57 | **2a-Ac** | $Cu(OAc)_2 \cdot H_2O$ (12) | P(3,5-$F_2C_6H_3$)$_3$ (24) | CsOPiv (3.0) | 1,4-dioxane | 61 | 34 | 2 | 0 | 8 | 97:3 |
| 58 | **2a-NMe$_2$** | $Cu(OAc)_2 \cdot H_2O$ (12) | P(3,5-$F_2C_6H_3$)$_3$ (24) | CsOPiv (3.0) | 1,4-dioxane | 64 | 36 | 1 | 0 | 12 | 97:3 |
| 59 | **2a-Ad** | $Cu(OAc)_2 \cdot H_2O$ (12) | P(3,5-$F_2C_6H_3$)$_3$ (24) | CsOAc (3.0) | 1,4-dioxane | 0 | 0 | 0 | 86 | 106 | - |
| 60 | **2a-Piv** | $Cu(OAc)_2$ (12) | P(3,5-$F_2C_6H_3$)$_3$ (24) | CsOPiv (3.0) | 1,4-dioxane | 80 | 18 | 2 | 0 | 0 | 97:3 |
| 61 | **2a-Piv** | $Cu(OTf)_2$ (12) | P(3,5-$F_2C_6H_3$)$_3$ (24) | CsOPiv (3.0) | 1,4-dioxane | 61 | 36 | 3 | 0 | 7 | 97:3 |
| 62 | **2a-Piv** | $Cu(OPiv)_2$ (12) | P(3,5-$F_2C_6H_3$)$_3$ (24) | CsOPiv (3.0) | 1,4-dioxane | 75 | 21 | 4 | 0 | 0 | 98:2 |
| 63 | **2a-Piv** | CuOAc (12) | P(3,5-$F_2C_6H_3$)$_3$ (24) | CsOPiv (3.0) | 1,4-dioxane | 73 | 25 | 2 | 0 | 0 | 97:3 |
| 64 | **2a-Piv** | $Cu(MeCN)_4BF_4$ (12) | P(3,5-$F_2C_6H_3$)$_3$ (24) | CsOPiv (3.0) | 1,4-dioxane | 75 | 22 | 3 | 0 | 0 | 97:3 |
| 65 | **2a-Piv** | CuCl (12) | P(3,5-$F_2C_6H_3$)$_3$ (24) | CsOPiv (3.0) | 1,4-dioxane | 78 | 21 | 1 | 0 | 0 | 97:3 |
| 66 | **2a-Piv** | - | P(3,5-$F_2C_6H_3$)$_3$ (24) | CsOPiv (3.0) | 1,4-dioxane | 0 | 0 | 0 | 97 | 149 | - |
| 67 | **2a-Piv** | $Cu(OAc)_2$ (12) | P(3,5-$F_2C_6H_3$)$_3$ (24) | CsOPiv (3.0) | THF | 62 | 36 | 0 | 0 | 9 | 97:3 |
| 68 | **2a-Piv** | $Cu(OAc)_2$ (12) | P(3,5-$F_2C_6H_3$)$_3$ (24) | CsOPiv (3.0) | CPME | 71 | 29 | 0 | 0 | 0 | 96:4 |
| 69 | **2a-Piv** | $Cu(OAc)_2$ (12) | P(3,5-$F_2C_6H_3$)$_3$ (24) | CsOPiv (3.0) | DCE | 0 | 23 | 0 | 78 | 148 | - |
| 70 | **2a-Piv** | $Cu(OAc)_2$ (12) | P(3,5-$F_2C_6H_3$)$_3$ (24) | CsOPiv (3.0) | DMF | 0 | 96 | 0 | 0 | 39 | - |
| ***71*** | ***2a-Piv*** | ***$Cu(OAc)_2$ (12)*** | ***P(3,5-$F_2C_6H_3$)$_3$ (24)*** | ***CsOPiv (3.0)*** | ***toluene*** | ***78*** | ***20*** | ***2*** | ***0*** | ***9*** | ***>99:1*** |
| 72 | **2a-Piv** | $Cu(OAc)_2$ (12) | P(3,5-$F_2C_6H_3$)$_3$ (24) | CsOPiv (3.0) | cyclohexane | 55 | 33 | 2 | 0 | 53 | >99:1 |

[a] Reaction conditions: Cu, ligand, **1a** (0.25 mmol), $B_2pin_2$, **2a**, base, solvent (1.0 mL),RT, 18 h, $N_2$. $^1H$ NMR yields based on 0.25 mmol with 1-methylnaphthalene internal standard are shown.

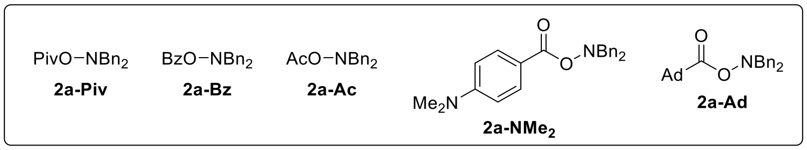


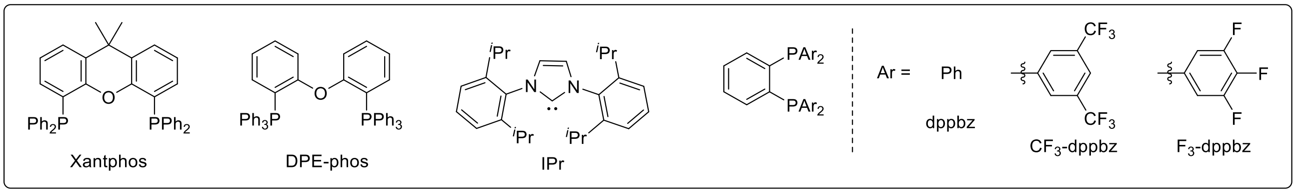


***Table S2.*** Further optimisation studies for Cu-catalysed regio- and diastereoselective borylamination of *α*,*β*-unsaturated ester **1a** with $B_2pin_2$ and *N*,*N*-dibenzylhydroxylamine **2a-Piv**[a]

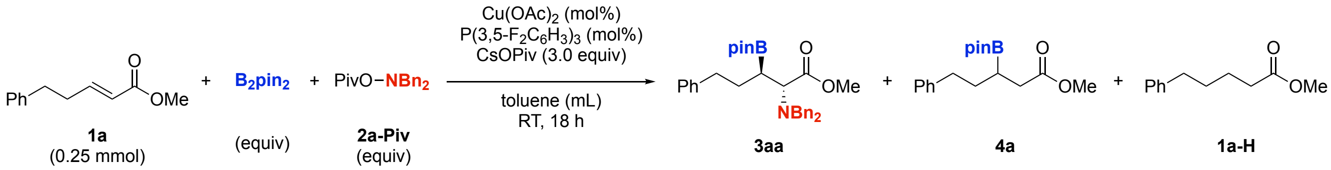


| entry | $B_2pin_2$ (equiv) | **2a-Piv** (equiv) | $Cu(OAc)_2$ (mol%) | $P(3,5\text{-}F_2C_6H_3)_3$ (mol%) | toluene (mL) | NMR yield (%) 3aa | 4a | 1a-H | 1a | 2a-Piv | d.r. |
|---|---|---|---|---|---|---|---|---|---|---|---|
| 1 | **$B_2pin_2$** (2.5) | **2a-Piv** (1.5) | $Cu(OAc)_2$ (12) | $P(3,5\text{-}F_2C_6H_3)_3$ (24) | toluene (1.0) | 78 | 20 | 2 | 0 | 9 | >99:1 |
| 2 | **$B_2pin_2$** (2.5) | **2a-Piv** (1.5) | $Cu(OAc)_2$ (12) | $P(3,5\text{-}F_2C_6H_3)_3$ (24) | toluene (0.5) | 77 | 22 | 2 | 0 | 17 | 97:3 |
| ***3*** | ***$B_2pin_2$ (2.5)*** | ***2a-Piv (1.5)*** | ***$Cu(OAc)_2$ (10)*** | ***$P(3,5\text{-}F_2C_6H_3)_3$ (20)*** | ***toluene (1.0)*** | ***80 (74)*** | ***20*** | ***1*** | ***0*** | ***0*** | ***>99:1*** |
| 4 | **$B_2pin_2$** (2.5) | **2a-Piv** (1.5) | $Cu(OAc)_2$ (5) | $P(3,5\text{-}F_2C_6H_3)_3$ (10) | toluene (1.0) | 74 | 27 | 1 | 0 | 0 | >99:1 |
| 5 | **$B_2pin_2$** (2.0) | **2a-Piv** (1.5) | $Cu(OAc)_2$ (10) | $P(3,5\text{-}F_2C_6H_3)_3$ (20) | toluene (1.0) | 78 | 21 | 2 | 0 | 0 | >99:1 |
| 6 | **$B_2pin_2$** (3.0) | **2a-Piv** (1.5) | $Cu(OAc)_2$ (10) | $P(3,5\text{-}F_2C_6H_3)_3$ (20) | toluene (1.0) | 75 | 24 | 2 | 0 | 0 | >99:1 |
| 7 | **$B_2pin_2$** (2.5) | **2a-Piv** (2.0) | $Cu(OAc)_2$ (10) | $P(3,5\text{-}F_2C_6H_3)_3$ (20) | toluene (1.0) | 73 | 26 | 2 | 0 | 0 | >99:1 |
| 8 | **$B_2pin_2$** (2.5) | **2a-Piv** (1.3) | $Cu(OAc)_2$ (10) | $P(3,5\text{-}F_2C_6H_3)_3$ (20) | toluene (1.0) | 74 | 25 | 2 | 0 | 0 | 98:2 |
| 9[b] | **$B_2pin_2$** (2.5) | **2a-Piv** (1.5) | $Cu(OAc)_2$ (10) | $P(3,5\text{-}F_2C_6H_3)_3$ (20) | toluene (1.0) | 79 | 21 | 2 | 0 | 0 | >99:1 |
| 10 | **$B_2pin_2$** (2.5) | **2a-Piv** (1.5) | $Cu(OAc)_2$ (10) | $P(3,4,5\text{-}F_3C_6H_2)_3$ (20) | toluene (1.0) | 75 | 23 | 2 | 0 | 0 | >99:1 |
| 11[c] | **$B_2pin_2$** (2.5) | **2a-Piv** (1.5) | $Cu(OAc)_2$ (10) | $P(3,5\text{-}F_2C_6H_3)_3$ (20) | toluene (1.0) | 75 | 25 | 0 | 0 | 0 | >99:1 |
| 12[d] | **$B_2pin_2$** (2.5) | **2a-Piv** (1.5) | $Cu(OAc)_2$ (10) | $P(3,5\text{-}F_2C_6H_3)_3$ (20) | toluene (1.0) | 80 | 18 | 0 | 0 | 0 | >99:1 |

[a] Reaction conditions: $Cu(OAc)_2$, ligand, **1a** (0.25 mmol), $B_2pin_2$, **2a-Piv**, CsOPiv (0.75 mmol), toluene, RT, 18 h, $N_2$. $^1$H NMR yields based on 0.25 mmol with 1-methylnaphthalene internal standard are shown. Isolated yield is in parentheses. [b] CsOPiv (4.0 equiv). [c] With MS 4A (50 mg). [d] Set up and reaction with a glovebox filled with rigorously dry nitrogen.

***Table S3.*** Optimisation studies for Cu-catalysed regio- and diastereoselective borylamination of *α*,*β*-unsaturated ester **1b** with $B_2pin_2$ and *N*,*N*-dibenzylhydroxylamine **2a-Piv**: effect of alkoxy moiety of ester on diastereoselectivity[a]

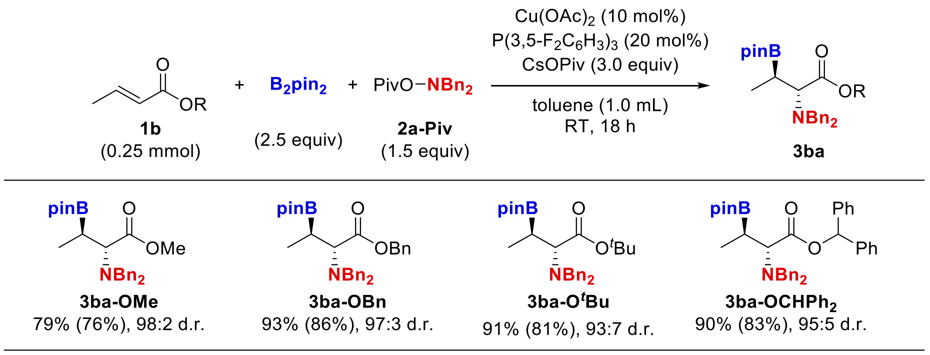


[a] Reaction conditions: $Cu(OAc)_2$ (0.025 mmol), $P(3,5\text{-}F_2C_6H_3)_3$ (0.050 mmol), **1b** (0.25 mmol), $B_2pin_2$ (0.63 mmol), **2a-Piv** (0.38 mmol), CsOPiv (0.75 mmol), toluene (1.0 mL), RT, 18 h, $N_2$. $^1$H NMR yields based on 0.25 mmol with 1-methylnaphthalene internal standard are shown. Isolated yields are in parentheses.

***Table S4.*** Optimisation studies for Cu-catalysed regio- and diastereoselective borylamination of *α*,*β*-unsaturated ester **1b** with diboron and *N*,*N*-dibenzylhydroxylamine **2a-Piv**: effect of diboron on diastereoselectivity[a]

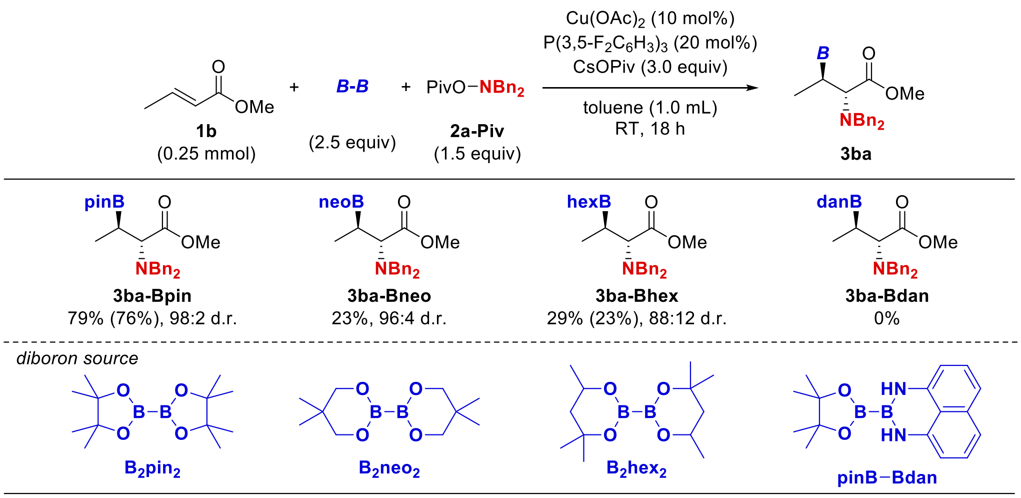


[a] Reaction conditions: $Cu(OAc)_2$ (0.025 mmol), $P(3,5\text{-}F_2C_6H_3)_3$ (0.050 mmol), **1b** (0.25 mmol), diboron (0.63 mmol), **2a-Piv** (0.38 mmol), CsOPiv (0.75 mmol), toluene (1.0 mL), RT, 18 h, $N_2$. $^1$H NMR yields based on 0.25 mmol with 1-methylnaphthalene internal standard are shown. Isolated yields are in parentheses.

***Table S5.*** Optimisation studies for Cu-catalysed regio- and diastereoselective borylamination of *α,β*-unsaturated ester **1a** with $B_2pin_2$ and *N,N*-diethylhydroxylamine **2b**: effect of leaving group of hydroxylamine on yield and diastereoselectivity[a]

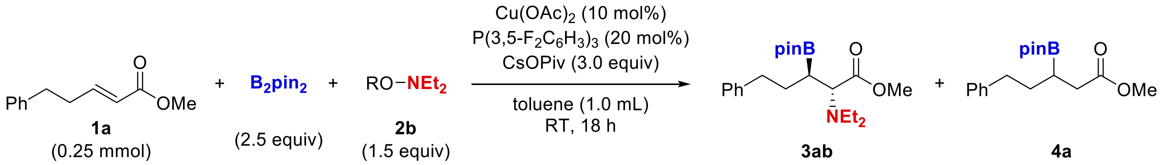


| entry | 2b | NMR yield (%) | | | d.r. |
|---|---|---|---|---|---|
| | | 3ab | 4a | 1a | |
| 1 | **2b-Piv** | 43 | 45 | 0 | 88:12 |
| 2 | **2b-Ad** | 37 | 49 | 0 | 90:10 |
| 3 | **2b-Bz** | 43 | 45 | 0 | 68:32 |
| 4 | **2b-Mes** | 53 | 46 | 0 | 70:30 |
| 5 | **2b-OMe** | 58 | 28 | 0 | 85:15 |
| 6 | **2b-NMe$_2$** | 33 | 34 | 0 | 91:9 |
| 7 | **2b-*o*-MeO** | 50 | 33 | 0 | 81:19 |
| ***8*** | ***2b-(MeO)$_2$*** | ***65 (56)*** | ***27*** | ***0*** | ***90:10*** |
| 9 | **2b-(MeO)$_3$** | 67 | 24 | 0 | 89:11 |

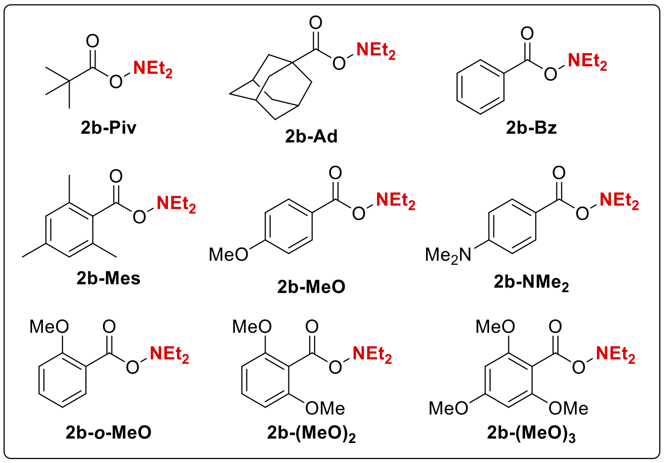


[a] Reaction conditions: $Cu(OAc)_2$ (0.025 mmol), $P(3,5\text{-}F_2C_6H_3)_3$ (0.050 mmol), **1a** (0.25 mmol), $B_2pin_2$ (0.63 mmol), **2b** (0.38 mmol), CsOPiv (0.75 mmol), toluene (1.0 mL), RT, 18 h, $N_2$. $^1H$ NMR yields based on 0.25 mmol with 1-methylnaphthalene internal standard are shown. Isolated yield is in parentheses.

***Table S6.*** Optimisation studies for Cu-catalysed regioselective borylamination of *β,β*-disubstituted *α,β*-unsaturated ester **1A** with $B_2pin_2$ and *N,N*-diethylhydroxylamine **2b**[a]

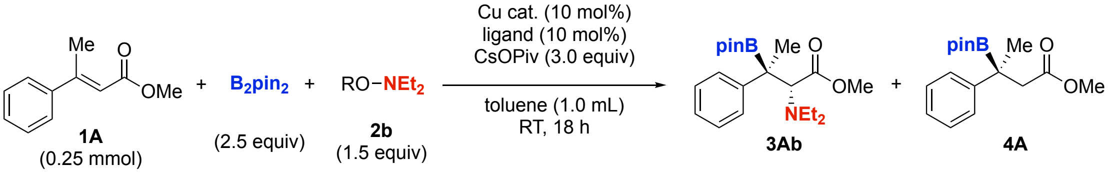


| entry | 2b | Cu cat. | ligand | NMR yield (%) | | | d.r. |
|---|---|---|---|---|---|---|---|
| | | | | 3Ab | 4A | 1A | |
| 1 | **2b-Ac** | $Cu(OAc)_2$ | DTBM-dppbz | 9 (2) | 46 | 13 | 89:11 |
| 2 | **2b-Piv** | $Cu(OAc)_2$ | DTBM-dppbz | < 5 | 81 | 0 | - |
| 3 | **2b-Bz** | $Cu(OAc)_2$ | DTBM-dppbz | 8 | 41 | 14 | 72:28 |
| 4 | **2b-Mes** | $Cu(OAc)_2$ | DTBM-dppbz | 9 | 63 | 0 | 72:28 |
| 5 | **2b-MeO** | $Cu(OAc)_2$ | DTBM-dppbz | 11 | 44 | 16 | 84:16 |

| | | | | | | | |
|---|---|---|---|---|---|---|---|
| 6 | **2b-$NMe_2$** | $Cu(OAc)_2$ | DTBM-dppbz | < 5 | 38 | 30 | - |
| 7 | **2b-$(MeO)_2$** | $Cu(OAc)_2$ | DTBM-dppbz | 40 | 40 | 0 | 80:20 |
| 8 | **2b-$(MeO)_2$** | $Cu(OAc)_2$ | TMS-dppbz | 35 | 44 | 0 | 75:25 |
| 9 | **2b-$(MeO)_2$** | $Cu(OAc)_2$ | $CF_3$-dppbz | < 5 | 51 | 0 | - |
| 10 | **2b-$(MeO)_2$** | $Cu(OAc)_2$ | DMM-dppbz | 0 | 0 | 99 | - |
| 11 | **2b-$(MeO)_2$** | $Cu(OAc)_2$ | *p*-$^t$Bu-dppbz | 14 | 32 | 29 | 80:20 |
| 12 | **2b-$(MeO)_2$** | $Cu(OAc)_2$ | DTBM-dppe | 32 | 41 | 0 | 79:21 |
| 13 | **2b-$(MeO)_2$** | $Cu(OAc)_2$ | $^t$Bu-dppe | 39 | 49 | 0 | 80:20 |
| 14 | **2b-$(MeO)_2$** | $Cu(OAc)_2$ | TMS-dppe | 43 | 48 | 0 | 77:23 |
| 15 | **2b-$(MeO)_2$** | $Cu(OAc)_2$ | $CF_3$-dppe | 0 | 16 | 84 | - |
| 16 | **2b-$(MeO)_2$** | $Cu(OAc)_2$ | Xyl-dppe | 0 | 0 | 98 | - |
| 17 | **2b-$(MeO)_2$** | $Cu(OAc)_2$ | *p*-$^t$Bu-dppe | 0 | 0 | 92 | - |
| 18 | **2b-$(MeO)_2$** | $Cu(OAc)_2$ | dppe | 0 | 0 | 97 | - |
| 19 | **2b-$(MeO)_2$** | $Cu(CH_3CN)_4BF_4$ | TMS-dppe | 44 | 52 | 0 | 69:31 |
| 20[b] | **2b-$(MeO)_2$** | $Cu(CH_3CN)_4BF_4$ | TMS-dppe | 32 | 50 | 0 | 66:34 |
| 21 | **2b-$(MeO)_2$** | $Cu(CH_3CN)_4OTf$ | TMS-dppe | 43 | 40 | 0 | 71:29 |
| 22 | **2b-$(MeO)_2$** | $Cu(OTf)_2$ | TMS-dppe | 21 | 50 | 0 | 71:29 |
| ***23*** | ***2b-$(MeO)_2$*** | ***$Cu(CH_3CN)_4PF_6$*** | ***TMS-dppe*** | ***47 (41)*** | ***44*** | ***0*** | ***70:30*** |
| 24 | **2b-$(MeO)_2$** | CuCl | TMS-dppe | 6 | 38 | 29 | 79:21 |
| 25 | **2b-$(MeO)_2$** | CuBr · $SMe_2$ | TMS-dppe | 20 | 36 | 42 | 80:20 |
| 26 | **2b-$(MeO)_2$** | $Cu(CH_3CN)_4PF_6$ | DTBM-dppbz | 25 | 45 | 7 | 69:31 |

[a] Reaction conditions: Cu (0.025 mmol), ligand (0.025 mmol), **1A** (0.25 mmol), $B_2pin_2$ (0.63 mmol), **2b** (0.38 mmol), CsOPiv (0.75 mmol), toluene (1.0 mL), RT, 18 h, $N_2$. $^1$H NMR yields based on 0.25 mmol with 1-methylnaphthalene internal standard are shown. Isolated yield is in parentheses. [b] 5 $^o$C.

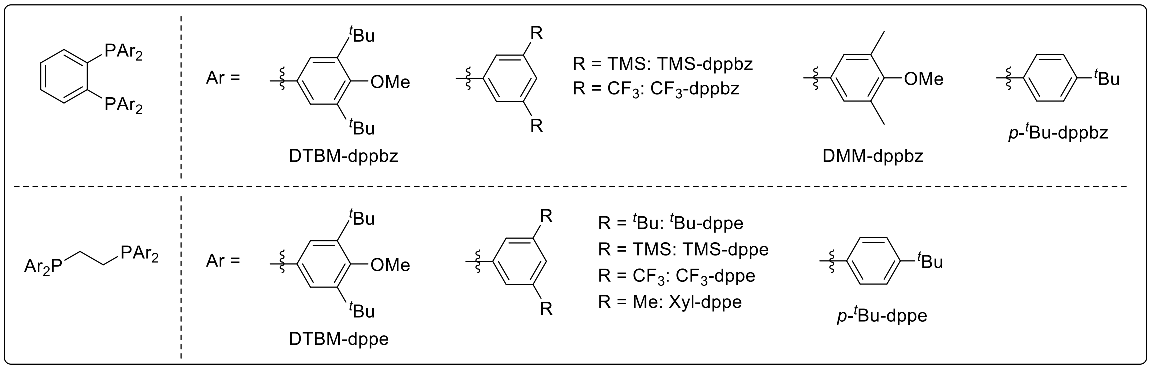

***Table S7.*** Optimisation studies for Cu-catalysed regio-, diastereo-, and enantioselective borylamination of *α*,*β*-unsaturated ester **1b** with $B_2pin_2$ and *N*,*N*-dibenzylhydroxylamine **2a-Piv**[a]

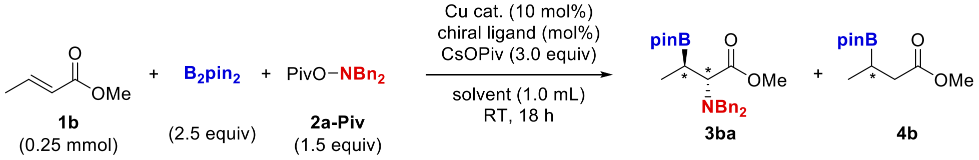


| entry | Cu cat. | chiral ligand (mol%) | solvent | NMR yield (%) | | | d.r. | e.r. |
|---|---|---|---|---|---|---|---|---|
| | | | | **3ba** | **4b** | **2a-Piv** | | |
| 1 | $Cu(OTf)_2$ | (*R*,*R*)-QuinoxP* (10) | toluene | 36 | 43 | 24 | 96:4 | - |
| 2 | $Cu(OAc)_2$ | (*R*,*R*)-QuinoxP* (10) | toluene | 41 (35) | 36 | 40 | 97:3 | 60:40 |
| 3 | $Cu(OAc)_2$ | (*R*,*R*)-QuinoxP* (10) | 1,4-dioxane | 31 (23) | 41 | 70 | 95:5 | 57:43 |
| 4 | $Cu(OAc)_2$ | (*R*,*R*)-BenzP* (10) | 1,4-dioxane | 9 (4) | 32 | 120 | 94:6 | 60:40 |
| 5 | $Cu(OAc)_2$ | (*S*,*S*)-Me-Duphos (10) | 1,4-dioxane | 2 | 54 | 103 | - | - |
| 6 | $Cu(OAc)_2$ | (*R*,*R*)-Ph-BPE (10) | 1,4-dioxane | 51 (42) | 35 | 19 | 94:6 | 53:47 |
| 7 | $Cu(OAc)_2$ | (*S*,*S*)-BDPP (10) | 1,4-dioxane | 40 (31) | 45 | 49 | 94:6 | 53:47 |
| 8 | $Cu(OAc)_2$ | (*S*,*S'*,*R*,*R'*)-Tangphos (10) | 1,4-dioxane | 47 (43) | 50 | 37 | 94:6 | 57:43 |
| 9 | $Cu(OAc)_2$ | (*R*,*R*)-DIOP (10) | 1,4-dioxane | 0 | 41 | 144 | - | - |
| 10 | $Cu(OAc)_2$ | (*R*,*R*)-Dipamp (10) | 1,4-dioxane | 0 | 43 | 120 | - | - |
| 11 | $Cu(OAc)_2$ | (*R*)-MeO-$F_{12}$-BIPHEP (10) | toluene | 37 (27) | 50 | 14 | 89:11 | 52:48 |
| 12 | $Cu(OAc)_2$ | (*R*)-MeO-$F_{12}$-BIPHEP (10) | 1,4-dioxane | 56 (47) | 34 | 28 | 93:7 | 52:48 |
| 13 | $Cu(OAc)_2$ | (*R*)-DTBM-MeO-BIPHEP (10) | 1,4-dioxane | 0 | 35 | 114 | - | - |
| 14 | $Cu(OAc)_2$ | (*R*)-MeO-BIPHEP (10) | 1,4-dioxane | 0 | 20 | 148 | - | - |
| 15 | $Cu(OAc)_2$ | (*R*)-SEGPHOS (10) | 1,4-dioxane | 0 | 34 | 145 | - | - |
| 16 | $Cu(OAc)_2$ | (*R*)-DTBM-SEGPHOS (10) | 1,4-dioxane | 0 | 37 | 150 | - | - |
| 17 | CuCl | (*R*)-DTBM-SEGPHOS (10) | 1,4-dioxane | 0 | 23 | 118 | - | - |
| 18[b] | $Cu(OAc)_2$ | (*R*)-DTBM-SEGPHOS (10) | 1,4-dioxane | 0 | 46 | 0 | - | - |
| 19 | CuCl | (*R*)-DTBM-BINAP (10) | 1,4-dioxane | 0 | 40 | 106 | - | - |
| 20 | $Cu(OAc)_2$ | (*R*)-BINAP (10) | 1,4-dioxane | 0 | 26 | 142 | - | - |
| 21 | $Cu(OAc)_2$ | (*R*)-Difluorophos (10) | 1,4-dioxane | 0 | 12 | 148 | - | - |
| 22 | $Cu(OAc)_2$ | (*R*)-Phanephos (10) | 1,4-dioxane | 45 (36) | 46 | 16 | 93:7 | 53:47 |
| 23 | $Cu(OAc)_2$ | (*R*)-($S_p$)-$PPh_2$-$PCy_2$-Josiphos (10) | 1,4-dioxane | 0 | 25 | 150 | - | - |
| 24 | $Cu(OAc)_2$ | ($R_p$)-(*R*)-Taniaphos (10) | 1,4-dioxane | 0 | 14 | 135 | - | - |
| 25 | $Cu(OAc)_2$ | ($R_p$,$R'_p$)-(*S*)-$CF_3$-Mandyphos (10) | 1,4-dioxane | 52 (43) | 42 | 0 | 94:6 | 59:41 |
| 26 | $Cu(OAc)_2$ | (*R*)-($R_p$)-$CF_3$-Walphos (10) | 1,4-dioxane | 0 | 35 | 134 | - | - |
| 27 | $Cu(OAc)_2$ | (*R*,*R*)-Me-Ferrocelane (10) | 1,4-dioxane | 22 (14) | 45 | 0 | 93:7 | 50:50 |
| 28 | $Cu(OAc)_2$ | (*R*)-MOP (20) | 1,4-dioxane | 0 | 16 | 130 | - | - |
| 29 | $Cu(OAc)_2$ | (*R*)-H-MOP (20) | 1,4-dioxane | 37 (29) | 50 | 0 | 92:8 | 51:49 |
| 30 | $Cu(OAc)_2$ | (*S*)-NMDPP (20) | 1,4-dioxane | 74 (61) | 25 | 0 | 95:5 | 51:49 |
| 31 | $Cu(OAc)_2$ | (*S*)-Monophos (20) | 1,4-dioxane | 76 (70) | 24 | 0 | 94:6 | 55:45 |
| 32 | $Cu(OAc)_2$ | **L1** (20) | 1,4-dioxane | 11 (5) | 79 | 0 | 92:8 | 44:56 |
| 33 | $Cu(OAc)_2$ | **L2** (20) | 1,4-dioxane | 52 (35) | 44 | 27 | 94:6 | 51:49 |
| 34 | $Cu(OAc)_2$ | **L3** (20) | 1,4-dioxane | 0 | 22 | 135 | - | - |
| 35 | $Cu(OAc)_2$ | **L4** (20) | 1,4-dioxane | 0 | 5 | 42 | - | - |
| 36 | $Cu(OAc)_2$ | (*S*)-Siphos (20) | 1,4-dioxane | 68 (59) | 23 | 11 | 95:5 | 62:38 |
| 37 | $Cu(OAc)_2$ | **L5** (20) | 1,4-dioxane | 44 (37) | 42 | 0 | 92:8 | 73:27 |

| | | | | | | | | |
|---|---|---|---|---|---|---|---|---|
| 38 | $Cu(OAc)_2$ | **L5** (20) | toluene | 34 (25) | 37 | 12 | 93:7 | 75:25 |
| 39 | $Cu(OAc)_2$ | **L6** (20) | 1,4-dioxane | 30 (20) | 50 | 9 | 92:8 | 75:25 |
| 40 | $Cu(OAc)_2$ | **L6** (20) | toluene | 0 | 0 | 140 | - | - |
| 41 | $Cu(OAc)_2$ | **L7** (20) | 1,4-dioxane | 0 | 0 | 133 | - | - |
| 42 | $Cu(OAc)_2$ | **L8** (20) | 1,4-dioxane | 41 (40) | 42 | 0 | 93:7 | 51:49 |
| 43 | $Cu(OAc)_2$ | **L9** (20) | 1,4-dioxane | 0 | 20 | 130 | - | - |
| 44 | $Cu(OAc)_2$ | **L10** (20) | 1,4-dioxane | 0 | 0 | 118 | - | - |
| 45 | $Cu(OAc)_2$ | **L11** (20) | 1,4-dioxane | 35 (27) | 39 | 5 | 94:6 | 71:29 |
| 46 | $Cu(OAc)_2$ | **L12** (20) | 1,4-dioxane | 0 | 12 | 139 | - | - |
| 47 | $Cu(OAc)_2$ | **L13** (20) | 1,4-dioxane | 0 | 9 | 122 | - | - |
| 48 | $Cu(OAc)_2$ | **L14** (20) | 1,4-dioxane | 62 (48) | 31 | 0 | 96:4 | 77:23 |
| 49 | $Cu(OAc)_2$ | **L15** (20) | 1,4-dioxane | 43 (35) | 35 | 3 | 94:6 | 73:27 |
| 50 | $Cu(OAc)_2$ | **L16** (20) | 1,4-dioxane | 37 (29) | 46 | 5 | 93:7 | 67:33 |
| 51 | $Cu(OAc)_2$ | **L17** (20) | 1,4-dioxane | 57 (47) | 27 | 4 | 96:4 | 76:24 |
| 52 | $Cu(OAc)_2$ | **L18** (20) | 1,4-dioxane | 0 | 4 | 48 | - | - |
| 53 | $Cu(OAc)_2$ | **L19** (20) | 1,4-dioxane | 34 (26) | 44 | 3 | 93:7 | 73:27 |
| 54 | $Cu(OAc)_2$ | **L20** (20) | 1,4-dioxane | 56 (48) | 33 | 5 | 94:6 | 75:25 |
| 55 | $Cu(OAc)_2$ | **L21** (20) | 1,4-dioxane | 64 (51) | 29 | 0 | 96:4 | 76:24 |
| 56 | $Cu(OAc)_2$ | **L22** (20) | 1,4-dioxane | 0 | 59 | 135 | - | - |
| ***57*** | ***$Cu(OAc)_2$*** | ***L14 (20)*** | ***toluene*** | ***54 (46)*** | ***33*** | ***2*** | ***96:4*** | ***78:22*** |

[a] Reaction conditions: Cu (0.025 mmol), chiral ligand, **1b** (0.25 mmol), $B_2pin_2$ (0.63 mmol), **2a-Piv** (0.38 mmol), CsOPiv (0.75 mmol), solvent (1.0 mL), RT, 18 h, $N_2$. $^1$H NMR yields based on 0.25 mmol with 1-methylnaphthalene internal standard are shown. Isolated yields are in parentheses. [b] LiO$^t$Bu (3.0 equiv).

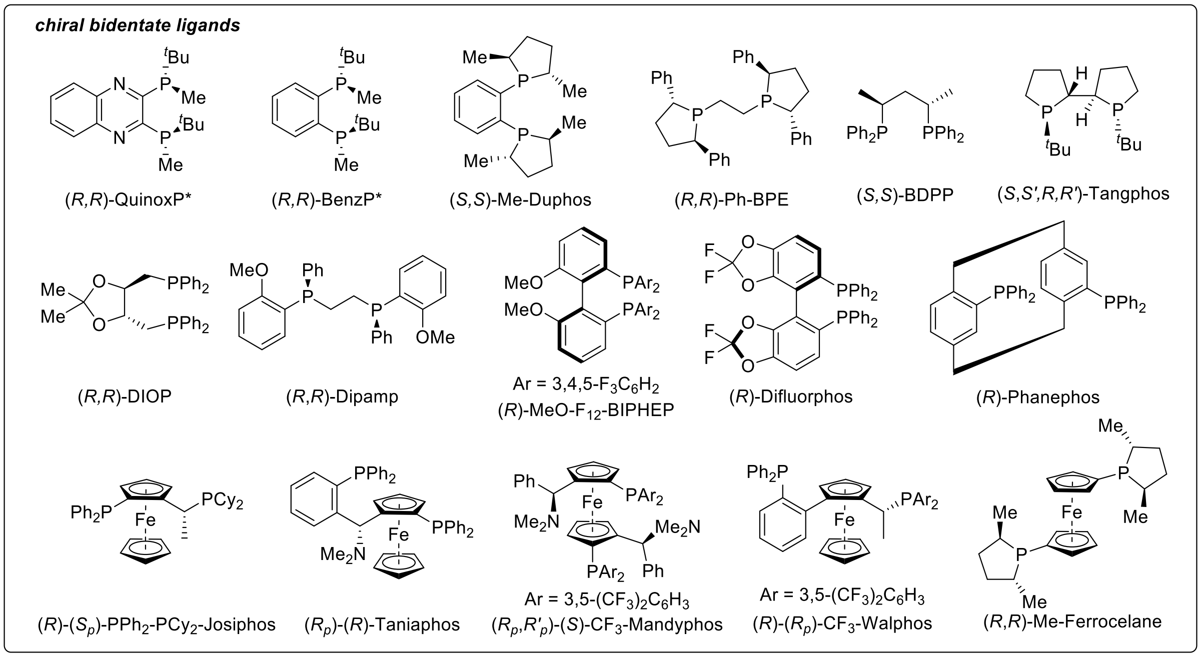

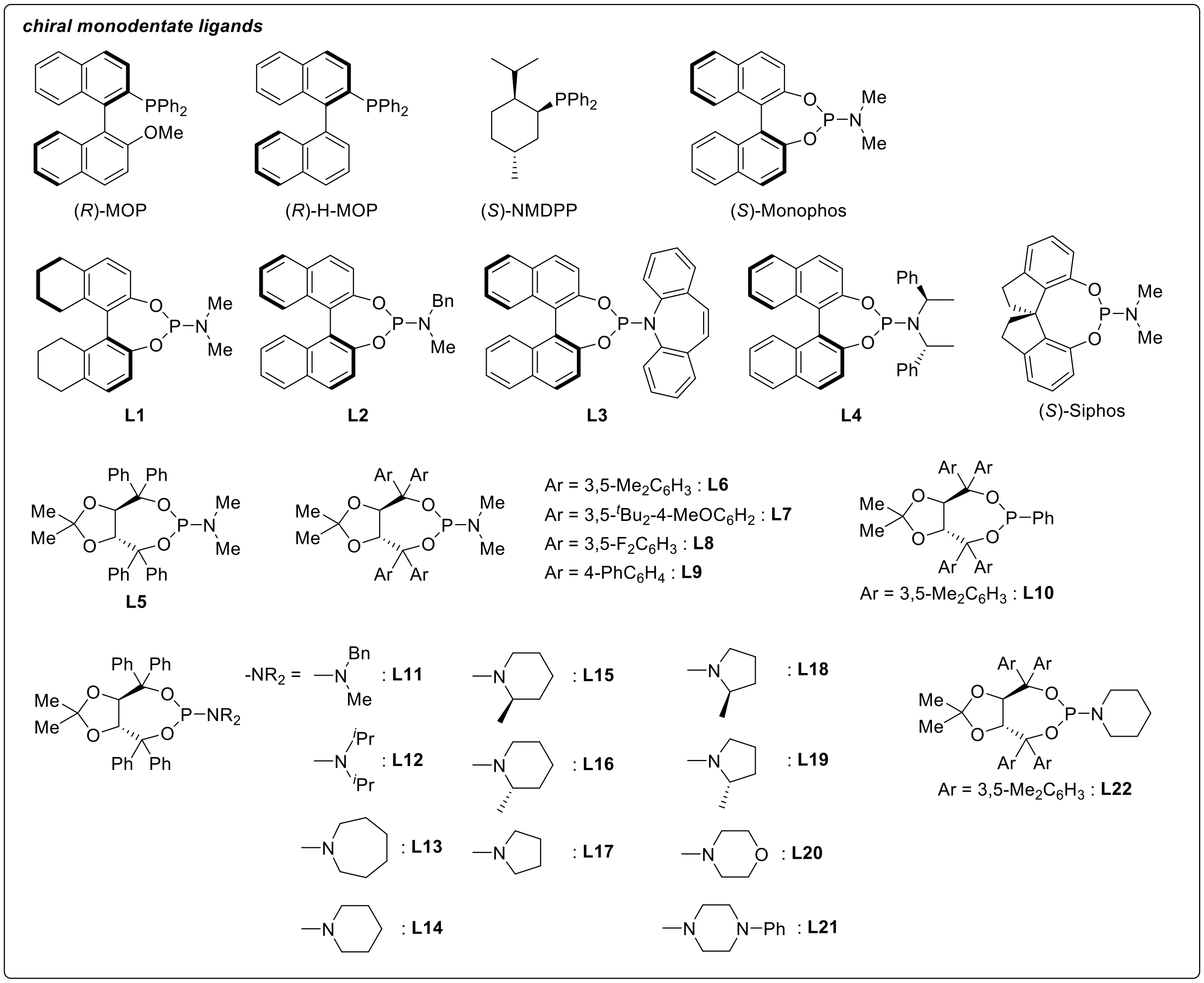


***Table S8.*** Optimisation studies for Cu-catalysed regio-, diastereo-, and enantioselective borylamination of *α*,*β*-unsaturated ester **1** with $B_2pin_2$ and *N*,*N*-dibenzylhydroxylamine **2a-Piv**: effect of alkoxy moiety of ester on diastereoselectivity and enantioselectivity[a]

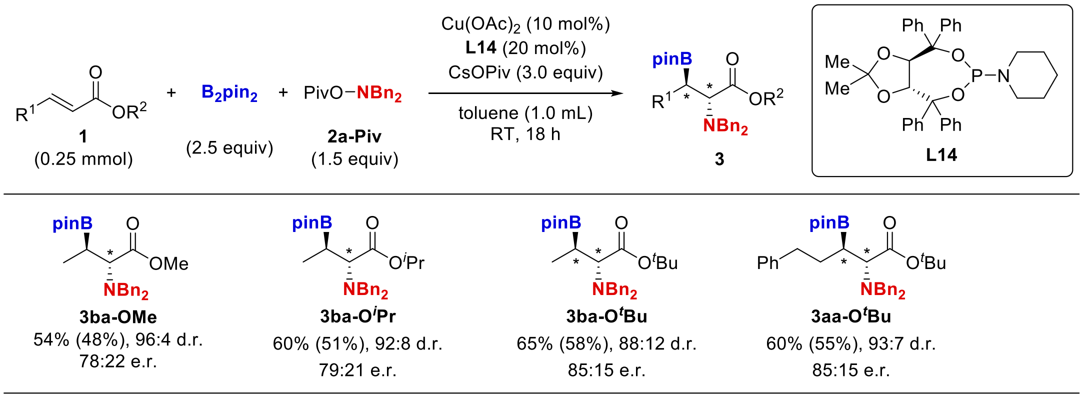

[a] Reaction conditions: Cu(OAc)$_2$ (0.025 mmol), **L14** (0.050 mmol), **1** (0.25 mmol), B$_2$pin$_2$ (0.63 mmol), **2a-Piv** (0.38 mmol), CsOPiv (0.75 mmol), toluene (1.0 mL), RT, 18 h, N$_2$. $^1$H NMR yields based on 0.25 mmol with 1-methylnaphthalene internal standard are shown. Isolated yield is in parentheses.

***Table S9.*** Optimisation studies for Cu-catalysed regio-, diastereo-, and enantioselective borylamination of $\alpha$,$\beta$-unsaturated ester **1a-O$^t$Bu** with B$_2$pin$_2$ and *N*,*N*-dibenzylhydroxylamine **2a**: effects of Cu salts, leaving groups, and temperature[a]

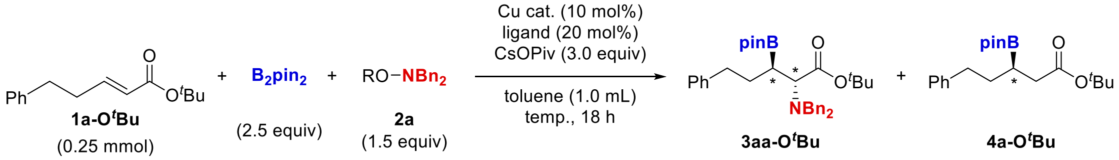


| entry | 2a | Cu cat. | ligand | temp. (°C) | NMR yield (%) 3aa-O$^t$Bu | 4a-O$^t$Bu | 1a-O$^t$Bu | 2a | d.r. | e.r. |
|---|---|---|---|---|---|---|---|---|---|---|
| 1 | **2a-Piv** | Cu(OAc)$_2$ | **L14** | r.t. | 60 (55) | 30 | 7 | 6 | 93:7 | 85:15 |
| 2 | **2a-Piv** | Cu(OAc)$_2$ | **L17** | r.t. | 59 (51) | 28 | 0 | 0 | 93:7 | 82:18 |
| 3 | **2a-Piv** | Cu(OAc)$_2$ | **L20** | r.t. | 64 (57) | 30 | 0 | 12 | 93:7 | 84:16 |
| 4 | **2a-Piv** | Cu(OAc)$_2$ | **L21** | r.t. | 60 (52) | 30 | 0 | 8 | 93:7 | 78:22 |
| 5 | **2a-Bz** | Cu(OAc)$_2$ | **L14** | r.t. | 65 (59) | 26 | 0 | 2 | 91:9 | 87:13 |
| 6 | **2a-MeO** | Cu(OAc)$_2$ | **L14** | r.t. | 64 (56) | 25 | 0 | 0 | 93:7 | 87:13 |
| 7 | **2a-NMe$_2$** | Cu(OAc)$_2$ | **L14** | r.t. | 58 (50) | 32 | 0 | 18 | 95:5 | 83:17 |
| 8 | **2a-Mes** | Cu(OAc)$_2$ | **L14** | r.t. | 59 (52) | 41 | 0 | 48 | 82:18 | 85:15 |
| 9 | **2a-(MeO)$_2$** | Cu(OAc)$_2$ | **L14** | r.t. | 54 (50) | 40 | 0 | 7 | 92:8 | 87:13 |
| 10 | **2a-MeO** | Cu(OAc)$_2$ | **L14** | 15 | 59 (52) | 28 | 0 | 21 | 95:5 | 89:11 |
| 11 | **2a-MeO** | Cu(OAc)$_2$ | **L14** | 5 | 63 (58) | 28 | 0 | 52 | 96:4 | 91:9 |
| 12 | **2a-MeO** | Cu(OAc)$_2$ | **L14** | - 5 | 46 | 33 | 19 | 78 | 96:4 | - |
| 13[b] | **2a-MeO** | Cu(OAc)$_2$ | **L14** | - 5 | 58 (53) | 32 | 0 | 51 | 96:4 | 92:8 |
| 14[c] | **2a-MeO** | Cu(OAc)$_2$ | **L14** | - 5 | 56 | 29 | 5 | 56 | 95:5 | - |
| 15 | **2a-MeO** | CuCl | **L14** | 5 | 74 (68) | 28 | 0 | 52 | 96:4 | 91:9 |
| 16 | **2a-MeO** | CuBr | **L14** | 5 | 73 (69) | 20 | 0 | 52 | 96:4 | 91:9 |
| 17 | **2a-MeO** | CuBr$_2$ | **L14** | 5 | 63 | 22 | 0 | 26 | 96:4 | - |
| 18 | **2a-MeO** | CuBr·SMe$_2$ | **L14** | 5 | 83 (75) | 20 | 0 | 52 | 94:6 | 90:10 |
| 19 | **2a-MeO** | CuI | **L14** | 5 | 66 (58) | 24 | 0 | 51 | 96:4 | 90:10 |
| 20 | **2a-MeO** | Cu(OAc)$_2$·H$_2$O | **L14** | 5 | 55 (47) | 33 | 0 | 32 | 96:4 | 90:10 |
| 21 | **2a-MeO** | Cu(OTf)$_2$ | **L14** | 5 | 62 | 27 | 0 | 32 | 96:4 | - |
| 22 | **2a-MeO** | CuOTf·1/2Tol | **L14** | 5 | 14 | 33 | 45 | 120 | 97:3 | - |
| 23 | **2a-MeO** | Cu(CH$_3$CN)$_4$OTf | **L14** | 5 | 82 (75) | 20 | 0 | 17 | 95:5 | 90:10 |
| 24 | **2a-MeO** | Cu(CH$_3$CN)$_4$BF$_4$ | **L14** | 5 | 82 (78) | 20 | 0 | 22 | 95:5 | 90:10 |
| 25 | **2a-MeO** | Cu(CH$_3$CN)$_4$PF$_6$ | **L14** | 5 | 83 (76) | 19 | 0 | 11 | 95:5 | 89:11 |
| 26 | **2a-MeO** | Cu(CH$_3$CN)$_4$OTf | **L14** | - 5 | 74 (68) | 25 | 0 | 37 | 95:5 | 91:9 |
| ***27*** | ***2a-MeO*** | ***Cu(CH$_3$CN)$_4$BF$_4$*** | ***L14*** | ***- 5*** | ***80 (74)*** | ***22*** | ***0*** | ***31*** | ***95:5*** | ***91:9*** |
| 28 | **2a-MeO** | Cu(CH$_3$CN)$_4$BF$_6$ | **L14** | - 15 | 23 | 24 | 44 | 110 | 97:3 | - |
| 29 | **2a-MeO** | Cu(CH$_3$CN)$_4$PF$_6$ | **L14** | - 5 | 75 (71) | 25 | 0 | 44 | 95:5 | 90:10 |
| 30 | **2a-MeO** | CuBr·SMe$_2$ | **L14** | - 5 | 67 | 23 | 10 | 54 | 95:5 | - |

[a] Reaction conditions: Cu (0.025 mmol), chiral ligand (0.050 mmol), **1a-O$^t$Bu** (0.25 mmol), $B_2pin_2$ (0.63 mmol), **2a** (0.38 mmol), CsOPiv (0.75 mmol), toluene (1.0 mL), 18 h, $N_2$. $^1$H NMR yields based on 0.25 mmol with 1-methylnaphthalene internal standard are shown. Isolated yields are in parentheses. [b] 36 h. [c] Cu cat. (15 mol%), ligand (30 mol%).

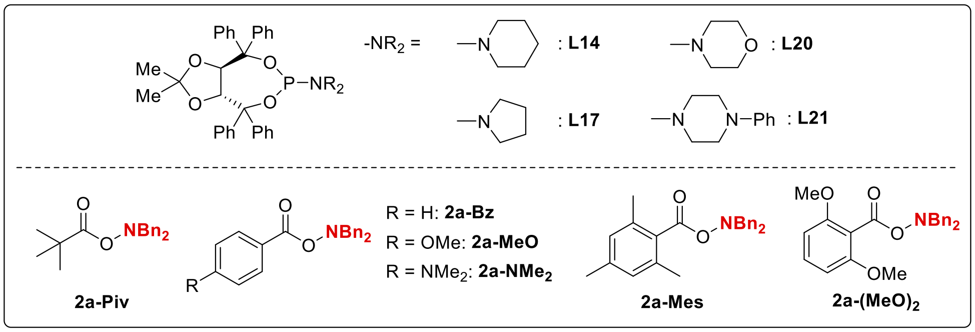


***Table S10.*** Optimisation studies for Cu-catalysed regio-, diastereo-, and enantioselective borylamination of *α*,*β*-unsaturated ester **1b** with $B_2pin_2$ and *N*,*N*-dibenzylhydroxylamine **2a-OMe**: effect of alkoxy moiety of ester on diastereoselectivity and enantioselectivity with conditions of entry 27 in Table S9[a]

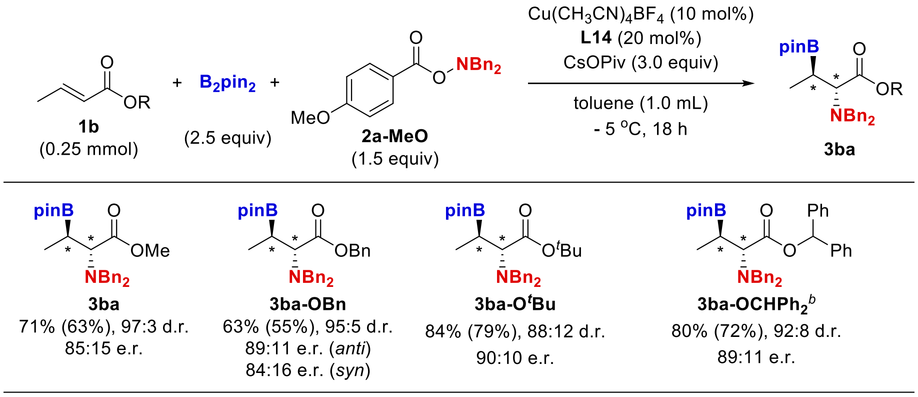


[a] Reaction conditions: $Cu(CH_3CN)_4BF_4$ (0.025 mmol), **L14** (0.050 mmol), **1b** (0.25 mmol), $B_2pin_2$ (0.63 mmol), **2a-OMe** (0.38 mmol), CsOPiv (0.75 mmol), toluene (1.0 mL), -5 °C, 18 h, $N_2$. $^1$H NMR yields based on 0.25 mmol with 1-methylnaphthalene internal standard are shown. Isolated yields are in parentheses. [b] 36 h.

***Table S11.*** Optimisation studies for Cu-catalysed regio- and enantioselective borylamination of *β,β*-disubstituted *α,β*-unsaturated ester **1A** with $B_2pin_2$ and hydroxylamine **2e-(OMe)₂**[a]

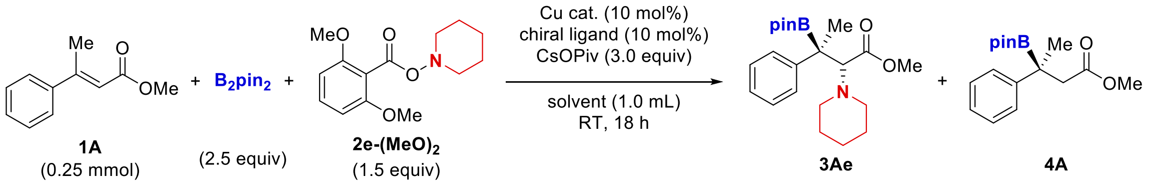

| entry | Cu cat. | chiral ligand | solvent | NMR yield (%) 3Ae | 4A | 1A | d.r. | e.r. |
|---|---|---|---|---|---|---|---|---|
| 1 | $Cu(CH_3CN)_4PF_6$ | (*R*)-Xyl-BINAP (10) | toluene | < 5 | 5 | 51 | - | - |
| 2 | $Cu(CH_3CN)_4PF_6$ | (*R*)-DTBM-BINAP (10) | toluene | 0 | 0 | 85 | - | - |
| 3 | $Cu(CH_3CN)_4PF_6$ | (*R*)-DTBM-SEGPHOS (10) | toluene | 0 | 0 | 88 | - | - |
| 4 | $Cu(CH_3CN)_4PF_6$ | (*R*)-MeO-$F_{12}$-BIPHEP (10) | toluene | 0 | 0 | 86 | - | - |
| 5 | $Cu(CH_3CN)_4PF_6$ | (*R,R*)-QuinoxP* (10) | toluene | 0 | 0 | 91 | - | - |
| 6 | $Cu(CH_3CN)_4PF_6$ | (*S,S*)-Me-Duphos (10) | toluene | 0 | 0 | 83 | - | - |
| 7 | $Cu(CH_3CN)_4PF_6$ | ($R_p$,$R'_p$)-(*S*)-$CF_3$-Mandyphos (10) | toluene | 0 | 0 | 97 | - | - |
| 8 | $Cu(CH_3CN)_4PF_6$ | (*R,R*)-Ph-BPE (10) | toluene | 0 | 0 | 79 | - | - |
| 9 | $Cu(CH_3CN)_4PF_6$ | (*R,R*)-Chiraphos (10) | toluene | 0 | 18 | 68 | - | - |
| 10 | $Cu(CH_3CN)_4PF_6$ | (*R,R*)-DIOP (10) | toluene | < 5 | 10 | 52 | - | - |
| 11 | $Cu(CH_3CN)_4PF_6$ | **L22** (10) | toluene | < 5 | 12 | 67 | - | - |
| 12 | $Cu(CH_3CN)_4PF_6$ | **L23** (10) | toluene | 20 (11) | 17 | 63 | 76:24 | 76:24 |
| 13 | $Cu(CH_3CN)_4PF_6$ | **L24** (10) | toluene | < 5 | 8 | 82 | - | - |
| 14 | $Cu(CH_3CN)_4PF_6$ | (*S,S*)-BDPP (10) | toluene | 57 (50) | 13 | 16 | 77:23 | 82:18 |
| 15 | $Cu(CH_3CN)_4PF_6$ | (*R,R*)-PTBP-BDPP (10) | toluene | 80 (78) | 21 | 0 | 73:27 | 17:83 |
| **16** | **$Cu(CH_3CN)_4PF_6$** | **(*R,R*)-Xyl-BDPP (10)** | **toluene** | **80 (75)** | **14** | **0** | **75:25** | **14:86** |
| 17 | $Cu(CH_3CN)_4PF_6$ | (*R,R*)-$^t$Bu-BDPP (10) | toluene | 46 (38) | 29 | 8 | 73:27 | 36:64 |
| 18 | $Cu(CH_3CN)_4PF_6$ | (*S,S*)-DTBM-BDPP (10) | toluene | 55 (43) | 24 | 7 | 76:24 | 63:37 |
| 19[b] | $Cu(CH_3CN)_4PF_6$ | (*R,R*)-Xyl-BDPP (10) | toluene | < 5 | < 5 | 69 | - | - |
| 20[c] | $Cu(CH_3CN)_4PF_6$ | (*S,S*)-BDPP (10) | toluene | 34 (25) | 25 | 30 | 75:25 | 81:19 |
| 21 | $Cu(CH_3CN)_4BF_4$ | (*R,R*)-Xyl-BDPP (10) | toluene | 74 (68) | 24 | 0 | 74:26 | 14:86 |
| 22 | $Cu(CH_3CN)_4OTf$ | (*R,R*)-Xyl-BDPP (10) | toluene | 76 (74) | 11 | 0 | 75:25 | 15:85 |
| 23 | $CuBr·SMe_2$ | (*R,R*)-Xyl-BDPP (10) | toluene | 0 | 31 | 61 | - | - |
| 24 | $Cu(OAc)_2$ | (*R,R*)-Xyl-BDPP (10) | toluene | 74 (65) | 12 | 0 | 62:38 | 14:86 |
| 25 | $Cu(CH_3CN)_4PF_6$ | (*R,R*)-Xyl-BDPP (10) | 1,4-dioxane | 60 (53) | 13 | 15 | 60:40 | 15:85 |
| 26 | $Cu(CH_3CN)_4PF_6$ | (*R,R*)-Xyl-BDPP (10) | CPME | 68 (67) | 23 | 0 | 71:29 | 14:86 |

[a] Reaction conditions: Cu (0.025 mmol), chiral ligand (0.025 mmol), **1A** (0.25 mmol), $B_2pin_2$ (0.63 mmol), **2e-(OMe)₂** (0.38 mmol), CsOPiv (0.75 mmol), solvent (1.0 mL), RT, 18 h, $N_2$. $^1$H NMR yields based on 0.25 mmol with 1-methylnaphthalene internal standard are shown. Isolated yields are in parentheses. [b] With *tert*-butyl ester, **1A-O$^t$Bu**. [c] 5 °C, 36 h.

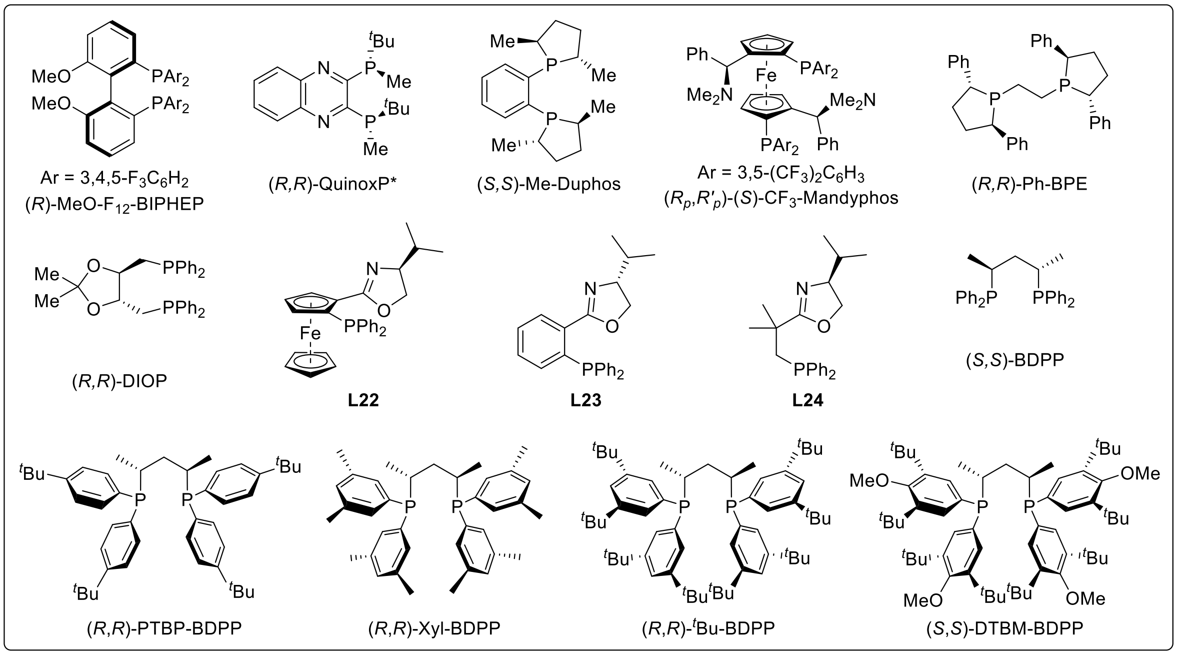


***Table S12.*** Optimisation studies for Cu-catalysed regio-, diastereo-, and enantioselective borylamination of cyclobutenecarboxylate ester **1v** with $B_2pin_2$ and *N*,*N*-dibenzylhydroxylamine **2a-OMe**[a]

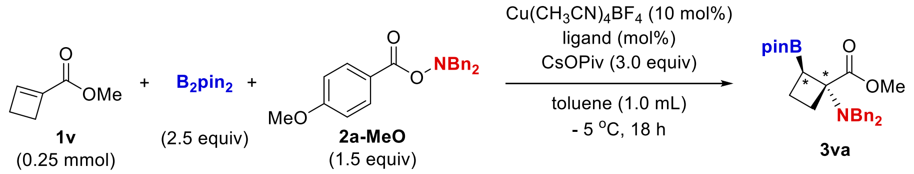


| entry | ligand (mol%) | NMR yield of **3va** (%) | d.r. | e.r. |
|---|---|---|---|---|
| 1 | **L14** (20) | 56 (53) | >99:1 | 67:33 |
| 2 | **L23** (10) | 40 (37) | >99:1 | 41:59 |
| 3 | (*R*,*R*)-Xyl-BDPP (10) | 29 (22) | >99:1 | 56:44 |
| 4 | (*R*,*R*)-Ph-BPE (10) | 0 | - | - |
| 5 | (*R*)-MeO-F$_{12}$-BIPHEP (10) | 0 | - | - |

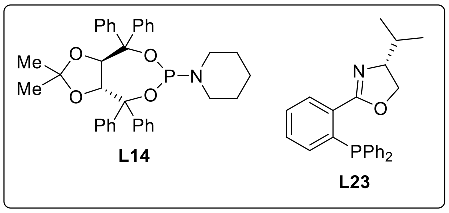


[a] Reaction conditions: $Cu(CH_3CN)_4BF_4$ (0.025 mmol), chiral ligand, **1v** (0.25 mmol), $B_2pin_2$ (0.63 mmol), **2a-OMe** (0.38 mmol), CsOPiv (0.75 mmol), toluene (1.0 mL), -5 °C, 18 h, $N_2$. $^1$H NMR yields based on 0.25 mmol with 1-methylnaphthalene internal standard are shown. Isolated yields are in parentheses.

## Substrate Limitation

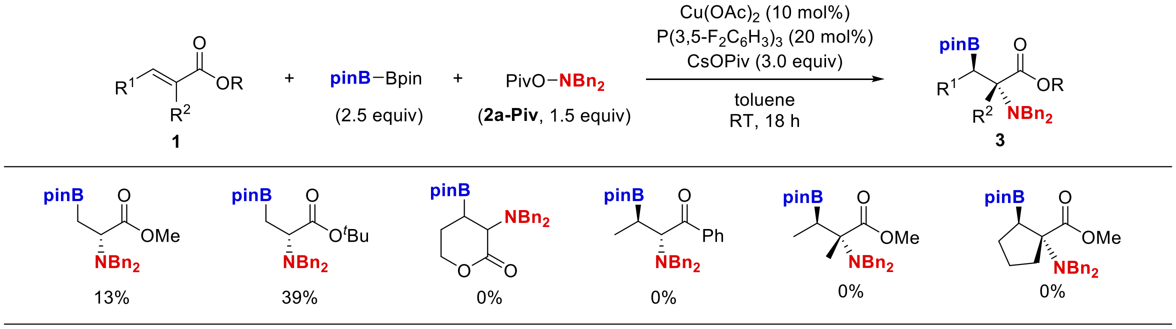


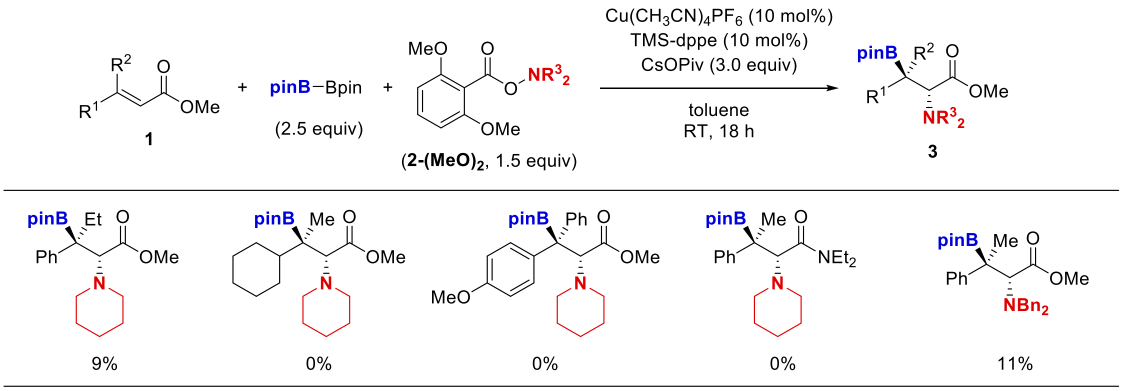

## Stereochemical Assignment

### Assignment of Relative Stereochemistry of 3ba (Scheme 2)

The relative stereochemistry of major diastereomer of **3ba** (Scheme 2) was determined to be *anti* by comparison of $^1$H NMR with the reported values[S10] after the oxidation (Scheme S1). Additionally, X-ray analysis also indicated that the major isomer of **3ba** was *anti* (see Figure S1).

***Scheme S1.*** Oxidation and determination of relative stereochemistry of **3ba**

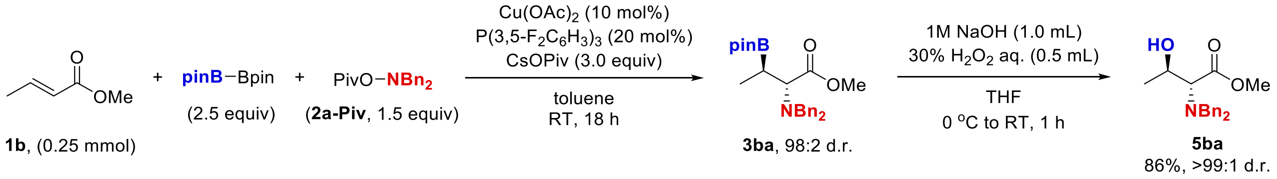


### Assignment of Absolute Configuration of 3ba

The absolute configuration of major isomer of **3ba** was determined by comparison of HPLC chart with the authentic sample after the derivatisation (Scheme S2).

***Scheme S2.*** Determination of absolute configuration of **3ba** and preparation of authentic sample

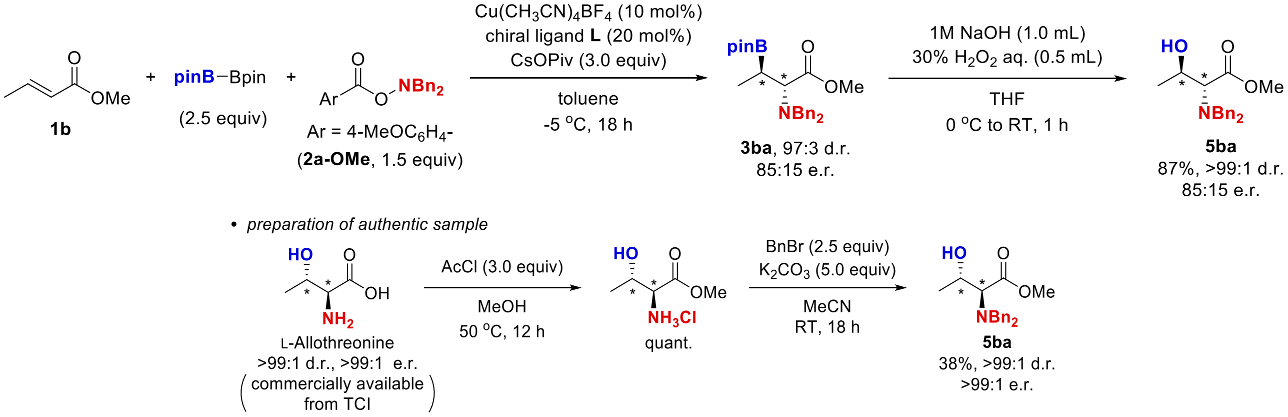


The authentic sample was prepared according to the literature.[S11]

## X-Ray Analysis

The X-ray quality crystals of **3ba** were grown from hexane.

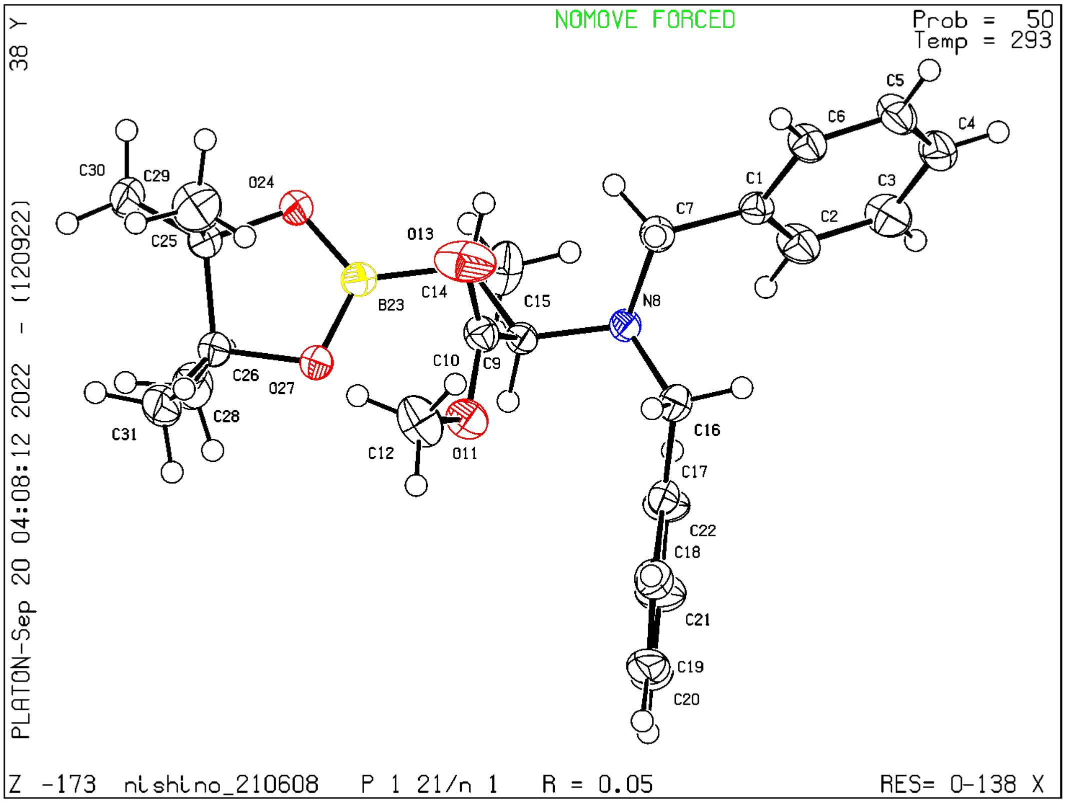


***Figure S1.*** ORTEP drawing of **3ba** (CCDC 2206174).

***Table S13.*** Crystal Data for **3ba**

| | |
|---|---|
| Crystal system | monoclinic |
| Space group IT number | 14 |
| Space group name H-M alt | P 1 21/n 1 |
| Space group name Hall | -P 2yn |
| Cell length a | 9.4689(2) |
| Cell length b | 20.7919(4) |
| Cell length c | 12.8601(3) |
| Cell angle alpha | 90 |
| Cell angle beta | 109.311(2) |
| Cell angle gamma | 90 |

| | |
|---|---|
| Cell volume | 2389.40(9) |
| Cell formula units Z | 4 |
| Refine ls R factor all | 0.0493 |
| Refine ls R factor gt | 0.0462 |
| Refine ls wR factor gt | 0.1198 |
| Refine ls wR factor ref | 0.1221 |
| Refine ls goodness of fit ref | 1.049 |

**Chiral HPLC Charts of Enantioenriched Products**

**3aa-O$^t$Bu**: The enantiomeric ratio was determined by HPLC analysis in comparison with authentic racemic material (CHIRALPAK AD-H column, 98.5/1.5 hexane/isopropyl alcohol, 0.5 mL/min, major isomers: $t_R$ = 8.9, 17.1 min, minor isomers: $t_R$ = 11.9, 15.6 min, UV detection at 210 nm, 30 °C).

*rac*-**3aa-O$^t$Bu**

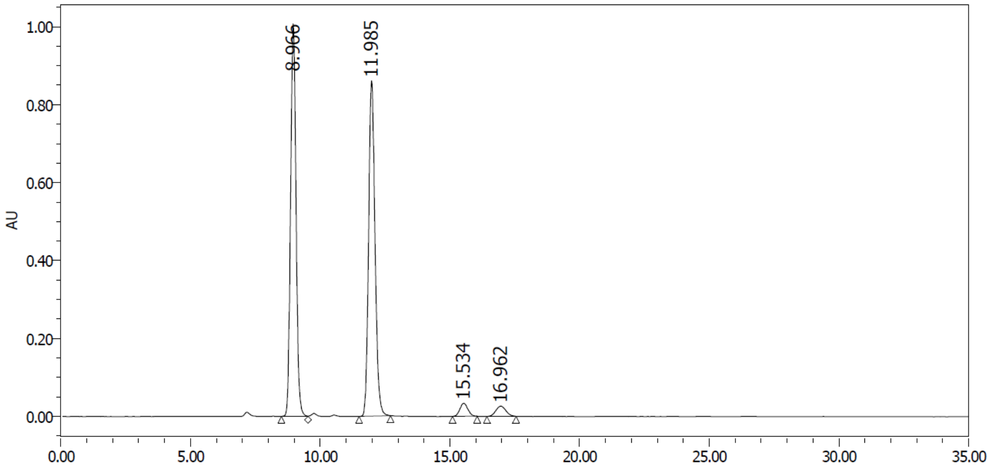


| Peak # | Ret. Time | Area | Area % |
|---|---|---|---|
| 1 | 8.966 | 14721901 | 47.69 |
| 2 | 11.985 | 14726129 | 47.71 |
| 3 | 15.534 | 711071 | 2.30 |
| 4 | 16.962 | 709046 | 2.30 |

*chiral*-**3aa-O$^t$Bu**

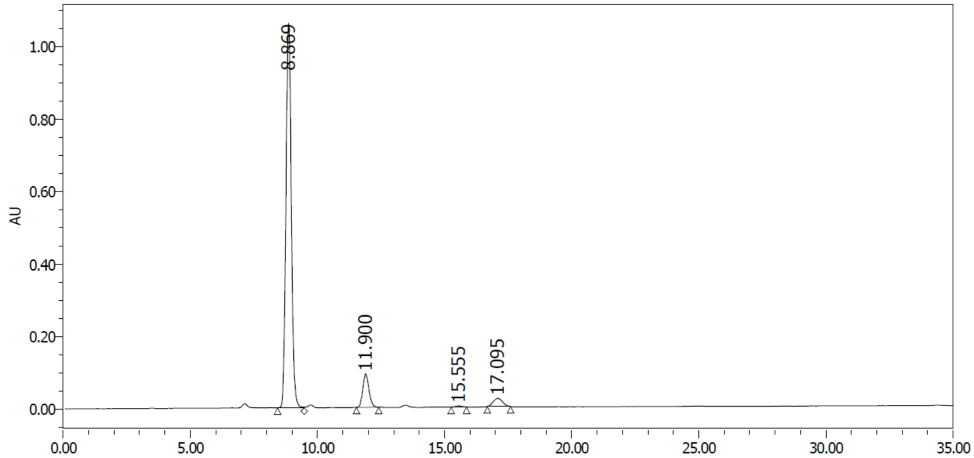


| Peak # | Ret. Time | Area | Area % |
|---|---|---|---|
| 1 | 8.869 | 15517518 | 87.74 |
| 2 | 11.900 | 1537418 | 8.69 |
| 3 | 15.555 | 54904 | 0.31 |
| 4 | 17.095 | 576195 | 3.26 |

***anti*-3aa-O$^t$Bu**: The enantiomeric ratio was determined by HPLC analysis in comparison with authentic racemic material (CHIRALPAK AD-H column, 98.5/1.5 hexane/isopropyl alcohol, 0.5 mL/min, major isomer: $t_R$ = 9.0 min, minor isomer: $t_R$ = 11.9 min, UV detection at 210 nm, 30 °C).

*rac*-***anti*-3aa-O$^t$Bu** (after recrystallisation from hexane)

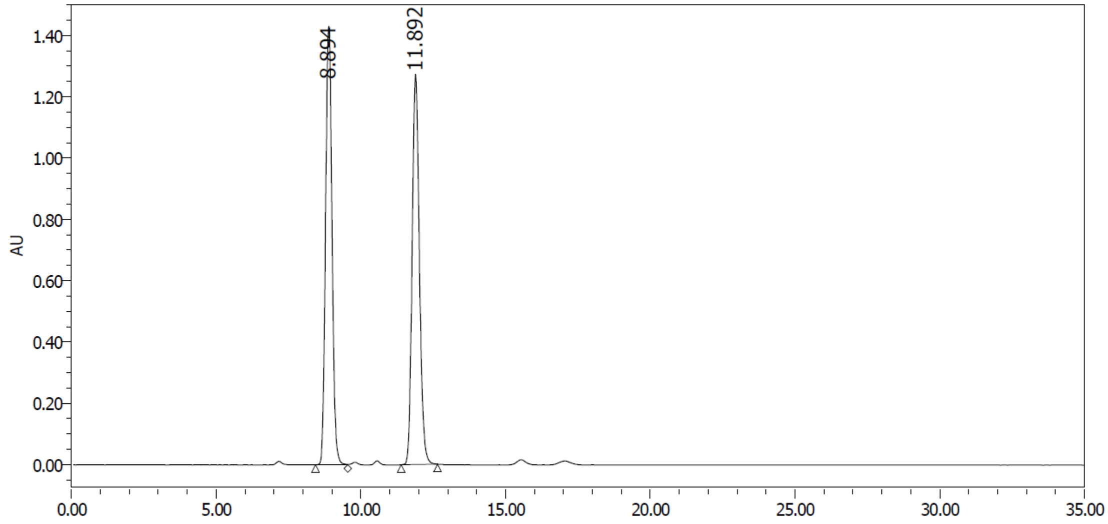


| Peak # | Ret. Time | Area | Area % |
|---|---|---|---|
| 1 | 8.894 | 22152500 | 50.02 |
| 2 | 11.892 | 22133162 | 49.98 |

*chiral*-***anti*-3aa-O$^t$Bu** (after recrystallisation from $Et_2O$/hexane)

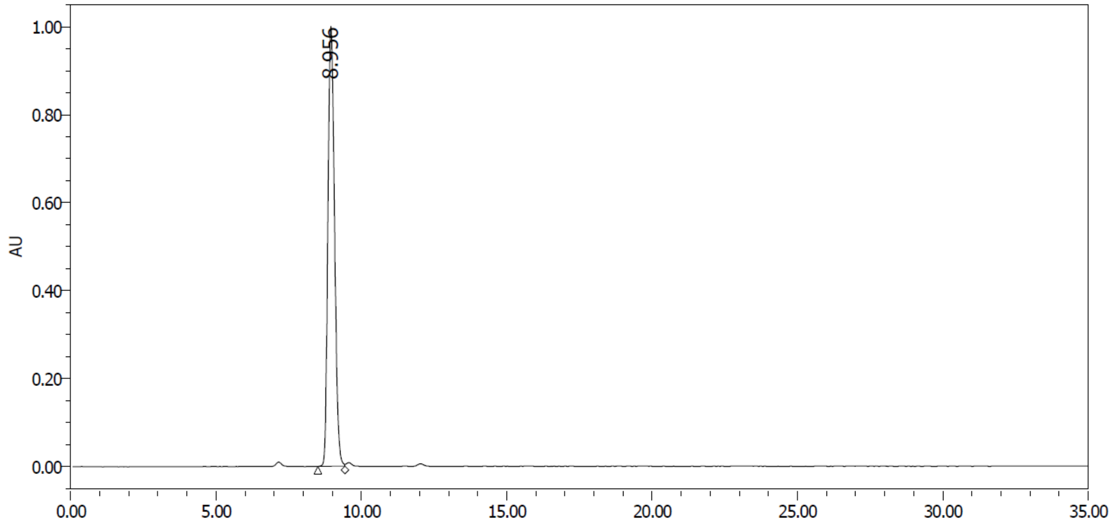


| Peak # | Ret. Time | Area | Area % |
|---|---|---|---|
| 1 | 8.956 | 15777750 | 100.00 |

**3ba-O$^t$Bu**: The enantiomeric ratio was determined by HPLC analysis in comparison with authentic racemic material (CHIRALPAK AD-H column, 99.4/0.6 hexane/isopropyl alcohol, 0.5 mL/min, major isomer: $t_R$ = 12.0 min, minor isomer: $t_R$ = 24.7 min, UV detection at 210 nm, 30 °C).

*rac*-**3ba-O$^t$Bu**

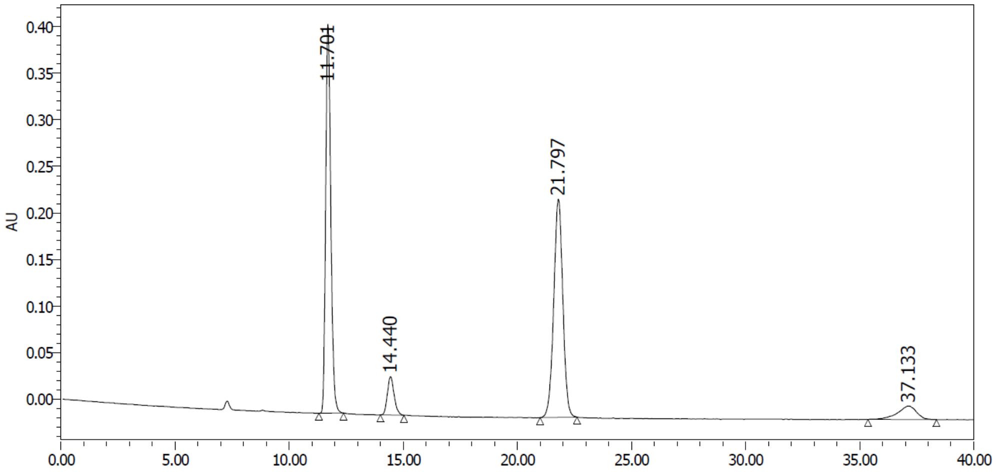


| Peak # | Ret. Time | Area | Area % |
|---|---|---|---|
| 1 | 11.701 | 6533935 | 44.40 |
| 2 | 14.440 | 846708 | 5.75 |
| 3 | 21.797 | 6490243 | 44.10 |
| 4 | 37.133 | 846092 | 5.75 |

*chiral*-**3ba-O$^t$Bu**

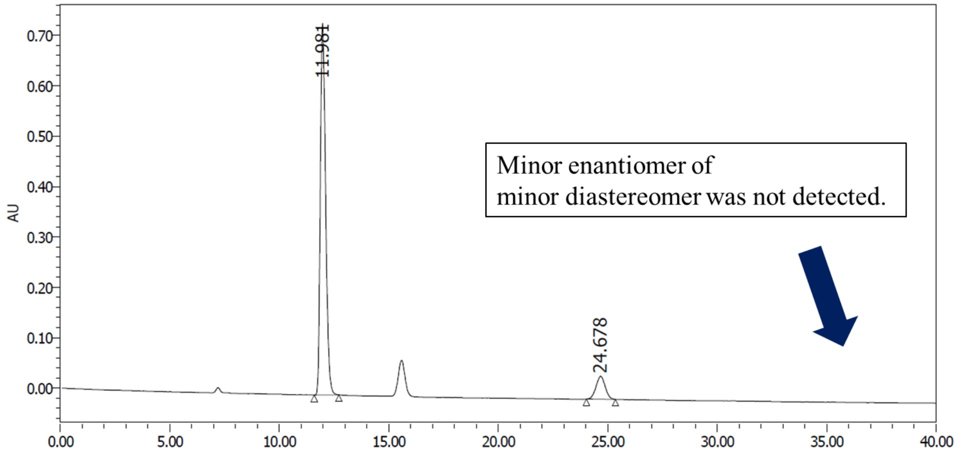


| Peak # | Ret. Time | Area | Area % |
|---|---|---|---|
| 1 | 11.981 | 12336514 | 90.34 |
| 2 | 24.678 | 1319166 | 9.66 |

*chiral*-***anti*-3ba-O$^t$Bu** (after recrystallisation from THF/hexane)

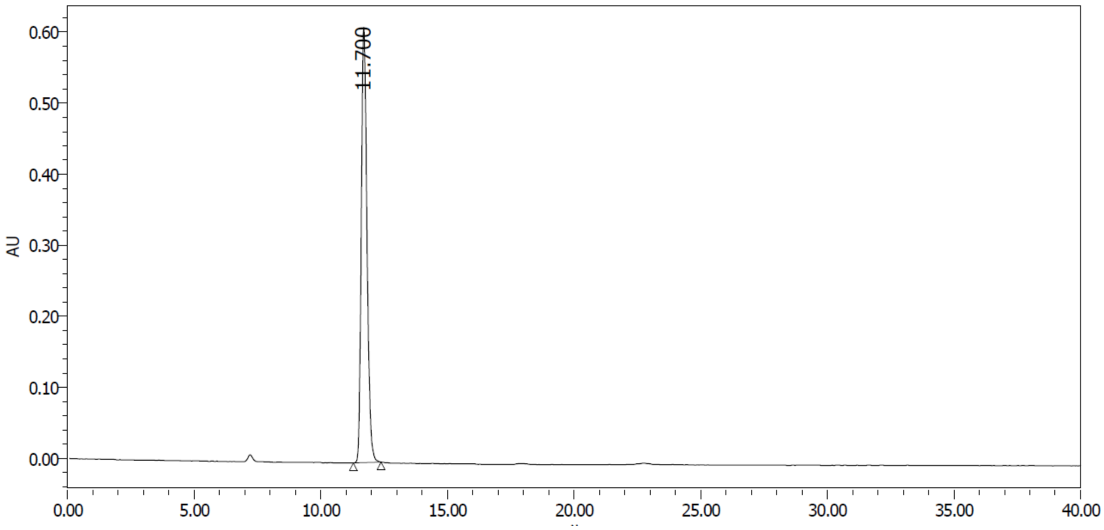


| Peak # | Ret. Time | Area | Area % |
|---|---|---|---|
| 1 | 11.700 | 9575709 | 100.00 |

**3ea-O$^t$Bu**: The enantiomeric ratio was determined by HPLC analysis in comparison with authentic racemic material (CHIRALPAK AD-H column, 99.2/0.8 hexane/isopropyl alcohol, 0.5 mL/min, major isomers: $t_R$ = 10.8, 16.1 min, minor isomers: $t_R$ = 19.8, 21.2 min, UV detection at 210 nm, 30 °C).

*rac*-**3ea-O$^t$Bu**

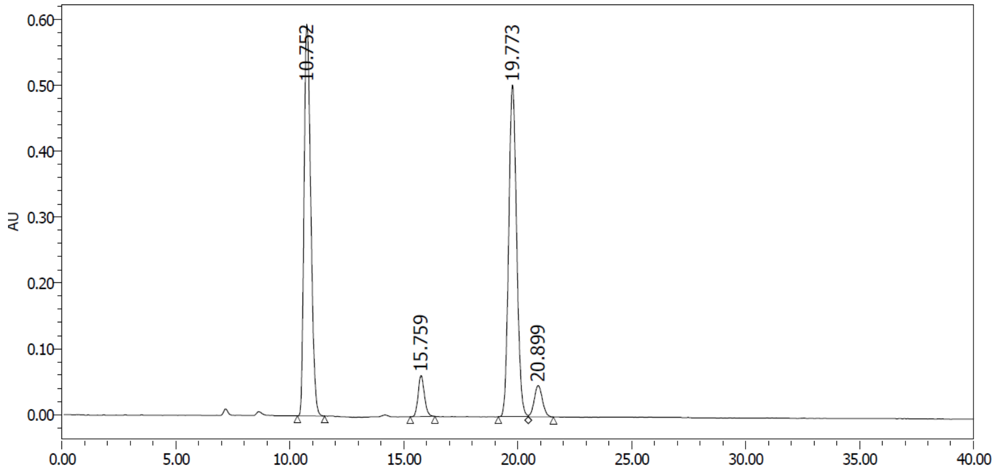


| Peak # | Ret. Time | Area | Area % |
|---|---|---|---|
| 1 | 10.752 | 12207712 | 45.53 |
| 2 | 15.759 | 1196417 | 4.46 |
| 3 | 19.773 | 12214332 | 45.56 |
| 4 | 20.899 | 1191196 | 4.44 |

*chiral*-**3ea-O$^t$Bu**

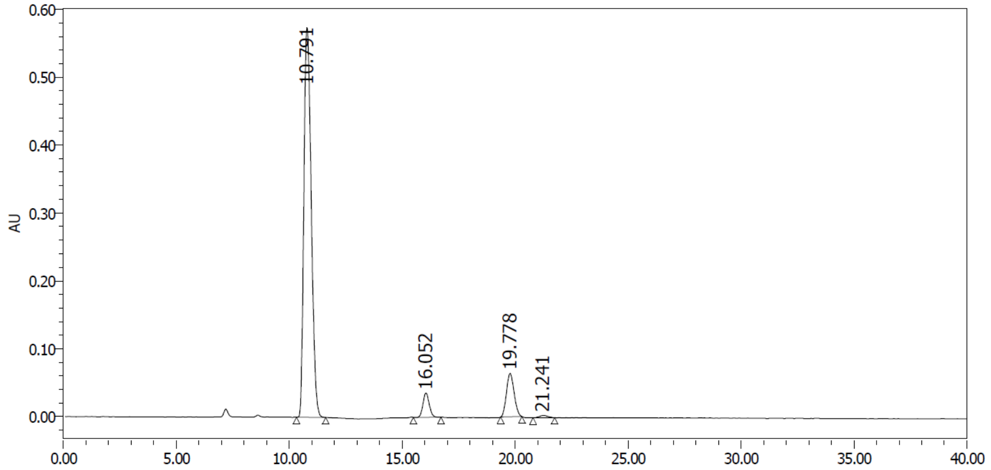


| Peak # | Ret. Time | Area | Area % |
|---|---|---|---|
| 1 | 10.791 | 12943843 | 85.15 |
| 2 | 16.052 | 686694 | 4.52 |
| 3 | 19.778 | 1493401 | 9.82 |
| 4 | 21.241 | 77824 | 0.51 |

**3fa-O$^t$Bu**: The enantiomeric ratio was determined by HPLC analysis in comparison with authentic racemic material (CHIRALPAK AD-H column, 99.2/0.8 hexane/isopropyl alcohol, 0.5 mL/min, major isomers: $t_R$ = 11.3, 16.7 min, minor isomers: $t_R$ = 20.6, 22.5 min, UV detection at 210 nm, 30 °C).

*rac*-**3fa-O$^t$Bu**

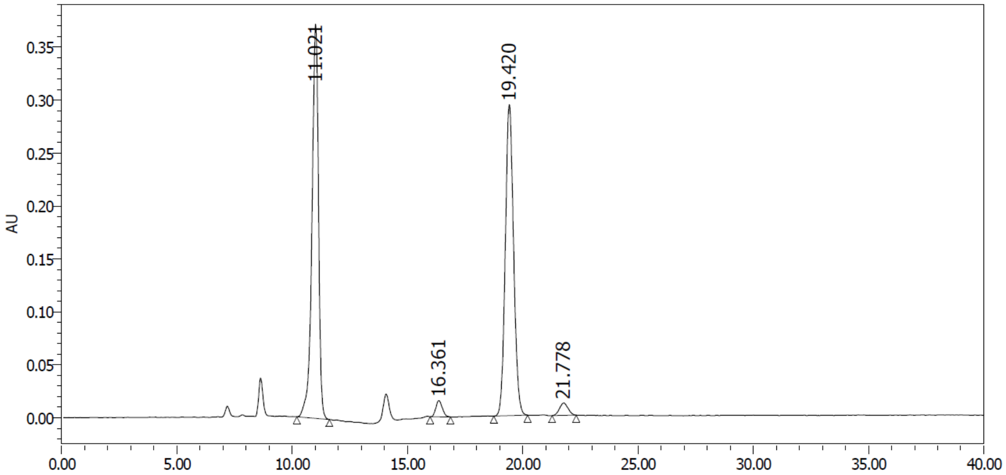


| Peak # | Ret. Time | Area | Area % |
|---|---|---|---|
| 1 | 11.021 | 7364090 | 48.13 |
| 2 | 16.361 | 300455 | 1.96 |
| 3 | 19.420 | 7336348 | 47.95 |
| 4 | 21.778 | 300051 | 1.96 |

*chiral*-**3fa-O$^t$Bu**

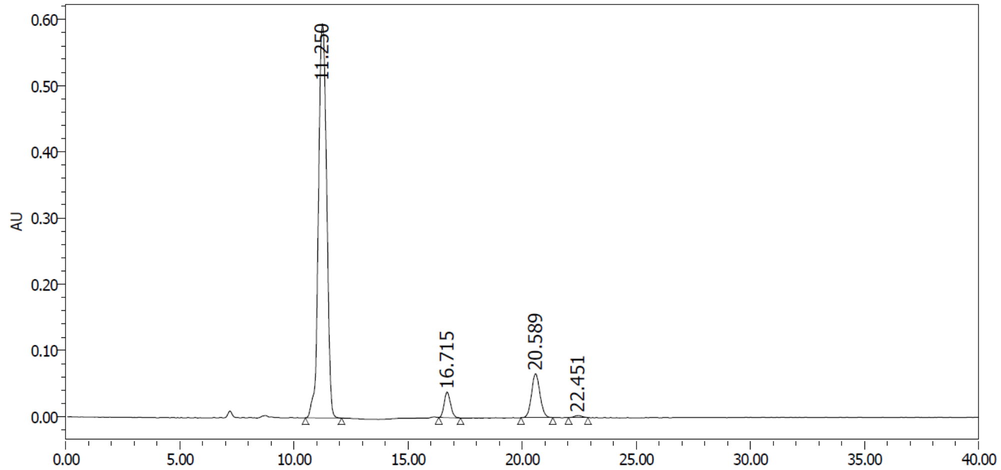


| Peak # | Ret. Time | Area | Area % |
|---|---|---|---|
| 1 | 11.250 | 14815404 | 85.39 |
| 2 | 16.715 | 745354 | 4.30 |
| 3 | 20.589 | 1709545 | 9.85 |
| 4 | 22.451 | 79899 | 0.46 |

**3ha-O$^t$Bu**: The enantiomeric ratio was determined by HPLC analysis in comparison with authentic racemic material (CHIRALCEL OD-H column, 99.9/0.1 hexane/isopropyl alcohol, 0.5 mL/min, major isomers: $t_R$ = 14.1, 17.5 min, minor isomers: $t_R$ = 11.0, 15.8 min, UV detection at 210 nm, 30 °C).

*rac*-**3ha-O$^t$Bu**

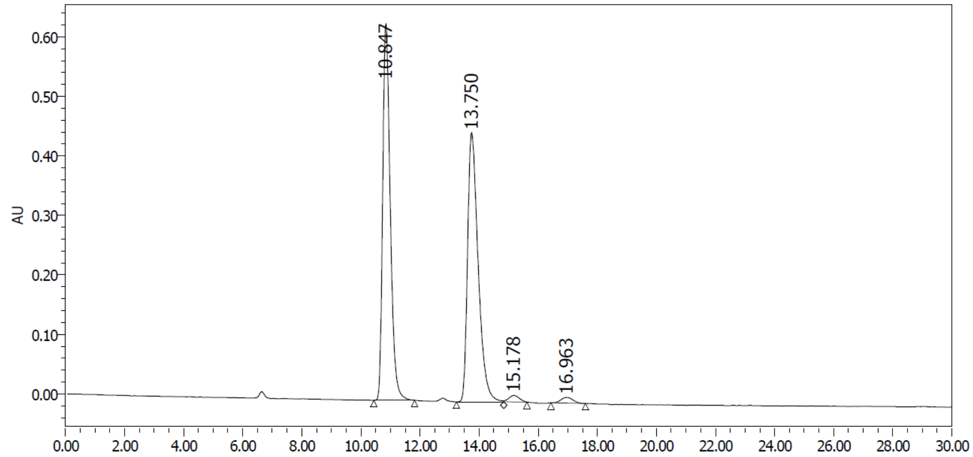


| Peak # | Ret. Time | Area | Area % |
|---|---|---|---|
| 1 | 10.847 | 11262559 | 48.83 |
| 2 | 13.750 | 11252977 | 48.79 |
| 3 | 15.178 | 272755 | 1.18 |
| 4 | 16.963 | 275140 | 1.19 |

*chiral*-**3ha-O$^t$Bu**

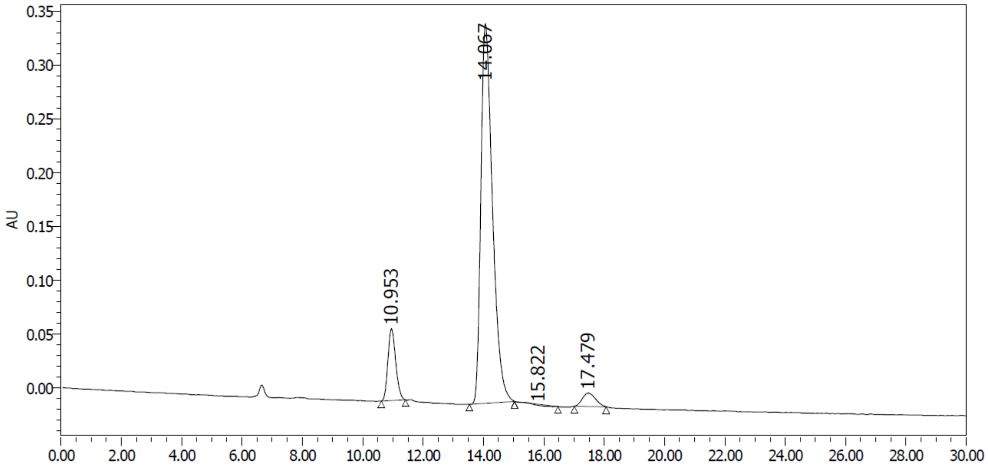


| Peak # | Ret. Time | Area | Area % |
|---|---|---|---|
| 1 | 10.953 | 1201503 | 10.88 |
| 2 | 14.067 | 9418077 | 85.27 |
| 3 | 15.822 | 48405 | 0.44 |
| 4 | 17.479 | 377207 | 3.42 |

**3ia-O$^t$Bu**: The enantiomeric ratio was determined by HPLC analysis in comparison with authentic racemic material (CHIRALPAK AD-H column, 99/1 hexane/isopropyl alcohol, 0.5 mL/min, major isomers: $t_R$ = 11.0, 18.7 min, minor isomers: $t_R$ = 21.5, 25.8 min, UV detection at 210 nm, 30 °C).

*rac*-**3ia-O$^t$Bu**

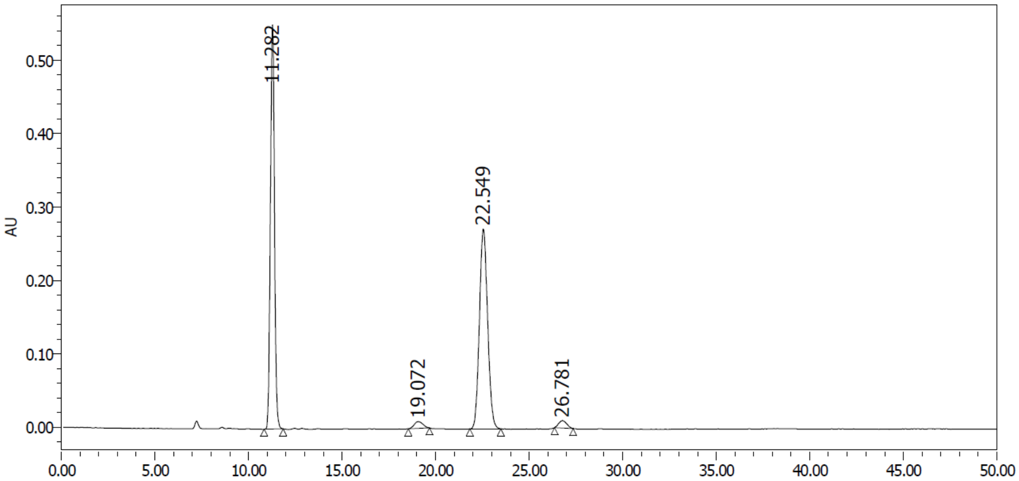


| Peak # | Ret. Time | Area | Area % |
|---|---|---|---|
| 1 | 11.282 | 8291385 | 48.18 |
| 2 | 19.072 | 300687 | 1.75 |
| 3 | 22.549 | 8312011 | 48.30 |
| 4 | 26.781 | 303500 | 1.76 |

*chiral*-**3ia-O$^t$Bu**

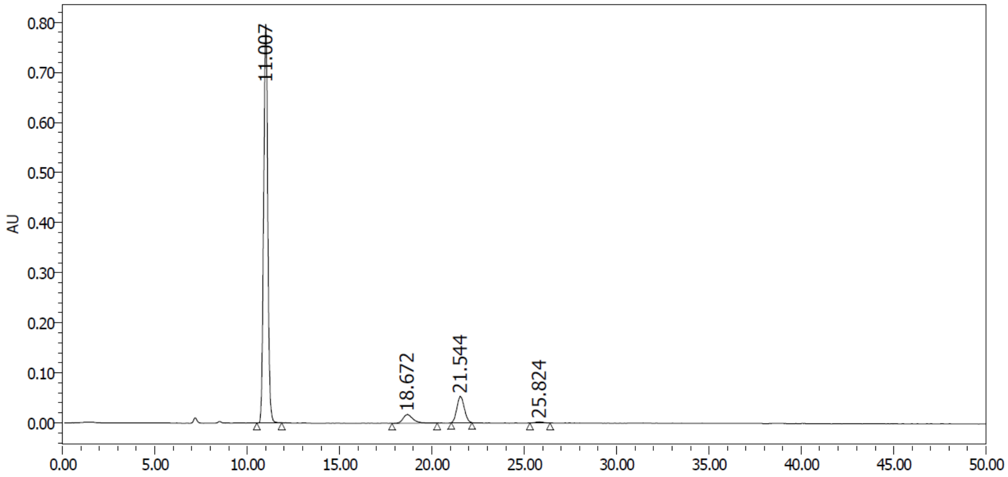


| Peak # | Ret. Time | Area | Area % |
|---|---|---|---|
| 1 | 11.007 | 12390139 | 85.10 |
| 2 | 18.672 | 648455 | 4.45 |
| 3 | 21.544 | 1452373 | 9.98 |
| 4 | 25.824 | 67731 | 0.47 |

**3ja-O$^t$Bu**: The enantiomeric ratio was determined by HPLC analysis in comparison with authentic racemic material (CHIRALPAK AD-H column, 98/2 hexane/isopropyl alcohol, 0.5 mL/min, major isomers: $t_R$ = 9.9, 13.3 min, minor isomers: $t_R$ = 19.1, 20.1 min, UV detection at 210 nm, 30 °C).

*rac*-**3ja-O$^t$Bu**

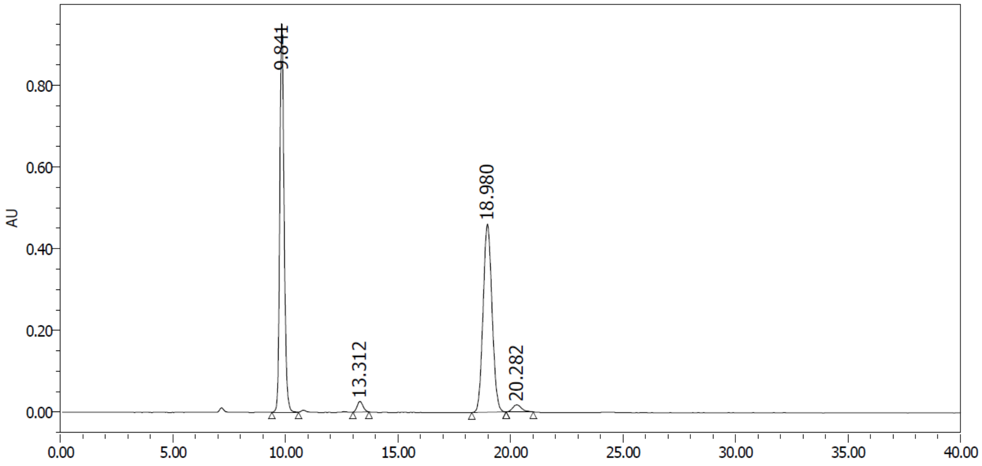


| Peak # | Ret. Time | Area | Area % |
|---|---|---|---|
| 1 | 9.841 | 12723848 | 48.21 |
| 2 | 13.312 | 473322 | 1.79 |
| 3 | 18.980 | 12722722 | 48.20 |
| 4 | 20.282 | 473838 | 1.80 |

*chiral*-**3ja-O$^t$Bu**

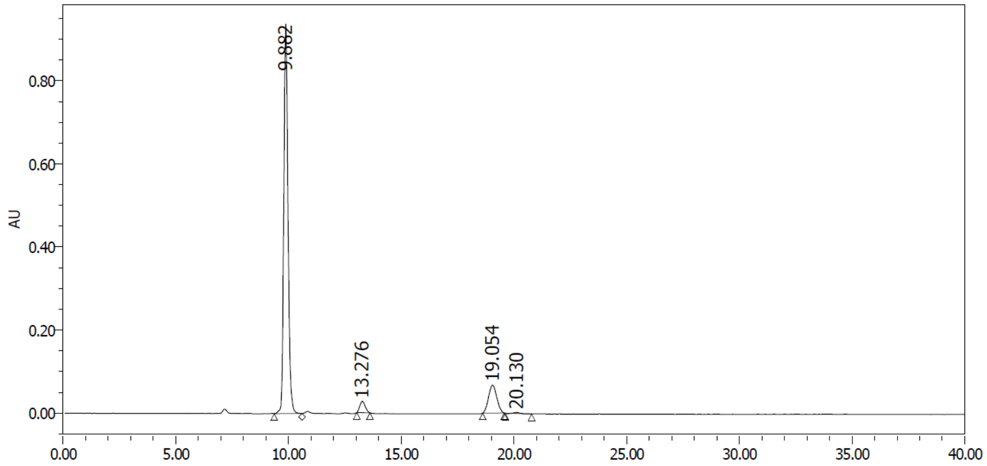


| Peak # | Ret. Time | Area | Area % |
|---|---|---|---|
| 1 | 9.882 | 12154688 | 85.45 |
| 2 | 13.276 | 459416 | 3.23 |
| 3 | 19.054 | 1555615 | 10.94 |
| 4 | 20.130 | 53837 | 0.38 |

**3ka-O$^t$Bu**: The enantiomeric ratio was determined by HPLC analysis in comparison with authentic racemic material (CHIRALPAK AD-H column, 98.8/1.2 hexane/isopropyl alcohol, 0.5 mL/min, major isomers: $t_R$ = 16.3, 25.7 min, minor isomers: $t_R$ = 37.7, 44.5 min, UV detection at 210 nm, 30 ºC).

*rac*-**3ka-O$^t$Bu**

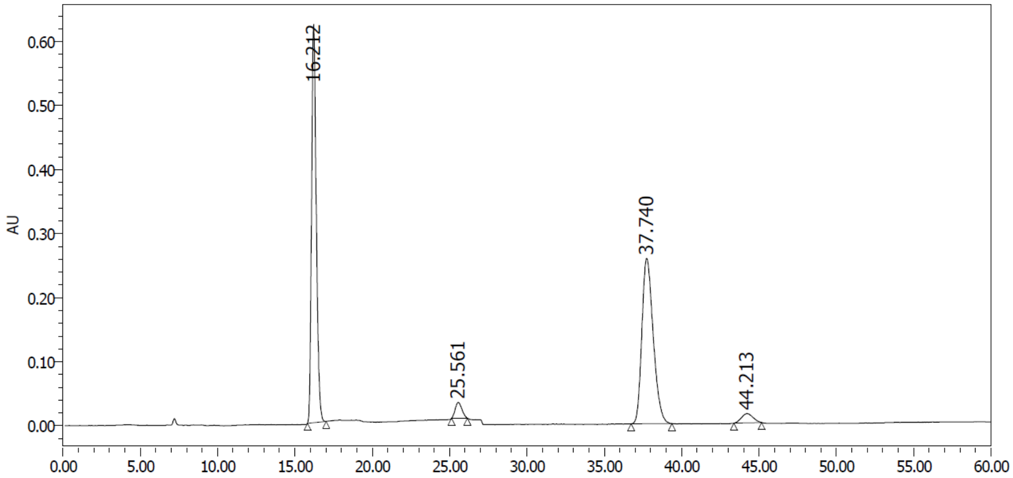


| Peak # | Ret. Time | Area | Area % |
|---|---|---|---|
| 1 | 16.212 | 13147144 | 47.32 |
| 2 | 25.561 | 736748 | 2.65 |
| 3 | 37.740 | 13152460 | 47.34 |
| 4 | 44.213 | 747173 | 2.69 |

*chiral*-**3ka-O$^t$Bu**

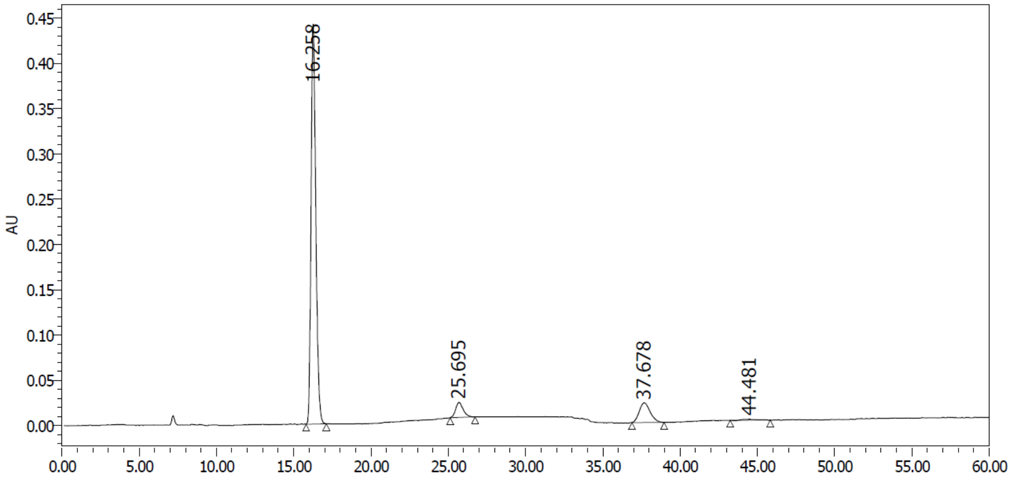


| Peak # | Ret. Time | Area | Area % |
|---|---|---|---|
| 1 | 16.258 | 9429710 | 84.71 |
| 2 | 25.695 | 572022 | 5.14 |
| 3 | 37.678 | 1063707 | 9.56 |
| 4 | 44.481 | 66610 | 0.60 |

**3ma-O$^t$Bu**: The enantiomeric ratio was determined by HPLC analysis in comparison with authentic racemic material (CHIRALPAK AD-H column, 99.4/0.6 hexane/isopropyl alcohol, 0.5 mL/min, major isomer: $t_R$ = 13.8 min, minor isomer: $t_R$ = 26.4 min, UV detection at 210 nm, 30 °C).

*rac*-**3ma-O$^t$Bu**

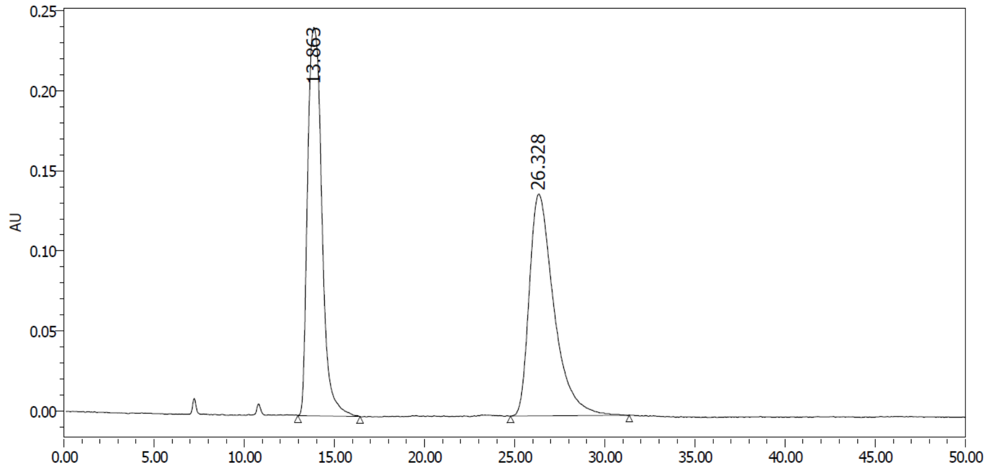


| Peak # | Ret. Time | Area | Area % |
|---|---|---|---|
| 1 | 13.863 | 13275148 | 50.29 |
| 2 | 26.328 | 13121270 | 49.71 |

*chiral*-**3ma-O$^t$Bu**

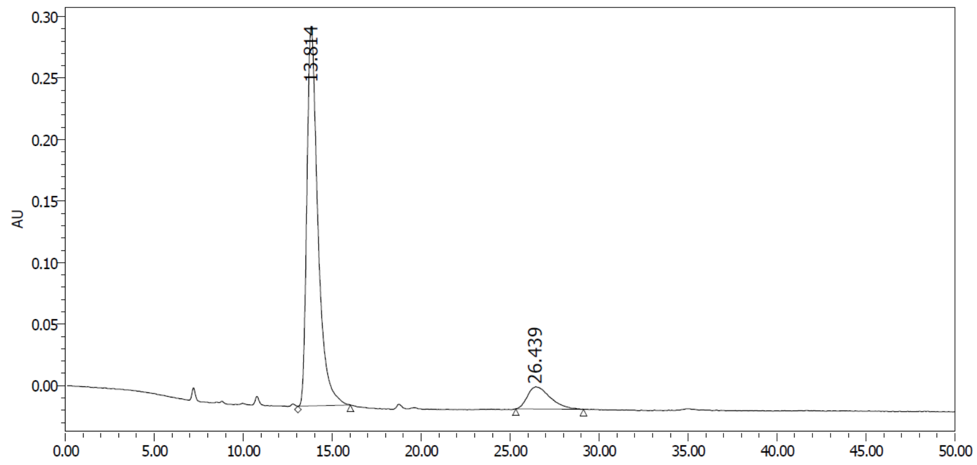


| Peak # | Ret. Time | Area | Area % |
|---|---|---|---|
| 1 | 13.814 | 13258317 | 89.22 |
| 2 | 26.439 | 1602044 | 10.78 |

**3ac-O$^t$Bu**: The enantiomeric ratio was determined by HPLC analysis in comparison with authentic racemic material (CHIRALPAK AD-H column, 99.2/0.8 hexane/isopropyl alcohol, 0.5 mL/min, major isomers: $t_R$ = 12.3, 18.1 min, minor isomers: $t_R$ = 13.5, 15.7 min, UV detection at 210 nm, 30 °C).

*rac*-**3ac-O$^t$Bu**

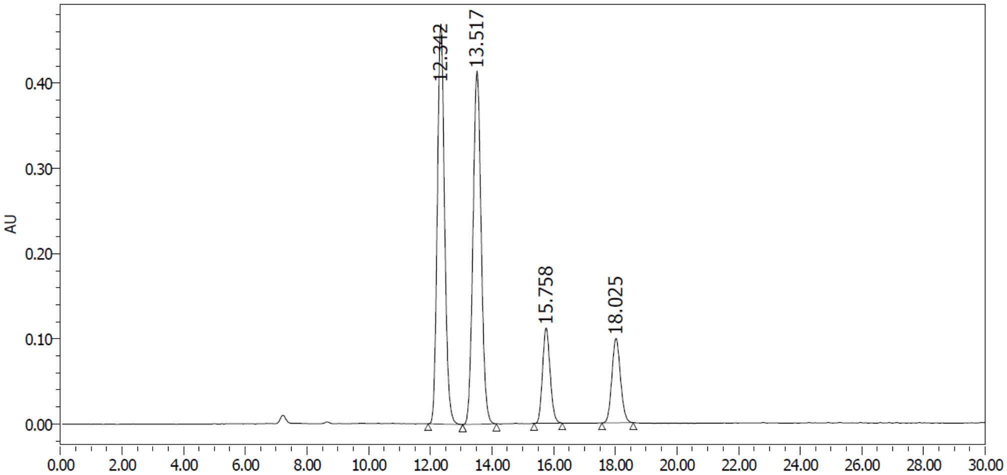


| Peak # | Ret. Time | Area | Area % |
|---|---|---|---|
| 1 | 12.342 | 7678808 | 40.07 |
| 2 | 13.517 | 7656084 | 39.95 |
| 3 | 15.758 | 1908981 | 9.96 |
| 4 | 18.025 | 1921711 | 10.03 |

*chiral*-**3ac-O$^t$Bu**

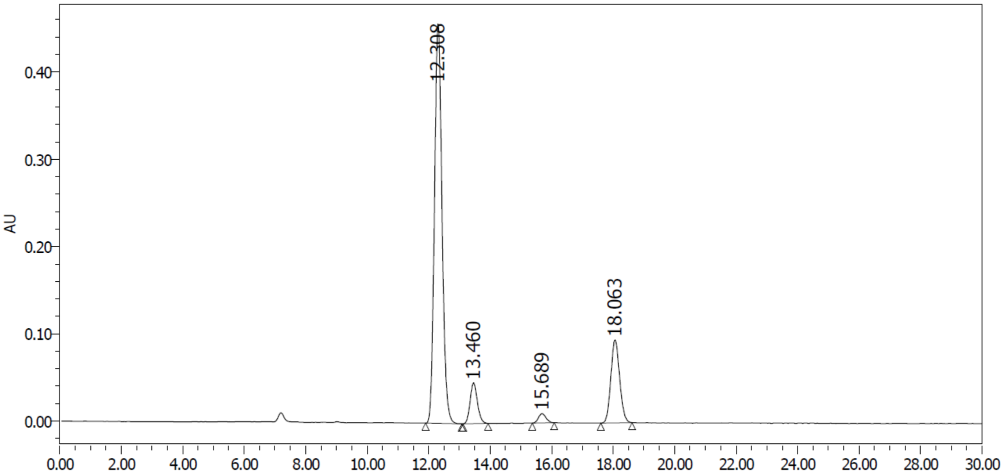


| Peak # | Ret. Time | Area | Area % |
|---|---|---|---|
| 1 | 12.308 | 7823557 | 73.32 |
| 2 | 13.460 | 771535 | 7.23 |
| 3 | 15.689 | 183569 | 1.72 |
| 4 | 18.063 | 1892128 | 17.73 |

**3af-O$^t$Bu**: The enantiomeric ratio was determined by HPLC analysis in comparison with authentic racemic material (CHIRALPAK AD-H column, 99.5/0.5 hexane/isopropyl alcohol, 0.5 mL/min, major isomers: $t_R$ = 30.7, 32.9 min, minor isomers: $t_R$ = 25.6, 28.9 min, UV detection at 210 nm, 30 °C).

*rac*-**3af-O$^t$Bu**

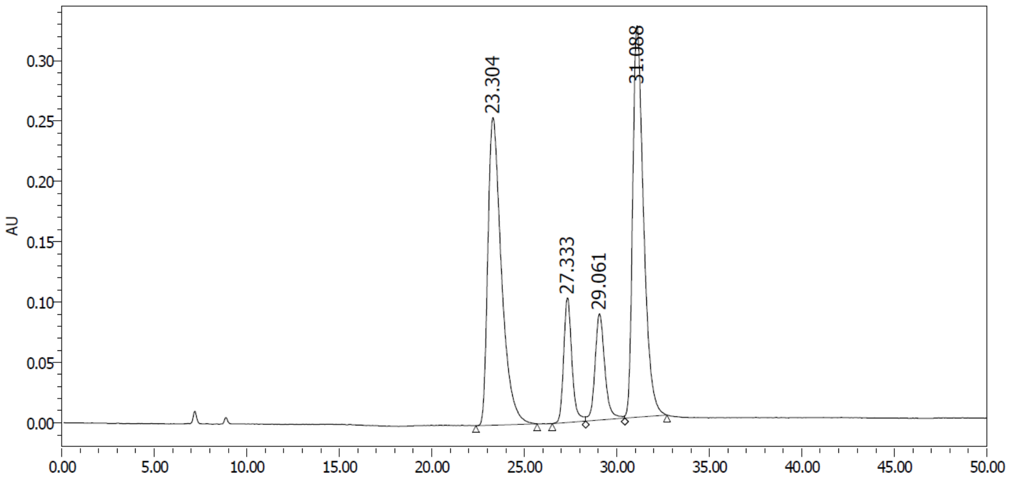


| Peak # | Ret. Time | Area | Area % |
|---|---|---|---|
| 1 | 23.304 | 13021799 | 39.81 |
| 2 | 27.333 | 3189874 | 9.75 |
| 3 | 29.061 | 3228185 | 9.87 |
| 4 | 31.088 | 13267160 | 40.56 |

*chiral*-**3af-O$^t$Bu**

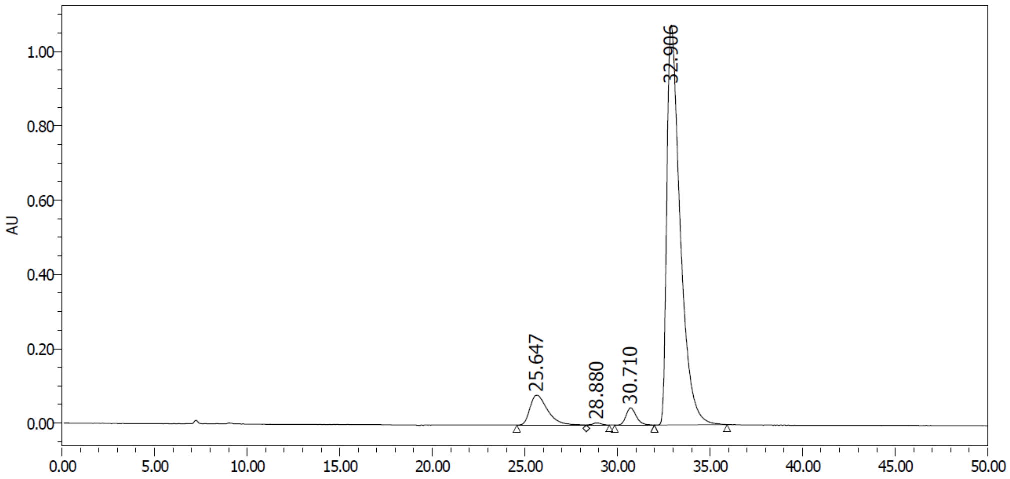


| Peak # | Ret. Time | Area | Area % |
|---|---|---|---|
| 1 | 25.647 | 5309355 | 8.69 |
| 2 | 28.880 | 180447 | 0.30 |
| 3 | 30.710 | 1791684 | 2.93 |
| 4 | 32.906 | 53799853 | 88.08 |

***anti*-5**: The enantiomeric ratio was determined by HPLC analysis in comparison with authentic racemic material (CHIRALCEL OD-H column, 97/3 hexane/isopropyl alcohol, 0.5 mL/min, major isomer: $t_R$ = 13.5 min, minor isomer: $t_R$ = 14.9 min, UV detection at 210 nm, 30 °C).

*rac*- ***anti*-5**

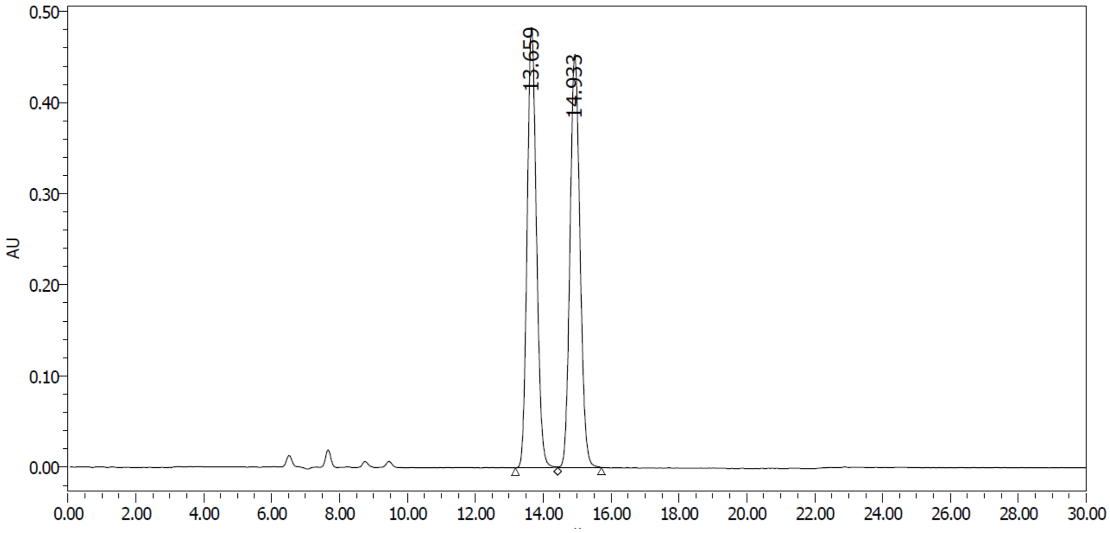


| Peak # | Ret. Time | Area | Area % |
|---|---|---|---|
| 1 | 13.659 | 9207018 | 49.98 |
| 2 | 14.933 | 9212661 | 50.02 |

*chiral*- ***anti*-5**

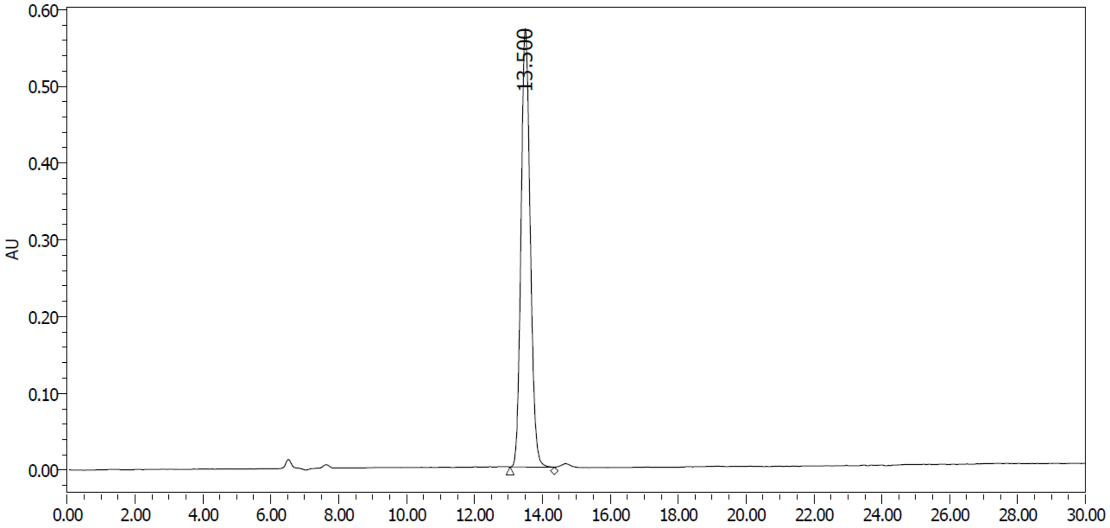


| Peak # | Ret. Time | Area | Area % |
|---|---|---|---|
| 1 | 13.500 | 10746767 | 100.00 |

***anti*-6**: The enantiomeric ratio was determined by HPLC analysis in comparison with authentic racemic material (CHIRALCEL OD-H column, 99.9/0.1 hexane/isopropyl alcohol, 0.5 mL/min, major isomer: $t_R$ = 19.7 min, minor isomer: $t_R$ = 19.0 min, UV detection at 210 nm, 30 °C).

*rac*- ***anti*-6**

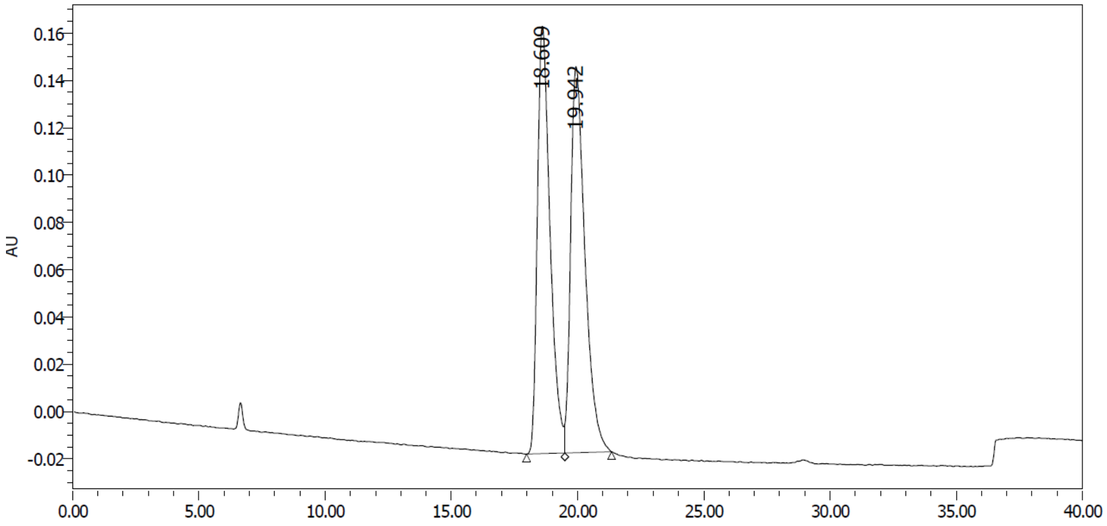


| Peak # | Ret. Time | Area | Area % |
|---|---|---|---|
| 1 | 18.609 | 6325471 | 49.62 |
| 2 | 19.942 | 6423226 | 50.38 |

*chiral*- ***anti*-6**

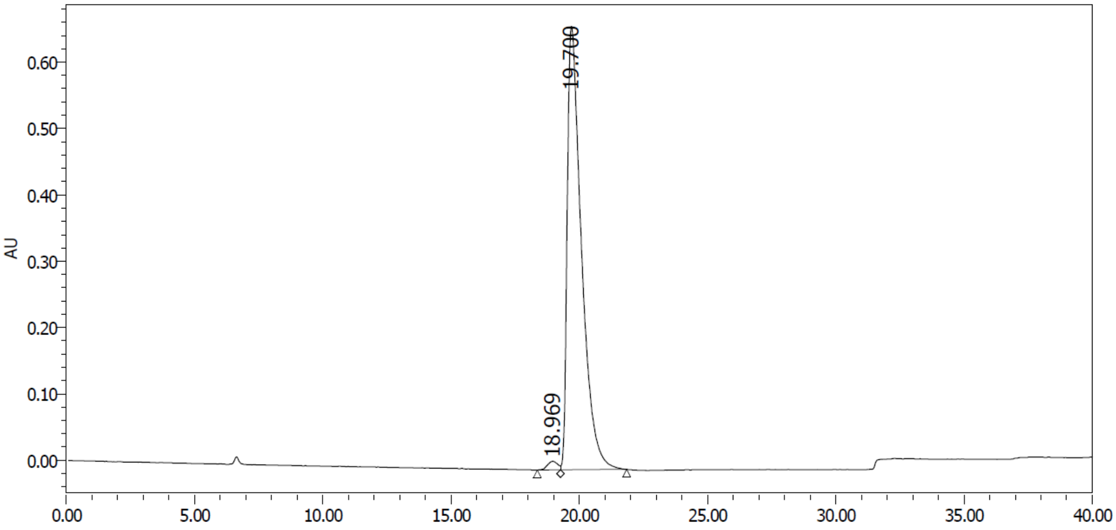


| Peak # | Ret. Time | Area | Area % |
|---|---|---|---|
| 1 | 18.969 | 356815 | 1.37 |
| 2 | 19.700 | 25691999 | 98.63 |

***anti*-7**: The enantiomeric ratio was determined by HPLC analysis in comparison with authentic racemic material (CHIRALPAK AD-H column, 99.6/0.4 hexane/isopropyl alcohol, 0.5 mL/min, major isomer: $t_R$ = 11.6 min, minor isomer: $t_R$ = 15.4 min, UV detection at 210 nm, 30 ºC).

*rac*- ***anti*-7**

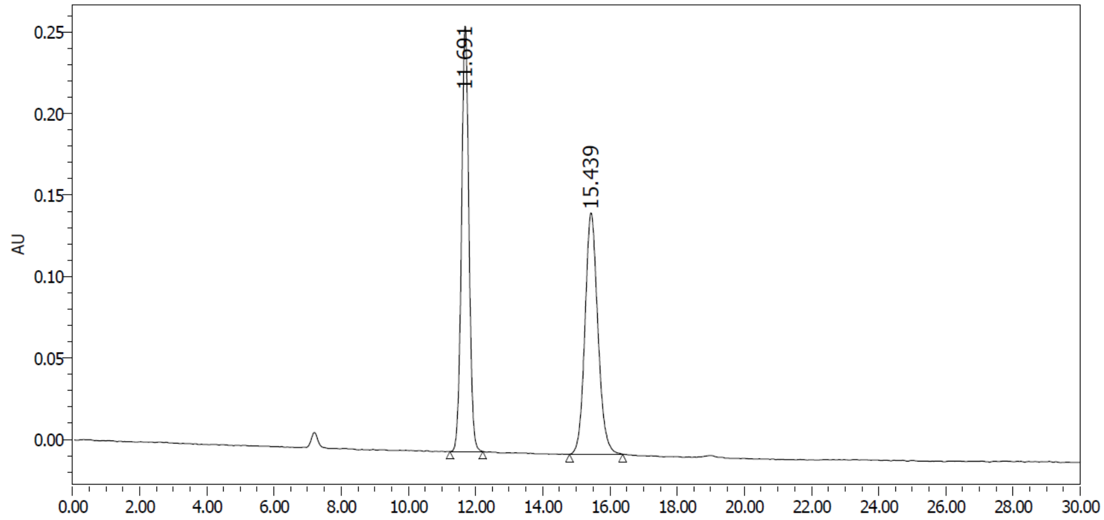


| Peak # | Ret. Time | Area | Area % |
|---|---|---|---|
| 1 | 11.691 | 3989763 | 50.29 |
| 2 | 15.439 | 3944436 | 49.71 |

*chiral*- ***anti*-7**

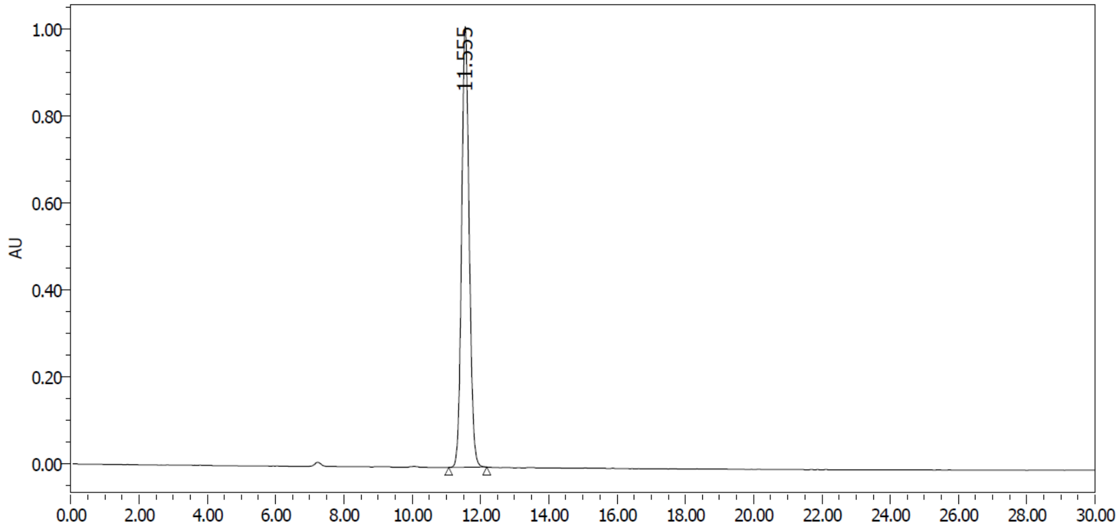


| Peak # | Ret. Time | Area | Area % |
|---|---|---|---|
| 1 | 11.555 | 15547138 | 100.00 |

***anti*-8**: The enantiomeric ratio was determined by HPLC analysis in comparison with authentic racemic material (CHIRALPAK AD-H column, 99.7/0.3 hexane/isopropyl alcohol, 0.5 mL/min, major isomer: $t_R$ = 17.6 min, minor isomer: $t_R$ = 22.2 min, UV detection at 210 nm, 30 °C).

*rac*- ***anti*-8**

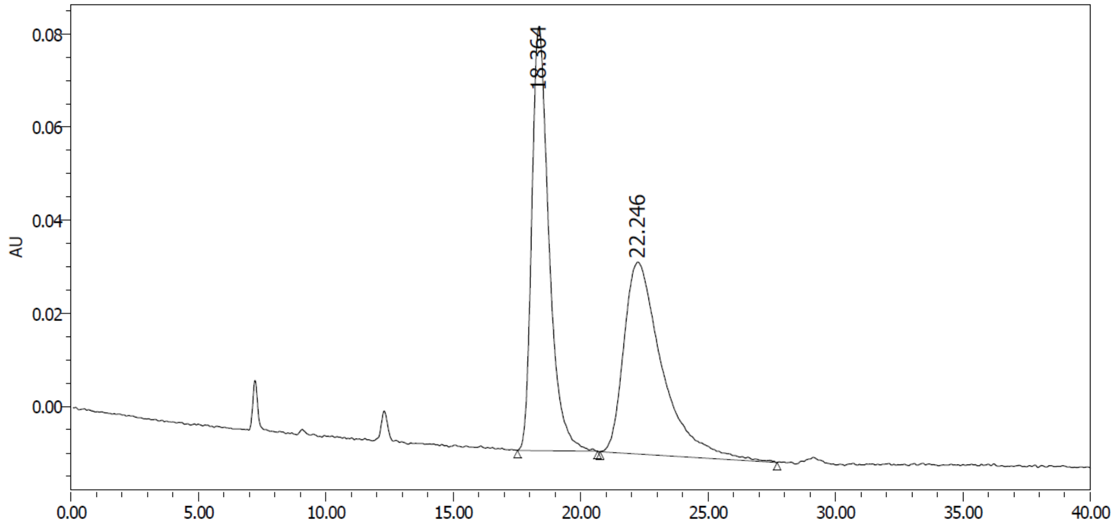


| Peak # | Ret. Time | Area | Area % |
|---|---|---|---|
| 1 | 18.364 | 4275833 | 49.95 |
| 2 | 22.246 | 4283619 | 50.05 |

*chiral*- ***anti*-8**

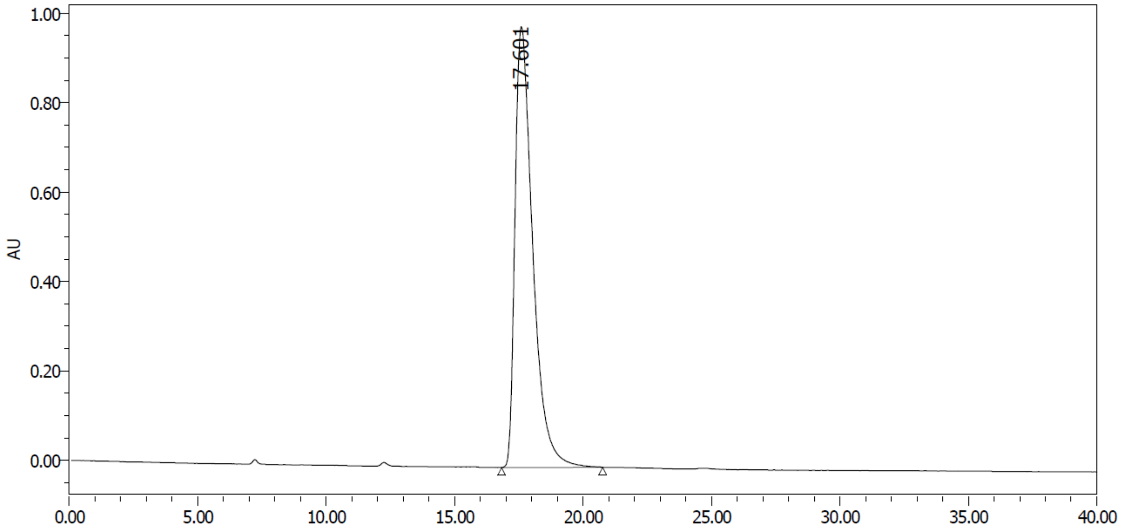


| Peak # | Ret. Time | Area | Area % |
|---|---|---|---|
| 1 | 17.601 | 47209901 | 100.00 |

***anti*-9**: The enantiomeric ratio was determined by HPLC analysis in comparison with authentic racemic material (CHIRALCEL OD-H column, 99.5/0.5 hexane/isopropyl alcohol, 0.5 mL/min, major isomer: $t_R$ = 19.1 min, minor isomer: $t_R$ = 19.8 min, UV detection at 210 nm, 30 °C).

*rac*- ***anti*-9**

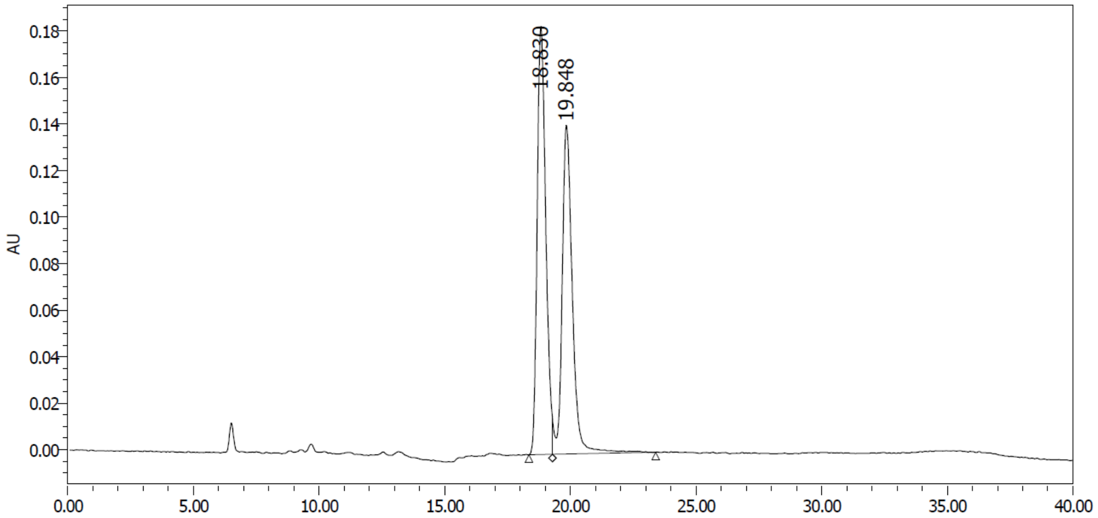


| Peak # | Ret. Time | Area | Area % |
|---|---|---|---|
| 1 | 18.830 | 4214871 | 50.32 |
| 2 | 19.848 | 4160537 | 49.68 |

*chiral*- ***anti*-9**

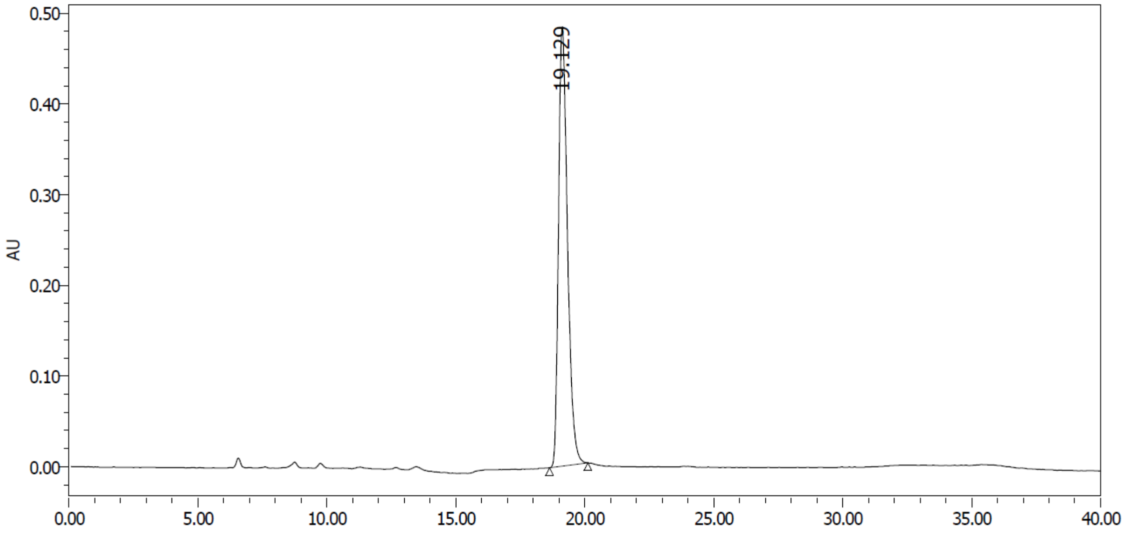


| Peak # | Ret. Time | Area | Area % |
|---|---|---|---|
| 1 | 19.129 | 11718446 | 100.00 |

**5ba**: The enantiomeric ratio was determined by HPLC analysis in comparison with authentic racemic material (CHIRALCEL OD-H column, 92.0/8.0 hexane/isopropyl alcohol, 0.5 mL/min, major isomer: $t_R$ = 13.1 min, minor isomer: $t_R$ = 14.7 min, UV detection at 210 nm, 30 °C).

*rac*-**5ba**

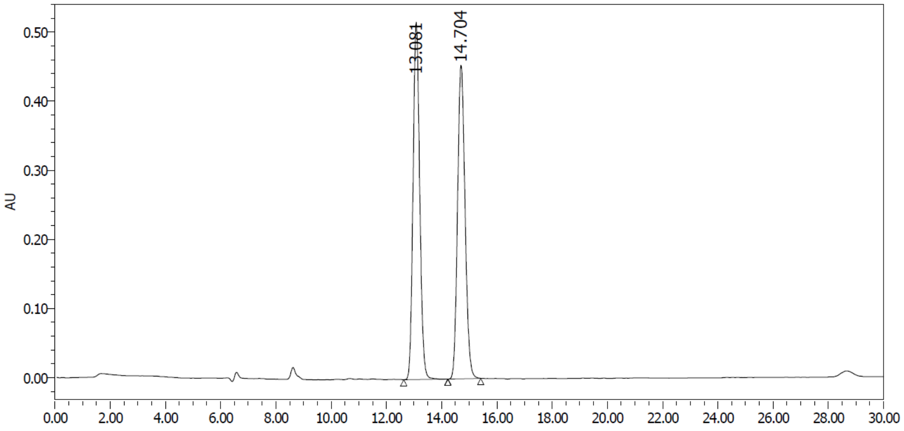


| Peak # | Ret. Time | Area | Area % |
|---|---|---|---|
| 1 | 13.081 | 8610437 | 50.13 |
| 2 | 14.704 | 8564871 | 49.87 |

*chiral*-**5ba** from **3ba**

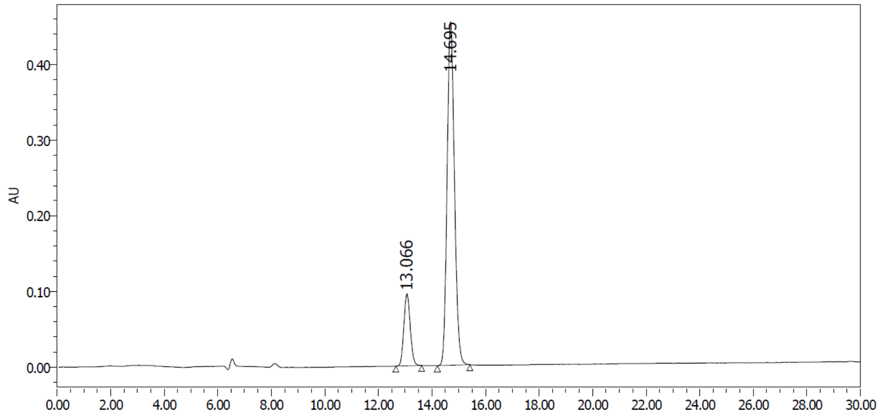


| Peak # | Ret. Time | Area | Area % |
|---|---|---|---|
| 1 | 13.066 | 1577833 | 15.47 |
| 2 | 14.695 | 8624452 | 84.53 |

*chiral*-**5ba** from L-Allothreonine

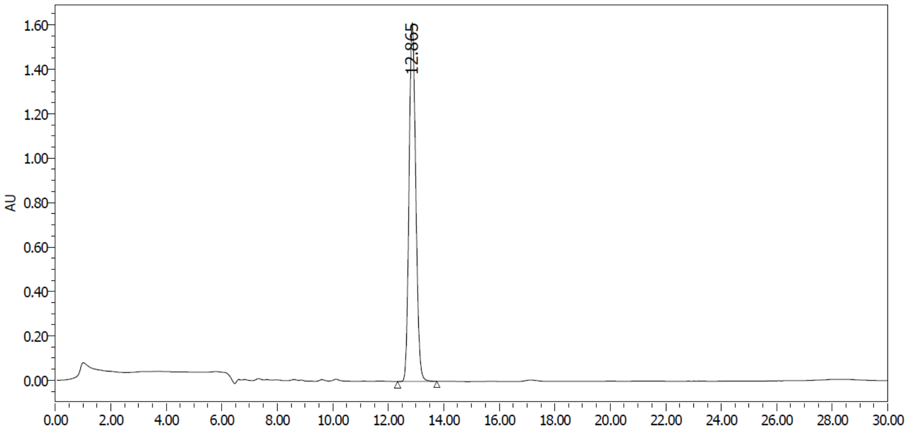


| Peak # | Ret. Time | Area | Area % |
|---|---|---|---|
| 1 | 12.865 | 27149253 | 100 |

**Characterisation Data for Products**

Copy of $^{1}H$, $^{13}C\{^{1}H\}$, $^{19}F\{^{1}H\}$, and $^{11}B$ NMR spectra for all compounds are attached in the last part.

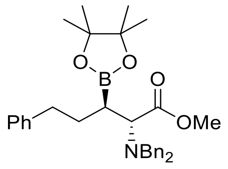

***anti*-3aa**

**Methyl (2*R**,3*R**)-2-(dibenzylamino)-5-phenyl-3-(4,4,5,5-tetramethyl-1,3,2-dioxaborolan-2-yl)pentanoate (*anti*-3aa)**

It was purified by silica gel column chromatography with hexane/ethyl acetate (5/1, v/v) and GPC ($CHCl_3$): 95.0 mg (74%, 0.25 mmol scale), 365 mg (71%, 1.0 mmol scale); white solid; mp 112.3-113.3 °C; $^{1}H$ NMR ($CDCl_3$, 400 MHz): δ 7.30-7.11 (m, 15H), 3.87 (d, $J$ = 13.6 Hz, 2H), 3.76 (s, 3H), 3.42 (d, $J$ = 11.3 Hz, 1H), 3.36 (d, $J$ = 13.6 Hz, 2H), 2.46-2.31 (m, 2H), 2.09-2.00 (m, 1H), 1.69-1.58 (m, 2H), 1.22 (s, 6H), 1.19 (s, 6H); $^{13}C\{^{1}H\}$ NMR ($CDCl_3$, 100 MHz): δ 173.4, 143.0, 139.7, 129.3, 128.5, 128.3 (2C), 127.0, 125.7, 83.3, 61.9, 54.6, 51.0, 34.4, 29.2, 25.2, 24.6, 23.7 (broad); $^{11}B$ NMR ($CDCl_3$, 128 MHz): δ 32.48; HRMS (APCI) m/z ($[M+H]^+$) calcd for $C_{32}H_{41}BNO_4$: 514.3129, found: 514.3122.

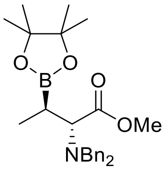

***anti*-3ba**

**Methyl (2*R**,3*R**)-2-(dibenzylamino)-3-(4,4,5,5-tetramethyl-1,3,2-dioxaborolan-2-yl)butanoate (*anti*-3ba, *anti*/*syn* = 98:2)**

It was purified by silica gel column chromatography with hexane/ethyl acetate (5/1, v/v) and GPC ($CHCl_3$): 80.4 mg (76%, 0.25 mmol scale); white solid; mp 110.5-111.5 °C; $^{1}H$ NMR ($CDCl_3$, 400 MHz): δ 7.35 (d, $J$ = 7.2 Hz, 4H), 7.29 (t, $J$ = 7.2 Hz, 4H), 7.21 (t, $J$ = 7.2 Hz, 2H), 3.90 (d, $J$ = 13.7 Hz, 2H), 3.74 (s, 3H), 3.42 (d, $J$ = 13.7 Hz, 2H), 3.27 (d, $J$ = 11.8 Hz, 1H), 1.63-1.55 (m, 1H), 1.18 (s, 6H), 1.15 (s, 6H), 1.00 (d, $J$ = 7.4 Hz, 3H); $^{13}C\{^{1}H\}$ NMR ($CDCl_3$, 100 MHz): δ 173.6, 139.8, 129.2, 128.2, 126.9, 83.1, 63.8, 54.5, 51.0, 24.9, 24.4, 17.5 (broad), 12.6; $^{11}B$ NMR ($CDCl_3$, 128 MHz): δ 32.78; HRMS (APCI) m/z ($[M+H]^+$) calcd for $C_{25}H_{35}BNO_4$: 424.2658, found: 424.2641.

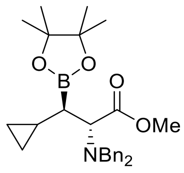

***anti*-3ca**

**Methyl (2*R**,3*R**)-3-cyclopropyl-2-(dibenzylamino)-3-(4,4,5,5-tetramethyl-1,3,2-dioxaborolan-2-yl)propanoate (*anti*-3ca)**

It was purified by silica gel column chromatography with hexane/ethyl acetate (5/1, v/v) and GPC ($CHCl_3$): 92.1 mg (82%, 0.25 mmol scale); white solid; mp 81.7-82.7 °C; $^1$H NMR ($CDCl_3$, 400 MHz): δ 7.41 (d, *J* = 7.2 Hz, 4H), 7.29 (t, *J* = 7.1 Hz, 4H), 7.21 (t, *J* = 7.2 Hz, 2H), 3.94 (d, *J* = 13.9 Hz, 2H), 3.74 (s, 3H), 3.57 (d, *J* = 11.0 Hz, 1H), 3.45 (d, *J* = 13.9 Hz, 2H), 1.48 (dd, *J* = 11.0, 7.4 Hz, 1H), 1.18 (s, 6H), 1.15 (s, 6H), 0.91-0.82 (m, 1H), 0.59-0.52 (m, 1H), 0.31-0.24 (m, 1H), 0.21-0.14 (m, 1H), 0.05--0.01 (m, 1H); $^{13}$C{$^1$H} NMR ($CDCl_3$, 100 MHz): δ 173.7, 139.9, 129.1, 128.2, 126.9, 83.3, 63.7, 54.6, 51.0, 27.7 (broad), 25.0, 24.6, 10.3, 6.3, 2.8; $^{11}$B NMR ($CDCl_3$, 128 MHz): δ 31.59; HRMS (APCI) m/z ([M+H]$^+$) calcd for $C_{27}H_{37}BNO_4$: 450.2815, found: 450.2801.

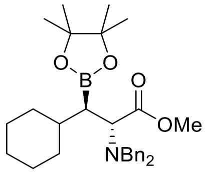

***anti*-3da**

**Methyl (2*R**,3*R**)-3-cyclohexyl-2-(dibenzylamino)-3-(4,4,5,5-tetramethyl-1,3,2-dioxaborolan-2-yl)propanoate (*anti*-3da)**

It was purified by silica gel column chromatography with hexane/ethyl acetate (5/1, v/v) and GPC ($CHCl_3$): 102.0 mg (83%, 0.25 mmol scale); white solid; mp 137.1-138.1 °C; $^1$H NMR ($CDCl_3$, 400 MHz): δ 7.34-7.27 (m, 8H), 7.21 (t, *J* = 6.7 Hz, 2H), 3.89 (d, *J* = 13.4 Hz, 2H), 3.75 (s, 3H), 3.45 (d, *J* = 12.4 Hz, 1H), 3.30 (d, *J* = 13.4 Hz, 2H), 2.00-1.94 (m, 1H), 1.74-1.56 (m, 3H), 1.46-1.45 (m, 1H), 1.30-1.26 (m, 3H), 1.19 (s, 6H), 1.15 (s, 6H), 1.03-0.98 (m, 2H), 0.87-0.84 (m, 1H), 0.59-0.49 (m, 1H); $^{13}$C{$^1$H} NMR ($CDCl_3$, 100 MHz): δ 173.4, 139.9, 129.4, 128.3, 127.0, 83.2, 59.6, 54.5, 50.9, 35.1, 34.3, 30.4 (broad), 30.3, 27.7, 27.0, 26.9, 25.3, 24.6; $^{11}$B NMR ($CDCl_3$, 128 MHz): δ 32.30; HRMS (APCI) m/z ([M+H]$^+$) calcd for $C_{30}H_{43}BNO_4$: 492.3285, found: 492.3296.

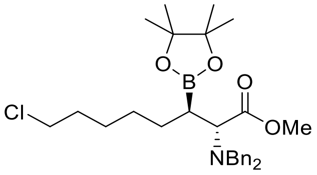

*anti*-3ea

**Methyl (2*R**,3*R**)-8-chloro-2-(dibenzylamino)-3-(4,4,5,5-tetramethyl-1,3,2-dioxaborolan-2-yl)octanoate (*anti*-3ea)**

It was purified by silica gel column chromatography with hexane/ethyl acetate (5/1, v/v) and GPC ($CHCl_3$): 95.1 mg (74%, 0.25 mmol scale); colorless oil; $^1$H NMR ($CDCl_3$, 400 MHz): δ 7.34-7.28 (m, 8H), 7.22 (t, *J* = 6.8 Hz, 2H), 3.89 (d, *J* = 13.6 Hz, 2H), 3.76 (s, 3H), 3.48 (t, *J* = 6.8 Hz, 2H), 3.36 (d, *J* = 13.6 Hz, 2H), 3.35 (d, *J* = 11.8 Hz, 1H), 1.73-1.59 (m, 3H), 1.40-1.24 (m, 4H), 1.19 (s, 6H), 1.15 (s, 6H), 1.06-0.91 (m, 2H); $^{13}$C{$^1$H} NMR ($CDCl_3$, 100 MHz): δ 173.4, 139.8, 129.3, 128.3, 127.0, 83.2, 61.6, 54.5, 51.0, 45.2, 32.7, 27.5, 27.3, 27.1, 25.1, 24.6, 23.7 (broad); $^{11}$B NMR ($CDCl_3$, 128 MHz): δ 31.94; HRMS (APCI) m/z ([M+H]$^+$) calcd for $C_{29}H_{42}BClNO_4$: 514.2895, found: 514.2886.

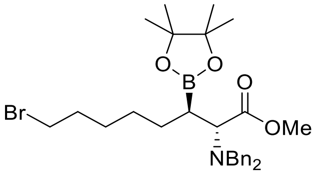

*anti*-3fa

**Methyl (2*R**,3*R**)-8-bromo-2-(dibenzylamino)-3-(4,4,5,5-tetramethyl-1,3,2-dioxaborolan-2-yl)octanoate (*anti*-3fa)**

It was purified by silica gel column chromatography with hexane/ethyl acetate (5/1, v/v) and GPC ($CHCl_3$): 101.9 mg (73%, 0.25 mmol scale); colorless oil; $^1$H NMR ($CDCl_3$, 400 MHz): δ 7.34-7.28 (m, 8H), 7.23 (t, *J* = 6.7 Hz, 2H), 3.89 (d, *J* = 13.6 Hz, 2H), 3.76 (s, 3H), 3.37 (d, *J* = 13.6 Hz, 2H), 3.36 (t, *J* = 6.9 Hz, 2H), 3.35 (d, *J* = 11.8 Hz, 1H), 1.79-1.65 (m, 3H), 1.40-1.24 (m, 4H), 1.19 (s, 6H), 1.15 (s, 6H), 1.08-0.92 (m, 2H); $^{13}$C{$^1$H} NMR ($CDCl_3$, 100 MHz): δ 173.4, 139.8, 129.3, 128.3, 127.0, 83.2, 61.6, 54.5, 51.0, 34.0, 32.8, 28.8, 27.15, 27.06, 25.1, 24.6, 23.7 (broad); $^{11}$B NMR ($CDCl_3$, 128 MHz): δ 31.92; HRMS (APCI) m/z ([M+H]$^+$) calcd for $C_{29}H_{42}BBrNO_4$: 558.2390, found: 558.2383.

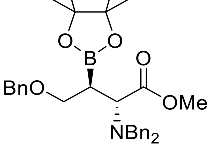

*anti*-3ga

**Methyl (2*R**,3*S**)-4-(benzyloxy)-2-(dibenzylamino)-3-(4,4,5,5-tetramethyl-1,3,2-dioxaborolan-2-yl)butanoate (*anti*-3ga)**

It was purified by silica gel column chromatography with hexane/ethyl acetate (5/1, v/v) and GPC ($CHCl_3$): 80.7 mg (61%, 0.25 mmol scale); white solid; mp 98.1-99.1 °C; $^1$H NMR ($CDCl_3$, 400 MHz): δ 7.30-7.14 (m, 15H), 4.31 (d, $J$ = 12.1 Hz, 1H), 4.26 (d, $J$ = 12.1 Hz, 1H), 3.87 (d, $J$ = 13.7 Hz, 2H), 3.76 (s, 3H), 3.71 (dd, $J$ = 8.4, 4.8 Hz, 1H), 3.65 (d, $J$ = 11.6 Hz, 1H), 3.64 (dd, $J$ = 8.4, 5.4 Hz, 1H), 3.39 (d, $J$ = 13.7 Hz, 2H), 1.90 (dt, $J$ = 11.6, 5.1 Hz, 1H), 1.16 (s, 6H), 1.12 (s, 6H); $^{13}$C{$^1$H} NMR ($CDCl_3$, 100 MHz): δ 173.0, 139.7, 138.9, 129.2, 128.3, 128.1, 127.5, 127.2, 127.0, 83.4, 72.7, 69.1, 60.0, 54.6, 51.1, 26.0 (broad), 25.0, 24.4; $^{11}$B NMR ($CDCl_3$, 128 MHz): δ 32.16; HRMS (APCI) m/z ($[M+H]^+$) calcd for $C_{32}H_{41}BNO_5$: 530.3078, found: 530.3094.

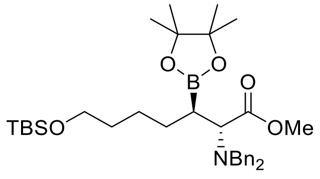


***anti*-3ha**

**Methyl (2*R**,3*R**)-7-((*tert*-butyldimethylsilyl)oxy)-2-(dibenzylamino)-3-(4,4,5,5-tetramethyl-1,3,2-dioxaborolan-2-yl)heptanoate (*anti*-3ha)**

It was purified by silica gel column chromatography with hexane/ethyl acetate (5/1, v/v) and GPC ($CHCl_3$): 116.2 mg (78%, 0.25 mmol scale); colorless oil; $^1$H NMR ($CDCl_3$, 400 MHz): δ 7.34-7.27 (m, 8H), 7.22 (t, $J$ = 7.0 Hz, 2H), 3.89 (d, $J$ = 13.6 Hz, 2H), 3.76 (s, 3H), 3.54-3.47 (m, 2H), 3.360 (d, $J$ = 13.6 Hz, 2H), 3.357 (d, $J$ = 11.5 Hz, 1H), 1.77-1.68 (m, 1H), 1.57-1.23 (m, 4H), 1.19 (s, 6H), 1.15 (s, 6H), 1.10-0.97 (m, 2H), 0.89 (s, 9H), 0.04 (s, 6H); $^{13}$C{$^1$H} NMR ($CDCl_3$, 100 MHz): δ 173.4, 139.8, 129.3, 128.2, 127.0, 83.2, 63.4, 61.8, 54.5, 50.9, 33.7, 27.3, 26.1, 25.1, 24.6, 24.4, 23.8 (broad), 18.5, -5.1; $^{11}$B NMR ($CDCl_3$, 128 MHz): δ 31.90; HRMS (APCI) m/z ($[M+H]^+$) calcd for $C_{34}H_{55}BNO_5Si$: 596.3943, found: 596.3931.

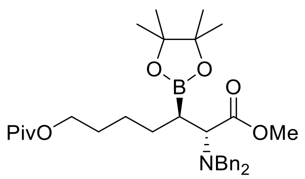


***anti*-3ia**

**Methyl (2*R**,3*R**)-2-(dibenzylamino)-7-(pivaloyloxy)-3-(4,4,5,5-tetramethyl-1,3,2-dioxaborolan-2-yl)heptanoate (*anti*-3ia)**

It was purified by silica gel column chromatography with hexane/ethyl acetate (5/1, v/v) and GPC ($CHCl_3$): 101.8 mg (72%, 0.25 mmol scale); white solid; mp 71.4-72.4 °C; $^1H$ NMR ($CDCl_3$, 400 MHz): δ 7.34-7.28 (m, 8H), 7.22 (t, $J$ = 6.7 Hz, 2H), 3.99-3.87 (m, 2H), 3.89 (d, $J$ = 13.6 Hz, 2H), 3.76 (s, 3H), 3.36 (d, $J$ = 13.6 Hz, 2H), 3.35 (d, $J$ = 11.8 Hz, 1H), 1.77-1.68 (m, 1H), 1.57-1.24 (m, 4H), 1.183 (s, 6H), 1.179 (s, 9H), 1.15 (s, 6H), 1.10-0.98 (m, 2H); $^{13}C\{^1H\}$ NMR ($CDCl_3$, 100 MHz): δ 178.6, 173.3, 139.7, 129.3, 128.2, 127.0, 83.2, 64.6, 61.6, 54.5, 50.9, 38.7, 29.3, 27.3, 27.0, 25.0, 24.52, 24.46, 23.6 (broad); $^{11}B$ NMR ($CDCl_3$, 128 MHz): δ 31.95; HRMS (APCI) m/z ($[M+H]^+$) calcd for $C_{33}H_{49}BNO_6$: 566.3653, found: 566.3676.

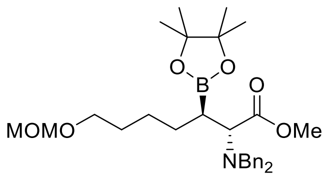


***anti*-3ja**

**Methyl (2*R**,3*R**)-2-(dibenzylamino)-7-(methoxymethoxy)-3-(4,4,5,5-tetramethyl-1,3,2-dioxaborolan-2-yl)heptanoate (*anti*-3ja)**

It was purified by silica gel column chromatography with hexane/ethyl acetate (5/1, v/v) and GPC ($CHCl_3$): 97.2 mg (74%, 0.25 mmol scale); colorless oil; $^1H$ NMR ($CDCl_3$, 400 MHz): δ 7.34-7.27 (m, 8H), 7.22 (t, $J$ = 7.0 Hz, 2H), 4.59 (s, 2H), 3.89 (d, $J$ = 13.6 Hz, 2H), 3.75 (s, 3H), 3.41 (t, $J$ = 6.9 Hz, 2H), 3.364 (d, $J$ = 13.6 Hz, 2H), 3.356 (d, $J$ = 11.7 Hz, 1H), 3.348 (s, 3H), 1.78-1.70 (m, 1H), 1.59-1.28 (m, 4H), 1.18 (s, 6H), 1.15 (s, 6H), 1.12-0.99 (m, 2H); $^{13}C\{^1H\}$ NMR ($CDCl_3$, 100 MHz): δ 173.4, 139.8, 129.3, 128.2, 127.0, 96.4, 83.2, 67.8, 61.7, 55.1, 54.5, 50.9, 30.3, 27.1, 25.0, 24.6, 24.5, 23.7 (broad); $^{11}B$ NMR ($CDCl_3$, 128 MHz): δ 32.74; HRMS (APCI) m/z ($[M+H]^+$) calcd for $C_{30}H_{45}BNO_6$: 526.3340, found: 526.3310.

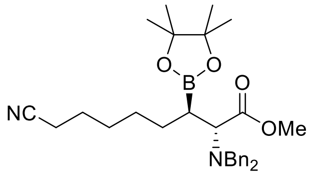


***anti*-3ka**

**Methyl (2*R**,3*R**)-8-cyano-2-(dibenzylamino)-3-(4,4,5,5-tetramethyl-1,3,2-dioxaborolan-2-yl)octanoate (*anti*-3ka)**

It was purified by silica gel column chromatography with hexane/ethyl acetate (5/1, v/v) and GPC ($CHCl_3$): 70.6 mg (56%, 0.25 mmol scale); colorless oil; $^1H$ NMR ($CDCl_3$, 400 MHz): δ 7.34-7.28 (m,

8H), 7.25-7.21 (m, 2H), 3.88 (d, *J* = 13.6 Hz, 2H), 3.76 (s, 3H), 3.37 (d, *J* = 13.6 Hz, 2H), 3.35 (d, *J* = 11.8 Hz, 1H), 2.26 (t, *J* = 7.2 Hz, 2H), 1.70-1.63 (m, 1H), 1.58-1.49 (m, 3H), 1.40-1.27 (m, 3H), 1.19 (s, 6H), 1.15 (s, 6H), 1.04-0.89 (m, 2H); $^{13}C\{^1H\}$ NMR ($CDCl_3$, 100 MHz): δ 173.4, 139.7, 129.3, 128.3, 127.0, 119.9, 83.2, 61.5, 54.5, 51.0, 29.2, 27.1, 26.8, 25.4, 25.1, 24.5, 23.6 (broad), 17.1; $^{11}B$ NMR ($CDCl_3$, 128 MHz): δ 31.89; HRMS (APCI) m/z ($[M+H]^+$) calcd for $C_{30}H_{42}BN_2O_4$: 505.3237, found: 505.3252.

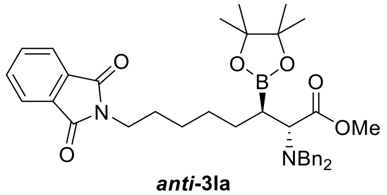

***anti*-3la**

**Methyl (2*R**,3*R**)-2-(dibenzylamino)-8-(1,3-dioxoisoindolin-2-yl)-3-(4,4,5,5-tetramethyl-1,3,2-dioxaborolan-2-yl)octanoate (*anti*-3la)**

It was purified by silica gel column chromatography with hexane/ethyl acetate (5/1, v/v) and GPC ($CHCl_3$): 98.4 mg (63%, 0.25 mmol scale); colorless oil; $^1H$ NMR ($CDCl_3$, 400 MHz): δ 7.87-7.83 (m, 2H), 7.74-7.69 (m, 2H), 7.33-7.26 (m, 8H), 7.20 (t, *J* = 6.9 Hz, 2H), 3.88 (d, *J* = 13.5 Hz, 2H), 3.75 (s, 3H), 3.63 (t, *J* = 7.4 Hz, 2H), 3.344 (d, *J* = 13.6 Hz, 2H), 3.343 (d, *J* = 12.1 Hz, 1H), 1.71-1.49 (m, 4H), 1.40-1.23 (m, 3H), 1.18 (s, 6H), 1.14 (s, 6H), 1.07-0.88 (m, 2H); $^{13}C\{^1H\}$ NMR ($CDCl_3$, 100 MHz): δ 173.4, 168.5, 139.7, 133.9, 132.3, 129.3, 128.2, 127.0, 123.2, 83.1, 61.5, 54.5, 50.9, 38.2, 28.7, 27.64, 27.58, 27.1, 25.0, 24.5, 23.6 (broad); $^{11}B$ NMR ($CDCl_3$, 128 MHz): δ 31.40; HRMS (APCI) m/z ($[M+H]^+$) calcd for $C_{37}H_{46}BN_2O_6$: 625.3450, found: 625.3459.

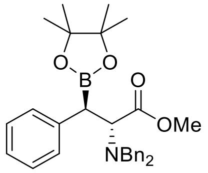

***anti*-3ma**

**Methyl (2*R**,3*R**)-2-(dibenzylamino)-3-phenyl-3-(4,4,5,5-tetramethyl-1,3,2-dioxaborolan-2-yl)propanoate (*anti*-3ma)**

It was purified by silica gel column chromatography with hexane/ethyl acetate (5/1, v/v) and GPC ($CHCl_3$): 76.5 mg (63%, 0.25 mmol scale); white solid; mp 116.5-117.5 °C; $^1H$ NMR ($CDCl_3$, 400 MHz): δ 7.23-7.19 (m, 3H), 7.17-7.13 (m, 6H), 6.97-6.95 (m, 6H), 3.86 (d, *J* = 13.8 Hz, 2H), 3.85 (d, *J* = 12.3 Hz, 1H), 3.80 (s, 3H), 3.38 (d, *J* = 13.8 Hz, 2H), 2.98 (d, *J* = 12.3 Hz, 1H), 1.12 (s, 6H), 1.05 (s, 6H);

$^{13}C\{^1H\}$ NMR ($CDCl_3$, 100 MHz): δ 173.4, 139.3, 139.2, 129.8, 129.1, 128.2, 128.1, 126.8, 125.7, 83.5, 63.2, 54.1, 51.2, 33.0 (broad), 24.5, 24.4; $^{11}B$ NMR ($CDCl_3$, 128 MHz): δ 32.80; HRMS (APCI) m/z ($[M+H]^+$) calcd for $C_{30}H_{37}BNO_4$: 486.2815, found: 486.2828.

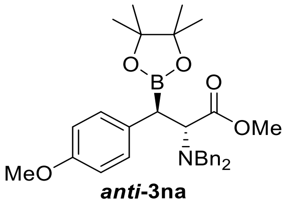

***anti*-3na**

**Methyl (2*R**,3*R**)-2-(dibenzylamino)-3-(4-methoxyphenyl)-3-(4,4,5,5-tetramethyl-1,3,2-dioxaborolan-2-yl)propanoate (*anti*-3na)**

It was purified by silica gel column chromatography with hexane/ethyl acetate (5/1, v/v) and GPC ($CHCl_3$): 87.6 mg (68%, 0.25 mmol scale); white solid; mp 106.1-107.1 °C; $^1H$ NMR ($CDCl_3$, 400 MHz): δ 7.18-7.14 (m, 6H), 7.01-6.97 (m, 4H), 6.87 (d, *J* = 8.7 Hz, 2H), 6.76 (d, *J* = 8.7 Hz, 2H), 3.86 (d, *J* = 13.8 Hz, 2H), 3.85 (s, 3H), 3.80 (s, 3H), 3.78 (d, *J* = 12.3 Hz, 1H), 3.38 (d, *J* = 13.8 Hz, 2H), 2.91 (d, *J* = 12.3 Hz, 1H), 1.13 (s, 6H), 1.06 (s, 6H); $^{13}C\{^1H\}$ NMR ($CDCl_3$, 100 MHz): δ 173.5, 157.9, 139.4, 131.2, 130.6, 129.0, 128.1, 126.8, 113.6, 83.4, 63.3, 55.4, 54.1, 51.2, 31.9 (broad), 24.5, 24.4; $^{11}B$ NMR ($CDCl_3$, 128 MHz): δ 31.09; HRMS (APCI) m/z ($[M+H]^+$) calcd for $C_{31}H_{39}BNO_5$: 516.2921, found: 516.2930.

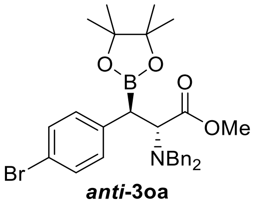

***anti*-3oa**

**Methyl (2*R**,3*R**)-3-(4-bromophenyl)-2-(dibenzylamino)-3-(4,4,5,5-tetramethyl-1,3,2-dioxaborolan-2-yl)propanoate (*anti*-3oa)**

It was purified by silica gel column chromatography with hexane/ethyl acetate (5/1, v/v) and GPC ($CHCl_3$): 86.1 mg (61%, 0.25 mmol scale); white solid; mp 151.7-152.7 °C; $^1H$ NMR ($CDCl_3$, 400 MHz): δ 7.29 (d, *J* = 8.4 Hz, 2H), 7.20-7.17 (m, 6H), 6.99-6.96 (m, 4H), 6.78 (d, *J* = 8.4 Hz, 2H), 3.83 (d, *J* = 13.8 Hz, 2H), 3.82 (s, 3H), 3.79 (d, *J* = 12.2 Hz, 1H), 3.37 (d, *J* = 13.8 Hz, 2H), 2.90 (d, *J* = 12.2 Hz, 1H), 1.12 (s, 6H), 1.05 (s, 6H); $^{13}C\{^1H\}$ NMR ($CDCl_3$, 100 MHz): δ 173.3, 139.1, 138.5, 131.4, 131.2, 129.1, 128.2, 127.0, 119.4, 83.7, 63.1, 54.2, 51.4, 32.6 (broad), 24.5, 24.4; $^{11}B$ NMR ($CDCl_3$, 128 MHz): δ 30.47; HRMS (APCI) m/z ($[M+H]^+$) calcd for $C_{30}H_{36}BBrNO_4$: 564.1920, found: 564.1927.

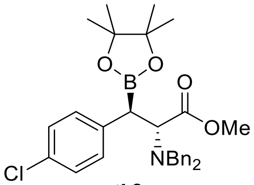


***anti*-3pa**

**Methyl (2*R**,3*R**)-3-(4-chlorophenyl)-2-(dibenzylamino)-3-(4,4,5,5-tetramethyl-1,3,2-dioxaborolan-2-yl)propanoate (*anti*-3pa)**

It was purified by silica gel column chromatography with hexane/ethyl acetate (5/1, v/v) and GPC ($CHCl_3$): 91.0 mg (70%, 0.25 mmol scale); white solid; mp 145.4-146.4 °C; $^1H$ NMR ($CDCl_3$, 400 MHz): δ 7.19-7.17 (m, 6H), 7.15 (d, *J* = 8.4 Hz, 2H), 6.99-6.97 (m, 4H), 6.84 (d, *J* = 8.4 Hz, 2H), 3.83 (d, *J* = 13.7 Hz, 2H), 3.82 (s, 3H), 3.79 (d, *J* = 12.2 Hz, 1H), 3.37 (d, *J* = 13.7 Hz, 2H), 2.92 (d, *J* = 12.2 Hz, 1H), 1.12 (s, 6H), 1.05 (s, 6H); $^{13}C\{^1H\}$ NMR ($CDCl_3$, 100 MHz): δ 173.3, 139.1, 137.9, 131.3, 130.9, 129.0, 128.2, 128.1, 126.9, 83.6, 63.1, 54.2, 51.3, 32.5 (broad), 24.5, 24.4; $^{11}B$ NMR ($CDCl_3$, 128 MHz): δ 30.90; HRMS (APCI) m/z ($[M+H]^+$) calcd for $C_{30}H_{36}BClNO_4$: 520.2426, found: 520.2402.

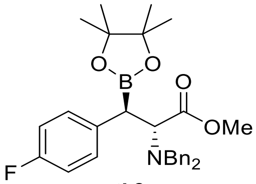


***anti*-3qa**

**Methyl (2*R**,3*R**)-2-(dibenzylamino)-3-(4-fluorophenyl)-3-(4,4,5,5-tetramethyl-1,3,2-dioxaborolan-2-yl)propanoate (*anti*-3qa)**

It was purified by silica gel column chromatography with hexane/ethyl acetate (5/1, v/v) and GPC ($CHCl_3$): 101.9 mg (81%, 0.25 mmol scale); white solid; mp 148.7-149.7 °C; $^1H$ NMR ($CDCl_3$, 400 MHz): δ 7.19-7.16 (m, 6H), 7.00-6.96 (m, 4H), 6.91-6.84 (m, 4H), 3.84 (d, *J* = 13.8 Hz, 2H), 3.82 (s, 3H), 3.79 (d, *J* = 12.3 Hz, 1H), 3.38 (d, *J* = 13.8 Hz, 2H), 2.93 (d, *J* = 12.3 Hz, 1H), 1.12 (s, 6H), 1.05 (s, 6H); $^{13}C\{^1H\}$ NMR ($CDCl_3$, 100 MHz): δ 173.3, 161.4 (d, *J* = 241.2 Hz), 139.2, 134.9 (d, *J* = 2.9 Hz), 130.9 (d, *J* = 7.6 Hz), 129.1, 128.2, 126.9, 114.9 (d, *J* = 21.0 Hz), 83.6, 63.1, 54.1, 51.3, 32.2 (broad), 24.5, 24.4; $^{19}F\{^1H\}$ NMR ($CDCl_3$, 376 MHz): δ −118.22 (s); $^{11}B$ NMR ($CDCl_3$, 128 MHz): δ 31.23; HRMS (APCI) m/z ($[M+H]^+$) calcd for $C_{30}H_{36}BFNO_4$: 504.2721, found: 504.2716.

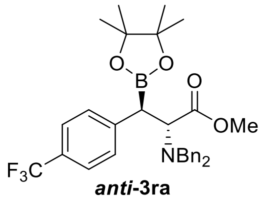


**Methyl (2*R**,3*R**)-2-(dibenzylamino)-3-(4,4,5,5-tetramethyl-1,3,2-dioxaborolan-2-yl)-3-(4-(trifluoromethyl)phenyl)propanoate (*anti*-3ra)**

It was purified by silica gel column chromatography with hexane/ethyl acetate (5/1, v/v) and GPC ($CHCl_3$): 71.9 mg (52%, 0.25 mmol scale); white solid; mp 89.1-90.1 °C; $^1$H NMR ($CDCl_3$, 400 MHz): δ 7.41 (d, *J* = 8.0 Hz, 2H), 7.20-7.13 (m, 6H), 6.99 (d, *J* = 8.0 Hz, 2H), 6.94-6.91 (m, 4H), 3.86 (d, *J* = 12.2 Hz, 1H), 3.84 (s, 3H), 3.82 (d, *J* = 13.7 Hz, 2H), 3.38 (d, *J* = 13.7 Hz, 2H), 3.01 (d, *J* = 12.2 Hz, 1H), 1.13 (s, 6H), 1.06 (s, 6H); $^{13}$C{$^1$H} NMR ($CDCl_3$, 100 MHz): δ 173.3, 143.9, 138.9, 129.8, 129.1, 128.1, 128.0 (q, *J* = 31.9 Hz), 127.0, 125.0 (q, *J* = 3.5 Hz), 124.7 (q, *J* = 270.0 Hz), 83.8, 63.2, 54.2, 51.4, 33.4 (broad), 24.5, 24.4; $^{19}$F{$^1$H} NMR ($CDCl_3$, 376 MHz): δ −62.09 (s); $^{11}$B NMR ($CDCl_3$, 128 MHz): δ 31.29; HRMS (APCI) m/z ([M+H]$^+$) calcd for $C_{31}H_{36}BF_3NO_4$: 554.2689, found: 554.2685.

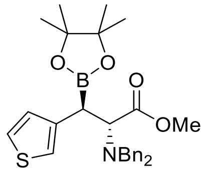


**Methyl (2*R**,3*R**)-2-(dibenzylamino)-3-(4,4,5,5-tetramethyl-1,3,2-dioxaborolan-2-yl)-3-(thiophen-3-yl)propanoate (*anti*-3sa)**

It was purified by silica gel column chromatography with hexane/ethyl acetate (5/1, v/v) and GPC ($CHCl_3$): 65.1 mg (53%, 0.25 mmol scale); white solid; mp 135.7-136.7 °C; $^1$H NMR ($CDCl_3$, 400 MHz): δ 7.22-7.15 (m, 7H), 7.05-7.03 (m, 4H), 6.76 (dd, *J* = 3.0, 1.2 Hz, 1H), 6.67 (dd, *J* = 4.9, 1.2 Hz, 1H), 3.86 (d, *J* = 13.9 Hz, 2H), 3.801 (d, *J* = 12.1 Hz, 1H), 3.798 (s, 3H), 3.41 (d, *J* = 13.9 Hz, 2H), 3.12 (d, *J* = 12.1 Hz, 1H), 1.14 (s, 6H), 1.07 (s, 6H); $^{13}$C{$^1$H} NMR ($CDCl_3$, 100 MHz): δ 173.4, 139.4, 138.9, 129.2, 129.0, 128.2, 126.9, 124.5, 121.6, 83.6, 63.2, 54.2, 51.3, 27.9 (broad), 24.5, 24.4; $^{11}$B NMR ($CDCl_3$, 128 MHz): δ 31.27; HRMS (APCI) m/z ([M+H]$^+$) calcd for $C_{28}H_{35}BNO_4S$: 492.2379, found: 492.2392.

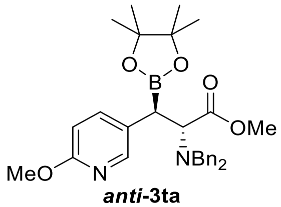

***anti*-3ta**

**Methyl (2*R**,3*R**)-2-(dibenzylamino)-3-(6-methoxypyridin-3-yl)-3-(4,4,5,5-tetramethyl-1,3,2-dioxaborolan-2-yl)propanoate (*anti*-3ta)**

It was purified by silica gel column chromatography with hexane/ethyl acetate (5/1, v/v) and GPC ($CHCl_3$): 102.0 mg (79%, 0.25 mmol scale); colorless oil; $^1$H NMR ($CDCl_3$, 400 MHz): δ 7.83 (d, *J* = 2.3 Hz, 1H), 7.20-7.17 (m, 6H), 7.03-6.98 (m, 5H), 6.56 (d, *J* = 8.5 Hz, 1H), 3.98 (s, 3H), 3.84 (d, *J* = 13.6 Hz, 2H), 3.82 (s, 3H), 3.73 (d, *J* = 12.2 Hz, 1H), 3.38 (d, *J* = 13.6 Hz, 2H), 2.86 (d, *J* = 12.2 Hz, 1H), 1.13 (s, 6H), 1.07 (s, 6H); $^{13}$C{$^1$H} NMR ($CDCl_3$, 100 MHz): δ 173.4, 162.7, 147.2, 139.6, 139.0, 129.0, 128.2, 127.6, 127.0, 110.2, 83.7, 63.1, 54.2, 53.5, 51.4, 29.1 (broad), 24.6, 24.4; $^{11}$B NMR ($CDCl_3$, 128 MHz): δ 32.43; HRMS (APCI) m/z ($[M+H]^+$) calcd for $C_{30}H_{38}BN_2O_5$: 517.2874, found: 517.2869.

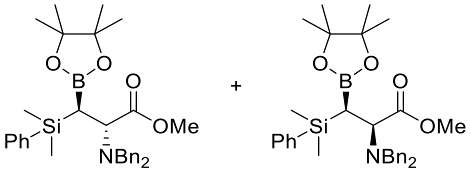

***anti*-3ua** + ***syn*-3ua**

**A 74:26 diastereomixture of Methyl (2*S**,3*S**)-2-(dibenzylamino)-3-(dimethyl(phenyl)silyl)-3-(4,4,5,5-tetramethyl-1,3,2-dioxaborolan-2-yl)propanoate (*anti*-3ua) and Methyl (2*R**,3*S**)-2-(dibenzylamino)-3-(dimethyl(phenyl)silyl)-3-(4,4,5,5-tetramethyl-1,3,2-dioxaborolan-2-yl)propanoate (*syn*-3ua)**

It was purified by silica gel column chromatography with hexane/ethyl acetate (5/1, v/v) and GPC ($CHCl_3$): 127.7 mg (94%, 0.25 mmol scale); colorless oil; $^1$H NMR ($CDCl_3$, 400 MHz): δ 7.45-7.16 (m, 0.74 × 15H for ***anti*-3ua** and 0.26 × 15H for ***syn*-3ua**), 4.06 (d, *J* = 14.0 Hz, 0.26 × 2H for ***syn*-3ua**), 3.78 (d, *J* = 12.8 Hz, 0.26H for ***syn*-3ua**), 3.77 (d, *J* = 13.7 Hz, 0.74× 2H for ***anti*-3ua**), 3.76 (d, *J* = 8.0 Hz, 0.74H for ***anti*-3ua**), 3.69 (s, 0.74× 3H for ***anti*-3ua**), 3.39 (d, *J* = 13.7 Hz, 0.74× 2H for ***anti*-3ua**), 3.24 (s, 0.26 × 3H for ***syn*-3ua**), 3.22 (d, *J* = 14.0 Hz, 0.26 × 2H for ***syn*-3ua**), 1.71 (d, *J* = 12.8 Hz, 0.26H for ***syn*-3ua**), 1.44 (d, *J* = 8.0 Hz, 0.74H for ***anti*-3ua**), 1.16 (s, 0.26 × 6H for ***syn*-3ua**), 1.11 (s, 0.74× 6H for ***anti*-3ua**), 1.08 (s, 0.26 × 6H for ***syn*-3ua**), 1.04 (s, 0.74× 6H for ***anti*-3ua**), 0.26 (s, 0.26 × 3H for ***syn*-3ua**), 0.22 (s, 0.26 × 3H for ***syn*-3ua**), 0.148 (s, 0.74× 3H for ***anti*-3ua**), 0.146 (s, 0.74× 3H for ***anti*-3ua**); $^{13}$C{$^1$H} NMR ($CDCl_3$, 100 MHz): δ 173.2, 171.8, 139.34, 139.30 (2C), 138.3, 134.1, 134.0, 129.5, 129.4,

129.0, 128.8, 128.1, 127.9, 127.7, 127.6, 126.9, 126.7, 83.2, 83.1, 60.22, 60.15, 54.5, 54.4, 50.8, 50.3, 25.9, 25.4, 25.2, 25.0, 15.6 (broad), 14.9 (broad), -1.8, -2.1, -2.3, -2.7; $^{11}$B NMR ($CDCl_3$, 128 MHz): δ 32.20; HRMS (APCI) m/z ($[M+H]^+$) calcd for $C_{32}H_{43}BNO_4Si$: 544.3055, found: 544.3044.

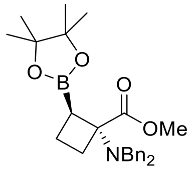

***anti*-3va**

**Methyl (1*R**,2*R**)-1-(dibenzylamino)-2-(4,4,5,5-tetramethyl-1,3,2-dioxaborolan-2-yl)cyclobutane-1-carboxylate (*anti*-3va)**

It was purified by silica gel column chromatography with hexane/ethyl acetate (5/1, v/v) and GPC ($CHCl_3$): 58.8 mg (54%, 0.25 mmol scale); white solid; mp 88.3-89.3 °C; $^1$H NMR ($CDCl_3$, 400 MHz): δ 7.23 (d, *J* = 7.2 Hz, 4H), 7.16 (t, *J* = 7.2 Hz, 4H), 7.09 (t, *J* = 7.2 Hz, 2H), 3.88 (d, *J* = 14.7 Hz, 2H), 3.72 (d, *J* = 14.7 Hz, 2H), 3.66 (s, 3H), 2.27-2.12 (m, 3H), 2.03-1.89 (m, 2H), 1.23 (s, 6H), 1.22 (s, 6H); $^{13}$C{$^1$H} NMR ($CDCl_3$, 100 MHz): δ 176.0, 140.8, 129.0, 127.8, 126.4, 83.3, 70.3, 53.9, 51.2, 32.7, 29.2 (broad), 25.1, 24.9, 16.0; $^{11}$B NMR ($CDCl_3$, 128 MHz): δ 32.35; HRMS (APCI) m/z ($[M+H]^+$) calcd for $C_{26}H_{35}BNO_4$: 436.2658, found: 436.2677.

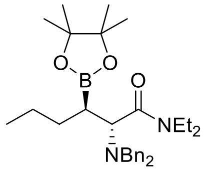

***anti*-3wa**

**(2*R**,3*R**)-2-(Dibenzylamino)-*N*,*N*-diethyl-3-(4,4,5,5-tetramethyl-1,3,2-dioxaborolan-2-yl)hexanamide (*anti*-3wa)**

It was purified by silica gel column chromatography with hexane/ethyl acetate (5/1, v/v) and GPC ($CHCl_3$): 94.8 mg (77%, 0.25 mmol scale); white solid; mp 92.0-93.0 °C; $^1$H NMR ($CDCl_3$, 400 MHz): δ 7.34-7.20 (m, 10H), 3.99 (dq, *J* = 14.5, 7.2 Hz, 1H), 3.72 (d, *J* = 13.8 Hz, 2H), 3.65 (dq, *J* = 14.5, 7.2 Hz, 1H), 3.61 (d, *J* = 13.8 Hz, 2H), 3.42 (d, *J* = 3.1 Hz, 1H), 2.94 (dq, *J* = 14.5, 7.2 Hz, 1H), 2.92 (dq, *J* = 14.5, 7.2 Hz, 1H), 1.63-1.42 (m, 3H), 1.38-1.34 (m, 1H), 1.26-1.18 (m, 1H), 1.21 (s, 6H), 1.20 (s, 6H), 1.05 (t, *J* =7.2 Hz, 3H), 0.96 (t, *J* = 7.2 Hz, 3H), 0.77 (t, *J* = 7.2 Hz, 3H); $^{13}$C{$^1$H} NMR ($CDCl_3$, 100 MHz): δ 176.8, 139.8, 129.5, 128.2, 127.1, 80.4, 67.5, 55.4, 42.1, 41.9, 34.9, 25.8, 25.2, 22.8, 22.7 (broad), 14.8, 13.6, 12.6; $^{11}$B NMR ($CDCl_3$, 128 MHz): δ 20.75; HRMS (APCI) m/z ($[M+H]^+$) calcd for

$C_{30}H_{46}BN_2O_3$: 493.3601, found: 493.3610.

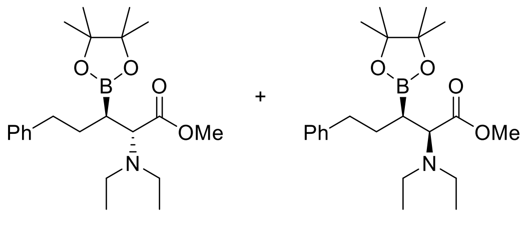

***anti*-3ab** ***syn*-3ab**

**A 90:10 diastereomixture of Methyl (2*R**,3*R**)-2-(diethylamino)-5-phenyl-3-(4,4,5,5-tetramethyl-1,3,2-dioxaborolan-2-yl)pentanoate (*anti*-3ab) and Methyl (2*S**,3*R**)-2-(diethylamino)-5-phenyl-3-(4,4,5,5-tetramethyl-1,3,2-dioxaborolan-2-yl)pentanoate (*syn*-3ab)**

It was purified by silica gel column chromatography with hexane/ethyl acetate (5/1, v/v) and GPC ($CHCl_3$): 54.5 mg (56%, 0.25 mmol scale); colorless oil; $^1$H NMR ($CDCl_3$, 400 MHz): δ 7.28-7.24 [(m, 0.90 × 2H for ***anti*-3ab** and 0.10 × 2H for ***syn*-3ab**)], 7.20-7.14 [(m, 0.90 × 3H for ***anti*-3ab** and 0.10 × 3H for ***syn*-3ab**)], 3.64 (s, 0.90 × 3H for ***anti*-3ab**), 3.63 (s, 0.10 × 3H for ***syn*-3ab**), 3.47 (d, *J* = 11.9 Hz, 0.10H for ***syn*-3ab**), 3.44 (d, *J* = 12.0 Hz, 0.90H for ***anti*-3ab**), 2.80-2.68 [(m, 0.90H for ***anti*-3ab** and 0.10 × 3H for ***syn*-3ab**)], 2.66-2.59 (m, 0.90 × 2H for ***anti*-3ab**), 2.56-2.48 [(m, 0.90H for ***anti*-3ab** and 0.10H for ***syn*-3ab**)], 2.43-2.35 (m, 0.90 × 2H for ***anti*-3ab**), 2.27-2.22 (m, 0.10 × 2H for ***syn*-3ab**), 2.08-1.99 [(m, 0.90H for ***anti*-3ab** and 0.10H for ***syn*-3ab**)], 1.73-1.64 [(m, 0.90H for ***anti*-3ab** and 0.10H for ***syn*-3ab**)], 1.50-1.44 [(m, 0.90H for ***anti*-3ab** and 0.10H for ***syn*-3ab**)], 1.30 (s, 0.10 × 6H for ***syn*-3ab**), 1.28 (s, 0.10 × 6H for ***syn*-3ab**), 1.27 (s, 0.90 × 6H for ***anti*-3ab**), 1.24 (s, 0.90 × 6H for ***anti*-3ab**), 1.04 (t, *J* = 7.1 Hz, 0.10 × 6H for ***syn*-3ab**), 0.99 (t, *J* = 7.1 Hz, 0.90 × 6H for ***anti*-3ab**); $^{13}C\{^1H\}$ NMR ($CDCl_3$, 100 MHz, for ***anti*-3ad**): δ 174.5, 143.3, 128.6, 128.4, 125.7, 83.3, 63.6, 50.9, 44.6, 35.0, 29.8, 25.2, 24.7, 24.0, 14.6 (broad); $^{11}$B NMR ($CDCl_3$, 128 MHz): δ 33.93; HRMS (APCI) m/z ($[M+H]^+$) calcd for $C_{22}H_{37}BNO_4$: 390.2814, found: 390.2825.

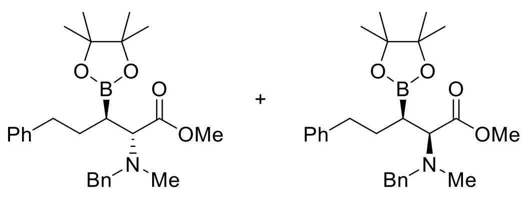

***anti*-3ac** ***syn*-3ac**

**An 85:15 diastereomixture of Methyl (2*R**,3*R**)-2-(benzyl(methyl)amino)-5-phenyl-3-(4,4,5,5-tetramethyl-1,3,2-dioxaborolan-2-yl)pentanoate (*anti*-3ac) and Methyl (2*S**,3*R**)-2-**

**(benzyl(methyl)amino)-5-phenyl-3-(4,4,5,5-tetramethyl-1,3,2-dioxaborolan-2-yl)pentanoate (*syn*-3ac)**

It was purified by silica gel column chromatography with hexane/ethyl acetate (5/1, v/v) and GPC ($CHCl_3$): 92.9 mg (85%, 0.25 mmol scale); colorless oil; $^1H$ NMR ($CDCl_3$, 400 MHz): δ 7.37-7.15 [(m, 0.85 × 10H for ***anti*-3ac** and 0.15 × 10H for ***syn*-3ac**)], 3.74 (d, *J* = 13.8 Hz, 0.85H for ***anti*-3ac**), 3.73 (s, 0.85 × 3H for ***anti*-3ac**), 3.70 (s, 0.15 × 3H for ***syn*-3ac**), 3.68 (d, *J* = 13.0 Hz, 0.15H for ***syn*-3ac**), 3.56 (d, *J* = 13.8 Hz, 0.85H for ***anti*-3ac**), 3.49 (d, *J* = 12.2 Hz, 0.85H for ***anti*-3ac**), 3.46 (d, *J* = 12.4 Hz, 0.15H for ***syn*-3ac**), 3.36 (d, *J* = 13.0 Hz, 0.15H for ***syn*-3ac**), 2.75-2.68 (m, 0.85 H for ***anti*-3ac**), 2.62-2.49 [(m, 0.85H for ***anti*-3ac** and 0.15 × 2H for ***syn*-3ac**)], 2.21 (s, 0.15 × 3H for ***syn*-3ac**), 2.12 (s, 0.85 × 3H for ***anti*-3ac**), 2.10-2.05 (m, 0.85H for ***anti*-3ac**), 1.93-1.61 [(m, 0.85 × 2H for ***anti*-3ac** and 0.15 × 3H for ***syn*-3ac**)], 1.30 (s, 0.15 × 6H for ***syn*-3ac**), 1.29 (s, 0.15 × 6H for ***syn*-3ac**), 1.28 (s, 0.85 × 6H for ***anti*-3ac**), 1.24 (s, 0.85 × 6H for ***anti*-3ac**); $^{13}C\{^1H\}$ NMR ($CDCl_3$, 100 MHz): δ 173.3, 172.0, 143.0, 142.6, 140.1, 139.2, 129.6, 128.7 (2C), 128.6, 128.4 (2C), 128.3, 128.0, 127.0, 126.9, 125.9, 125.8, 83.4 (2C), 68.6, 67.0, 59.4, 59.1, 50.9, 50.8, 38.4, 37.1, 35.9, 34.8, 31.0, 29.4, 25.2, 25.1, 25.0, 24.7, 23.7 (broad, 2C); $^{11}B$ NMR ($CDCl_3$, 128 MHz): δ 32.63; HRMS (APCI) m/z ([M+H]$^+$) calcd for $C_{26}H_{37}BNO_4$: 438.2815, found: 438.2832.

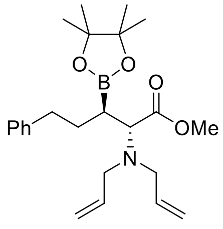

***anti*-3ad**

**Methyl (2*R**,3*R**)-2-(diallylamino)-5-phenyl-3-(4,4,5,5-tetramethyl-1,3,2-dioxaborolan-2-yl)pentanoate (*anti*-3ad)**

It was purified by silica gel column chromatography with hexane/ethyl acetate (5/1, v/v) and GPC ($CHCl_3$): 42.4 mg (41%, 0.25 mmol scale); colorless oil; $^1H$ NMR ($CDCl_3$, 400 MHz): δ 7.29-7.25 (m, 2H), 7.19-7.15 (m, 3H), 5.78-5.68 (m, 2H), 5.19-5.14 (m, 2H), 5.08-5.06 (m, 2H), 3.67 (s, 3H), 3.52 (d, *J* = 12.0 Hz, 1H), 3.33-3.28 (m, 2H), 2.93 (dd, *J* = 14.4, 8.0 Hz, 2H), 2.71 (ddd, *J* = 13.5, 11.8, 4.7 Hz, 1H), 2.49 (ddd, *J* = 13.5, 11.0, 6.3 Hz, 1H), 2.06-1.97 (m, 1H), 1.75-1.65 (m, 1H), 1.53-1.47 (m, 1H), 1.27 (s, 6H), 1.23 (s, 6H); $^{13}C\{^1H\}$ NMR ($CDCl_3$, 100 MHz): δ 174.0, 143.1, 136.9, 128.5, 128.4, 125.7, 117.0, 83.3, 62.7, 53.4, 51.0, 34.9, 29.6, 25.2, 24.7, 23.9 (broad); $^{11}B$ NMR ($CDCl_3$, 128 MHz): δ 32.96; HRMS (APCI) m/z ([M+H]$^+$) calcd for $C_{24}H_{37}BNO_4$: 414.2814, found: 414.2810.

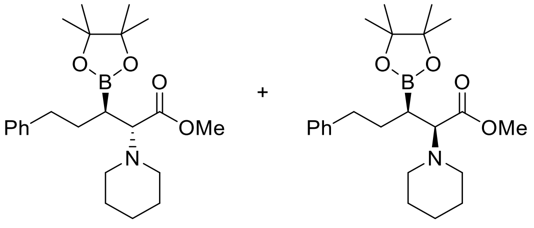

***anti*-3ae** **+** ***syn*-3ae**

**An 83:17 diastereomixture of Methyl (2*R**,3*R**)-5-phenyl-2-(piperidin-1-yl)-3-(4,4,5,5-tetramethyl-1,3,2-dioxaborolan-2-yl)pentanoate (*anti*-3ae) and Methyl (2*S**,3*R**)-5-phenyl-2-(piperidin-1-yl)-3-(4,4,5,5-tetramethyl-1,3,2-dioxaborolan-2-yl)pentanoate (*syn*-3ae)**

It was purified by silica gel column chromatography with hexane/ethyl acetate (5/1, v/v) and GPC ($CHCl_3$): 80.3 mg (80%, 0.25 mmol scale); colorless oil; $^1$H NMR ($CDCl_3$, 400 MHz): δ 7.29-7.23 [(m, 0.83 × 2H for ***anti*-3ae** and 0.17 × 2H for ***syn*-3ae**)], 7.21-7.14 [(m, 0.83 × 3H for ***anti*-3ae** and 0.17 × 3H for ***syn*-3ae**)], 3.66 (s, 0.83 × 3H for ***anti*-3ae**), 3.65 (s, 0.17 × 3H for ***syn*-3ae**), 3.28 (d, *J* = 12.1 Hz, 0.83H for ***anti*-3ae**), 3.23 (d, *J* = 12.1 Hz, 0.17H for ***syn*-3ae**), 2.73-2.60 [(m, 0.83 × 3H for ***anti*-3ae** and 0.17 × 3H for ***syn*-3ae**)], 2.56-2.48 [(m, 0.83H for ***anti*-3ae** and 0.17H for ***syn*-3ae**)], 2.32-2.24 [(m, 0.83 × 2H for ***anti*-3ae** and 0.17 × 2H for ***syn*-3ae**)], 2.01-1.92 (m, 0.83H for ***anti*-3ae**), 1.88-1.81 (m, 0.17H for ***syn*-3ae**), 1.78-1.38 [(m, 0.83 × 8H for ***anti*-3ae** and 0.17 × 8H for ***syn*-3ae**)], 1.32 (s, 0.17 × 6H for ***syn*-3ae**), 1.30 (s, 0.17 × 6H for ***syn*-3ae**), 1.26 (s, 0.83 × 6H for ***anti*-3ae**), 1.23 (s, 0.83 × 6H for ***anti*-3ae**); $^{13}$C{$^1$H} NMR ($CDCl_3$, 100 MHz): δ 173.0, 171.7, 143.3, 142.6, 128.6, 128.45, 128.39, 128.36, 125.8, 125.7, 83.3, 83.2, 70.6, 68.3, 50.7 (2C), 50.6 (2C), 36.0, 34.9, 31.0, 29.5, 26.9, 26.4, 25.5, 25.1, 25.0 (2C), 24.8, 24.6, 22.7 (broad, 2C); $^{11}$B NMR ($CDCl_3$, 128 MHz): δ 34.26; HRMS (APCI) m/z ($[M+H]^+$) calcd for $C_{23}H_{37}BNO_4$: 402.2814, found: 402.2817.

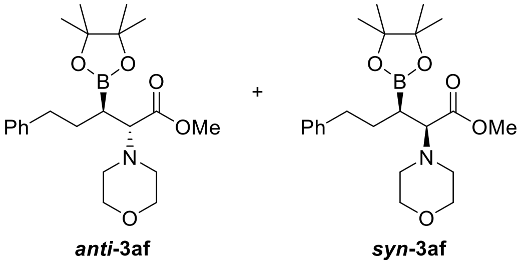

***anti*-3af** **+** ***syn*-3af**

**An 80:20 diastereomixture of Methyl (2*R**,3*R**)-2-morpholino-5-phenyl-3-(4,4,5,5-tetramethyl-1,3,2-dioxaborolan-2-yl)pentanoate (*anti*-3af) and Methyl (2*S**,3*R**)-2-morpholino-5-phenyl-3-(4,4,5,5-tetramethyl-1,3,2-dioxaborolan-2-yl)pentanoate (*syn*-3af)**

It was purified by silica gel column chromatography with hexane/ethyl acetate (5/1, v/v) and GPC

($CHCl_3$): 83.7 mg (83%, 0.25 mmol scale); white solid; mp 68.4-69.4 °C; $^1$H NMR ($CDCl_3$, 400 MHz): δ 7.29-7.24 [(m, 0.80 × 2H for ***anti*-3af** and 0.20 × 2H for ***syn*-3af**)], 7.20-7.14 [(m, 0.80 × 3H for ***anti*-3af** and 0.20 × 3H for ***syn*-3af**)], 3.675 (s, 0.80 × 3H for ***anti*-3af**), 3.667 (s, 0.20 × 3H for ***syn*-3af**), 3.65-3.58 [(m, 0.80 × 4H for ***anti*-3af** and 0.20 × 4H for ***syn*-3af**)], 3.29 (d, $J$ = 12.0 Hz, 0.80H for ***anti*-3af**), 3.26 (d, $J$ = 11.8 Hz, 0.20H for ***syn*-3af**), 2.75-2.48 [(m, 0.80 × 4H for ***anti*-3af** and 0.20 × 4H for ***syn*-3af**)], 2.45-2.42 (m, 0.20 × 2H for ***syn*-3af**), 2.36-2.31 (m, 0.80 × 2H for ***anti*-3af**), 2.05-1.95 (m, 0.80H for ***anti*-3af**), 1.85-1.64 [(m, 0.80H for ***anti*-3af** and 0.20 × 2H for ***syn*-3af**)], 1.59-1.52 [(m, 0.80H for ***anti*-3af** and 0.20H for ***syn*-3af**)], 1.32 (s, 0.20 × 6H for ***syn*-3af**), 1.31 (s, 0.20 × 6H for ***syn*-3af**), 1.27 (s, 0.80 × 6H for ***anti*-3af**), 1.23 (s, 0.80 × 6H for ***anti*-3af**); $^{13}$C{$^1$H} NMR ($CDCl_3$, 100 MHz): δ 172.5, 171.2, 142.9, 142.3, 128.5, 128.4 (2C), 128.3, 125.9, 125.8, 83.4, 83.3, 70.0, 67.8, 67.7, 67.3, 50.9 (2C), 50.3, 49.6, 35.8, 34.7, 30.7, 29.2, 25.4, 25.1, 25.0, 24.6, 22.1 (broad, 2C); $^{11}$B NMR ($CDCl_3$, 128 MHz): δ 32.62; HRMS (APCI) m/z ([M+H]$^+$) calcd for $C_{22}H_{35}BNO_5$: 404.2607, found: 404.2604.

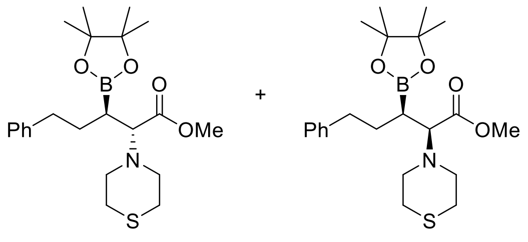

*anti*-3ag *syn*-3ag

**An 84:16 diastereomixture of Methyl (2*R**,3*R**)-5-phenyl-3-(4,4,5,5-tetramethyl-1,3,2-dioxaborolan-2-yl)-2-thiomorpholinopentanoate (*anti*-3ag) and Methyl (2*S**,3*R**)-5-phenyl-3-(4,4,5,5-tetramethyl-1,3,2-dioxaborolan-2-yl)-2-thiomorpholinopentanoate (*syn*-3ag)**

It was purified by silica gel column chromatography with hexane/ethyl acetate (5/1, v/v) and GPC ($CHCl_3$): 79.7 mg (76%, 0.25 mmol scale); colorless oil; $^1$H NMR ($CDCl_3$, 400 MHz): δ 7.30-7.24 [(m, 0.84 × 2H for ***anti*-3ag** and 0.16 × 2H for ***syn*-3ag**)], 7.20-7.14 [(m, 0.84 × 3H for ***anti*-3ag** and 0.16 × 3H for ***syn*-3ag**)], 3.68 (s, 0.84 × 3H for ***anti*-3ag**), 3.67 (s, 0.16 × 3H for ***syn*-3ag**), 3.25 (d, $J$ = 12.2 Hz, 0.84H for ***anti*-3ag**), 3.20 (d, $J$ = 12.2 Hz, 0.16H for ***syn*-3ag**), 3.01-2.92 [(m, 0.84 × 2H for ***anti*-3ag** and 0.16 × 2H for ***syn*-3ag**)], 2.73-2.47 [(m, 0.84 × 8H for ***anti*-3ag** and 0.16 × 8H for ***syn*-3ag**)], 2.00-1.91 (m, 0.84H for ***anti*-3ag**), 1.86-1.79 (m, 0.16H for ***syn*-3ag**), 1.76-1.65 [(m, 0.84H for ***anti*-3ag** and 0.16H for ***syn*-3ag**)], 1.57-1.50 [(m, 0.84H for ***anti*-3ag** and 0.16H for ***syn*-3ag**)], 1.33 (s, 0.16 × 6H for ***syn*-3ag**), 1.31 (s, 0.16 × 6H for ***syn*-3ag**), 1.27 (s, 0.84 × 6H for ***anti*-3ag**), 1.23 (s, 0.84 × 6H for ***anti*-3ag**); $^{13}$C{$^1$H} NMR ($CDCl_3$, 100 MHz): δ 172.8, 171.2, 142.9, 142.3, 128.5, 128.4 (3C), 125.9, 125.8, 83.35, 83.27, 71.2, 69.0, 52.6, 51.9, 51.01, 50.95, 35.8, 34.6, 30.8, 29.4, 28.8, 28.3, 25.6, 25.1, 25.0, 24.6, 22.4

(broad, 2C); $^{11}$B NMR ($CDCl_3$, 128 MHz): δ 33.06; HRMS (APCI) m/z ($[M+H]^+$) calcd for $C_{22}H_{35}BNO_4S$: 420.2378, found: 420.2375.

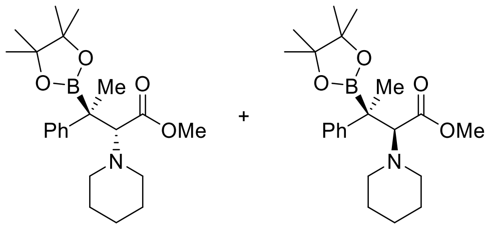

***anti*-3Ae** **syn-3Ae**

**A 72:28 diastereomixture of Methyl (2*R**,3*S**)-3-phenyl-2-(piperidin-1-yl)-3-(4,4,5,5-tetramethyl-1,3,2-dioxaborolan-2-yl)butanoate (*anti*-3Ae) and Methyl (2*S**,3*S**)-3-phenyl-2-(piperidin-1-yl)-3-(4,4,5,5-tetramethyl-1,3,2-dioxaborolan-2-yl)butanoate (*syn*-3Ae) (The relative stereochemistry was tentatively assigned.)**

It was purified by silica gel column chromatography with hexane/ethyl acetate (5/1, v/v) and GPC ($CHCl_3$): 83.3 mg (86%, 0.25 mmol scale); colorless oil; $^{1}$H NMR ($CDCl_3$, 400 MHz): δ 7.48 (d, $J$ = 7.3 Hz, 0.72 × 2H for ***anti*-3Ae**), 7.46 (d, $J$ = 7.3 Hz, 0.28 × 2H for ***syn*-3Ae**), 7.25-7.21 [(m, 0.72 × 2H for ***anti*-3Ae** and 0.28 × 2H for ***syn*-3Ae**)], 7.13 (t, $J$ = 7.3 Hz, 0.28H for ***syn*-3Ae**), 7.11 (t, $J$ = 7.3 Hz, 0.72H for ***anti*-3Ae**), 3.75 (s, 0.72H for ***anti*-3Ae**), 3.73 (s, 0.28H for ***syn*-3Ae**), 3.72 (s, 0.28 × 3H for ***syn*-3Ae**), 3.44 (s, 0.72 × 3H for ***anti*-3Ae**), 2.58-2.53 [(m, 0.72 × 2H for ***anti*-3Ae** and 0.28 × 2H for ***syn*-3Ae**)], 2.41 (br, 0.72 × 2H for ***anti*-3Ae**), 2.11-2.06 (m, 0.28 × 2H for ***syn*-3Ae**), 1.59 (s, 0.72 × 3H for ***anti*-3Ae**), 1.53-1.47 [(m, 0.72 × 3H for ***anti*-3Ae** and 0.28 × 3H for ***syn*-3Ae**)], 1.51 (s, 0.28 × 3H for ***syn*-3Ae**), 1.40-1.27 [(m, 0.72 × 3H for ***anti*-3Ae** and 0.28 × 3H for ***syn*-3Ae**)], 1.22 (s, 0.28 × 6H for ***syn*-3Ae**), 1.20 (s, 0.72 × 6H for ***anti*-3Ae**), 1.19 (s, 0.72 × 6H for ***anti*-3Ae**), 1.14 (s, 0.28 × 6H for ***syn*-3Ae**); $^{13}$C{$^{1}$H} NMR ($CDCl_3$, 100 MHz): δ 173.5, 171.8, 144.7, 142.6, 128.3, 128.0, 127.6, 127.5, 125.6, 125.3, 83.5, 83.4, 74.4, 74.2, 54.0 (2C), 50.8, 50.4, 35.0 (broad, 2C), 26.8, 26.7, 24.8, 24.7, 24.6, 24.5, 24.4, 24.3, 16.6, 15.6; $^{11}$B NMR ($CDCl_3$, 128 MHz): δ 32.61; HRMS (APCI) m/z ($[M+H]^+$) calcd for $C_{22}H_{35}BNO_4$: 388.2658, found: 388.2656.

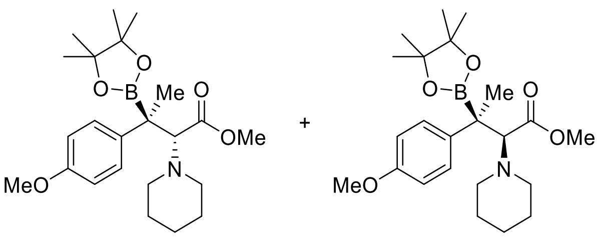

***anti*-3Be** ***syn*-3Be**

**A 70:30 diastereomixture of Methyl (2*R**,3*S**)-3-(4-methoxyphenyl)-2-(piperidin-1-yl)-3-(4,4,5,5-tetramethyl-1,3,2-dioxaborolan-2-yl)butanoate (*anti*-3Be) and Methyl (2*S**,3*S**)-3-(4-methoxyphenyl)-2-(piperidin-1-yl)-3-(4,4,5,5-tetramethyl-1,3,2-dioxaborolan-2-yl)butanoate (*syn*-3Be) (The relative stereochemistry was tentatively assigned.)**

It was purified by silica gel column chromatography with hexane/ethyl acetate (5/1, v/v) and GPC ($CHCl_3$): 77.2 mg (74%, 0.25 mmol scale); colorless oil; $^1$H NMR ($CDCl_3$, 400 MHz): δ 7.41 (d, $J$ = 9.0 Hz, 0.70 × 2H for ***anti*-3Be**), 7.38 (d, $J$ = 9.0 Hz, 0.30 × 2H for ***syn*-3Be**), 6.81 (d, $J$ = 9.0 Hz, 0.30 × 2H for ***syn*-3Be**), 6.78 (d, $J$ = 9.0 Hz, 0.70 × 2H for ***anti*-3Be**), 3.79 (s, 0.30 × 3H for ***syn*-3Be**), 3.76 (s, 0.70 × 3H for ***anti*-3Be**), 3.71 (s, 0.30 × 3H for ***syn*-3Be**), 3.66 [(s, 0.70H for ***anti*-3Be** and 0.30H for ***syn*-3Be**)], 3.46 (s, 0.70 × 3H for ***anti*-3Be**), 2.58-2.49 [(m, 0.70 × 2H for ***anti*-3Be** and 0.30 × 2H for ***syn*-3Be**)], 2.38 (br, 0.70 × 2H for ***anti*-3Be**), 2.12-2.07 (m, 0.30 × 2H for ***syn*-3Be**), 1.55 (s, 0.70 × 3H for ***anti*-3Be**), 1.52-1.46 [(m, 0.70 × 3H for ***anti*-3Be** and 0.30 × 2H for ***syn*-3Be**)], 1.47 (s, 0.30 × 3H for ***syn*-3Be**), 1.37-1.29 [(m, 0.70 × 3H for ***anti*-3Be** and 0.30 × 4H for ***syn*-3Be**)], 1.22 (s, 0.30 × 6H for ***syn*-3Be**), 1.20 (s, 0.70 × 6H for ***anti*-3Be**), 1.19 (s, 0.70 × 6H for ***anti*-3Be**), 1.15 (s, 0.30 × 6H for ***syn*-3Be**); $^{13}$C{$^1$H} NMR ($CDCl_3$, 100 MHz): δ 173.4, 172.1, 157.5, 157.3, 136.6, 134.5, 129.5, 129.0, 112.90, 112.86, 83.41, 83.37, 74.33, 74.27, 55.16, 55.15, 54.0 (2C), 50.7, 50.4, 33.9 (broad, 2C), 26.8 (2C), 24.8, 24.7 (2C), 24.5, 24.4, 24.3, 17.0, 15.9; $^{11}$B NMR ($CDCl_3$, 128 MHz): δ 32.42; HRMS (APCI) m/z ([M+H]$^+$) calcd for $C_{23}H_{37}BNO_5$: 418.2763, found: 418.2769.

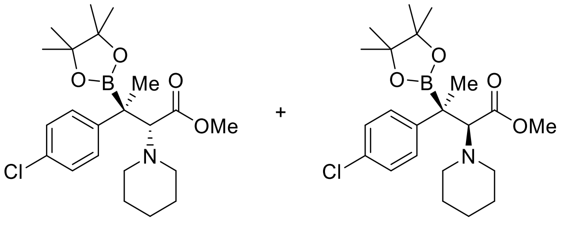

***anti*-3Ce** ***syn*-3Ce**

**A 68:32 diastereomixture of Methyl (2*R**,3*S**)-3-(4-chlorophenyl)-2-(piperidin-1-yl)-3-(4,4,5,5-tetramethyl-1,3,2-dioxaborolan-2-yl)butanoate (*anti*-3Ce) and Methyl (2*S**,3*S**)-3-(4-**

**chlorophenyl)-2-(piperidin-1-yl)-3-(4,4,5,5-tetramethyl-1,3,2-dioxaborolan-2-yl)butanoate (*syn*-3Ce) (The relative stereochemistry was tentatively assigned.)**

It was purified by silica gel column chromatography with hexane/ethyl acetate (5/1, v/v) and GPC ($CHCl_3$): 53.8 mg (51%, 0.25 mmol scale); colorless oil; $^1$H NMR ($CDCl_3$, 400 MHz): δ 7.44 (d, $J$ = 8.8 Hz, 0.68 × 2H for ***anti*-3Ce**), 7.40 (d, $J$ = 8.8 Hz, 0.32 × 2H for ***syn*-3Ce**), 7.22 (d, $J$ = 8.8 Hz, 0.32 × 2H for ***syn*-3Ce**), 6.78 (d, $J$ = 8.8 Hz, 0.68 × 2H for ***anti*-3Ce**), 3.71 (s, 0.32 × 3H for ***syn*-3Ce**), 3.69 (s, 0.32H for ***syn*-3Ce**), 3.66 (s, 0.68H for ***anti*-3Ce**), 3.50 (s, 0.68 × 3H for ***anti*-3Ce**), 2.57-2.55 (m, 0.32 × 2H for ***syn*-3Ce**), 2.50-2.46 (m, 0.68 × 2H for ***anti*-3Ce**), 2.36 (br, 0.68 × 2H for ***anti*-3Ce**), 2.13-2.08 (m, 0.32 × 2H for ***syn*-3Ce**), 1.54 (s, 0.68 × 3H for ***anti*-3Ce**), 1.50-1.45 [(m, 0.68 × 3H for ***anti*-3Ce** and 0.32 × 2H for ***syn*-3Ce**)], 1.47 (s, 0.32 × 3H for ***syn*-3Ce**), 1.38-1.27 [(m, 0.68 × 3H for ***anti*-3Ce** and 0.32 × 4H for ***syn*-3Ce**)], 1.22 (s, 0.32 × 6H for ***syn*-3Ce**), 1.20 (s, 0.68 × 12H for ***anti*-3Ce**), 1.15 (s, 0.32 × 6H for ***syn*-3Ce**); $^{13}C\{^1H\}$ NMR ($CDCl_3$, 100 MHz): δ 173.2, 172.0, 143.2, 141.3, 131.4, 131.1, 130.2, 129.6, 127.6 (2C), 83.62, 83.61, 74.3, 74.2, 54.0 (2C), 50.8, 50.5, 34.6 (broad, 2C), 26.81, 26.77, 24.8, 24.7 (2C), 24.5, 24.34, 24.29, 17.3, 15.8; $^{11}$B NMR ($CDCl_3$, 128 MHz): δ 33.79; HRMS (APCI) m/z ($[M+H]^+$) calcd for $C_{22}H_{34}BClNO_4$: 422.2268, found: 422.2278.

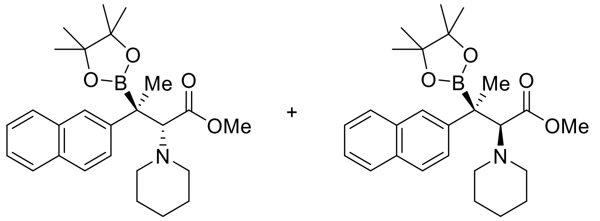


***anti*-3De** ***syn*-3De**

**A 76:24 diastereomixture of Methyl (2*R**,3*S**)-3-(naphthalen-2-yl)-2-(piperidin-1-yl)-3-(4,4,5,5-tetramethyl-1,3,2-dioxaborolan-2-yl)butanoate (*anti*-3De) and Methyl (2*S**,3*S**)-3-(naphthalen-2-yl)-2-(piperidin-1-yl)-3-(4,4,5,5-tetramethyl-1,3,2-dioxaborolan-2-yl)butanoate (*syn*-3De) (The relative stereochemistry was tentatively assigned.)**

It was purified by silica gel column chromatography with hexane/ethyl acetate (5/1, v/v) and GPC ($CHCl_3$): 66.7 mg (61%, 0.25 mmol scale); colorless oil; $^1$H NMR ($CDCl_3$, 400 MHz): δ 7.88-7.86 [(m, 0.76H for ***anti*-3De** and 0.24H for ***syn*-3De**)], 7.81-7.67 [(m, 0.76 × 4H for ***anti*-3De** and 0.24 × 4H for ***syn*-3De**)], 7.44-7.36 [(m, 0.76 × 2H for ***anti*-3De** and 0.24 × 2H for ***syn*-3De**)], 3.89 (s, 0.24H for ***syn*-3De**), 3.88 (s, 0.76H for ***anti*-3De**), 3.74 (s, 0.24 × 3H for ***syn*-3De**), 3.40 (s, 0.76 × 3H for ***anti*-3De**), 2.63-2.58 [(m, 0.76 × 2H for ***anti*-3De** and 0.24 × 2H for ***syn*-3De**)], 2.45 (br, 0.76 × 2H for ***anti*-3De**), 2.13-2.09 (m, 0.24 × 2H for ***syn*-3De**), 1.71 (s, 0.76 × 3H for ***anti*-3De**), 1.63 (s, 0.24 × 3H for ***syn*-3De**),

1.54-1.48 [(m, 0.76 × 3H for ***anti*-3De** and 0.24 × 3H for ***syn*-3De**)], 1.40-1.36 (m, 0.76 × 2H for ***anti*-3De**), 1.27-1.23 [(m, 0.76H for ***anti*-3De** and 0.24 × 3H for ***syn*-3De**)], 1.23 (s, 0.24 × 6H for ***syn*-3De**), 1.21 (s, 0.76 × 6H for ***anti*-3De**), 1.19 (s, 0.76 × 6H for ***anti*-3De**), 1.14 (s, 0.24 × 6H for ***syn*-3De**); $^{13}C\{^1H\}$ NMR ($CDCl_3$, 100 MHz): δ 173.5, 171.8, 142.4, 140.5, 133.5, 133.4, 131.9, 131.8, 128.2, 128.1, 127.36, 127.33, 127.30, 127.1, 126.8, 126.7, 126.6, 126.4, 125.41, 125.37, 125.12, 125.05, 83.57, 83.56, 74.20, 74.16, 54.1, 54.0, 50.8, 50.4, 35.4 (broad, 2C), 26.8, 26.7, 24.9, 24.7, 24.6, 24.5, 24.4, 24.3, 16.7, 15.9; $^{11}B$ NMR ($CDCl_3$, 128 MHz): δ 32.92; HRMS (APCI) m/z ($[M+H]^+$) calcd for $C_{26}H_{37}BNO_4$: 438.2815, found: 438.2828.

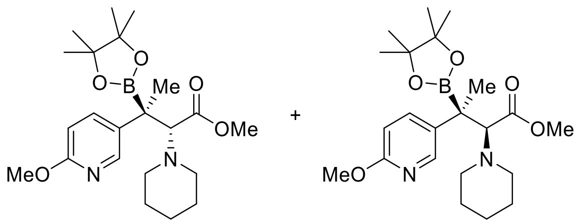

***anti*-3Ee** ***syn*-3Ee**

**A 65:35 diastereomixture of Methyl (2*R**,3*S**)-3-(6-methoxypyridin-3-yl)-2-(piperidin-1-yl)-3-(4,4,5,5-tetramethyl-1,3,2-dioxaborolan-2-yl)butanoate (*anti*-3Ee) and Methyl (2*S**,3*S**)-3-(6-methoxypyridin-3-yl)-2-(piperidin-1-yl)-3-(4,4,5,5-tetramethyl-1,3,2-dioxaborolan-2-yl)butanoate (*syn*-3Ee) (The relative stereochemistry was tentatively assigned.)**

It was purified by silica gel column chromatography with hexane/ethyl acetate (5/1, v/v) and GPC ($CHCl_3$): 77.4 mg (74%, 0.25 mmol scale); colorless oil; $^1H$ NMR ($CDCl_3$, 400 MHz): δ 8.30 (d, $J$ = 2.6 Hz, 0.65H for ***anti*-3Ee**), 8.21 (d, $J$ = 2.6 Hz, 0.35H for ***syn*-3Ee**), 7.81 (dd, $J$ = 8.7, 2.6 Hz, 0.65H for ***anti*-3Ee**), 7.74 (dd, $J$ = 8.7, 2.6 Hz, 0.35H for ***syn*-3Ee**), 6.66 (d, $J$ = 8.7 Hz, 0.35H for ***syn*-3Ee**), 6.63 (d, $J$ = 8.7 Hz, 0.65H for ***anti*-3Ee**), 3.92 (s, 0.35 × 3H for ***syn*-3Ee**), 3.90 (s, 0.65 × 3H for ***anti*-3Ee**), 3.71 (s, 0.35 × 3H for ***syn*-3Ee**), 3.62 (s, 0.35H for ***syn*-3Ee**), 3.57 (s, 0.65 × 3H for ***anti*-3Ee**), 3.52 (s, 0.65H for ***anti*-3Ee**), 2.56-2.52 (m, 0.35 × 2H for ***syn*-3Ee**), 2.38-2.30 (m, 0.65 × 4H for ***anti*-3Ee**), 2.17-2.13 (m, 0.35 × 2H for ***syn*-3Ee**), 1.50 (s, 0.65 × 3H for ***anti*-3Ee**), 1.47-1.29 [(m, 0.65 × 6H for ***anti*-3Ee** and 0.35 × 6H for ***syn*-3Ee**)], 1.46 (s, 0.35 × 3H for ***syn*-3Ee**), 1.24 (s, 0.35 × 6H for ***syn*-3Ee**), 1.23 (s, 0.65 × 6H for ***anti*-3Ee**), 1.21 (s, 0.65 × 6H for ***anti*-3Ee**), 1.18 (s, 0.35 × 6H for ***syn*-3Ee**); $^{13}C\{^1H\}$ NMR ($CDCl_3$, 100 MHz): δ 173.0, 172.4, 162.33, 162.27, 147.3, 146.3, 140.0, 139.1, 132.4, 130.5, 109.2, 109.0, 83.7, 83.6, 74.3, 73.9, 53.9 (2C), 53.35, 53.31, 50.8, 50.6, 32.7 (broad), 31.9 (broad), 26.71, 26.69, 24.8, 24.74, 24.69, 24.56, 24.3 (2C), 18.6, 16.0; $^{11}B$ NMR ($CDCl_3$, 128 MHz): δ 32.84; HRMS (APCI) m/z ($[M+H]^+$) calcd for $C_{22}H_{36}BN_2O_5$: 419.2716, found: 419.2733.

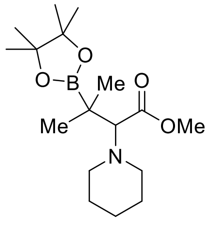

**3Fe**

**Methyl 3-methyl-2-(piperidin-1-yl)-3-(4,4,5,5-tetramethyl-1,3,2-dioxaborolan-2-yl)butanoate (3Fe)**

It was purified by silica gel column chromatography with hexane/ethyl acetate (5/1, v/v) and GPC ($CHCl_3$): 70.7 mg (87%, 0.25 mmol scale); colorless oil; $^1$H NMR ($CDCl_3$, 400 MHz): δ 3.68 (s, 3H), 3.15 (s, 1H), 2.72-2.67 (m, 2H), 2.42-2.37 (m, 2H), 1.56-1.50 (m, 4H), 1.42-1.36 (m, 2H), 1.23 (s, 6H), 1.20 (s, 6H), 1.03 (s, 3H), 0.98 (s, 3H); $^{13}$C{$^1$H} NMR ($CDCl_3$, 100 MHz): δ 173.2, 83.0, 74.3, 53.7, 50.6, 26.8, 24.8, 24.6, 24.5, 21.7, 20.8 (The carbon signal bound to boron was not observed due to quadrupolar relaxation.); $^{11}$B NMR ($CDCl_3$, 128 MHz): δ 33.84; HRMS (APCI) m/z ($[M+H]^+$) calcd for $C_{17}H_{33}BNO_4$: 326.2500, found: 326.2497.

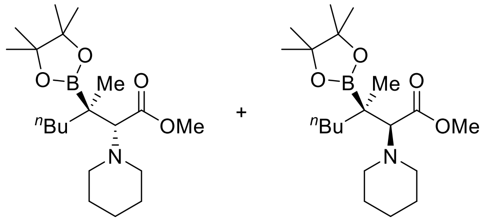

***anti*-3Ge** ***syn*-3Ge**

**A 58:42 diastereomixture of Methyl (2*R**,3*S**)-3-methyl-2-(piperidin-1-yl)-3-(4,4,5,5-tetramethyl-1,3,2-dioxaborolan-2-yl)heptanoate (*anti*-3Ge) and Methyl (2*S**,3*S**)-3-methyl-2-(piperidin-1-yl)-3-(4,4,5,5-tetramethyl-1,3,2-dioxaborolan-2-yl)heptanoate (*syn*-3Ge) (The relative stereochemistry was tentatively assigned.)**

It was purified by silica gel column chromatography with hexane/ethyl acetate (5/1, v/v) and GPC ($CHCl_3$): 79.0 mg (86%, 0.25 mmol scale); colorless oil; $^1$H NMR ($CDCl_3$, 400 MHz): δ 3.68 (s, 0.42 × 3H for ***syn*-3Ge**), 3.67 (s, 0.58 × 3H for ***anti*-3Ge**), 3.22 (s, 0.42H for ***syn*-3Ge**), 3.17 (s, 0.58H for ***anti*-3Ge**), 2.80-2.71 [(m, 0.58 × 2H for ***anti*-3Ge** and 0.42 × 2H for ***syn*-3Ge**)], 2.41-2.36 [(m, 0.58 × 2H for ***anti*-3Ge** and 0.42 × 2H for ***syn*-3Ge**)], 1.53-1.35 [(m, 0.58 × 8H for ***anti*-3Ge** and 0.42 × 8H for ***syn*-3Ge**)], 1.30-1.22 [(m, 0.58 × 2H for ***anti*-3Ge** and 0.42 × 2H for ***syn*-3Ge**)], 1.25 (s, 0.58 × 12H for ***anti*-**

**3Ge**), 1.22 (s, 0.42 × 6H for ***syn*-3Ge**), 1.19 (s, 0.42 × 6H for ***syn*-3Ge**), 1.19-1.08 [(m, 0.58 × 2H for ***anti*-3Ge** and 0.42 × 2H for ***syn*-3Ge**)], 1.08 (s, 0.58 × 3H for ***anti*-3Ge**), 1.04 (s, 0.42 × 3H for ***syn*-3Ge**), 0.89 (t, $J$ = 6.8 Hz, 0.42 × 3H for ***syn*-3Ge**), 0.87 (t, $J$ = 7.2 Hz, 0.58 × 3H for ***anti*-3Ge**); $^{13}C\{^1H\}$ NMR ($CDCl_3$, 100 MHz): δ 173.7, 172.2, 83.2, 83.1, 75.0, 72.9, 54.1, 53.5, 50.5, 50.4, 36.9, 36.5, 29.4 (broad, 2C), 28.3, 28.1, 26.9, 26.7, 25.2, 25.1, 25.0, 24.9, 24.6, 24.5, 23.9, 23.8, 17.8, 17.3, 14.3, 14.2; $^{11}B$ NMR ($CDCl_3$, 128 MHz): δ 33.70; HRMS (APCI) m/z ($[M+H]^+$) calcd for $C_{20}H_{39}BNO_4$: 368.2970, found: 368.2963.

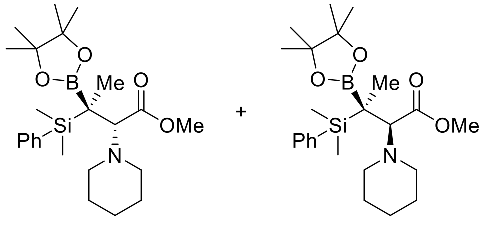

***anti*-3He** ***syn*-3He**

**A 58:42 diastereomixture of Methyl (2*S**,3*R**)-3-(dimethyl(phenyl)silyl)-2-(piperidin-1-yl)-3-(4,4,5,5-tetramethyl-1,3,2-dioxaborolan-2-yl)butanoate (*anti*-3He) and Methyl (2*R**,3*S**)-3-(dimethyl(phenyl)silyl)-2-(piperidin-1-yl)-3-(4,4,5,5-tetramethyl-1,3,2-dioxaborolan-2-yl)butanoate (*syn*-3He) (The relative stereochemistry was tentatively assigned.)**

It was purified by silica gel column chromatography with hexane/ethyl acetate (5/1, v/v) and GPC ($CHCl_3$): 82.4 mg (74%, 0.25 mmol scale); colorless oil; $^1H$ NMR ($CDCl_3$, 400 MHz): δ 7.65-7.63 (m, 0.44 × 2H for ***syn*-3He**), 7.61-7.58 (m, 0.56 × 2H for ***anti*-3He**), 7.32-7.29 [(m, 0.56 × 3H for ***anti*-3He** and 0.44 × 3H for ***syn*-3He**)], 3.63 (s, 0.44 × 3H for ***syn*-3He**), 3.57 (s, 0.56 × 3H for ***anti*-3He**), 3.50 (s, 0.44H for ***syn*-3He**), 2.99 (br, 0.56 × 2H for ***anti*-3He**), 2.95 (s, 0.56H for ***anti*-3He**), 2.72-2.68 (m, 0.44 × 2H for ***syn*-3He**), 2.18-2.08 [(m, 0.56 × 2H for ***anti*-3He** and 0.44 × 2H for ***syn*-3He**)], 1.57-1.51 [(m, 0.56 × 3H for ***anti*-3He** and 0.44 × 3H for ***syn*-3He**)], 1.43 (s, 0.44 × 3H for ***syn*-3He**), 1.41-1.33 [(m, 0.56 × 3H for ***anti*-3He** and 0.44 × 3H for ***syn*-3He**)], 1.27 (s, 0.56 × 6H for ***anti*-3He**), 1.21 (s, 0.44 × 6H for ***syn*-3He**), 1.20 (s, 0.56 × 6H for ***anti*-3He**), 1.10 (s, 0.56 × 3H for ***anti*-3He**), 1.07 (s, 0.44 × 6H for ***syn*-3He**), 0.48 (s, 0.44 × 3H for ***syn*-3He**), 0.47 (s, 0.56 × 3H for ***anti*-3He**), 0.36 (s, 0.56 × 3H for ***anti*-3He**), 0.34 (s, 0.44 × 3H for ***syn*-3He**); $^{13}C\{^1H\}$ NMR ($CDCl_3$, 100 MHz): δ 173.6, 172.1, 139.2, 138.4, 135.2, 134.9, 128.7 (2C), 127.4, 127.2, 83.1, 82.9, 75.1, 69.8, 53.9, 53.0, 50.41, 50.36, 26.54, 26.51, 25.5, 25.4, 25.3, 25.2, 24.5 (2C), 21.5 (broad), 19.6 (broad), 17.2, 13.6, -2.2, -2.4, -2.7, -3.2; $^{11}B$ NMR ($CDCl_3$, 128 MHz): δ 33.44; HRMS (APCI) m/z ($[M+H]^+$) calcd for $C_{24}H_{41}BNO_4Si$: 446.2897, found: 446.2904.

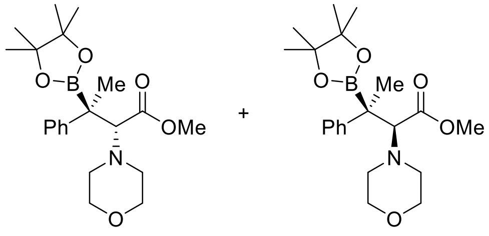

*anti*-**3Af** *syn*-**3Af**

**A 76:24 diastereomixture of Methyl (2*R**,3*S**)-2-morpholino-3-phenyl-3-(4,4,5,5-tetramethyl-1,3,2-dioxaborolan-2-yl)butanoate (*anti*-3Af) and Methyl (2*S**,3*S**)-2-morpholino-3-phenyl-3-(4,4,5,5-tetramethyl-1,3,2-dioxaborolan-2-yl)butanoate (*syn*-3Af) (The relative stereochemistry was tentatively assigned.)**

It was purified by silica gel column chromatography with hexane/ethyl acetate (5/1, v/v) and GPC ($CHCl_3$): 66.2 mg (68%, 0.25 mmol scale); colorless oil; $^1$H NMR ($CDCl_3$, 400 MHz): δ 7.48 (d, $J$ = 7.3 Hz, 0.76 × 2H for ***anti*-3Af**), 7.46 (d, $J$ = 7.3 Hz, 0.24 × 2H for ***syn*-3Af**), 7.28-7.22 [(m, 0.76 × 2H for ***anti*-3Af** and 0.24 × 2H for ***syn*-3Af**)], 7.14 (t, $J$ = 7.3 Hz, 0.24H for ***syn*-3Af**), 7.12 (t, $J$ = 7.3 Hz, 0.76H for ***anti*-3Af**), 3.77 (s, 0.24H for ***syn*-3Af**), 3.74 (s, 0.24 × 3H for ***syn*-3Af**), 3.71 (s, 0.76H for ***anti*-3Af**), 3.66-3.60 [(m, 0.76 × 3H for ***anti*-3Af** and 0.24 × 3H for ***syn*-3Af**)], 3.51-3.41 [(m, 0.76H for ***anti*-3Af** and 0.24H for ***syn*-3Af**)], 3.48 (s, 0.76 × 3H for ***anti*-3Af**), 2.67-2.61 [(m, 0.76 × 2H for ***anti*-3Af** and 0.24 × 2H for ***syn*-3Af**)], 2.48-2.45 (m, 0.76 × 2H for ***anti*-3Af**), 2.22-2.17 (m, 0.24 × 2H for ***syn*-3Af**), 1.59 (s, 0.76 × 3H for ***anti*-3Af**), 1.53 (s, 0.24 × 3H for ***syn*-3Af**), 1.21 [(s, 0.76 × 6H for ***anti*-3Af** and 0.24 × 6H for ***syn*-3Af**)], 1.19 (s, 0.76 × 6H for ***anti*-3Af**), 1.14 (s, 0.24 × 6H for ***syn*-3Af**); $^{13}$C{$^1$H} NMR ($CDCl_3$, 100 MHz): δ 172.9, 171.4, 144.1, 142.2, 128.4, 128.0, 127.7 (2C), 125.8, 125.5, 83.61, 83.58, 73.9 (2C), 67.63, 67.56, 53.1, 52.9, 51.0, 50.6, 35.0 (broad, 2C), 24.8, 24.63, 24.59, 24.55, 17.0, 15.7; $^{11}$B NMR ($CDCl_3$, 128 MHz): δ 32.78; HRMS (APCI) m/z ([M+H]$^+$) calcd for $C_{21}H_{33}BNO_5$: 390.2450, found: 390.2467.

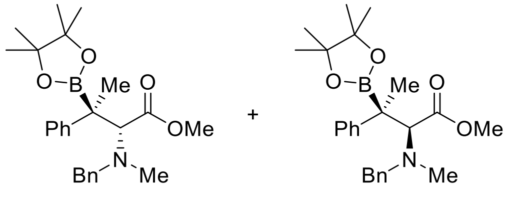

*anti*-**3Ac** *syn*-**3Ac**

**A 69:31 diastereomixture of Methyl (2*R**,3*S**)-2-(benzyl(methyl)amino)-3-phenyl-3-(4,4,5,5-**

**tetramethyl-1,3,2-dioxaborolan-2-yl)butanoate (*anti*-3Ac) and Methyl (2*S**,3*S**)-2-(benzyl(methyl)amino)-3-phenyl-3-(4,4,5,5-tetramethyl-1,3,2-dioxaborolan-2-yl)butanoate (*syn*-3Ac) (The relative stereochemistry was tentatively assigned.)**

It was purified by silica gel column chromatography with hexane/ethyl acetate (5/1, v/v) and GPC ($CHCl_3$): 69.9 mg (66%, 0.25 mmol scale); colorless oil; $^1$H NMR ($CDCl_3$, 400 MHz): δ 7.51-7.48 [(m, 0.69 × 2H for ***anti*-3Ac** and 0.31 × 2H for ***syn*-3Ac**)], 7.37 (d, *J* = 7.3 Hz, 0.69 × 2H for ***anti*-3Ac**), 7.32-7.10 [(m, 0.69 × 6H for ***anti*-3Ac** and 0.31 × 6H for ***syn*-3Ac**)], 6.91-6.89 (m, 0.31 × 2H for ***syn*-3Ac**), 4.25 (s, 0.31H for ***syn*-3Ac**), 4.01 (s, 0.69H for ***anti*-3Ac**), 3.89 (d, *J* = 13.8 Hz, 0.69H for ***anti*-3Ac**), 3.80 (s, 0.31 × 3H for ***syn*-3Ac**), 3.71 (d, *J* = 13.8 Hz, 0.31H for ***syn*-3Ac**), 3.54 (s, 0.69 × 3H for ***anti*-3Ac**), 3.48 (d, *J* = 13.8 Hz, 0.31H for ***syn*-3Ac**), 3.46 (d, *J* = 13.8 Hz, 0.69H for ***anti*-3Ac**), 2.15 (s, 0.69 × 3H for ***anti*-3Ac**), 2.09 (s, 0.31 × 3H for ***syn*-3Ac**), 1.67 (s, 0.69 × 3H for ***anti*-3Ac**), 1.58 (s, 0.31 × 3H for ***syn*-3Ac**), 1.23 (s, 0.69 × 6H for ***anti*-3Ac**), 1.20 (s, 0.69 × 6H for ***anti*-3Ac**), 1.19 (s, 0.31 × 6H for ***syn*-3Ac**), 1.12 (s, 0.31 × 6H for ***syn*-3Ac**); $^{13}$C{$^1$H} NMR ($CDCl_3$, 100 MHz): δ 174.0, 172.0, 144.1, 143.0, 140.32, 140.26, 128.5, 128.33, 128.29, 128.16, 128.0, 127.88, 127.87, 127.76, 126.9, 126.6, 125.6, 125.3, 83.6, 83.5, 73.6, 73.3, 62.2, 60.8, 51.1, 50.6, 41.7, 40.8, 35.7 (broad, 2C), 24.9, 24.7, 24.6, 24.5, 17.2, 15.7; $^{11}$B NMR ($CDCl_3$, 128 MHz): δ 32.62; HRMS (APCI) m/z ([M+H]$^+$) calcd for $C_{25}H_{35}BNO_4$: 424.2658, found: 424.2661.

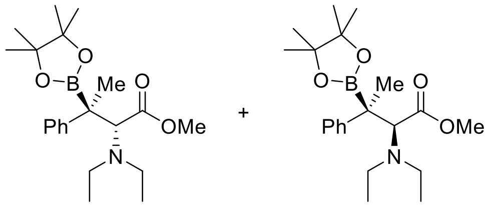

***anti*-3Ab** + ***syn*-3Ab**

**A 70:30 diastereomixture of Methyl (2*R**,3*S**)-2-(diethylamino)-3-phenyl-3-(4,4,5,5-tetramethyl-1,3,2-dioxaborolan-2-yl)butanoate (*anti*-3Ab) and Methyl (2*S**,3*S**)-2-(diethylamino)-3-phenyl-3-(4,4,5,5-tetramethyl-1,3,2-dioxaborolan-2-yl)butanoate (*syn*-3Ab) (The relative stereochemistry was tentatively assigned.)**

It was purified by silica gel column chromatography with hexane/ethyl acetate (5/1, v/v) and GPC ($CHCl_3$): 38.5 mg (41%, 0.25 mmol scale); colorless oil; $^1$H NMR ($CDCl_3$, 400 MHz): δ 7.51 (d, *J* = 7.3 Hz, 0.30 × 2H for ***syn*-3Ab**), 7.44 (d, *J* = 7.3 Hz, 0.70 × 2H for ***anti*-3Ab**), 7.25 (t, *J* = 7.3 Hz, 0.70 × 2H for ***anti*-3Ab**), 7.23 (t, *J* = 7.3 Hz, 0.30 × 2H for ***syn*-3Ab**), 7.12 [(t, *J* = 7.3 Hz, 0.70H for ***anti*-3Ab** and 0.30H for ***syn*-3Ab**)], 3.83 (s, 0.70H for ***anti*-3Ab**), 3.82 (s, 0.30H for ***syn*-3Ab**), 3.71 (s, 0.70 × 3H for

***anti*-3Ab**), 3.47 (s, 0.30 × 3H for ***syn*-3Ab**), 2.49-2.28 [(m, 0.70 × 4H for ***anti*-3Ab** and 0.30 × 4H for ***syn*-3Ab**)], 1.54 (s, 0.30 × 3H for ***syn*-3Ab**), 1.50 (s, 0.70 × 3H for ***anti*-3Ab**), 1.24 (s, 0.70 × 6H for ***anti*-3Ab**), 1.23 (s, 0.30 × 6H for ***syn*-3Ab**), 1.21 (s, 0.30 × 6H for ***syn*-3Ab**), 1.14 (s, 0.70 × 6H for ***anti*-3Ab**), 0.92 (t, *J* = 7.1 Hz, 0.30 × 6H for ***syn*-3Ab**), 0.63 (t, *J* = 7.1 Hz, 0.70 × 6H for ***anti*-3Ab**); $^{13}C\{^{1}H\}$ NMR ($CDCl_3$, 100 MHz): δ 174.6, 173.2, 144.7, 143.1, 129.0, 128.3, 127.6, 127.4, 125.6, 125.3, 83.45, 83.42, 71.2, 71.0, 50.9, 50.6, 47.4, 46.2, 34.9 (broad, 2C), 24.9, 24.73, 24.67, 24.5, 18.5, 15.2, 13.7, 13.5; $^{11}B$ NMR ($CDCl_3$, 128 MHz): δ 34.01; HRMS (APCI) m/z ($[M+H]^+$) calcd for $C_{21}H_{35}BNO_4$: 376.2657, found: 376.2666.

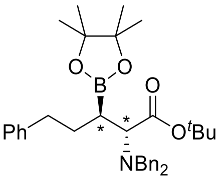

***anti*-3aa-O*^t*Bu**

***tert*-Butyl (2*R*,3*R*)-2-(dibenzylamino)-5-phenyl-3-(4,4,5,5-tetramethyl-1,3,2-dioxaborolan-2-yl)pentanoate (*anti*-3aa-O*^t*Bu, *anti*/*syn* = 95:5)**

It was purified by silica gel column chromatography with hexane/ethyl acetate (5/1, v/v) and GPC ($CHCl_3$): 102.7 mg (74%, 0.25 mmol scale), 405.6 mg (73%, 1.0 mmol scale); white solid; mp 82.9-83.9 °C; $^{1}H$ NMR ($CDCl_3$, 400 MHz): δ 7.29-7.13 (m, 15H), 3.85 (d, *J* = 13.5 Hz, 2H), 3.45 (d, *J* = 13.5 Hz, 2H), 3.29 (d, *J* = 11.3 Hz, 1H), 2.40-2.36 (m, 2H), 2.03-1.96 (m, 1H), 1.62-1.58 (m, 2H), 1.60 (s, 9H), 1.23 (s, 6H), 1.21 (s, 6H); $^{13}C\{^{1}H\}$ NMR ($CDCl_3$, 100 MHz): δ 173.0, 143.2, 139.9, 129.4, 128.6, 128.28, 128.25, 127.0, 125.6, 83.2, 81.2, 62.4, 54.7, 34.4, 29.2, 28.8, 25.1, 24.9, 23.7 (broad); $^{11}B$ NMR ($CDCl_3$, 128 MHz): δ 32.37; HRMS (APCI) m/z ($[M+H]^+$) calcd for $C_{35}H_{47}BNO_4$: 556.3599, found: 556.3618. CHIRALPAK AD-H column, 99/1 hexane/isopropyl alcohol, 0.5 mL/min, major isomer: $t_R$ = 11.5 min, minor isomer: $t_R$ = 18.8 min.

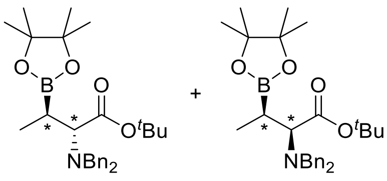

***anti*-3ba-O*^t*Bu** ***syn*-3ba-O*^t*Bu**

**An 88:12 diastereomixture of *tert*-Butyl (2*R*,3*R*)-2-(dibenzylamino)-3-(4,4,5,5-tetramethyl-1,3,2-dioxaborolan-2-yl)butanoate (*anti*-3ba-O*^t*Bu) and *tert*-Butyl (2*S*,3*R*)-2-(dibenzylamino)-3-(4,4,5,5-**

**tetramethyl-1,3,2-dioxaborolan-2-yl)butanoate (*syn*-3ba-O*t*Bu)**

It was purified by silica gel column chromatography with hexane/ethyl acetate (5/1, v/v) and GPC ($CHCl_3$): 91.9 mg (79%, 0.25 mmol scale); white solid; mp 139.2-140.2 °C; $^1$H NMR ($CDCl_3$, 400 MHz): δ 7.39 (d, *J* = 7.1 Hz, 0.12 × 4H for ***syn*-3ba-O*t*Bu**), 7.34 (d, *J* = 7.1 Hz, 0.88 × 4H for ***anti*-3ba-O*t*Bu**), 7.29 [(t, *J* = 7.1 Hz, 0.88 × 4H for ***anti*-3ba-O*t*Bu** and 0.12 × 4H for ***syn*-3ba-O*t*Bu**)], 7.23 (t, *J* = 7.1 Hz, 0.12 × 2H for ***syn*-3ba-O*t*Bu**), 7.21 (t, *J* = 7.1 Hz, 0.88 × 2H for ***anti*-3ba-O*t*Bu**), 4.01 (d, *J* = 13.8 Hz, 0.12 × 2H for ***syn*-3ba-O*t*Bu**), 3.89 (d, *J* = 13.6 Hz, 0.88 × 2H for ***anti*-3ba-O*t*Bu**), 3.48 (d, *J* = 13.6 Hz, 0.88 × 2H for ***anti*-3ba-O*t*Bu**), 3.37 (d, *J* = 13.8 Hz, 0.12 × 2H for ***syn*-3ba-O*t*Bu**), 3.35 (d, *J* = 11.8 Hz, 0.12H for ***syn*-3ba-O*t*Bu**), 3.13 (d, *J* = 11.7 Hz, 0.88H for ***anti*-3ba-O*t*Bu**), 1.58 (s, 0.88 × 9H for ***anti*-3ba-O*t*Bu**),1.54-1.46 [(m, 0.88H for ***anti*-3ba-O*t*Bu** and 0.12H for ***syn*-3ba-O*t*Bu**)], 1.53 (s, 0.12 × 9H for ***syn*-3ba-O*t*Bu**), 1.26 (s, 0.12 × 6H for ***syn*-3ba-O*t*Bu**), 1.24 (s, 0.12 × 6H for ***syn*-3ba-O*t*Bu**), 1.18 (s, 0.88 × 6H for ***anti*-3ba-O*t*Bu**), 1.15 (s, 0.88 × 6H for ***anti*-3ba-O*t*Bu**), 0.95 (d, *J* = 7.4 Hz, 0.88 × 3H for ***anti*-3ba-O*t*Bu**), 0.88 (d, *J* = 7.2 Hz, 0.12 × 3H for ***syn*-3ba-O*t*Bu**); $^{13}$C{$^1$H} NMR ($CDCl_3$, 100 MHz): δ 173.0, 171.6, 140.1, 139.8, 129.5, 129.3, 128.2, 128.0, 126.9 (2C), 83.2, 83.0, 81.0, 80.9, 65.4, 64.3, 55.3, 54.6, 28.8, 28.7, 25.2, 25.1, 24.8, 24.6, 17.4 (broad, 2C), 12.8, 12.6; $^{11}$B NMR ($CDCl_3$, 128 MHz): δ 32.61; HRMS (APCI) m/z ([M+H]$^+$) calcd for $C_{28}H_{41}BNO_4$: 466.3128, found: 466.3123. CHIRALPAK AD-H column, 99.4/0.6 hexane/isopropyl alcohol, 0.5 mL/min, major isomer: $t_R$ = 12.0 min, minor isomer: $t_R$ = 24.7 min.

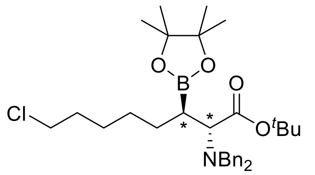


***anti*-3ea-O*t*Bu**

***tert*-Butyl (2*R*,3*R*)-8-chloro-2-(dibenzylamino)-3-(4,4,5,5-tetramethyl-1,3,2-dioxaborolan-2-yl)octanoate (*anti*-3ea-O*t*Bu, *anti*/*syn* = 95:5)**

It was purified by silica gel column chromatography with hexane/ethyl acetate (5/1, v/v) and GPC ($CHCl_3$): 104.2 mg (75%, 0.25 mmol scale); white solid; mp 97.4-98.4 °C; $^1$H NMR ($CDCl_3$, 400 MHz): δ 7.34-7.28 (m, 8H), 7.25-7.20 (m, 2H), 3.88 (d, *J* = 13.5 Hz, 2H), 3.48 (t, *J* = 6.8 Hz, 2H), 3.46 (d, *J* = 13.5 Hz, 2H), 3.22 (d, *J* = 11.8 Hz, 1H), 1.69-1.61 (m, 3H), 1.59 (s, 9H), 1.51-1.46 (m, 1H), 1.36-1.28 (m, 3H), 1.19 (s, 6H), 1.16 (s, 6H), 1.05-0.88 (m, 2H); $^{13}$C{$^1$H} NMR ($CDCl_3$, 100 MHz): δ 172.9, 139.9, 129.3, 128.2, 127.0, 83.0, 81.0, 61.8, 54.5, 45.2, 32.6, 28.8, 27.5, 27.2, 27.0, 24.9, 24.8, 23.6 (broad); $^{11}$B NMR ($CDCl_3$, 128 MHz): δ 32.20; HRMS (APCI) m/z ([M+H]$^+$) calcd for $C_{32}H_{48}BClNO_4$: 556.3365,

found: 556.3384. CHIRALPAK AD-H column, 99.2/0.8 hexane/isopropyl alcohol, 0.5 mL/min, major isomer: $t_R$ = 10.8 min, minor isomer: $t_R$ = 19.8 min.

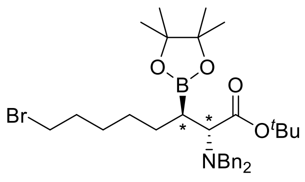

***anti*-3fa-O*t*Bu**

***tert*-Butyl (2*R*,3*R*)-8-bromo-2-(dibenzylamino)-3-(4,4,5,5-tetramethyl-1,3,2-dioxaborolan-2-yl)octanoate (*anti*-3fa-O*t*Bu, *anti*/*syn* = 95:5)**

It was purified by silica gel column chromatography with hexane/ethyl acetate (5/1, v/v) and GPC ($CHCl_3$): 100.6 mg (67%, 0.25 mmol scale); white solid; mp 100.7-101.7 °C; $^1$H NMR ($CDCl_3$, 400 MHz): δ 7.34-7.28 (m, 8H), 7.25-7.20 (m, 2H), 3.88 (d, $J$ = 13.5 Hz, 2H), 3.46 (d, $J$ = 13.5 Hz, 2H), 3.36 (t, $J$ = 6.9 Hz, 2H), 3.22 (d, $J$ = 11.8 Hz, 1H), 1.79-1.62 (m, 3H), 1.59 (s, 9H), 1.52-1.46 (m, 1H), 1.36-1.26 (m, 3H), 1.19 (s, 6H), 1.16 (s, 6H), 1.06-0.84 (m, 2H); $^{13}$C{$^1$H} NMR ($CDCl_3$, 100 MHz): δ 172.9, 139.9, 129.3, 128.2, 127.0, 83.0, 81.0, 61.8, 54.5, 34.1, 32.8, 28.79, 28.77, 27.1, 27.0, 24.9, 24.8, 23.6 (broad); $^{11}$B NMR ($CDCl_3$, 128 MHz): δ 32.52; HRMS (APCI) m/z ([M+H]$^+$) calcd for $C_{32}H_{48}BBrNO_4$: 600.2860, found: 600.2885. CHIRALPAK AD-H column, 99.2/0.8 hexane/isopropyl alcohol, 0.5 mL/min, major isomer: $t_R$ = 11.3 min, minor isomer: $t_R$ = 20.6 min.

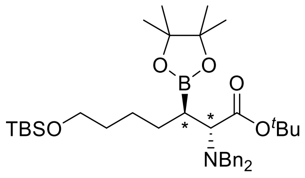

***anti*-3ha-O*t*Bu**

***tert*-Butyl (2*R*,3*R*)-7-((*tert*-butyldimethylsilyl)oxy)-2-(dibenzylamino)-3-(4,4,5,5-tetramethyl-1,3,2-dioxaborolan-2-yl)heptanoate (*anti*-3ha-O*t*Bu, *anti*/*syn* = 95:5)**

It was purified by silica gel column chromatography with hexane/ethyl acetate (5/1, v/v) and GPC ($CHCl_3$): 103.6 mg (65%, 0.25 mmol scale); colorless oil; $^1$H NMR ($CDCl_3$, 400 MHz): δ 7.33-7.26 (m, 8H), 7.21 (t, $J$ = 6.9 Hz, 2H), 3.88 (d, $J$ = 13.5 Hz, 2H), 3.51-3.47 (m, 2H), 3.45 (d, $J$ = 13.5 Hz, 2H), 3.22 (d, $J$ = 11.8 Hz, 1H), 1.72-1.63 (m, 1H), 1.59 (s, 9H), 1.51-1.45 (m, 2H), 1.41-1.28 (m, 2H), 1.18 (s, 6H), 1.16 (s, 6H), 1.08-0.95 (m, 2H), 0.89 (s, 9H), 0.04 (s, 6H); $^{13}$C{$^1$H} NMR ($CDCl_3$, 100 MHz): δ 172.9, 140.0, 129.3, 128.2, 126.9, 83.0, 81.0, 63.5, 62.0, 54.5, 33.8, 28.8, 27.3, 26.1, 24.9, 24.8, 24.3, 23.7 (broad), 18.5, -5.1; $^{11}$B NMR ($CDCl_3$, 128 MHz): δ 30.84; HRMS (APCI) m/z ([M+H]$^+$) calcd for

$C_{37}H_{61}BNO_5Si$: 638.4413, found: 638.4431. CHIRALPAK AD-H column, 99.4/0.6 hexane/isopropyl alcohol, 0.5 mL/min, major isomer: $t_R$ = 8.9 min, minor isomer: $t_R$ = 12.4 min.

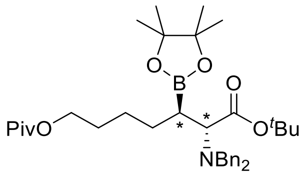


***anti*-3ia-O*t*Bu**

***tert*-Butyl (2*R*,3*R*)-2-(dibenzylamino)-7-(pivaloyloxy)-3-(4,4,5,5-tetramethyl-1,3,2-dioxaborolan-2-yl)heptanoate (*anti*-3ia-O*t*Bu, *anti*/*syn* = 95:5)**

It was purified by silica gel column chromatography with hexane/ethyl acetate (5/1, v/v) and GPC ($CHCl_3$): 115.5 mg (76%, 0.25 mmol scale); white solid; mp 72.9-73.9 °C; $^1$H NMR ($CDCl_3$, 400 MHz): δ 7.33-7.27 (m, 8H), 7.24-7.20 (m, 2H), 3.96-3.86 (m, 2H), 3.88 (d, $J$ = 13.4 Hz, 2H), 3.46 (d, $J$ = 13.4 Hz, 2H), 3.23 (d, $J$ = 11.8 Hz, 1H), 1.72-1.64 (m, 1H), 1.59 (s, 9H), 1.51-1.29 (m, 4H), 1.19 (s, 6H), 1.18 (s, 9H), 1.16 (s, 6H), 1.12-0.88 (m, 2H); $^{13}$C{$^1$H} NMR ($CDCl_3$, 100 MHz): δ 178.6, 172.8, 139.9, 129.3, 128.2, 127.0, 83.0, 81.0, 64.6, 61.9, 54.5, 38.7, 29.4, 28.7, 27.3, 27.0, 24.9, 24.8, 24.4, 23.6 (broad); $^{11}$B NMR ($CDCl_3$, 128 MHz): δ 32.37; HRMS (APCI) m/z ($[M+H]^+$) calcd for $C_{36}H_{55}BNO_6$: 608.4123, found: 608.4124. CHIRALPAK AD-H column, 99/1 hexane/isopropyl alcohol, 0.5 mL/min, major isomer: $t_R$ = 11.0 min, minor isomer: $t_R$ = 21.5 min.

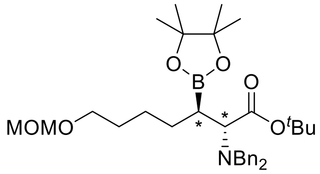


***anti*-3ja-O*t*Bu**

***tert*-Butyl (2*R*,3*R*)-2-(dibenzylamino)-7-(methoxymethoxy)-3-(4,4,5,5-tetramethyl-1,3,2-dioxaborolan-2-yl)heptanoate (*anti*-3ja-O*t*Bu, *anti*/*syn* = 95:5)**

It was purified by silica gel column chromatography with hexane/ethyl acetate (5/1, v/v) and GPC ($CHCl_3$): 119.2 mg (84%, 0.25 mmol scale); colorless oil; $^1$H NMR ($CDCl_3$, 400 MHz): δ 7.34-7.27 (m, 8H), 7.22 (t, $J$ = 6.8 Hz, 2H), 4.60 (s, 2H), 3.88 (d, $J$ = 13.4 Hz, 2H), 3.46 (d, $J$ = 13.4 Hz, 2H), 3.40 (t, $J$ = 7.0 Hz, 2H), 3.35 (s, 3H), 3.23 (d, $J$ = 11.8 Hz, 1H), 1.75-1.65 (m, 1H), 1.59 (s, 9H), 1.52-1.44 (m, 3H), 1.36-1.30 (m, 1H), 1.19 (s, 6H), 1.16 (s, 6H), 1.10-0.97 (m, 2H); $^{13}$C{$^1$H} NMR ($CDCl_3$, 100 MHz): δ 172.9, 139.9, 129.3, 128.2, 126.9, 96.4, 83.0, 81.0, 67.8, 62.0, 55.1, 54.5, 30.3, 28.7, 27.1, 24.9, 24.8, 24.5, 23.6 (broad); $^{11}$B NMR ($CDCl_3$, 128 MHz): δ 32.21; HRMS (APCI) m/z ($[M+H]^+$) calcd for

$C_{33}H_{51}BNO_6$: 568.3810, found: 568.3815. CHIRALPAK AD-H column, 99/1 hexane/isopropyl alcohol, 0.5 mL/min, major isomer: $t_R$ = 13.7 min, minor isomer: $t_R$ = 43.0 min.

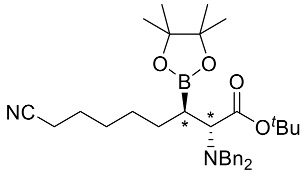


***anti*-3ka-O*t*Bu**

***tert*-Butyl (2*R*,3*R*)-8-cyano-2-(dibenzylamino)-3-(4,4,5,5-tetramethyl-1,3,2-dioxaborolan-2-yl)octanoate (*anti*-3ka-O*t*Bu, *anti*/*syn* = 95:5)**

It was purified by silica gel column chromatography with hexane/ethyl acetate (5/1, v/v) and GPC ($CHCl_3$): 95.6 mg (70%, 0.25 mmol scale); white solid; mp 86.7-87.7 °C; $^1$H NMR ($CDCl_3$, 400 MHz): δ 7.33-7.28 (m, 8H), 7.25-7.21 (m, 2H), 3.87 (d, $J$ = 13.4 Hz, 2H), 3.46 (d, $J$ = 13.4 Hz, 2H), 3.23 (d, $J$ = 11.8 Hz, 1H), 2.26 (t, $J$ = 7.2 Hz, 2H), 1.67-1.58 (m, 1H), 1.60 (s, 9H), 1.54-1.45 (m, 3H), 1.37-1.26 (m, 3H), 1.19 (s, 6H), 1.16 (s, 6H), 1.03-0.83 (m, 2H); $^{13}$C{$^1$H} NMR ($CDCl_3$, 100 MHz): δ 172.8, 139.9, 129.3, 128.2, 127.0, 120.0, 83.1, 81.1, 61.7, 54.5, 29.2, 28.8, 27.0, 26.8, 25.4, 24.9, 24.8, 23.5 (broad), 17.1; $^{11}$B NMR ($CDCl_3$, 128 MHz): δ 32.39; HRMS (APCI) m/z ([M+H]$^+$) calcd for $C_{33}H_{48}BN_2O_4$: 547.3707, found: 547.3718. CHIRALPAK AD-H column, 98.8/1.2 hexane/isopropyl alcohol, 0.5 mL/min, major isomer: $t_R$ = 16.3 min, minor isomer: $t_R$ = 37.7 min.

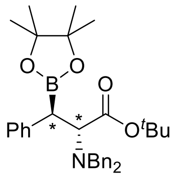


***anti*-3ma-O*t*Bu**

***tert*-Butyl (2*R*,3*R*)-2-(dibenzylamino)-3-phenyl-3-(4,4,5,5-tetramethyl-1,3,2-dioxaborolan-2-yl)propanoate (*anti*-3ma-O*t*Bu)**

It was purified by silica gel column chromatography with hexane/ethyl acetate (5/1, v/v) and GPC ($CHCl_3$): 163.6 mg (62%, 0.50 mmol scale); white solid; mp 123.8-124.8 °C; $^1$H NMR ($CDCl_3$, 400 MHz): δ 7.20-7.14 (m, 9H), 6.98-6.95 (m, 6H), 3.86 (d, $J$ = 13.8 Hz, 2H), 3.71 (d, $J$ = 12.2 Hz, 1H), 3.45 (d, $J$ = 13.8 Hz, 2H), 2.90 (d, $J$ = 12.2 Hz, 1H), 1.61 (s, 9H), 1.23 (s, 6H), 1.06 (s, 6H); $^{13}$C{$^1$H} NMR ($CDCl_3$, 100 MHz): δ 172.8, 139.6, 139.5, 130.0, 129.0, 128.10, 128.07, 126.7, 125.6, 83.4, 81.4, 63.9, 54.2, 32.9 (broad), 28.8, 24.7, 24.4; $^{11}$B NMR ($CDCl_3$, 128 MHz): δ 31.67; HRMS (APCI) m/z ([M+H]$^+$) calcd for $C_{33}H_{43}BNO_4$: 528.3285, found: 528.3267. CHIRALPAK AD-H column, 99.2/0.8

hexane/isopropyl alcohol, 0.5 mL/min, major isomer: $t_R$ = 14.1 min, minor isomer: $t_R$ = 36.4 min.

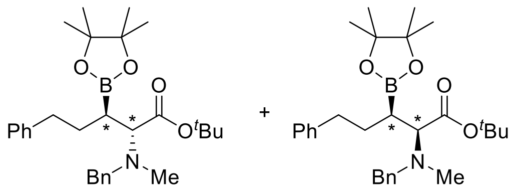


***anti*-3ac-O$^t$Bu** ***syn*-3ac-O$^t$Bu**

**An 80:20 diastereomixture of *tert*-Butyl (2*R*,3*R*)-2-(benzyl(methyl)amino)-5-phenyl-3-(4,4,5,5-tetramethyl-1,3,2-dioxaborolan-2-yl)pentanoate (*anti*-3ac-O$^t$Bu) and *tert*-Butyl (2*S*,3*R*)-2-(benzyl(methyl)amino)-5-phenyl-3-(4,4,5,5-tetramethyl-1,3,2-dioxaborolan-2-yl)pentanoate (*syn*-3ac-O$^t$Bu)**

It was purified by silica gel column chromatography with hexane/ethyl acetate (5/1, v/v) and GPC ($CHCl_3$): 100.7 mg (84%, 0.25 mmol scale); colorless oil; $^1$H NMR ($CDCl_3$, 400 MHz): δ 7.38 (d, *J* = 7.6 Hz, 0.20 × 2H for ***syn*-3ac-O$^t$Bu**), 7.31-7.15 [(m, 0.80 × 10H for ***anti*-3ac-O$^t$Bu** and 0.20 × 8H for ***syn*-3ac-O$^t$Bu**)], 3.78 (d, *J* = 13.7 Hz, 0.80H for ***anti*-3ac-O$^t$Bu**), 3.71 (d, *J* = 13.0 Hz, 0.20H for ***syn*-3ac-O$^t$Bu**), 3.63 (d, *J* = 13.7 Hz, 0.80H for ***anti*-3ac-O$^t$Bu**), 3.45 (d, *J* = 13.0 Hz, 0.20H for ***syn*-3ac-O$^t$Bu**), 3.38 (d, *J* = 12.0 Hz, 0.80H for ***anti*-3ac-O$^t$Bu**), 3.32 (d, *J* = 12.0 Hz, 0.20H for ***syn*-3ac-O$^t$Bu**), 2.74-2.49 [(m, 0.80 × 2H for ***anti*-3ac-O$^t$Bu** and 0.20 × 2H for ***syn*-3ac-O$^t$Bu**)], 2.24 (s, 0.20 × 3H for ***syn*-3ac-O$^t$Bu**), 2.14 (s, 0.80 × 3H for ***anti*-3ac-O$^t$Bu**), 2.12-2.03 (m, 0.80H for ***anti*-3ac-O$^t$Bu**), 1.91-1.84 (m, 0.20H for ***syn*-3ac-O$^t$Bu**), 1.82-1.69 [(m, 0.80H for ***anti*-3ac-O$^t$Bu** and 0.20H for ***syn*-3ac-O$^t$Bu**)], 1.60-1.53 [(m, 0.80H for ***anti*-3ac-O$^t$Bu** and 0.20H for ***syn*-3ac-O$^t$Bu**)], 1.53 (s, 0.80 × 9H for ***anti*-3ac-O$^t$Bu**), 1.46 (s, 0.20 × 9H for ***syn*-3ac-O$^t$Bu**), 1.30 (s, 0.20 × 6H for ***syn*-3ac-O$^t$Bu**), 1.28 (s, 0.20 × 6H for ***syn*-3ac-O$^t$Bu**), 1.28 (s, 0.80 × 6H for ***anti*-3ac-O$^t$Bu**), 1.26 (s, 0.80 × 6H for ***anti*-3ac-O$^t$Bu**); $^{13}$C{$^1$H} NMR ($CDCl_3$, 100 MHz): δ 172.8, 170.7, 143.2, 142.6, 140.3, 139.4, 129.6, 128.8, 128.5, 128.40, 128.36, 128.31, 128.2, 127.9, 126.9, 126.8, 125.8, 125.6, 83.3, 83.2, 80.9, 80.8, 69.2, 67.7, 59.5, 59.4, 38.4, 37.0, 36.0, 34.8, 30.9, 29.4, 28.7, 28.6, 25.1 (2C), 24.9, 24.8, 23.5 (broad, 2C); $^{11}$B NMR ($CDCl_3$, 128 MHz): δ 33.02; HRMS (APCI) m/z ($[M+H]^+$) calcd for $C_{29}H_{43}BNO_4$: 480.3285, found: 480.3270. CHIRALPAK AD-H column, 99.2/0.8 hexane/isopropyl alcohol, 0.5 mL/min, major isomers: $t_R$ = 12.3, 18.1 min, minor isomers: $t_R$ = 13.5, 15.7 min.

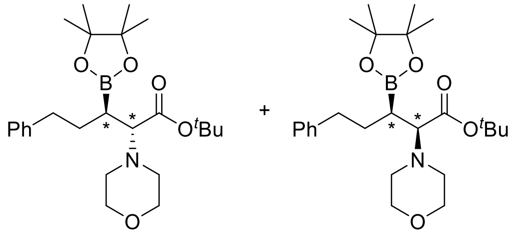

***anti*-3af-O$^t$Bu** ***syn*-3af-O$^t$Bu**

**An 85:15 diastereomixture of *tert*-Butyl (2*R*,3*R*)-2-morpholino-5-phenyl-3-(4,4,5,5-tetramethyl-1,3,2-dioxaborolan-2-yl)pentanoate (*anti*-3af-O$^t$Bu) and *tert*-Butyl (2*S*,3*R*)-2-morpholino-5-phenyl-3-(4,4,5,5-tetramethyl-1,3,2-dioxaborolan-2-yl)pentanoate (*syn*-3af-O$^t$Bu)**

It was purified by silica gel column chromatography with hexane/ethyl acetate (5/1, v/v) and GPC ($CHCl_3$): 98.0 mg (88%, 0.25 mmol scale); white solid; mp 154.8-155.8 °C; $^1$H NMR ($CDCl_3$, 400 MHz): δ 7.29-7.25 [(m, 0.85 × 2H for ***anti*-3af-O$^t$Bu** and 0.15 × 2H for ***syn*-3af-O$^t$Bu**)], 7.21-7.15 [(m, 0.85 × 3H for ***anti*-3af-O$^t$Bu** and 0.15 × 3H for ***syn*-3af-O$^t$Bu**)], 3.67-3.57 [(m, 0.85 × 4H for ***anti*-3af-O$^t$Bu** and 0.15 × 4H for ***syn*-3af-O$^t$Bu**)], 3.18 (d, *J* = 12.2 Hz, 0.85H for ***anti*-3af-O$^t$Bu**), 3.14 (d, *J* = 12.0 Hz, 0.15H for ***syn*-3af-O$^t$Bu**), 2.80-2.48 [(m, 0.85 × 4H for ***anti*-3af-O$^t$Bu** and 0.15 × 6H for ***syn*-3af-O$^t$Bu**)], 2.40-2.34 (m, 0.85 × 2H for ***anti*-3af-O$^t$Bu**), 2.02-1.94 (m, 0.85H for ***anti*-3af-O$^t$Bu**), 1.78-1.64 [(m, 0.85H for ***anti*-3af-O$^t$Bu** and 0.15 × 2H for ***syn*-3af-O$^t$Bu**)], 1.51-1.46 [(m, 0.85H for ***anti*-3af-O$^t$Bu** and 0.15H for ***syn*-3af-O$^t$Bu**)], 1.48 (s, 0.85 × 9H for ***anti*-3af-O$^t$Bu**), 1.43 (s, 0.15 × 9H for ***syn*-3af-O$^t$Bu**), 1.32 (s, 0.15 × 6H for ***syn*-3af-O$^t$Bu**), 1.31 (s, 0.15 × 6H for ***syn*-3af-O$^t$Bu**), 1.27 (s, 0.85 × 6H for ***anti*-3af-O$^t$Bu**), 1.25 (s, 0.85 × 6H for ***anti*-3af-O$^t$Bu**); $^{13}$C{$^1$H} NMR ($CDCl_3$, 100 MHz): δ 172.0, 170.0, 143.1, 142.4, 128.5, 128.40, 128.38, 128.32, 125.8, 125.7, 83.3, 83.2, 81.1 (2C), 70.9, 68.5, 67.8, 67.4, 50.3, 49.7, 35.9, 34.8, 30.6, 29.3, 28.6, 28.5, 25.4, 25.0 (2C), 24.8, 22.0 (broad, 2C); $^{11}$B NMR ($CDCl_3$, 128 MHz): δ 32.61; HRMS (APCI) m/z ([M+H]$^+$) calcd for $C_{25}H_{41}BNO_5$: 446.3077, found: 446.3067. CHIRALPAK AD-H column, 99.5/0.5 hexane/isopropyl alcohol, 0.5 mL/min, major isomers: $t_R$ = 30.7, 32.9 min, minor isomers: $t_R$ = 25.6, 28.9 min.

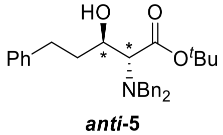

***anti*-5**

***tert*-Butyl (2*R*,3*R*)-2-(dibenzylamino)-3-hydroxy-5-phenylpentanoate (*anti*-5)**

It was purified by silica gel column chromatography with hexane/ethyl acetate (10/1 → 5/1, v/v) and GPC ($CHCl_3$): 43.5 mg (98%, 0.10 mmol scale); colorless oil; $^1$H NMR ($CDCl_3$, 400 MHz): δ 7.32-7.14

(m, 15H), 3.96-3.90 (m, 1H), 3.84 (d, $J$ = 13.4 Hz, 2H), 3.49 (d, $J$ = 13.4 Hz, 2H), 3.13 (d, $J$ = 9.0 Hz, 1H), 2.68-2.53 (m, 2H), 2.51 (d, $J$ = 4.4 Hz, 1H), 2.19-2.11 (m, 1H), 1.60 (s, 9H), 1.52-1.41 (m, 1H); $^{13}C\{^1H\}$ NMR ($CDCl_3$, 100 MHz): δ 172.1, 142.4, 139.0, 129.3, 128.7, 128.4 (2C), 127.3, 125.8, 82.2, 69.1, 65.8, 55.7, 34.7, 31.3, 28.7; HRMS (APCI) m/z ($[M+H]^+$) calcd for $C_{29}H_{36}NO_3$: 446.2690, found: 446.2686. CHIRALCEL OD-H column, 97/3 hexane/isopropyl alcohol, 0.5 mL/min, major isomer: $t_R$ = 13.5 min, minor isomer: $t_R$ = 14.9 min.

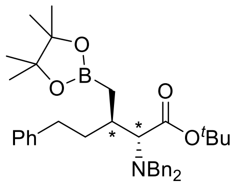

***anti*-6**

***tert*-Butyl (2*R*,3*R*)-2-(dibenzylamino)-5-phenyl-3-((4,4,5,5-tetramethyl-1,3,2-dioxaborolan-2-yl)methyl)pentanoate (*anti*-6)**

It was purified by silica gel column chromatography with hexane/ethyl acetate (10/1, v/v): 47.6 mg (84%, 0.10 mmol scale); white solid; mp 110.4-111.4 °C; $^1H$ NMR ($CDCl_3$, 400 MHz): δ 7.38 (d, $J$ = 7.2 Hz, 4H), 7.28 (t, $J$ = 7.2 Hz, 4H), 7.25-7.12 (m, 7H), 3.97 (d, $J$ = 13.8 Hz, 2H), 3.36 (d, $J$ = 13.8 Hz, 2H), 3.17 (d, $J$ = 11.1 Hz, 1H), 2.35 (t, $J$ = 8.2 Hz, 2H), 2.31-2.23 (m, 1H), 2.11-2.02 (m, 1H), 1.75-1.65 (m, 1H), 1.57 (s, 9H), 1.14 (s, 6H), 1.10 (s, 6H), 0.87 (dd, $J$ = 16.1, 3.8 Hz, 1H), 0.71 (dd, $J$ = 16.1, 8.1 Hz, 1H); $^{13}C\{^1H\}$ NMR ($CDCl_3$, 100 MHz): δ 171.5, 143.4, 139.9, 129.1, 128.5, 128.3, 128.3, 126.9, 125.5, 83.0, 81.0, 66.0, 54.7, 32.8, 32.5, 31.6, 28.7, 25.0, 24.7, 12.5 (broad); $^{11}B$ NMR ($CDCl_3$, 128 MHz): δ 33.05; HRMS (APCI) m/z ($[M+H]^+$) calcd for $C_{36}H_{49}BNO_4$: 570.3755, found: 570.3728. CHIRALCEL OD-H column, 99.9/0.1 hexane/isopropyl alcohol, 0.5 mL/min, major isomer: $t_R$ = 19.7 min, minor isomer: $t_R$ = 19.0 min.

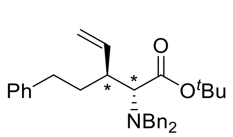

***anti*-7**

***tert*-Butyl (2*R*,3*R*)-2-(dibenzylamino)-3-phenethylpent-4-enoate (*anti*-7)**

It was purified by silica gel column chromatography with hexane/ethyl acetate (20/1, v/v): 42.2 mg (93%, 0.10 mmol scale); colorless oil; $^1H$ NMR ($CDCl_3$, 400 MHz): δ 7.36 (d, $J$ = 7.1 Hz, 4H), 7.30 (t, $J$ = 7.1 Hz, 4H), 7.25-7.20 (m, 4H), 7.16-7.09 (m, 3H), 5.46 (ddd, $J$ = 17.7, 13.6, 9.3 Hz, 1H), 5.10-5.05 (m, 2H),

3.94 (d, $J$ = 14.0 Hz, 2H), 3.38 (d, $J$ = 14.0 Hz, 2H), 3.05 (d, $J$ = 11.0 Hz, 1H), 2.62-2.54 (m, 2H), 2.41-2.30 (m, 2H), 1.50 (s, 9H), 1.39-1.31 (m, 1H); $^{13}C\{^1H\}$ NMR ($CDCl_3$, 100 MHz): δ 170.6, 142.6, 139.7, 138.7, 129.0, 128.6, 128.4 (2C), 127.1, 125.8, 118.2, 81.2, 65.2, 54.8, 43.2, 32.6, 31.9, 28.6; HRMS (APCI) m/z ($[M+H]^+$) calcd for $C_{31}H_{38}NO_2$: 456.2897, found: 456.2886. CHIRALPAK AD-H column, 99.6/0.4 hexane/isopropyl alcohol, 0.5 mL/min, major isomer: $t_R$ = 11.6 min, minor isomer: $t_R$ = 15.4 min.

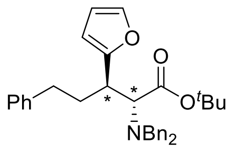


***anti*-8**

**_tert_-Butyl (2*R*,3*R*)-2-(dibenzylamino)-3-(furan-2-yl)-5-phenylpentanoate (*anti*-8)**

It was purified by silica gel column chromatography with hexane/ethyl acetate (40/1 → 20/1, v/v): 27.8 mg (56%, 0.10 mmol scale); colorless oil; $^1H$ NMR ($CDCl_3$, 400 MHz): δ 7.36 (d, $J$ = 7.1 Hz, 4H), 7.32 (t, $J$ = 7.1 Hz, 4H), 7.28-7.19 (m, 5H), 7.13 (t, $J$ = 7.3 Hz, 1H), 7.06 (d, $J$ = 7.3 Hz, 2H), 6.26 (dd, $J$ = 3.1, 1.9 Hz, 1H), 5.99 (d, $J$ = 3.1 Hz, 1H), 3.95 (d, $J$ = 13.9 Hz, 2H), 3.47 (d, $J$ = 11.4 Hz, 1H), 3.40 (d, $J$ = 13.9 Hz, 2H), 3.25 (td, $J$ = 11.4, 2.7 Hz, 1H), 2.49-2.38 (m, 2H), 2.30-2.21 (m, 1H), 1.79-1.69 (m, 1H), 1.34 (s, 9H); $^{13}C\{^1H\}$ NMR ($CDCl_3$, 100 MHz): δ 170.2, 155.2, 142.2, 141.3, 139.5, 129.1, 128.6, 128.41, 128.36, 127.1, 125.8, 110.0, 107.5, 81.0, 64.9, 54.7, 38.2, 32.8, 31.9, 28.3; HRMS (APCI) m/z ($[M+H]^+$) calcd for $C_{33}H_{38}NO_3$: 496.2846, found: 496.2858. CHIRALPAK AD-H column, 99.7/0.3 hexane/isopropyl alcohol, 0.5 mL/min, major isomer: $t_R$ = 19.5 min, minor isomer: $t_R$ = 28.4 min.

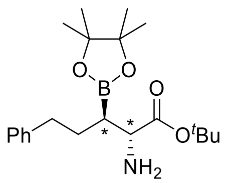


***anti*-9**

**_tert_-Butyl (2*R*,3*R*)-2-amino-5-phenyl-3-(4,4,5,5-tetramethyl-1,3,2-dioxaborolan-2-yl)pentanoate (*anti*-9)**

It was purified by filtration with celite: 37.0 mg (99%, 0.10 mmol scale); colorless oil; $^1H$ NMR ($CDCl_3$, 400 MHz): δ 7.28-7.24 (m, 2H), 7.17-7.14 (m, 3H), 3.51 (d, $J$ = 4.7 Hz, 1H), 2.75 (ddd, $J$ = 13.6, 9.8, 5.0 Hz, 1H), 2.56 (ddd, $J$ = 13.6, 9.5, 7.2 Hz, 1H), 1.92-1.83 (m, 1H), 1.60-1.43 (m, 2H), 1.58 (brs, 2H), 1.40

(s, 9H), 1.28 (s, 12H); $^{13}C\{^1H\}$ NMR ($CDCl_3$, 100 MHz): δ 175.2, 142.7, 128.6, 128.3, 125.7, 83.4, 80.8, 56.5, 35.6, 28.5, 28.1, 25.2, 24.8 (The carbon signal bound to boron was not observed due to quadrupolar relaxation.); $^{11}B$ NMR ($CDCl_3$, 128 MHz): δ 33.20; HRMS (APCI) m/z ($[M+H]^+$) calcd for $C_{21}H_{35}BNO_4$: 376.2657, found: 376.2662. CHIRALCEL OD-H column, 99.5/0.5 hexane/isopropyl alcohol, 0.5 mL/min, major isomer: $t_R$ = 19.1 min, minor isomer: $t_R$ = 19.8 min.

# NMR Spectra for Products

[$^{1}$H, $^{13}$C{$^{1}$H}, and $^{11}$B NMR Spectra of ***anti*-3aa**]

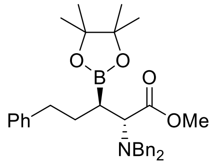


***anti*-3aa**

$^{1}$H NMR (400 MHz, $CDCl_3$)

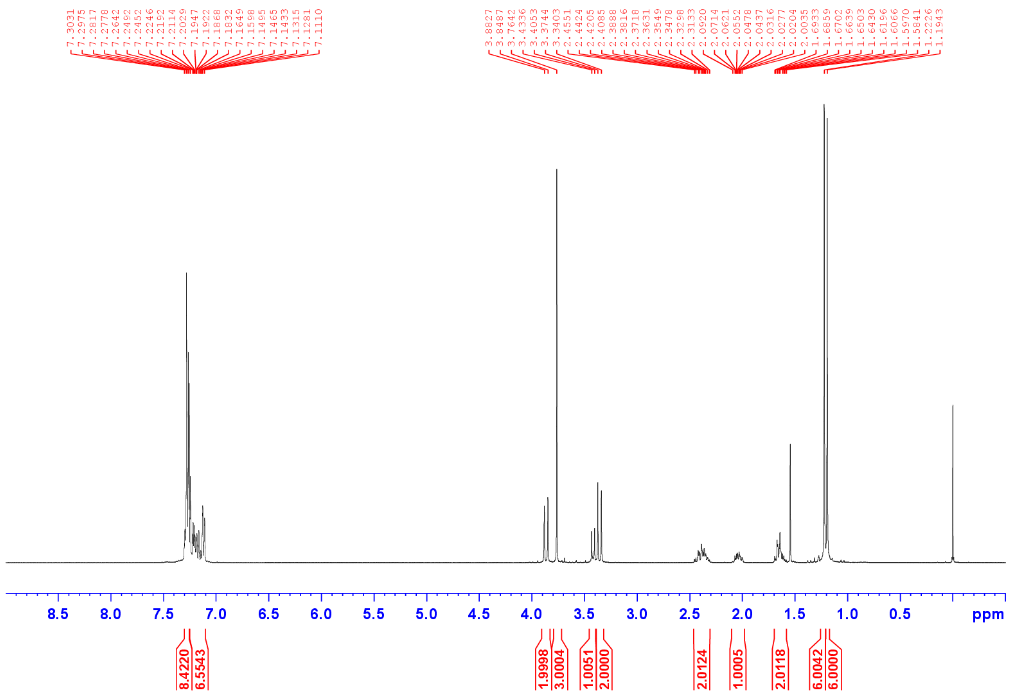

$^{13}$C{$^{1}$H} NMR (100 MHz, $CDCl_3$)

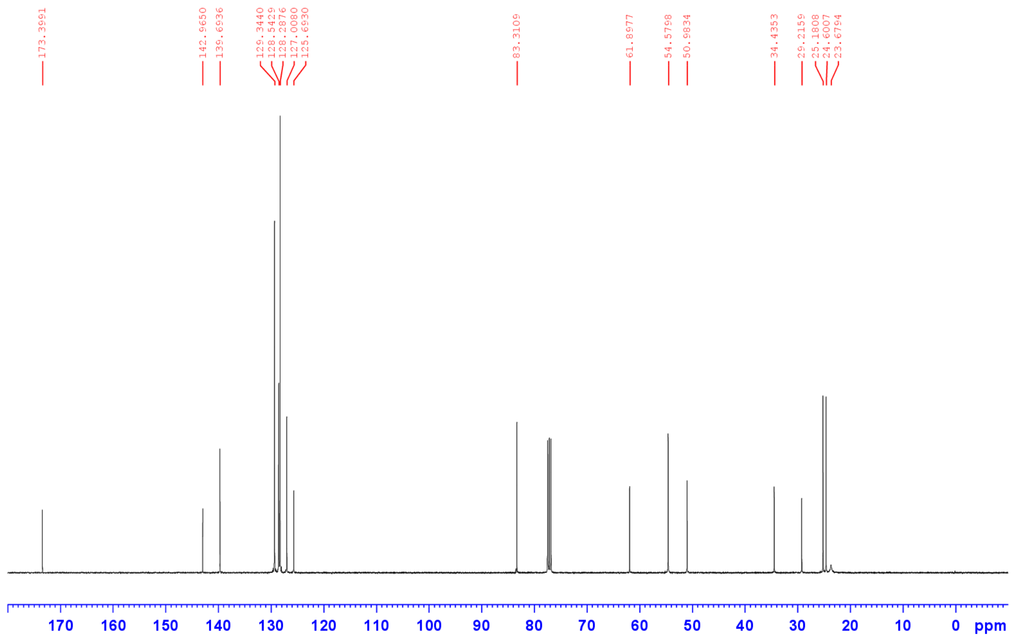

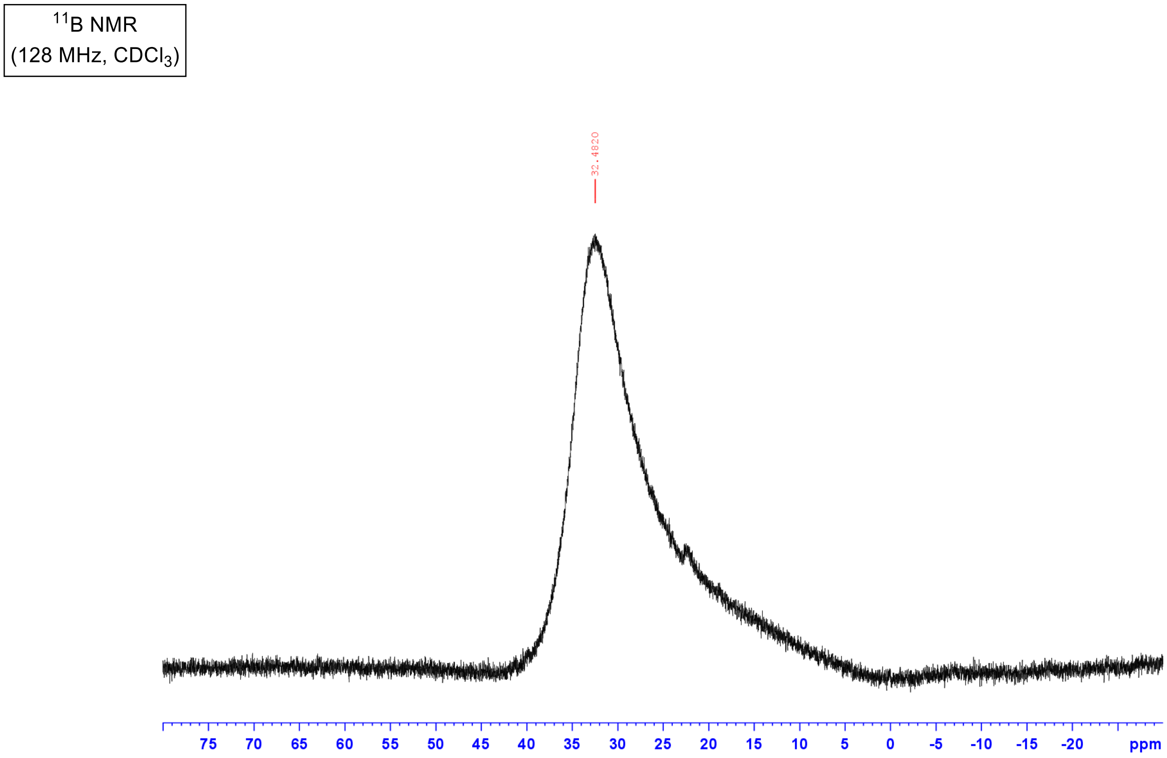

$^{11}$B NMR
(128 MHz, CDCl$_3$)
32.4820
75 70 65 60 55 50 45 40 35 30 25 20 15 10 5 0 -5 -10 -15 -20 ppm

[$^1$H, $^{13}$C{$^1$H}, and $^{11}$B NMR Spectra of ***anti*-3ba**]

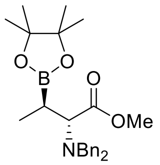


***anti*-3ba**

$^1$H NMR
(400 MHz, $CDCl_3$)

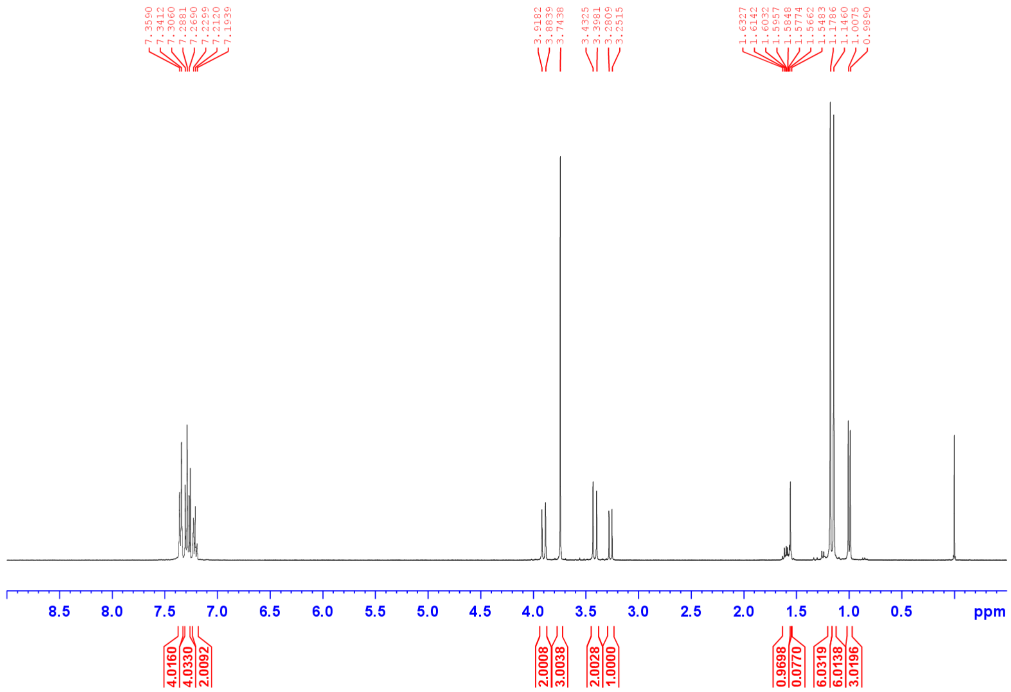


$^{13}$C{$^1$H} NMR
(100 MHz, $CDCl_3$)

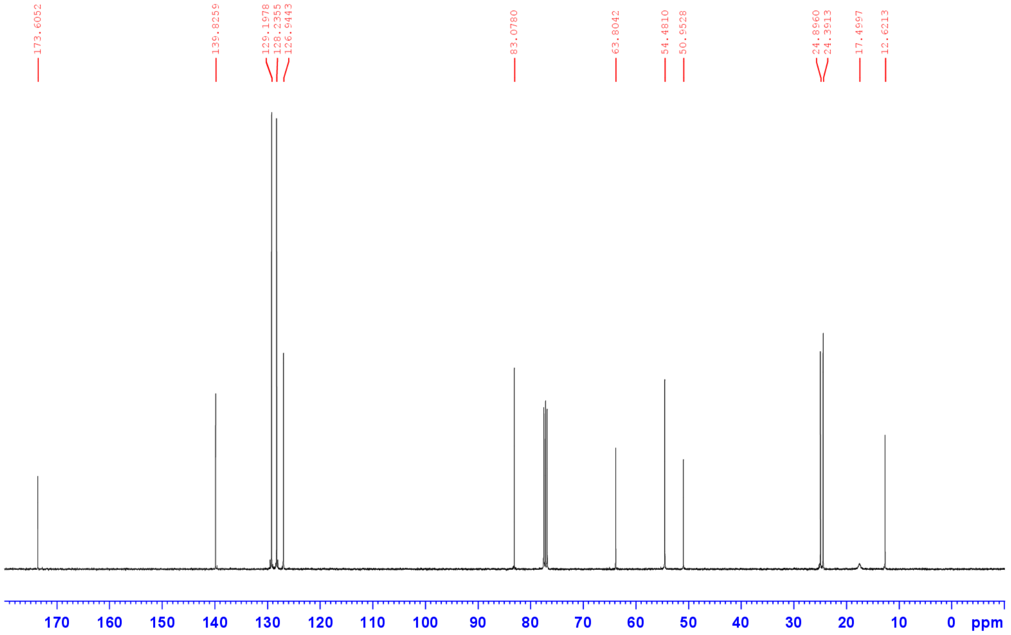

$^{11}$B NMR
(128 MHz, $CDCl_3$)

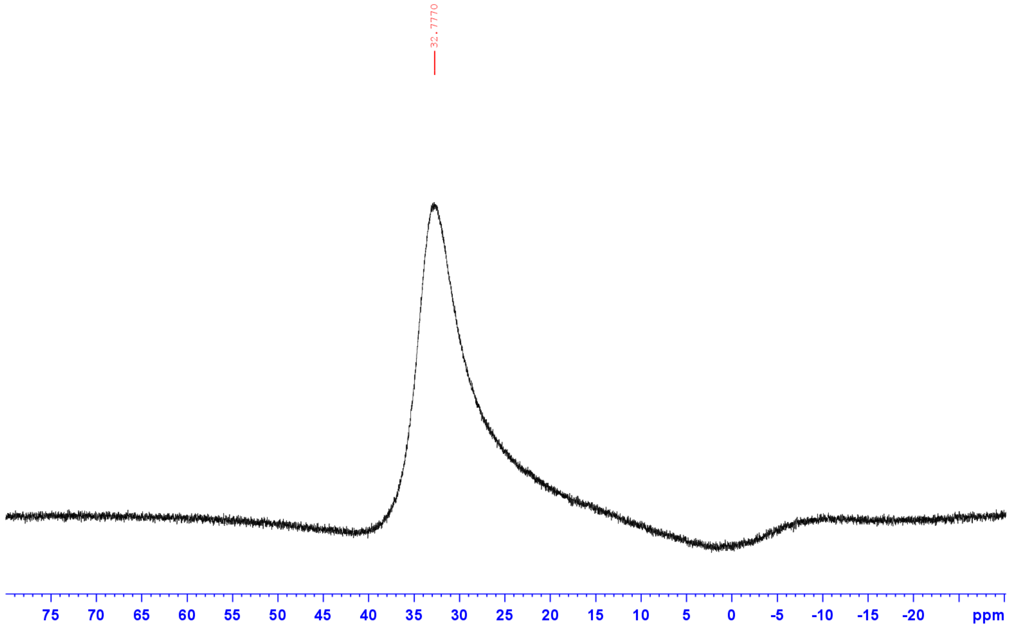

[$^{1}H$, $^{13}C\{^{1}H\}$, and $^{11}B$ NMR Spectra of ***anti*-3ca**]

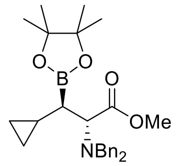


***anti*-3ca**

$^{1}H$ NMR
(400 MHz, $CDCl_3$)

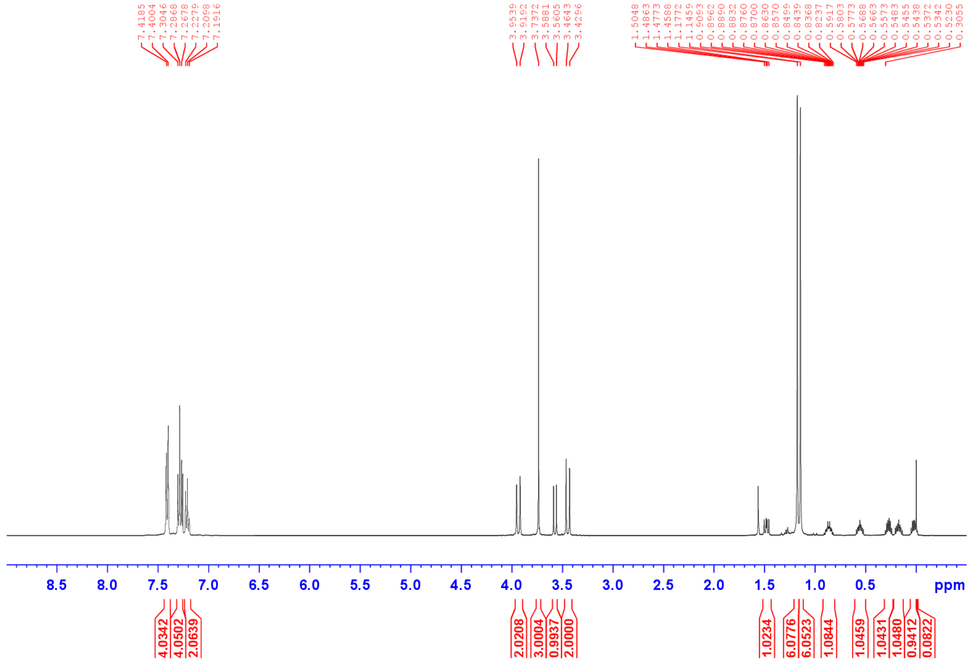

$^{13}C\{^{1}H\}$ NMR
(100 MHz, $CDCl_3$)

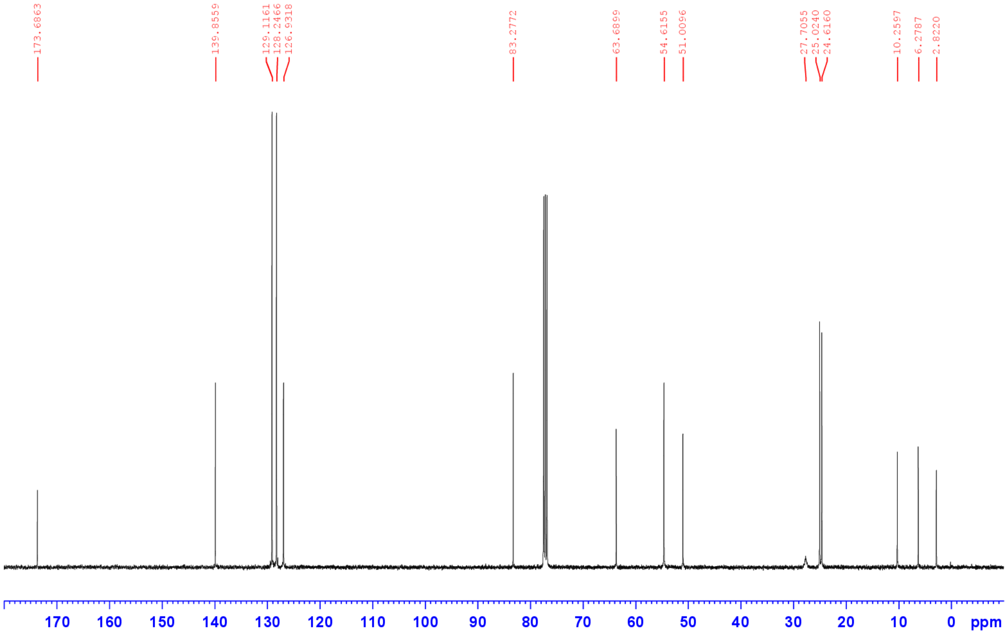

$^{11}B$ NMR
(128 MHz, $CDCl_3$)

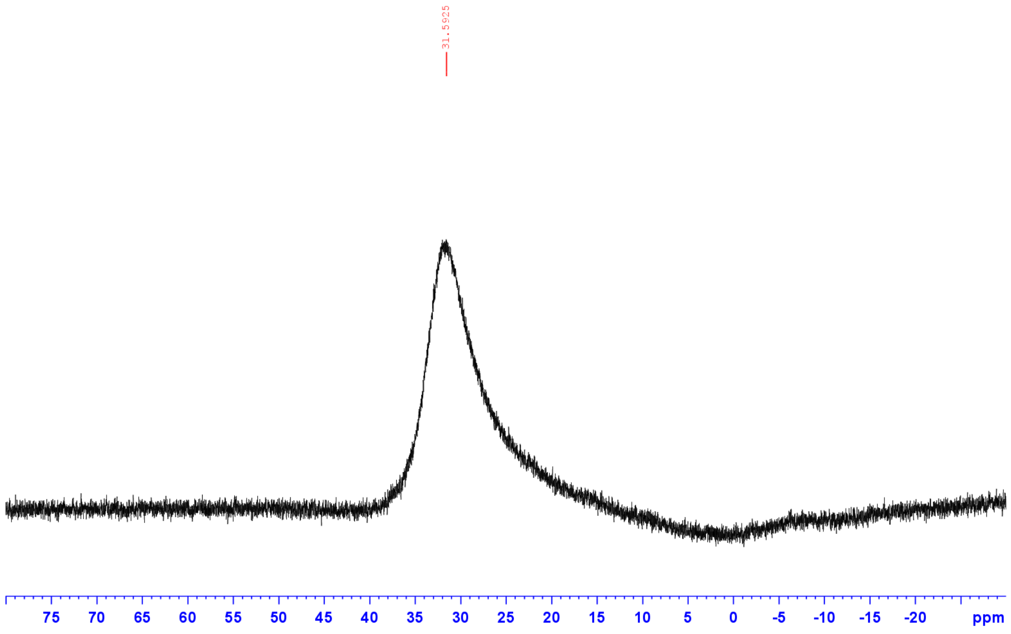
31.5925
75
70
65
60
55
50
45
40
35
30
25
20
15
10
5
0
-5
-10
-15
-20
ppm

[$^{1}H$, $^{13}C\{^{1}H\}$, and $^{11}B$ NMR Spectra of ***anti*-3da**]

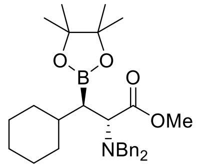


***anti*-3da**

$^{1}H$ NMR
(400 MHz, $CDCl_3$)

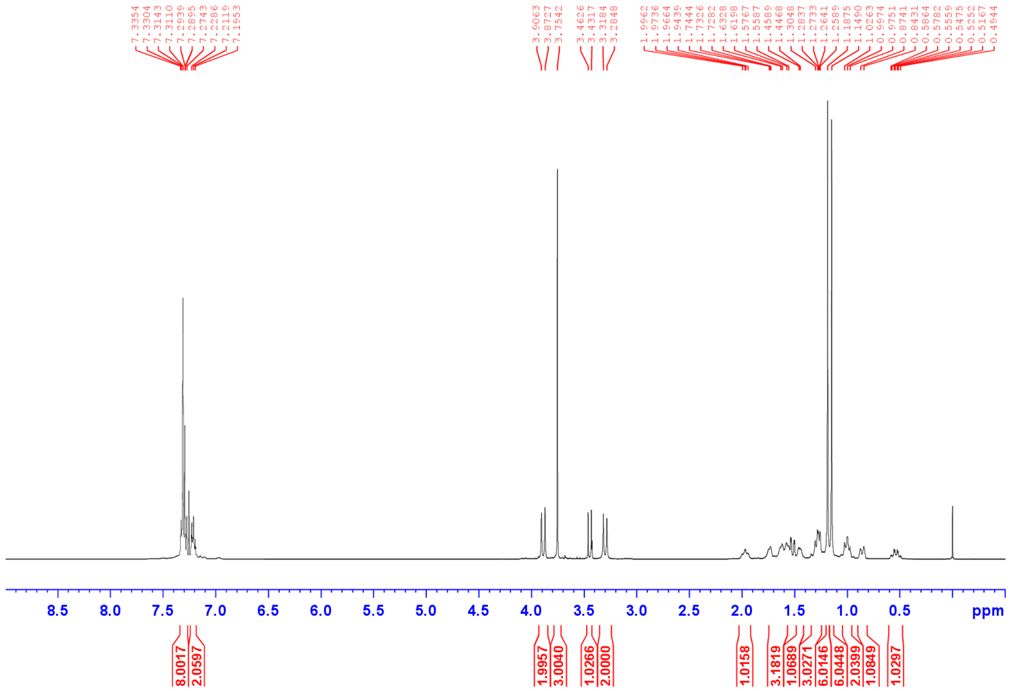


$^{13}C\{^{1}H\}$ NMR
(100 MHz, $CDCl_3$)

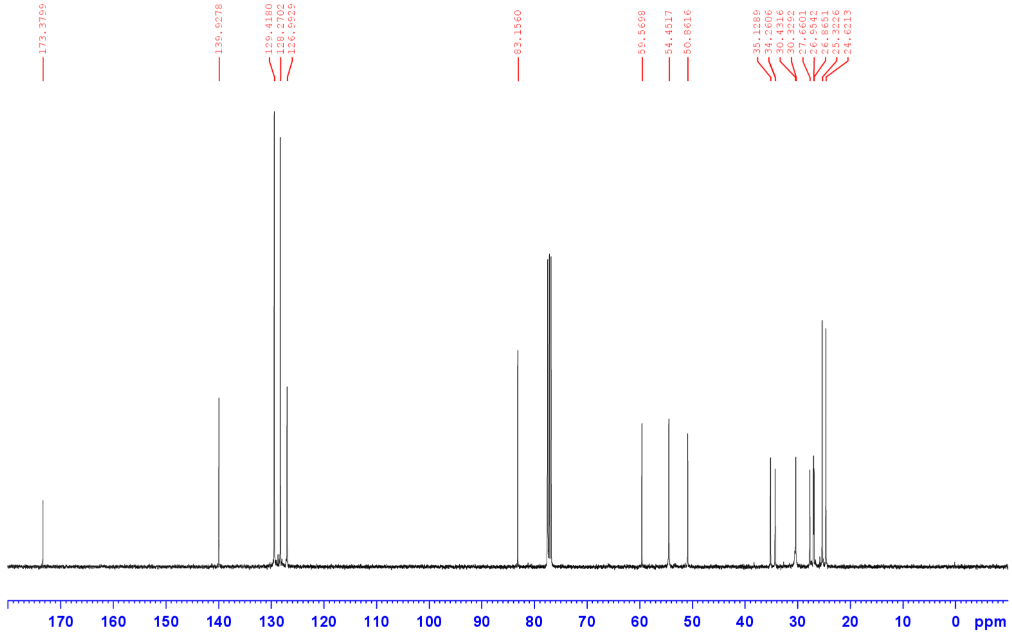

$^{11}B$ NMR
(128 MHz, $CDCl_3$)

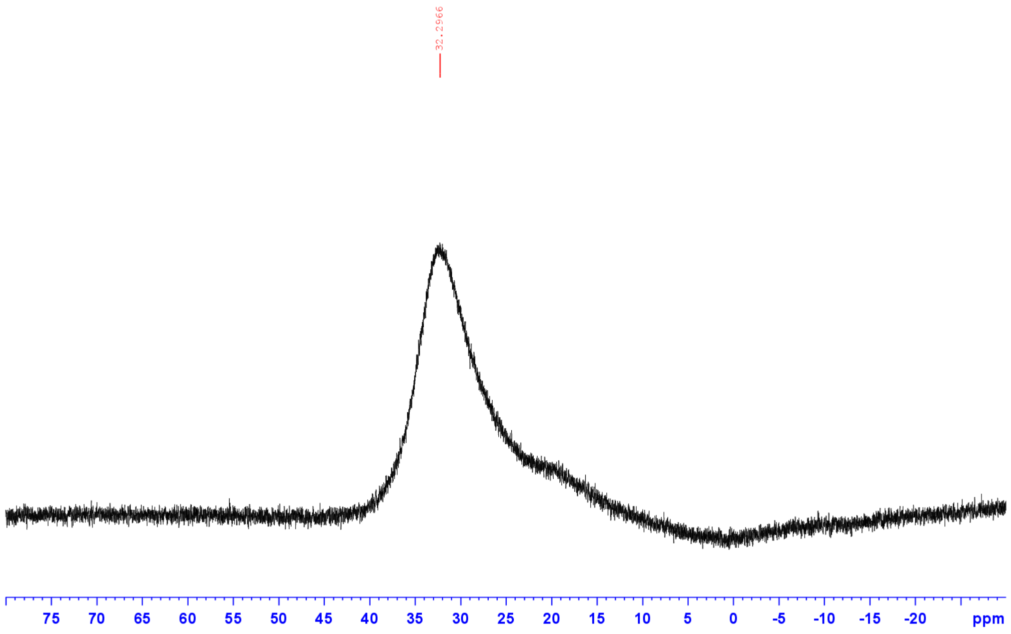

[$^{1}$H, $^{13}$C{$^{1}$H}, and $^{11}$B NMR Spectra of ***anti*-3ea**]

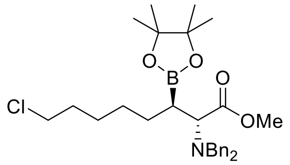

***anti*-3ea**

$^{1}$H NMR
(400 MHz, $CDCl_3$)

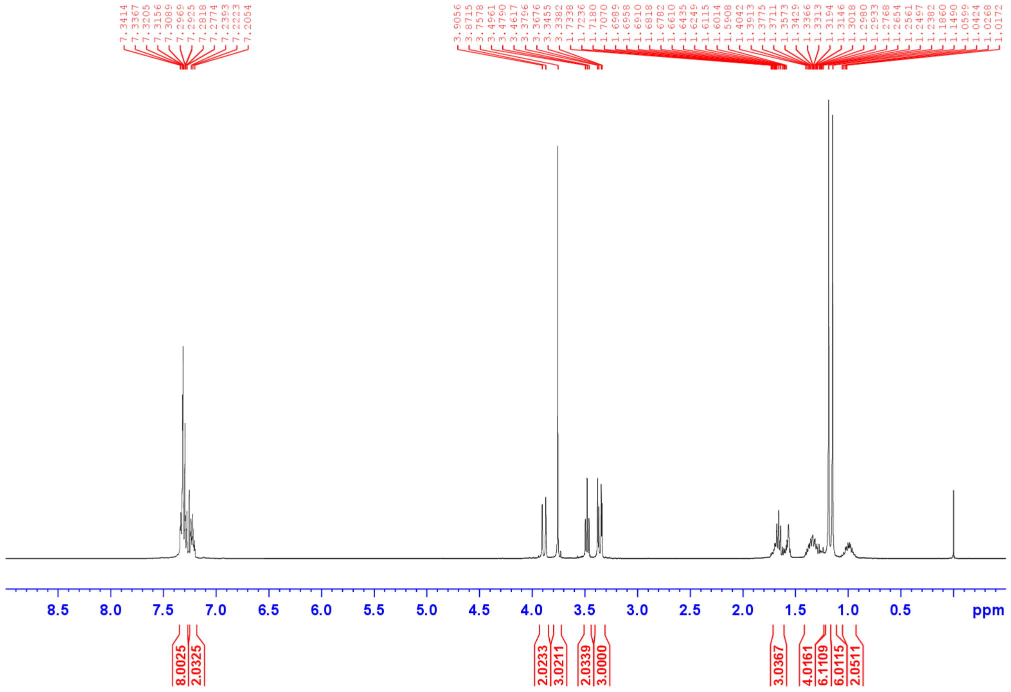


$^{13}$C{$^{1}$H} NMR
(100 MHz, $CDCl_3$)

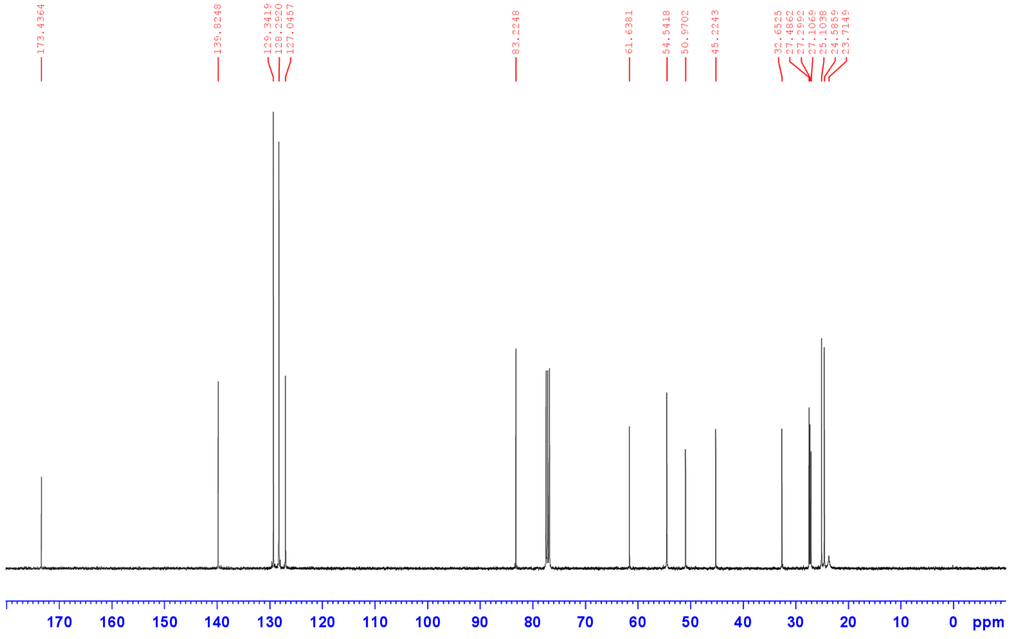

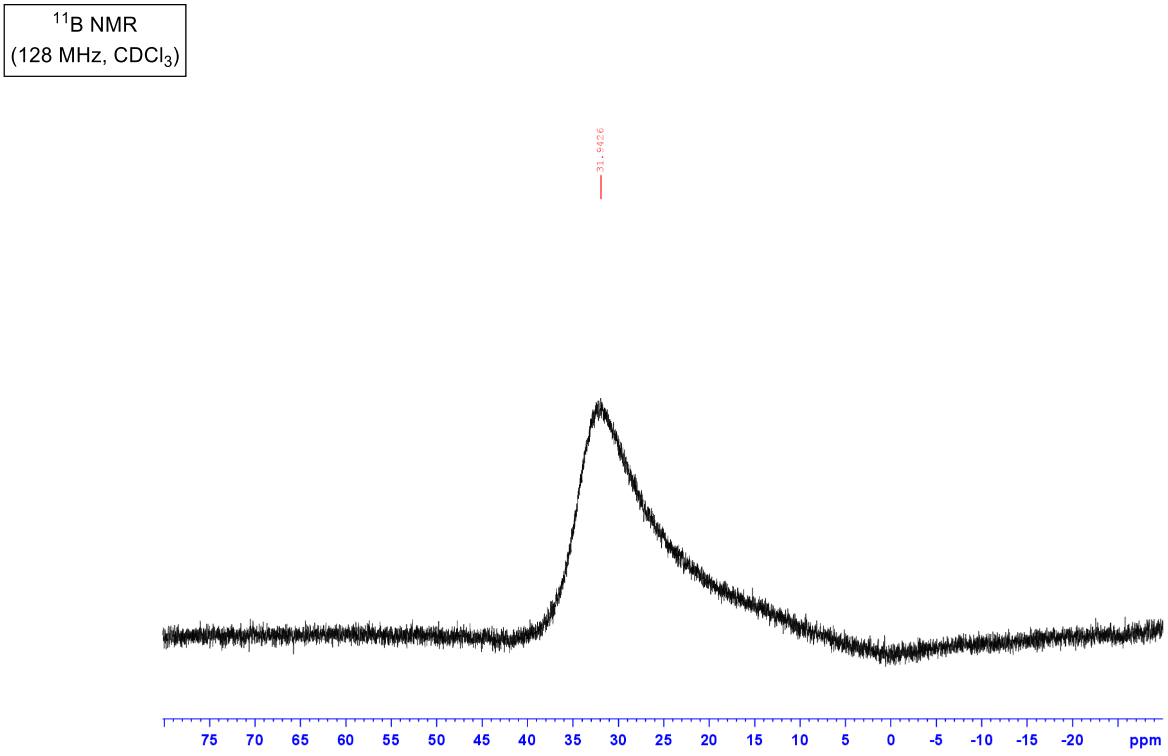

$^{11}B$ NMR
(128 MHz, $CDCl_3$)
31.9426
75 70 65 60 55 50 45 40 35 30 25 20 15 10 5 0 -5 -10 -15 -20 ppm

[$^{1}H$, $^{13}C\{^{1}H\}$, and $^{11}B$ NMR Spectra of ***anti*-3fa**]

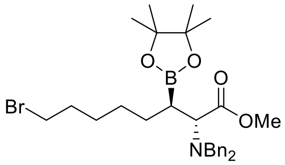


***anti*-3fa**

$^{1}H$ NMR
(400 MHz, $CDCl_3$)

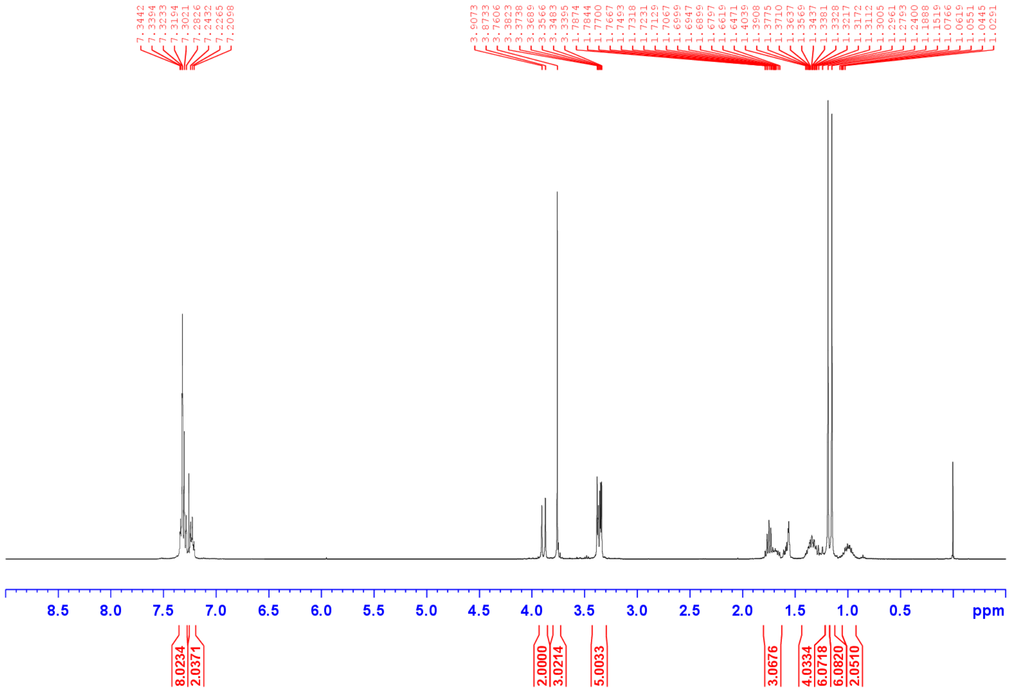


$^{13}C\{^{1}H\}$ NMR
(100 MHz, $CDCl_3$)

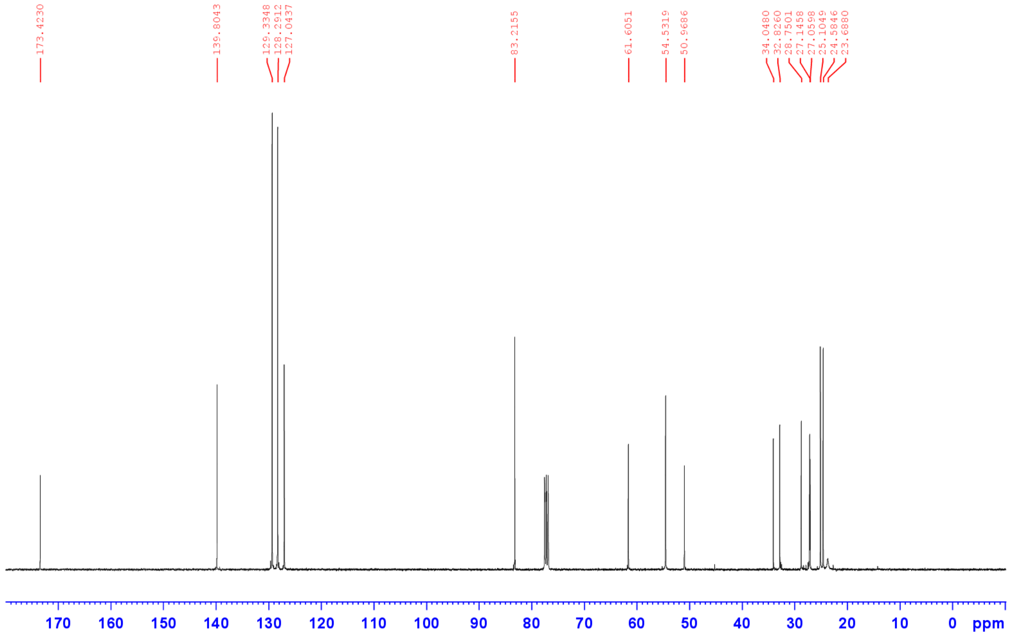

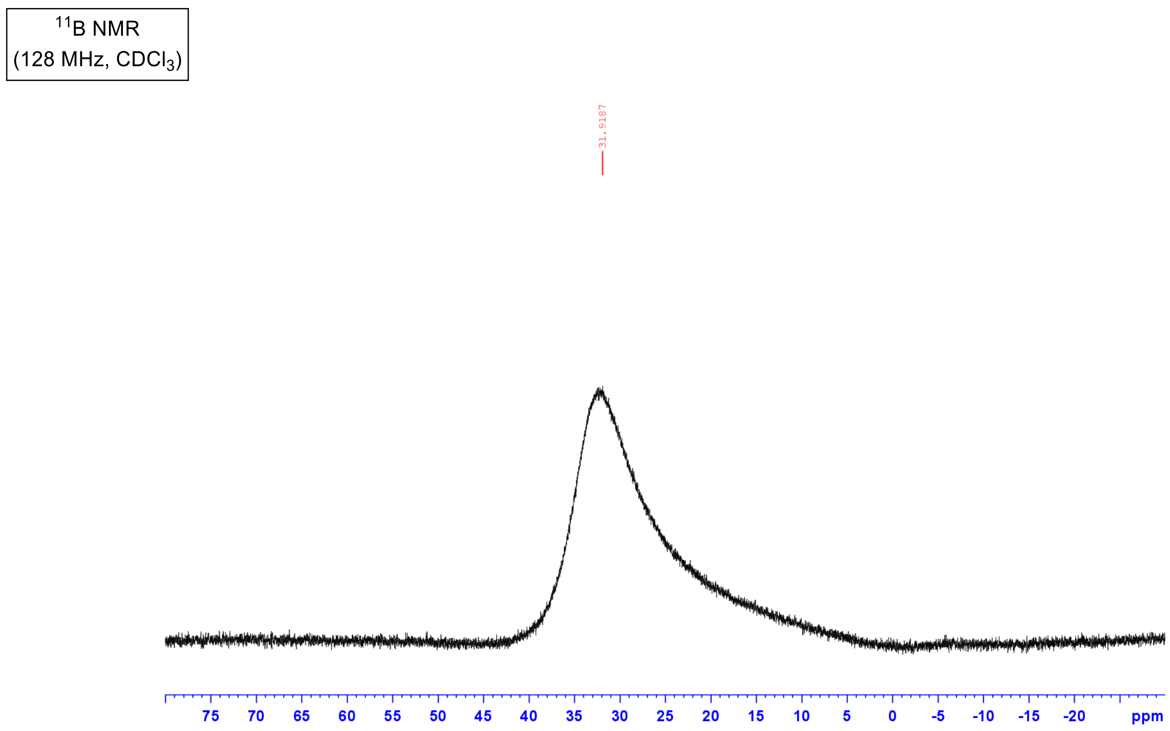

11B NMR
(128 MHz, CDCl3)
31.9187
75 70 65 60 55 50 45 40 35 30 25 20 15 10 5 0 -5 -10 -15 -20 ppm

[$^1$H, $^{13}$C{$^1$H}, and $^{11}$B NMR Spectra of ***anti*-3ga**]

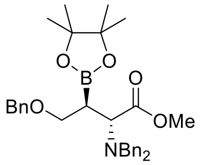


***anti*-3ga**

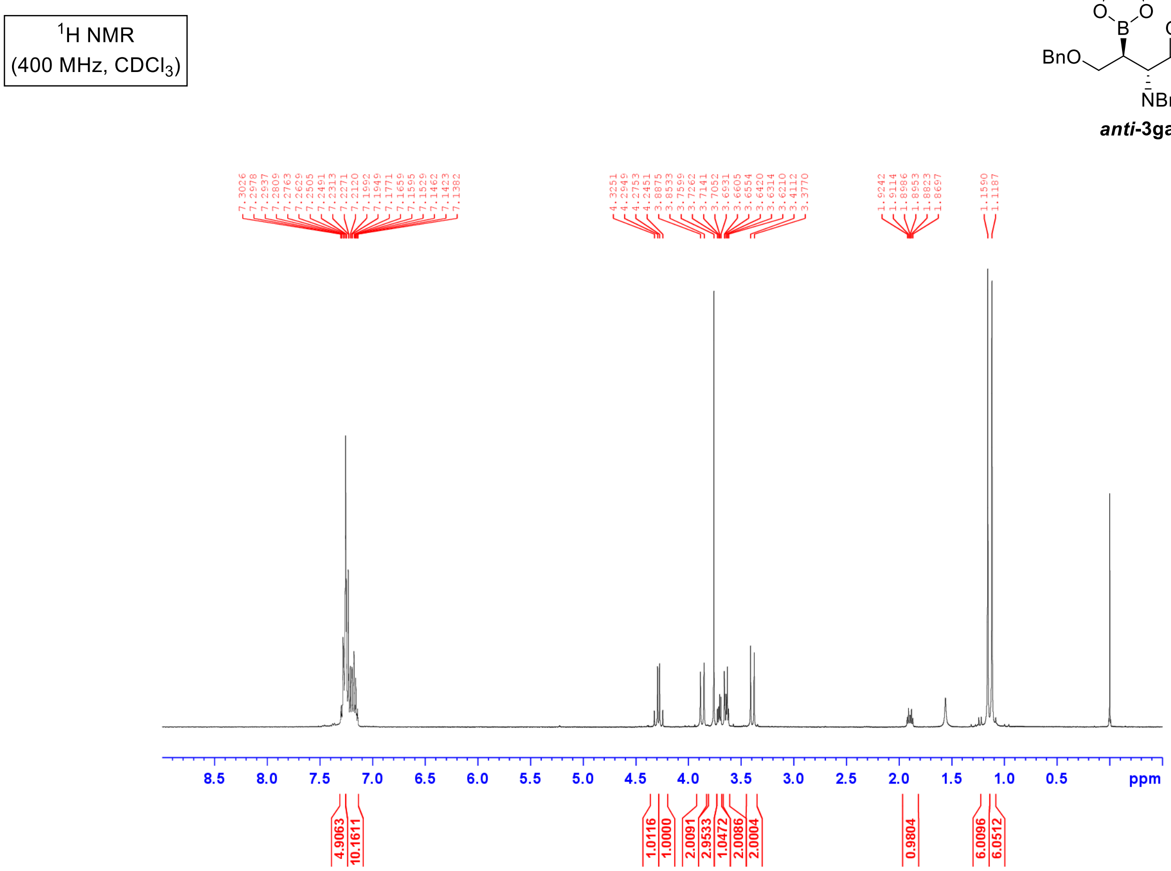


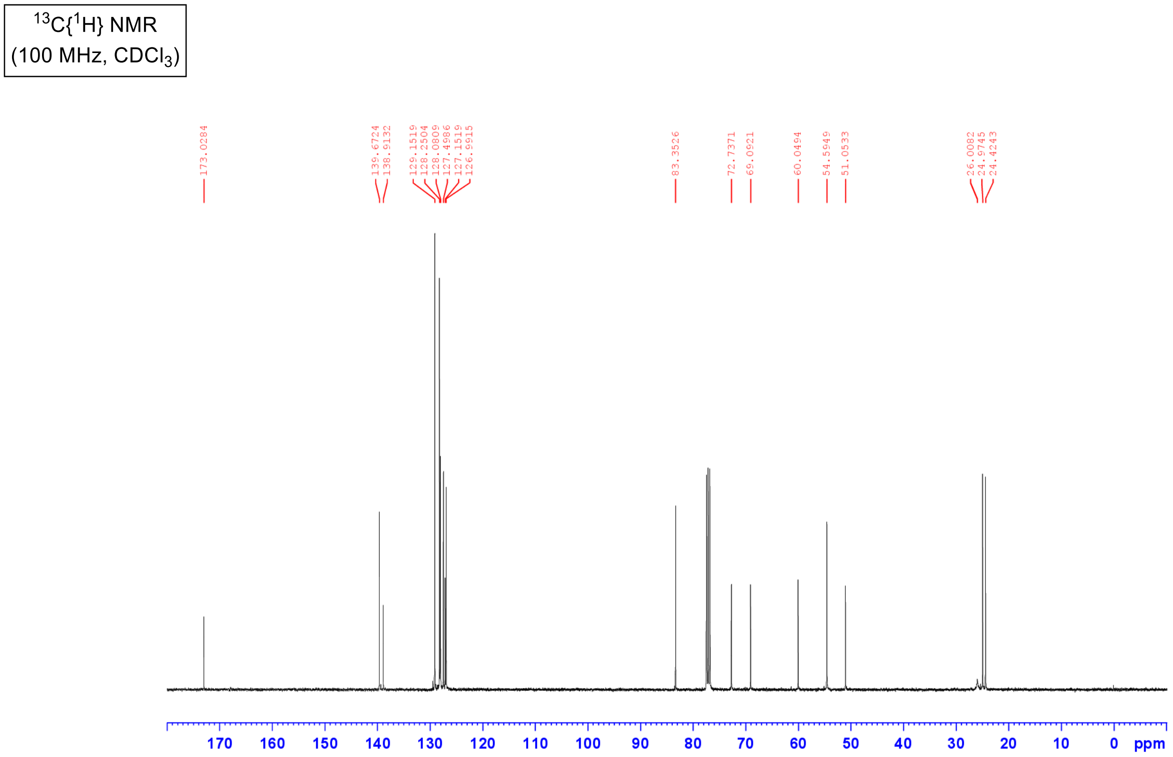

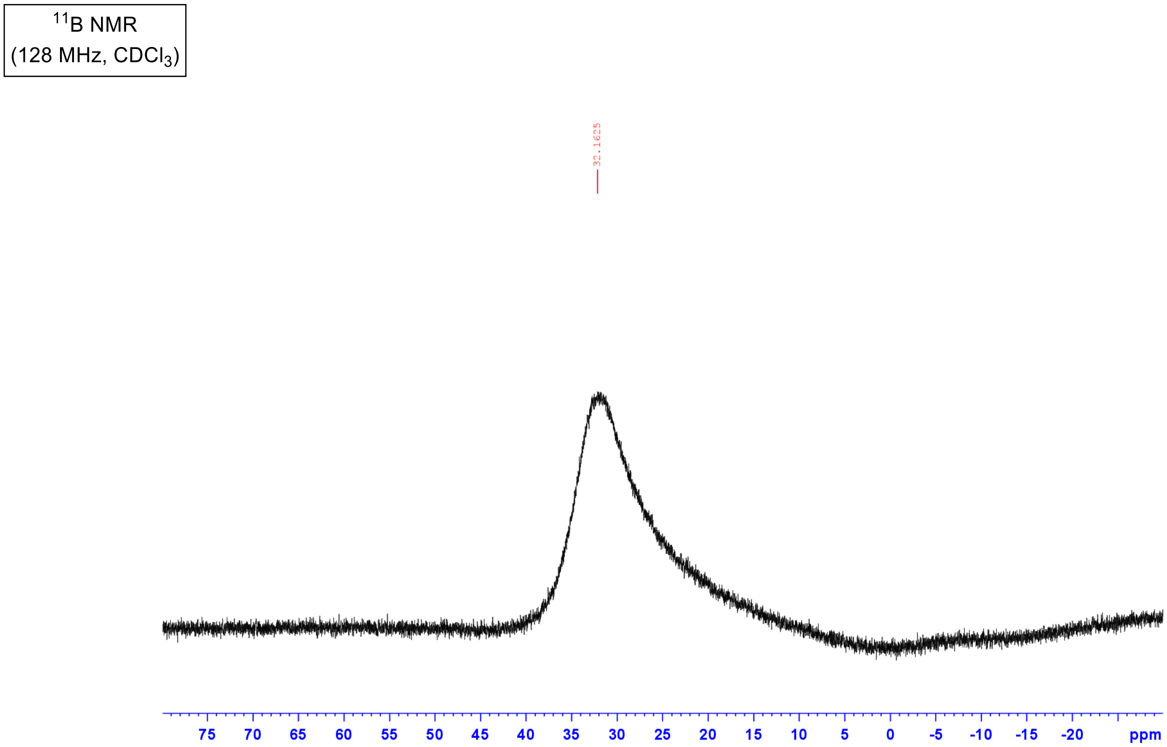

$^{11}B$ NMR
(128 MHz, $CDCl_3$)
32.1625
75 70 65 60 55 50 45 40 35 30 25 20 15 10 5 0 -5 -10 -15 -20 ppm

## [$^{1}$H, $^{13}$C{$^{1}$H}, and $^{11}$B NMR Spectra of ***anti*-3ha**]

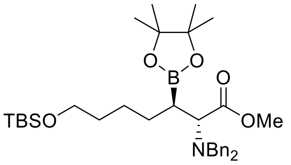


***anti*-3ha**

$^{1}$H NMR
(400 MHz, $CDCl_3$)

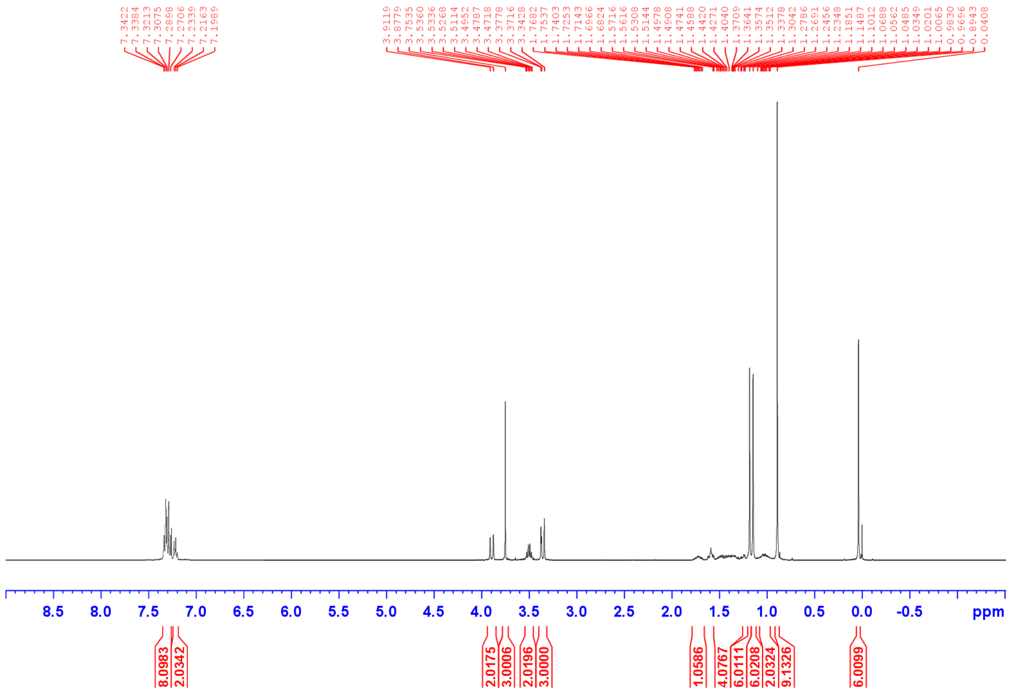

$^{13}$C{$^{1}$H} NMR
(100 MHz, $CDCl_3$)

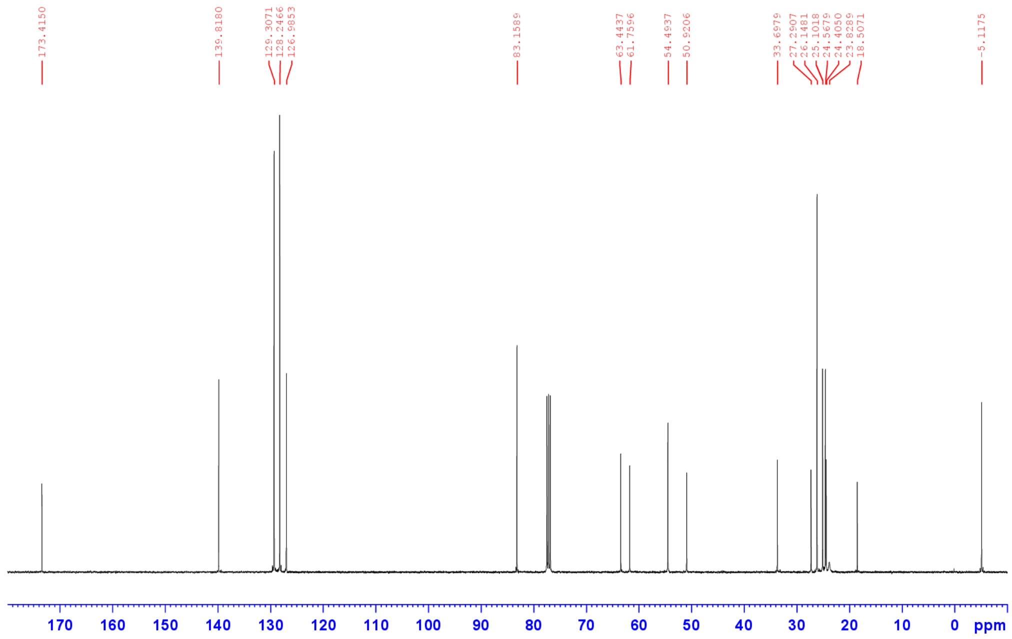

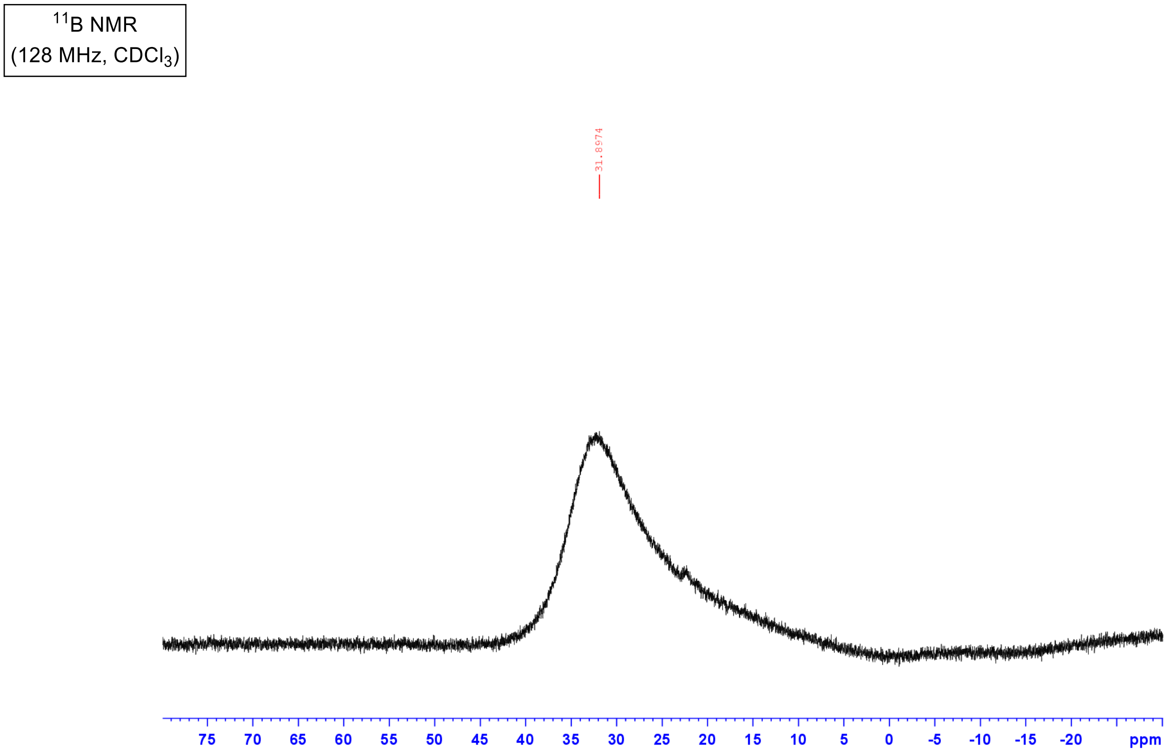

$^{11}$B NMR
(128 MHz, CDCl$_3$)
31.8974
75 70 65 60 55 50 45 40 35 30 25 20 15 10 5 0 -5 -10 -15 -20 ppm

[$^{1}$H, $^{13}$C{$^{1}$H}, and $^{11}$B NMR Spectra of ***anti*-3ia**]

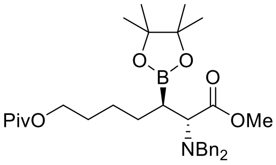

***anti*-3ia**

$^{1}$H NMR
(400 MHz, $CDCl_3$)

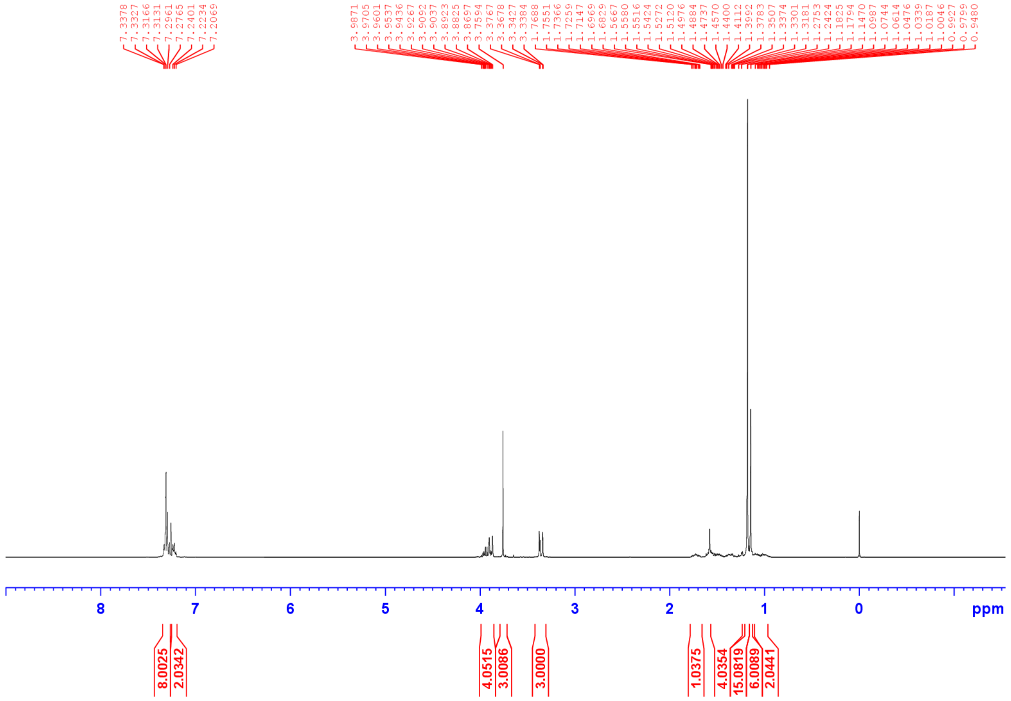


$^{13}$C{$^{1}$H} NMR
(100 MHz, $CDCl_3$)

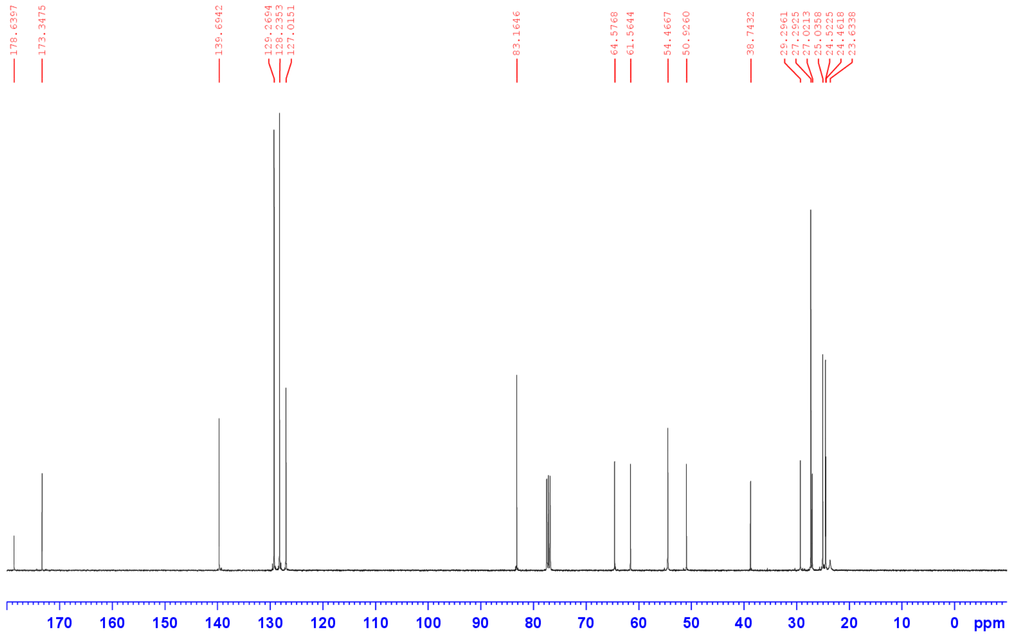

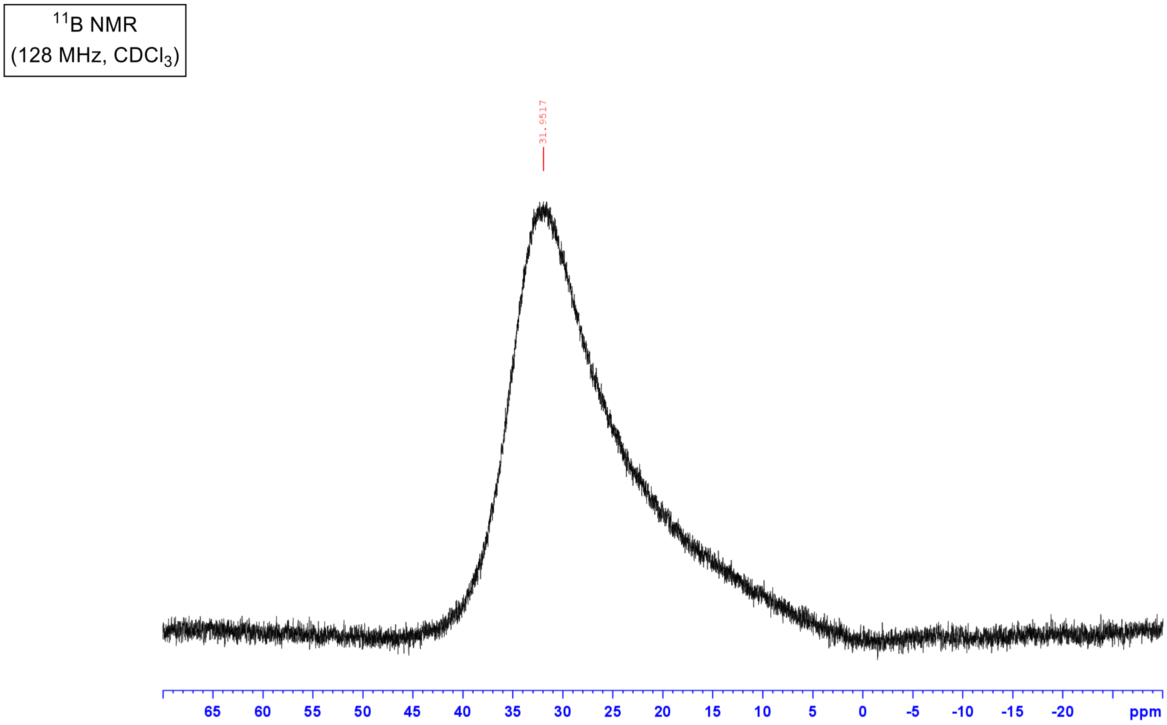

$^{11}B$ NMR
(128 MHz, $CDCl_3$)
31.9517
65 60 55 50 45 40 35 30 25 20 15 10 5 0 -5 -10 -15 -20 ppm

[$^{1}$H, $^{13}$C{$^{1}$H}, and $^{11}$B NMR Spectra of ***anti*-3ja**]

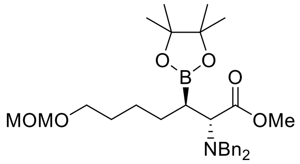


***anti*-3ja**

$^{1}$H NMR
(400 MHz, $CDCl_3$)

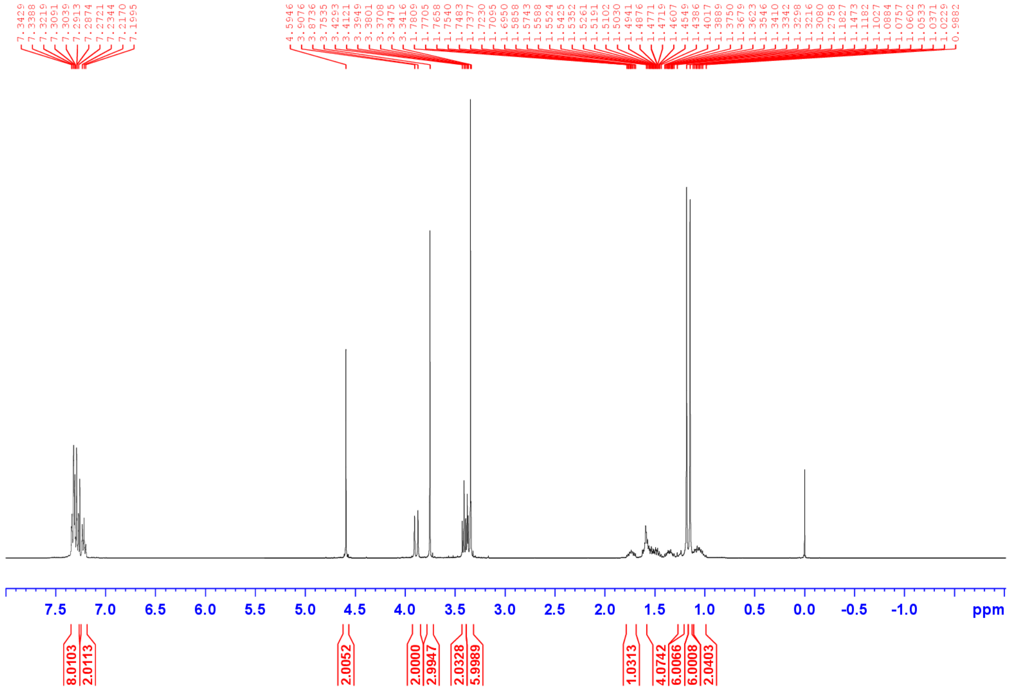


$^{13}$C{$^{1}$H} NMR
(100 MHz, $CDCl_3$)

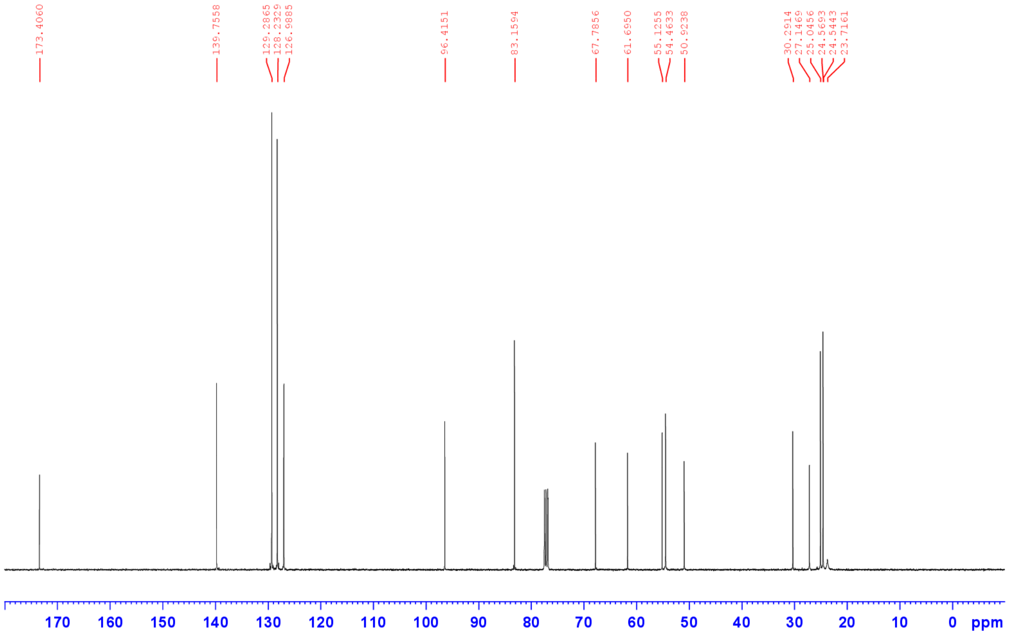

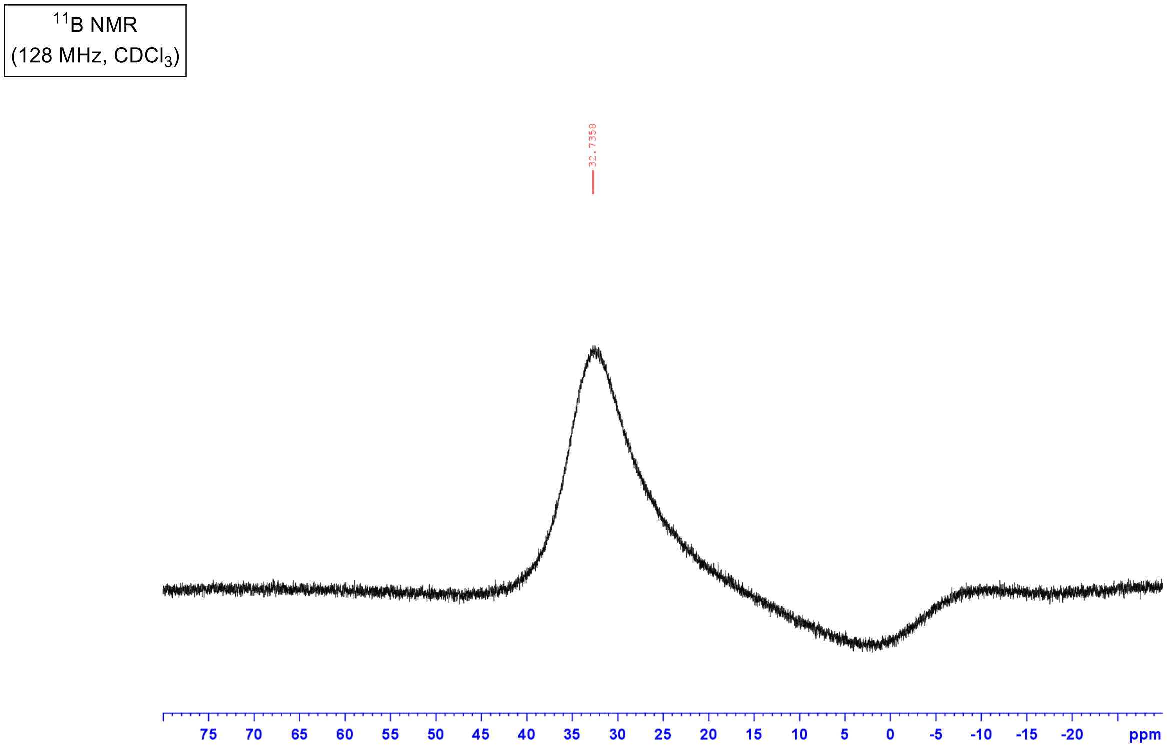
$^{11}$B NMR
(128 MHz, $CDCl_3$)
32.7358
75 70 65 60 55 50 45 40 35 30 25 20 15 10 5 0 -5 -10 -15 -20 ppm

[$^{1}H$, $^{13}C\{^{1}H\}$, and $^{11}B$ NMR Spectra of ***anti*-3ka**]

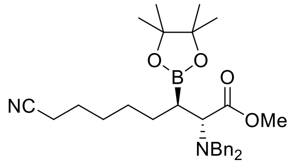


*anti*-3ka

$^{1}H$ NMR
(400 MHz, $CDCl_3$)

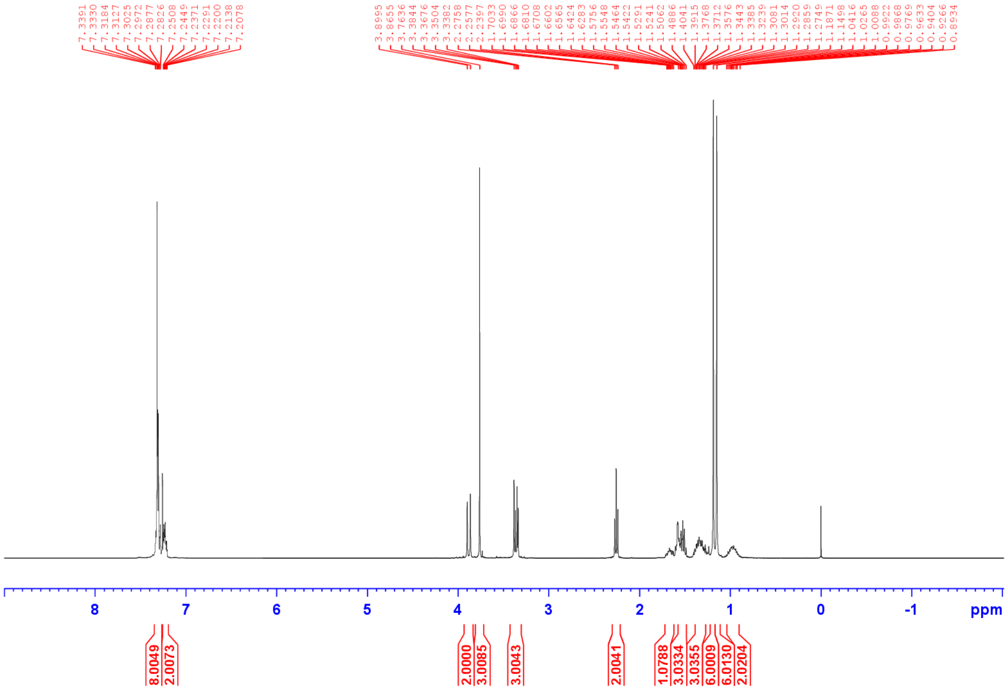


$^{13}C\{^{1}H\}$ NMR
(100 MHz, $CDCl_3$)

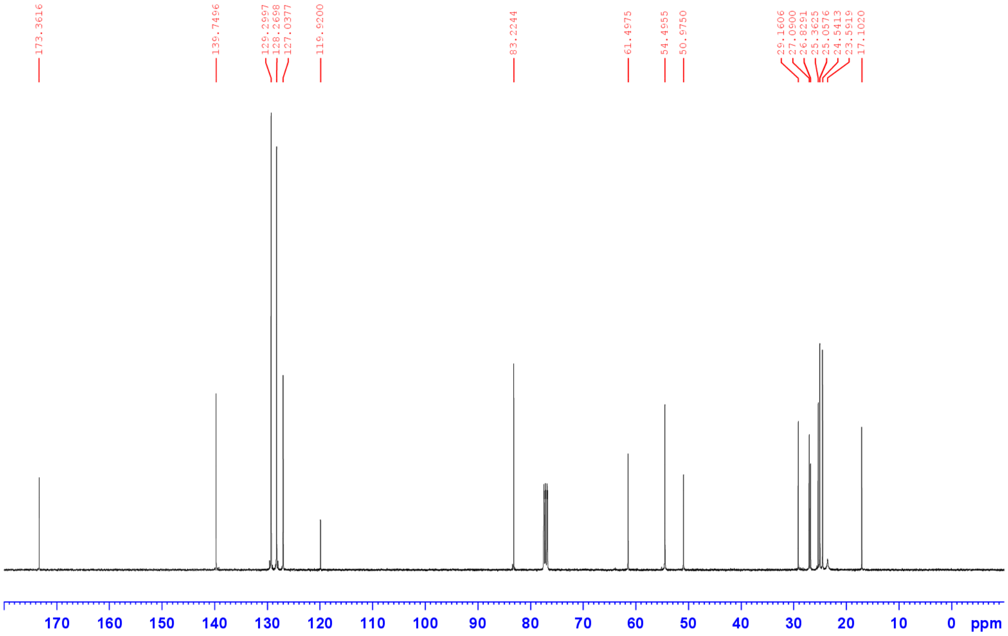

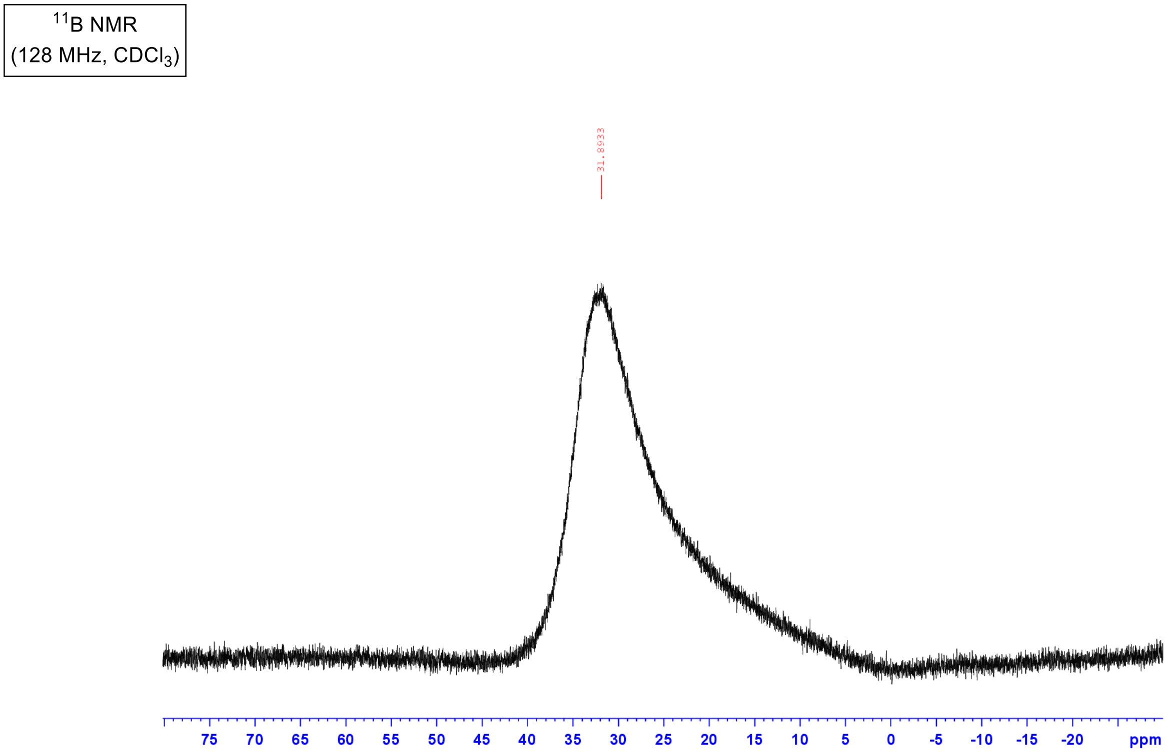

$^{11}B$ NMR
(128 MHz, $CDCl_3$)
31.8933
75 70 65 60 55 50 45 40 35 30 25 20 15 10 5 0 -5 -10 -15 -20 ppm

## [$^{1}H$, $^{13}C\{^{1}H\}$, and $^{11}B$ NMR Spectra of ***anti*-3la**]

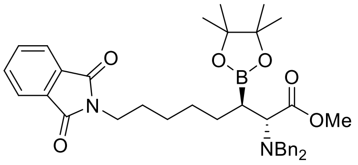


***anti*-3la**

$^{1}H$ NMR
(400 MHz, $CDCl_3$)

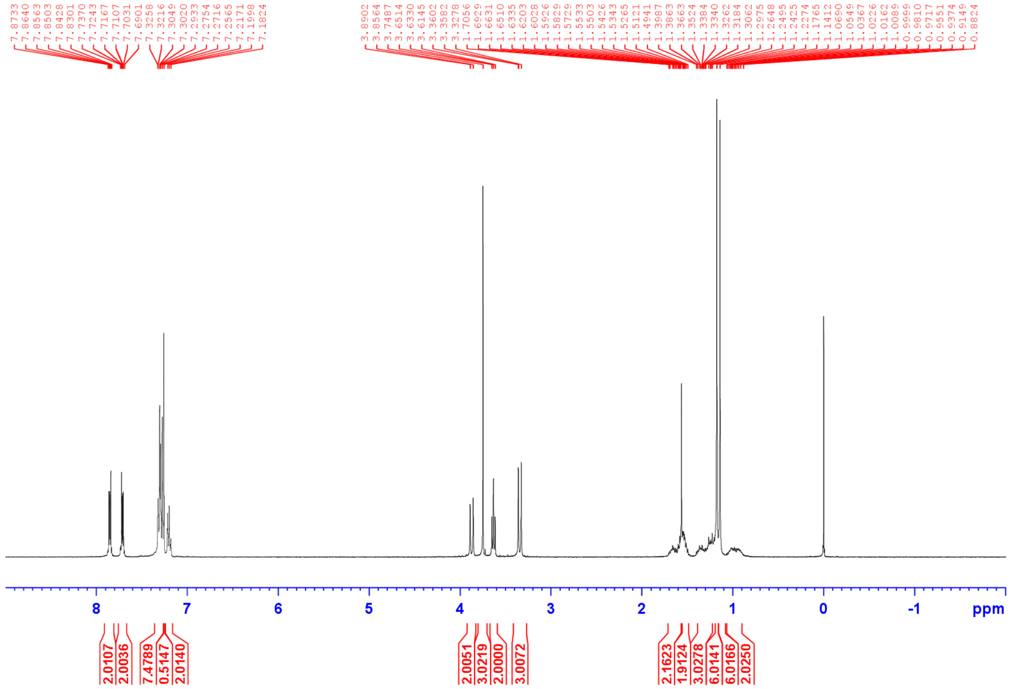


$^{13}C\{^{1}H\}$ NMR
(100 MHz, $CDCl_3$)

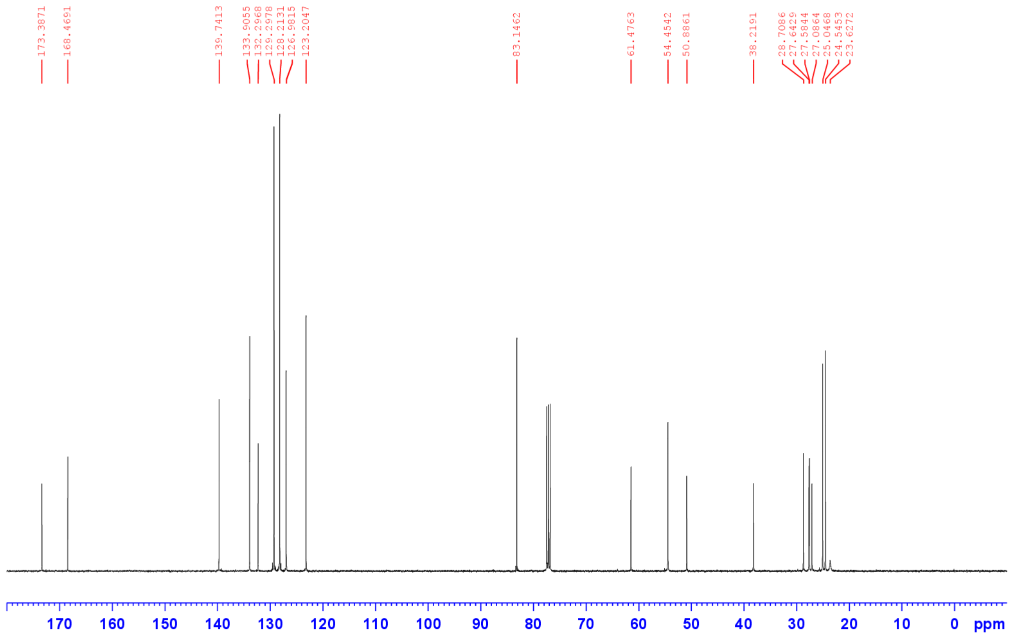

$^{11}B$ NMR
(128 MHz, $CDCl_3$)

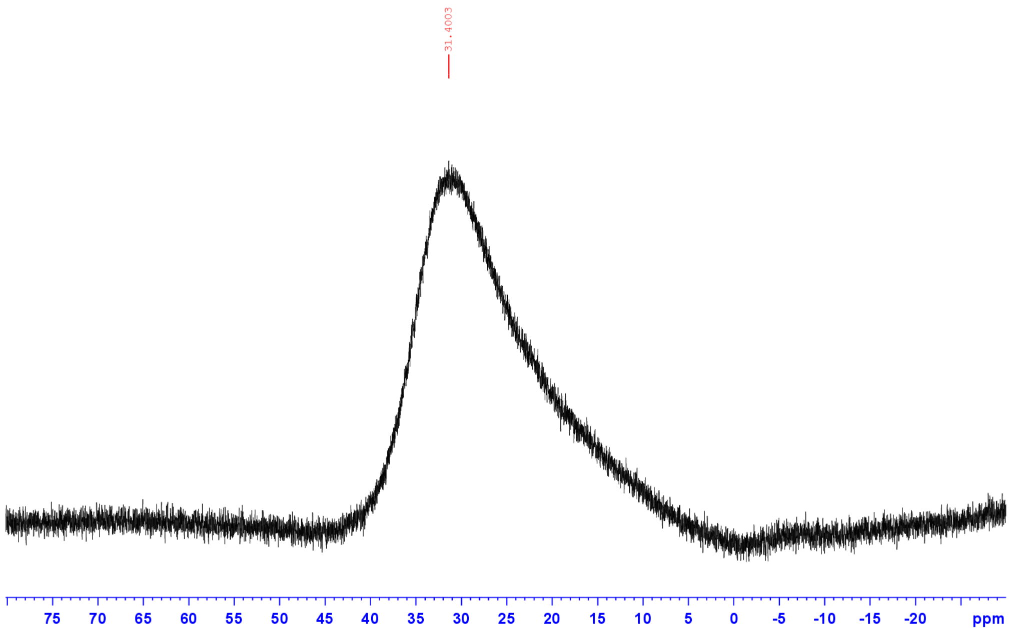

[$^{1}H$, $^{13}C\{^{1}H\}$, and $^{11}B$ NMR Spectra of ***anti*-3ma**]

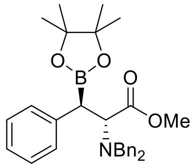


***anti*-3ma**

$^{1}H$ NMR
(400 MHz, $CDCl_3$)

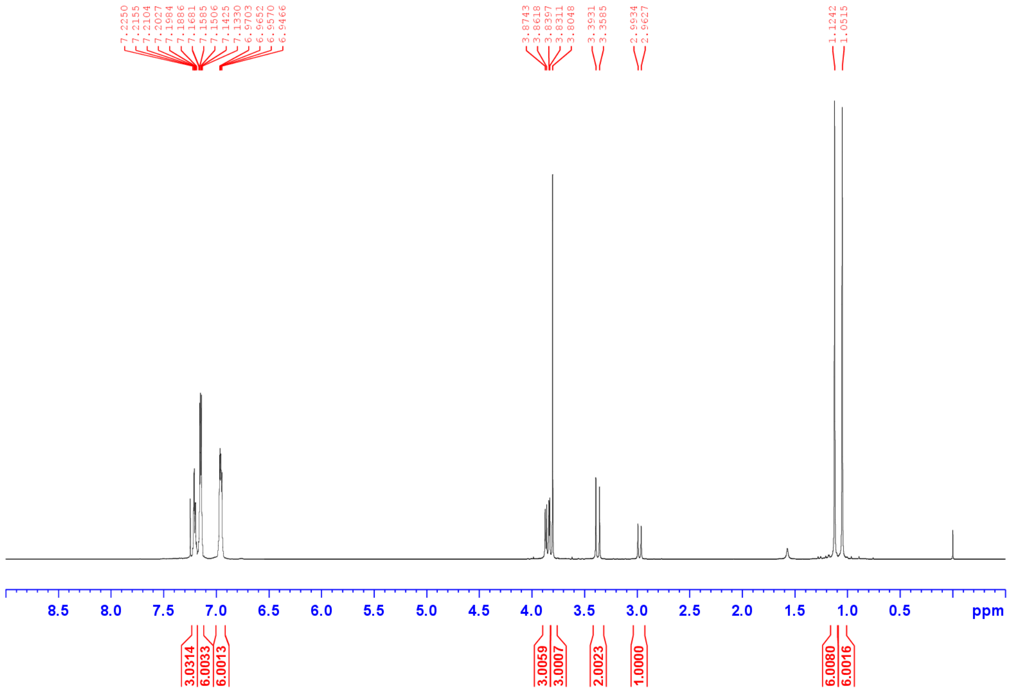


$^{13}C\{^{1}H\}$ NMR
(100 MHz, $CDCl_3$)

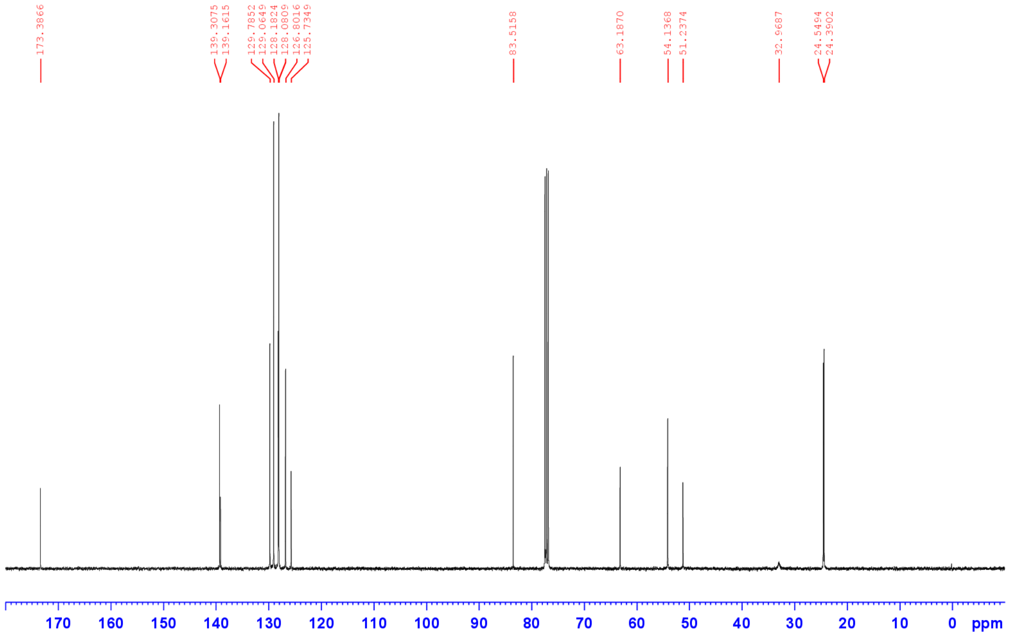

$^{11}B$ NMR
(128 MHz, $CDCl_3$)

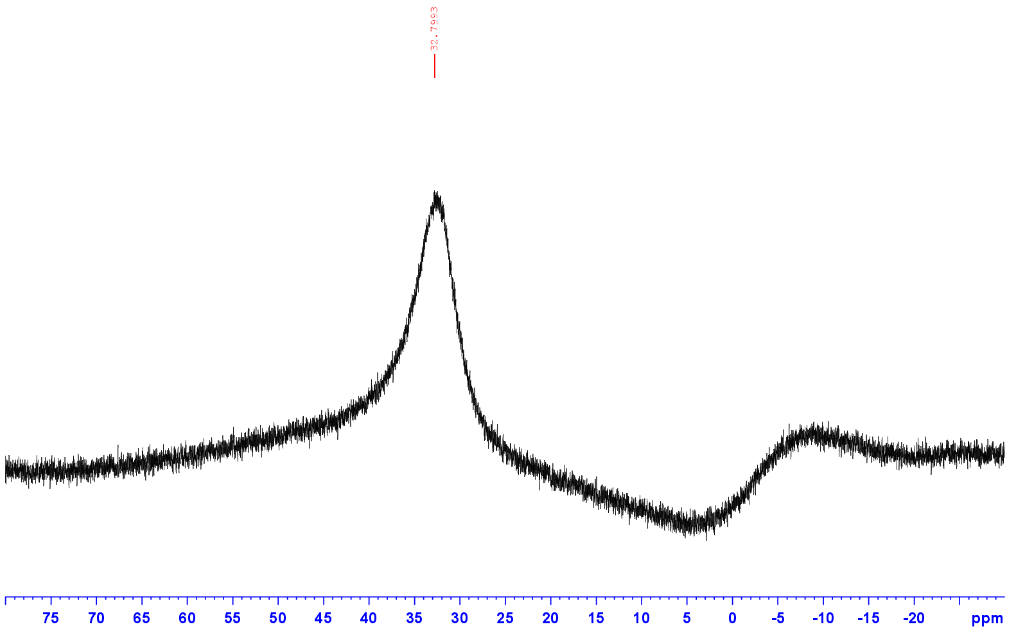

[$^{1}$H, $^{13}$C{$^{1}$H}, and $^{11}$B NMR Spectra of ***anti*-3na**]

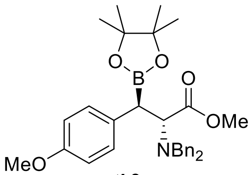


***anti*-3na**

$^{1}$H NMR
(400 MHz, $CDCl_3$)

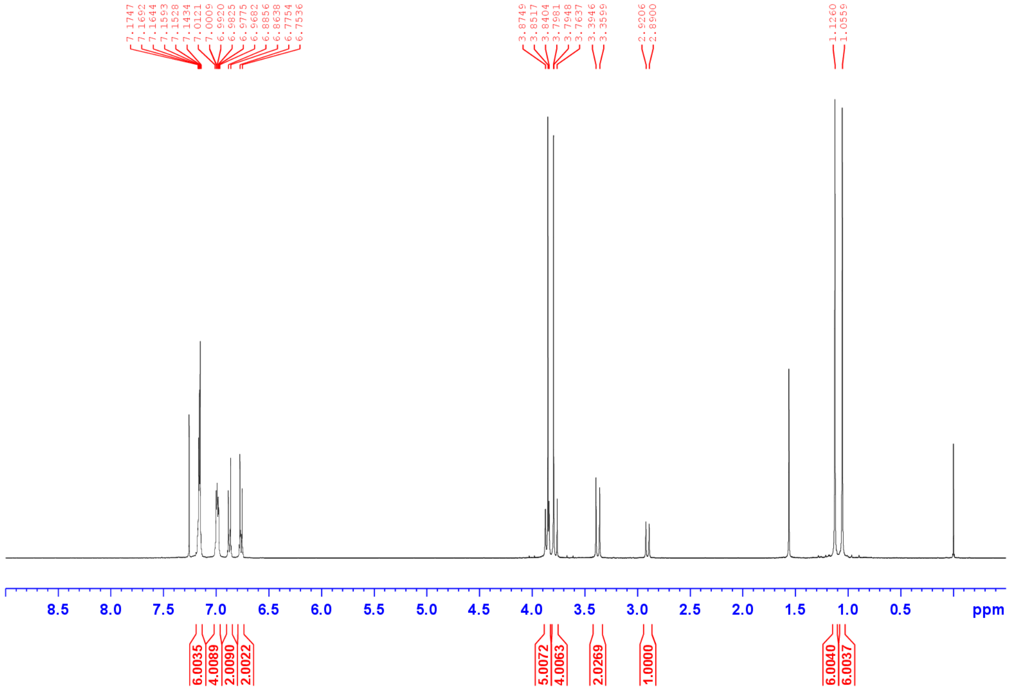


$^{13}$C{$^{1}$H} NMR
(100 MHz, $CDCl_3$)

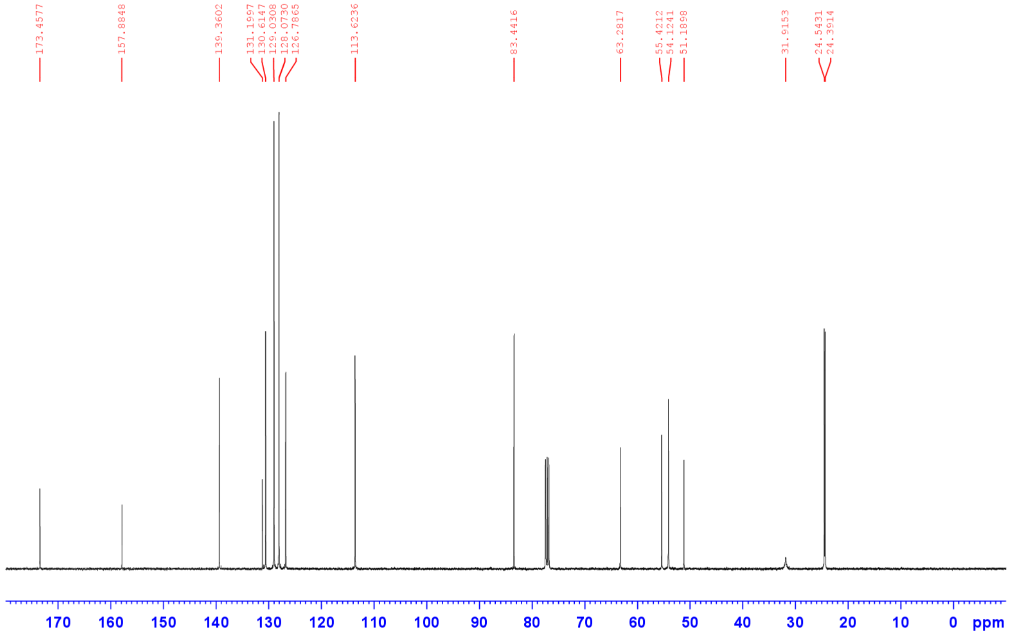

$^{11}$B NMR
(128 MHz, $CDCl_3$)

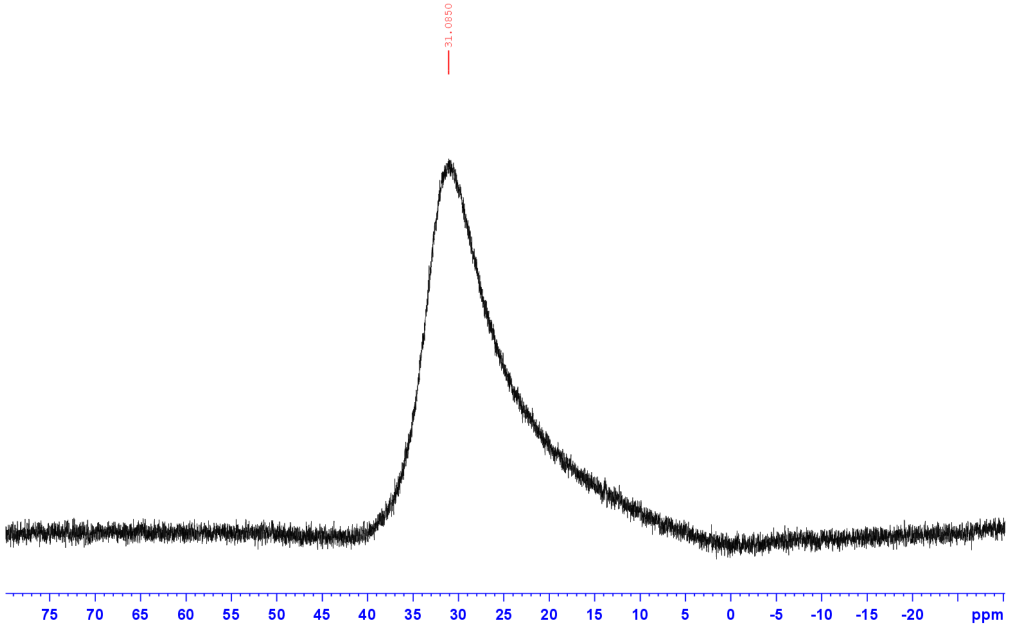

[$^{1}$H, $^{13}$C{$^{1}$H}, and $^{11}$B NMR Spectra of ***anti*-3oa**]

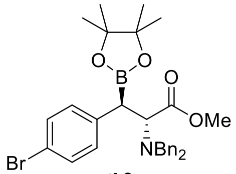

***anti*-3oa**

$^{1}$H NMR
(400 MHz, $CDCl_3$)

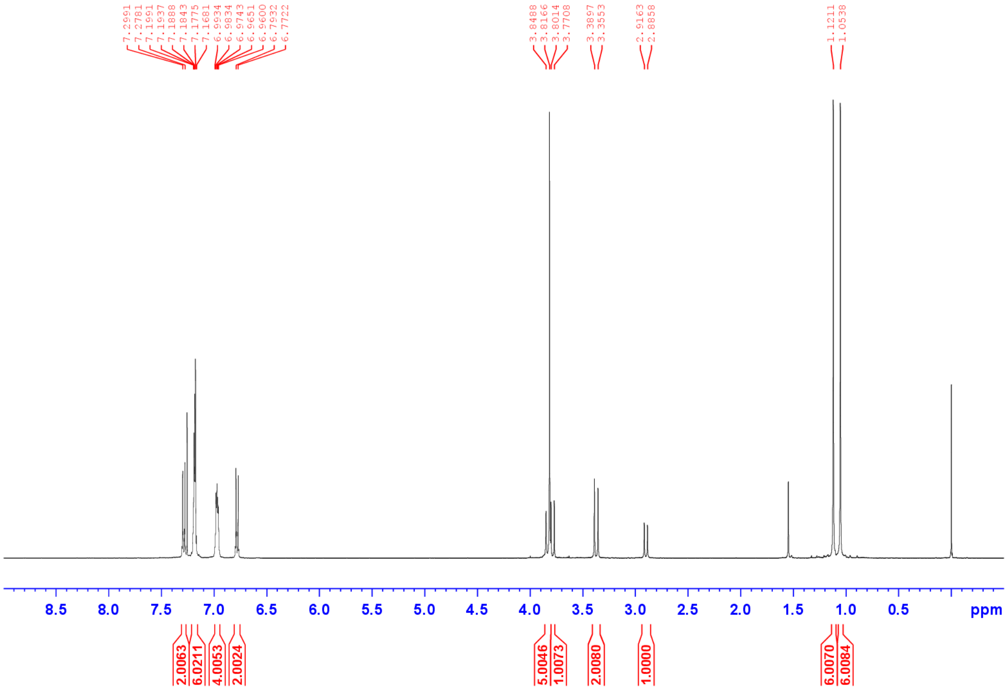

$^{13}$C{$^{1}$H} NMR
(100 MHz, $CDCl_3$)

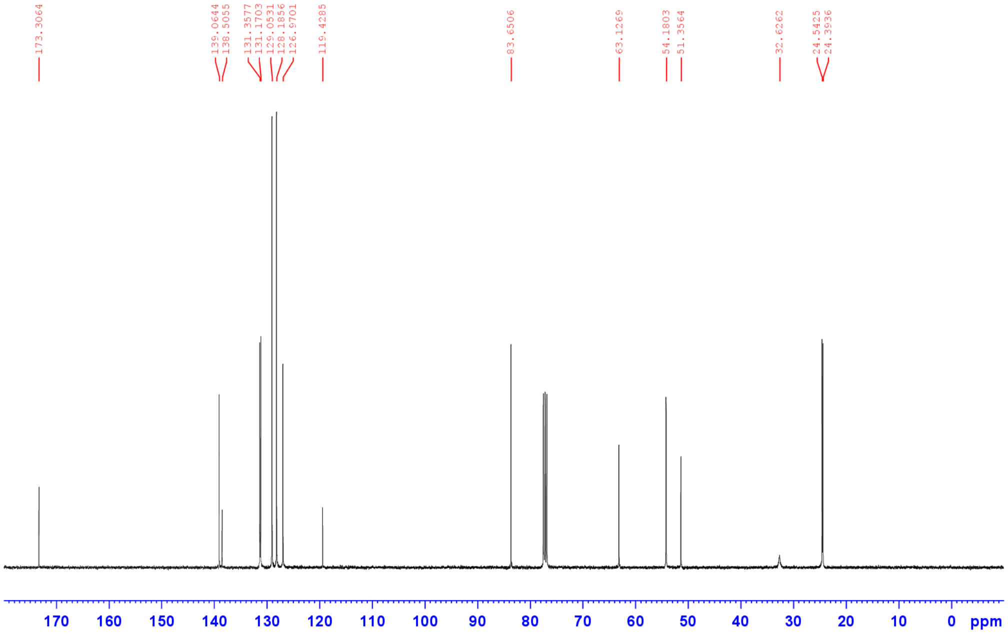

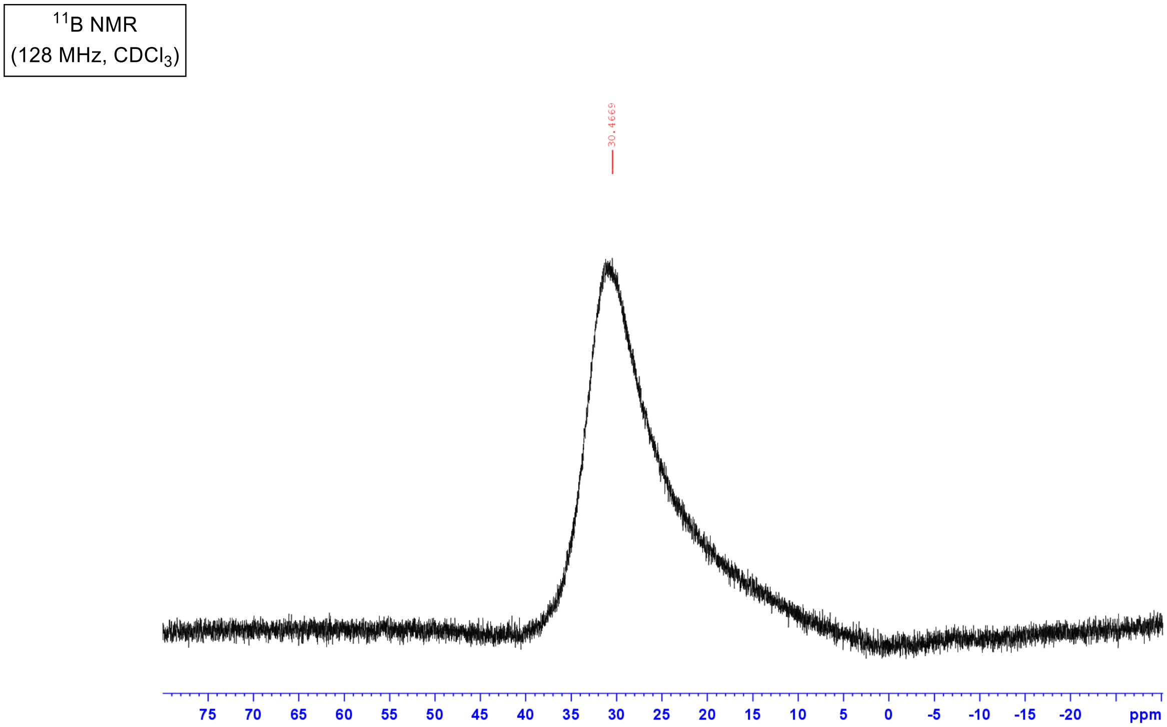

$^{11}$B NMR
(128 MHz, $CDCl_3$)
30.4669
75
70
65
60
55
50
45
40
35
30
25
20
15
10
5
0
-5
-10
-15
-20
ppm

[$^1$H, $^{13}$C{$^1$H}, and $^{11}$B NMR Spectra of ***anti*-3pa**]

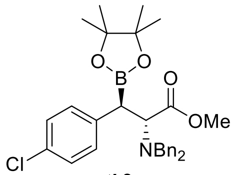


***anti*-3pa**

$^1$H NMR
(400 MHz, $CDCl_3$)

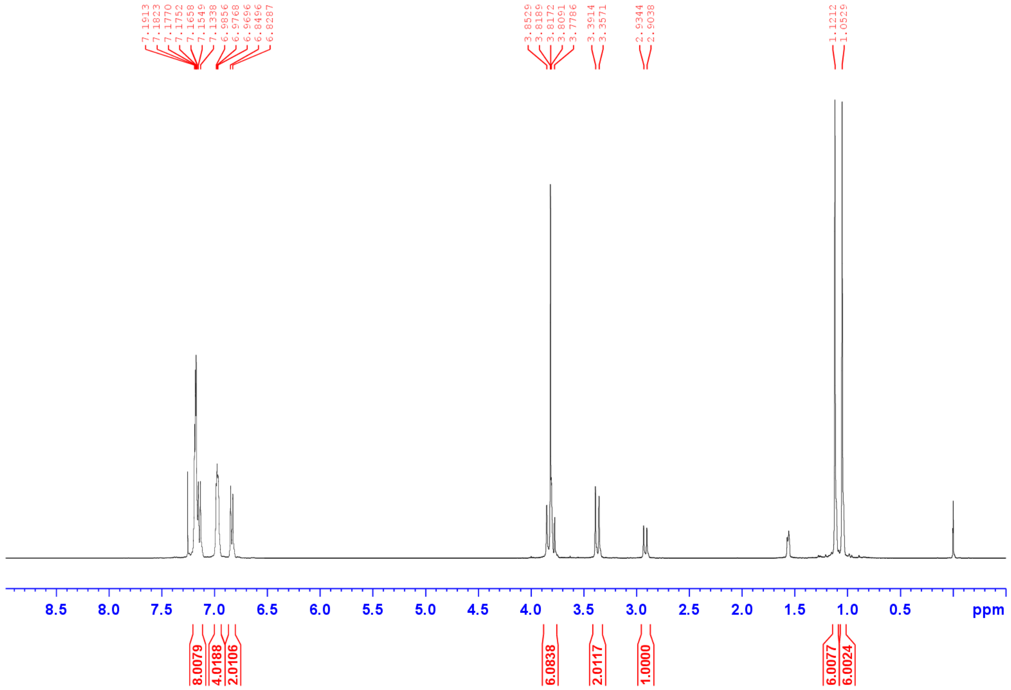


$^{13}$C{$^1$H} NMR
(100 MHz, $CDCl_3$)

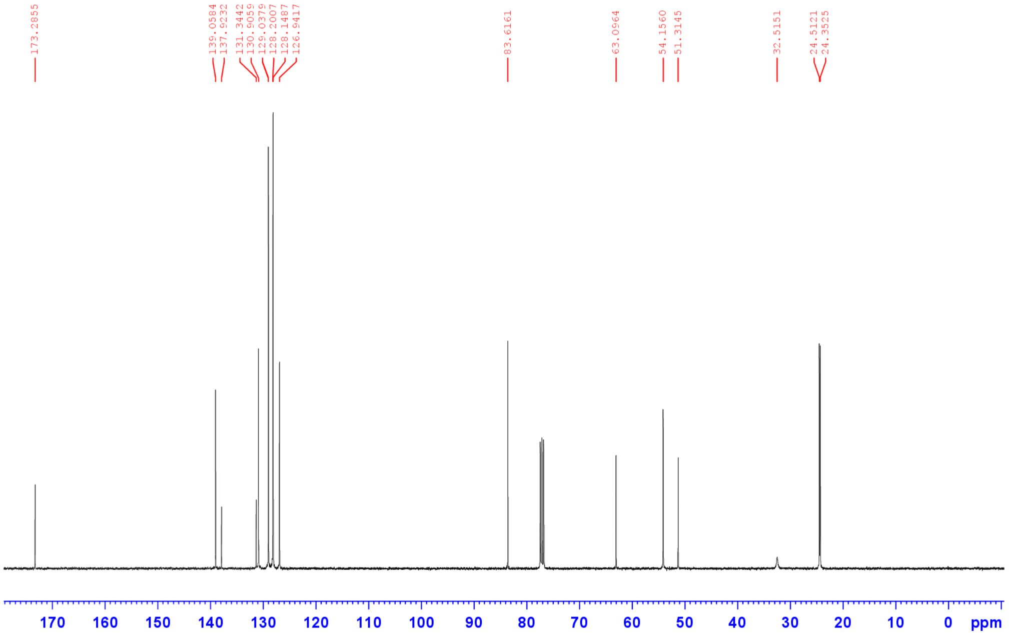

$^{11}B$ NMR
(128 MHz, $CDCl_3$)

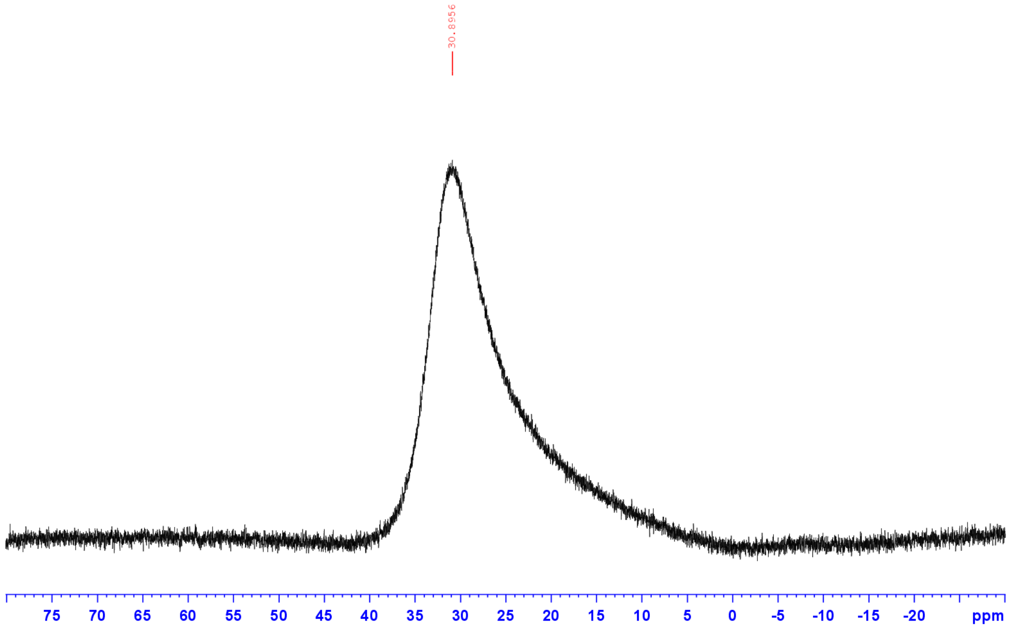

[$^{1}H$, $^{13}C\{^{1}H\}$, $^{19}F\{^{1}H\}$, and $^{11}B$ NMR Spectra of ***anti*-3qa**]

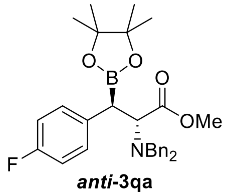


***anti*-3qa**

$^{1}H$ NMR
(400 MHz, $CDCl_3$)

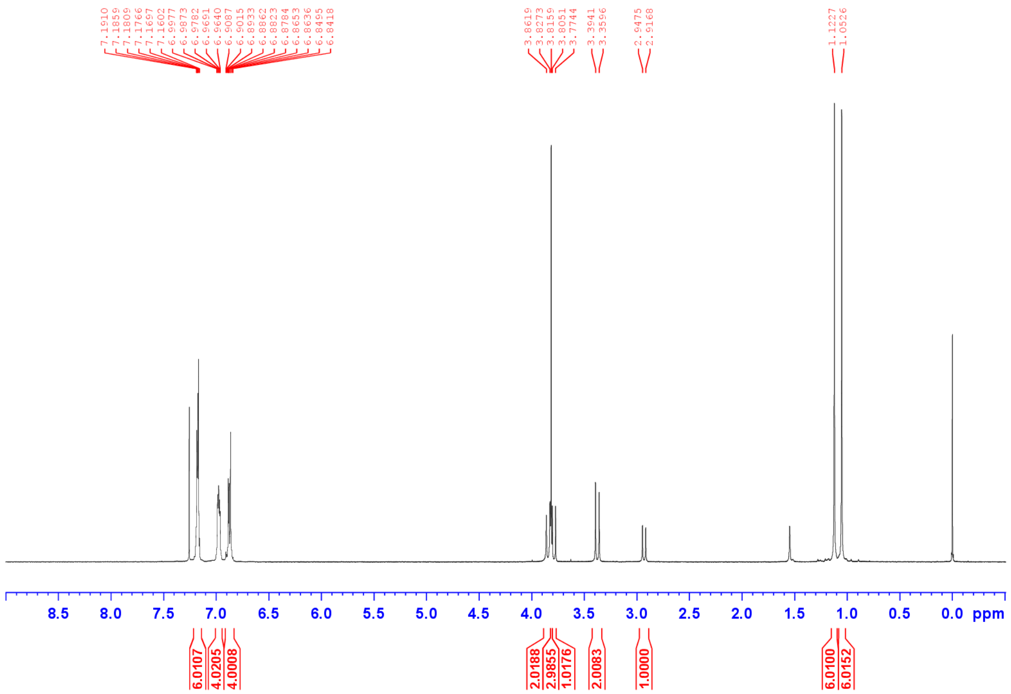


$^{13}C\{^{1}H\}$ NMR
(100 MHz, $CDCl_3$)

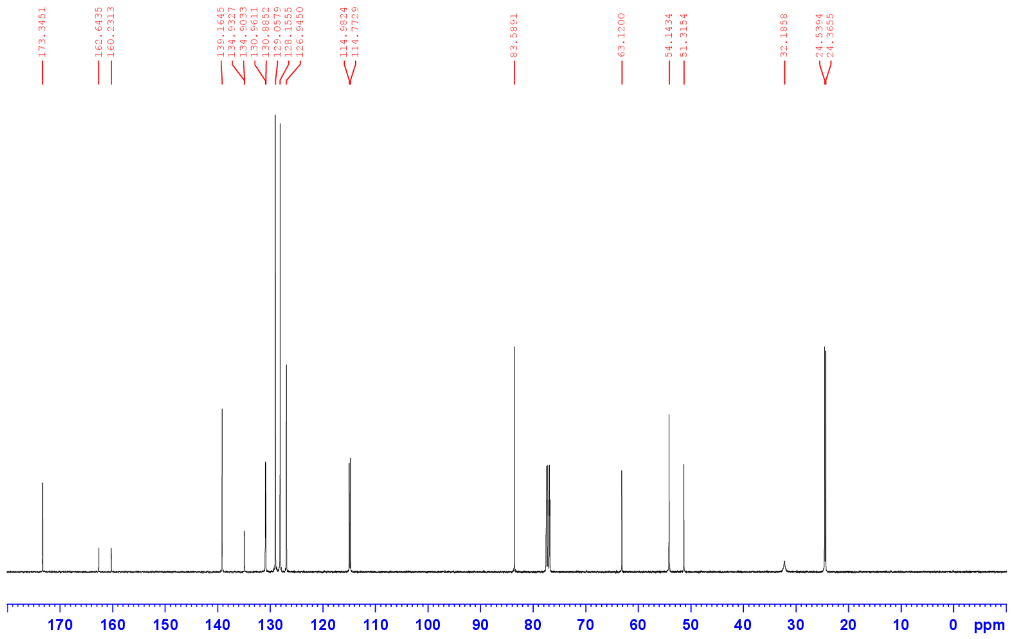

$^{19}F\{^1H\}$ NMR
(376 MHz, $CDCl_3$)

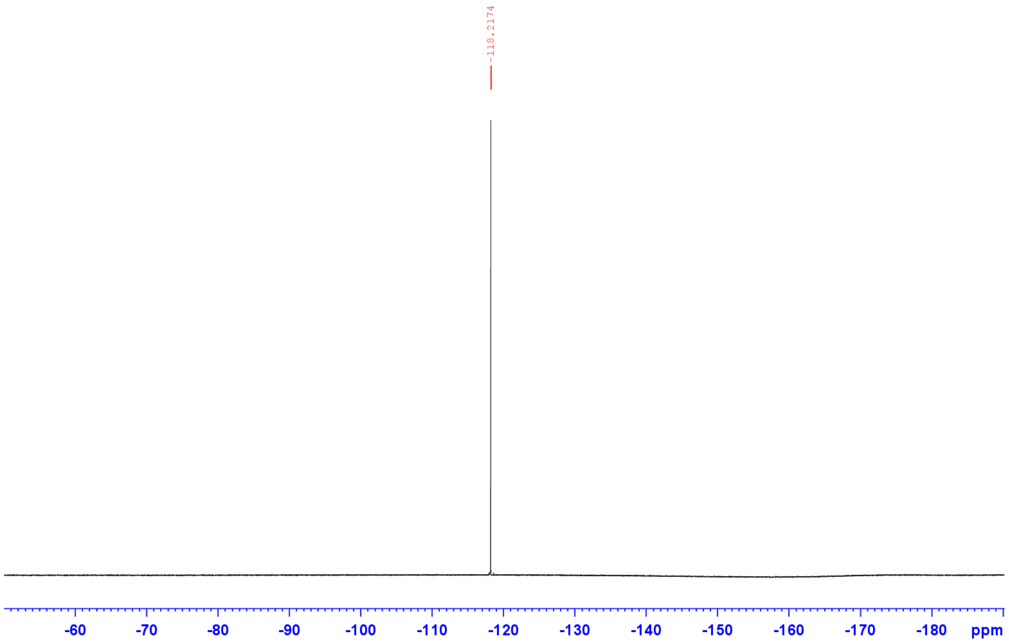


$^{11}B$ NMR
(128 MHz, $CDCl_3$)

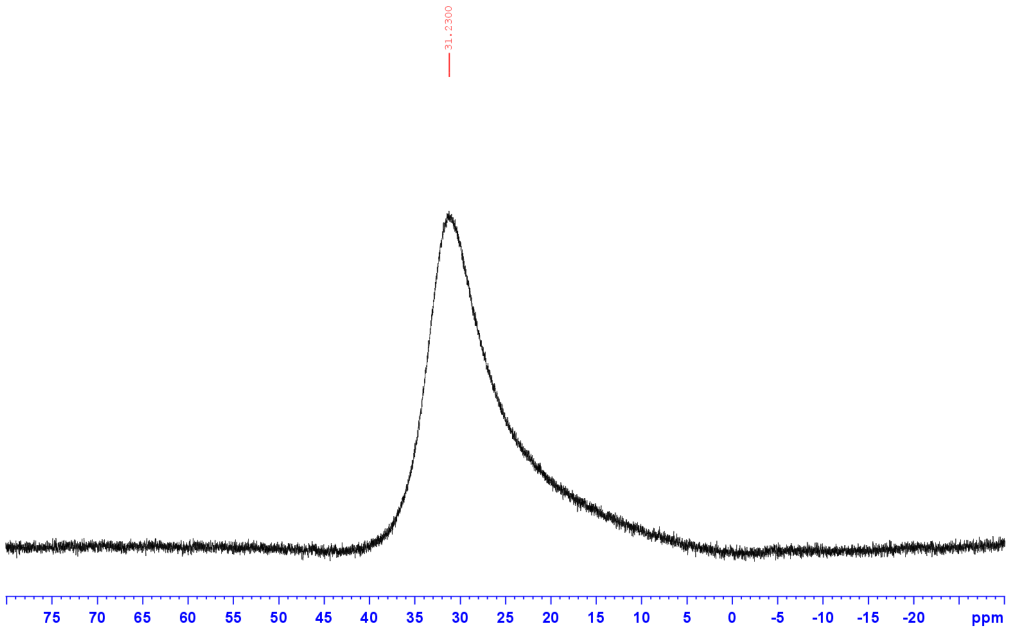

[$^{1}H$, $^{13}C\{^{1}H\}$, $^{19}F\{^{1}H\}$, and $^{11}B$ NMR Spectra of ***anti*-3ra**]

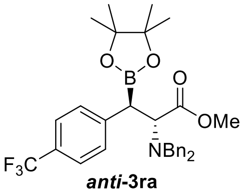


***anti*-3ra**

$^{1}H$ NMR
(400 MHz, $CDCl_3$)

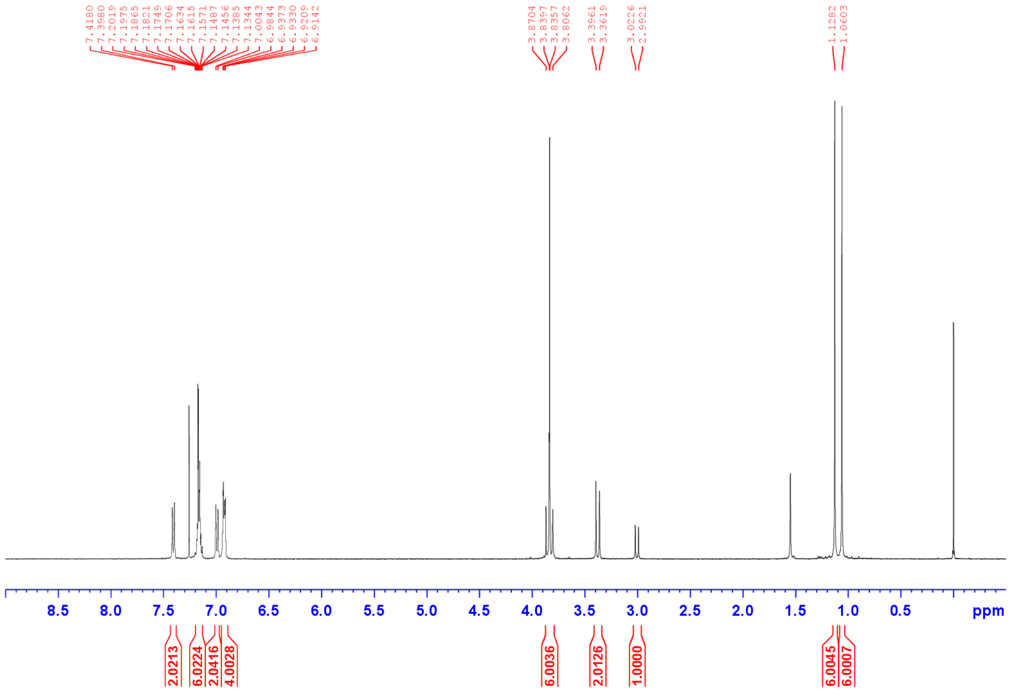

$^{13}C\{^{1}H\}$ NMR
(100 MHz, $CDCl_3$)

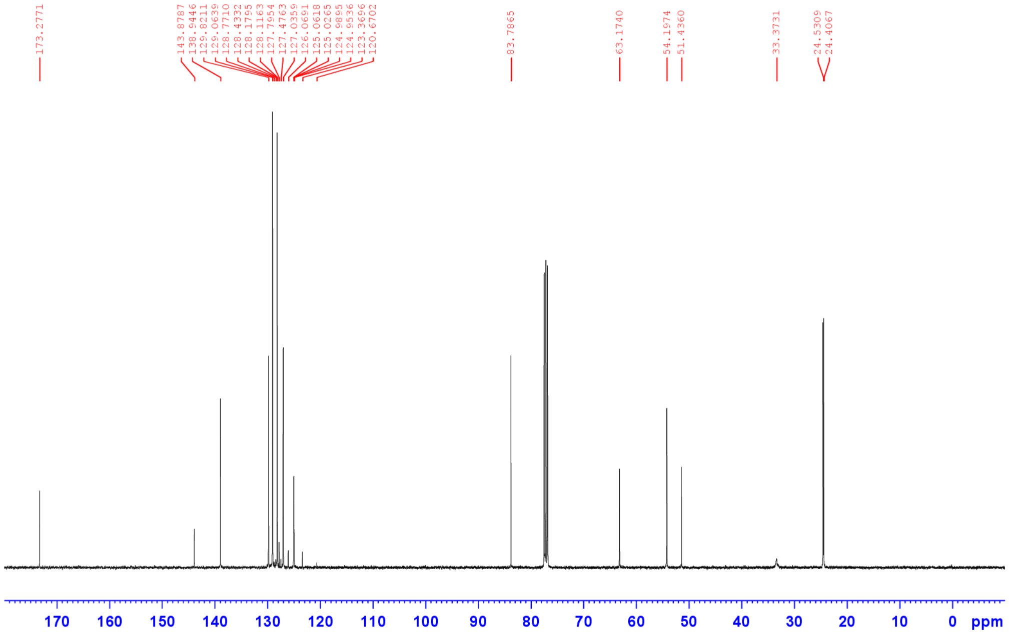

$^{19}F\{^{1}H\}$ NMR
(376 MHz, $CDCl_3$)

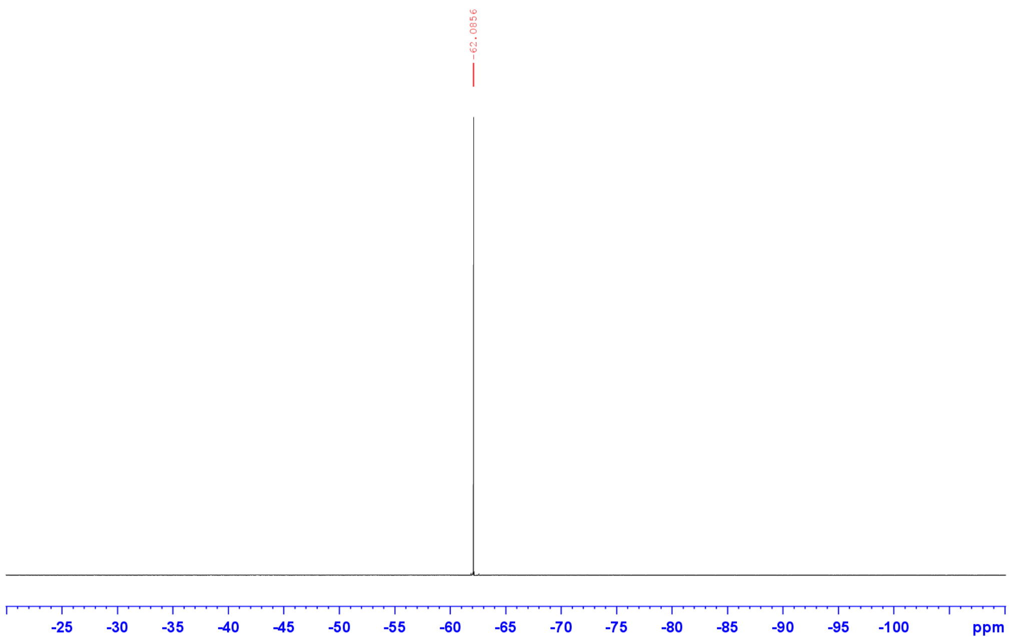


$^{11}B$ NMR
(128 MHz, $CDCl_3$)

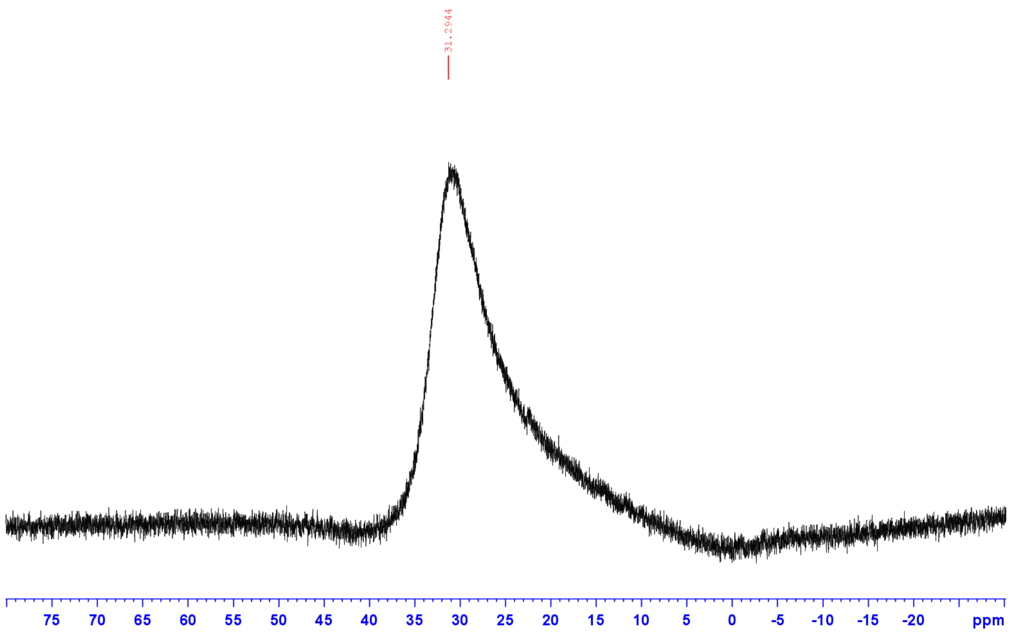

[$^{1}$H, $^{13}$C{$^{1}$H}, and $^{11}$B NMR Spectra of ***anti*-3sa**]

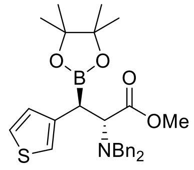


***anti*-3sa**

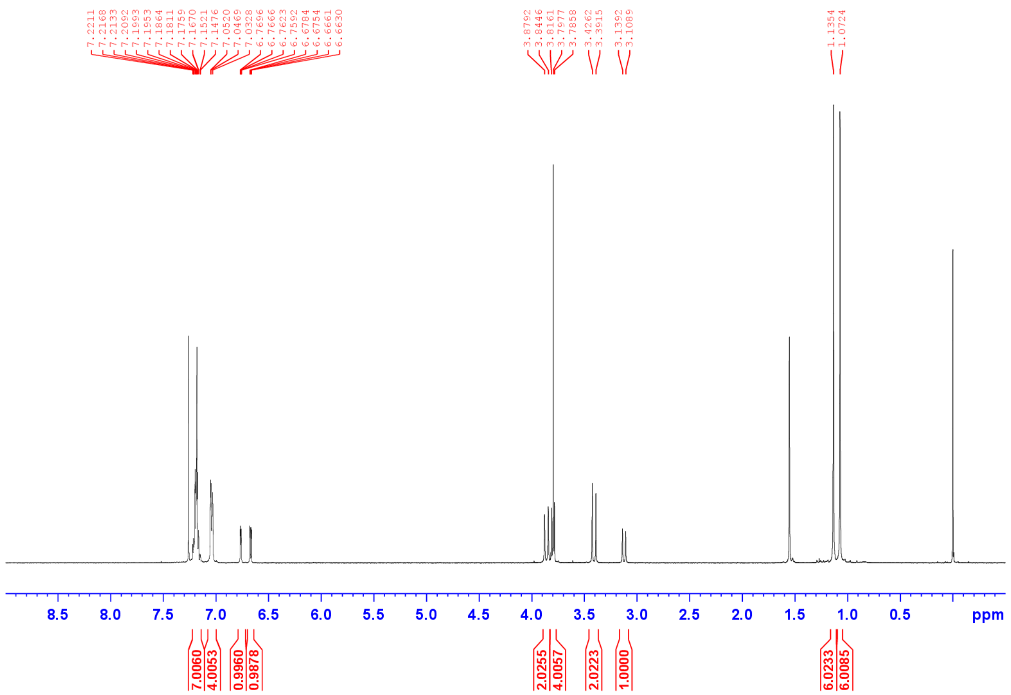


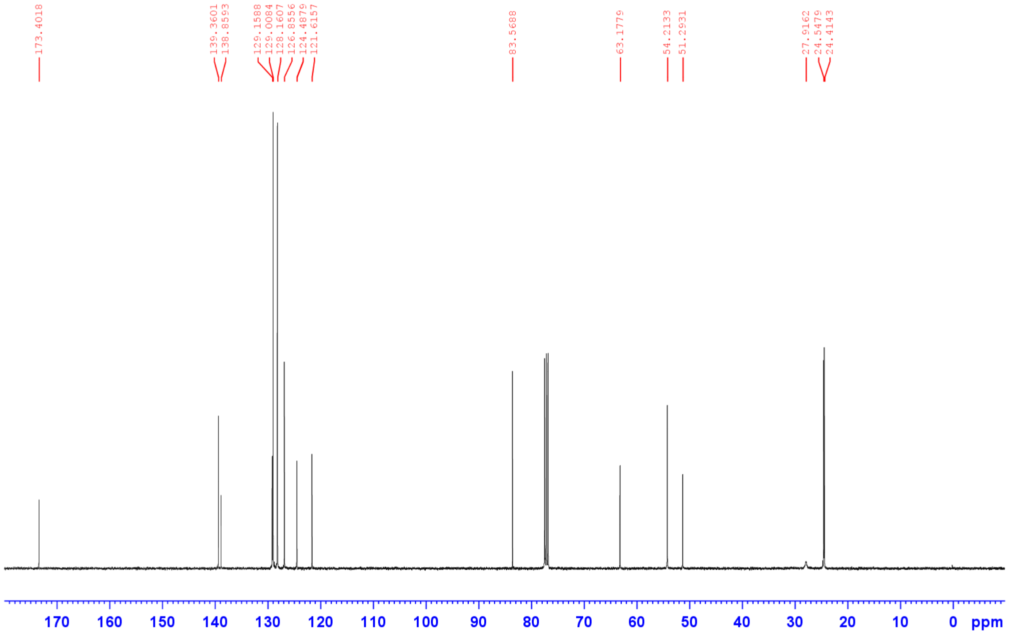

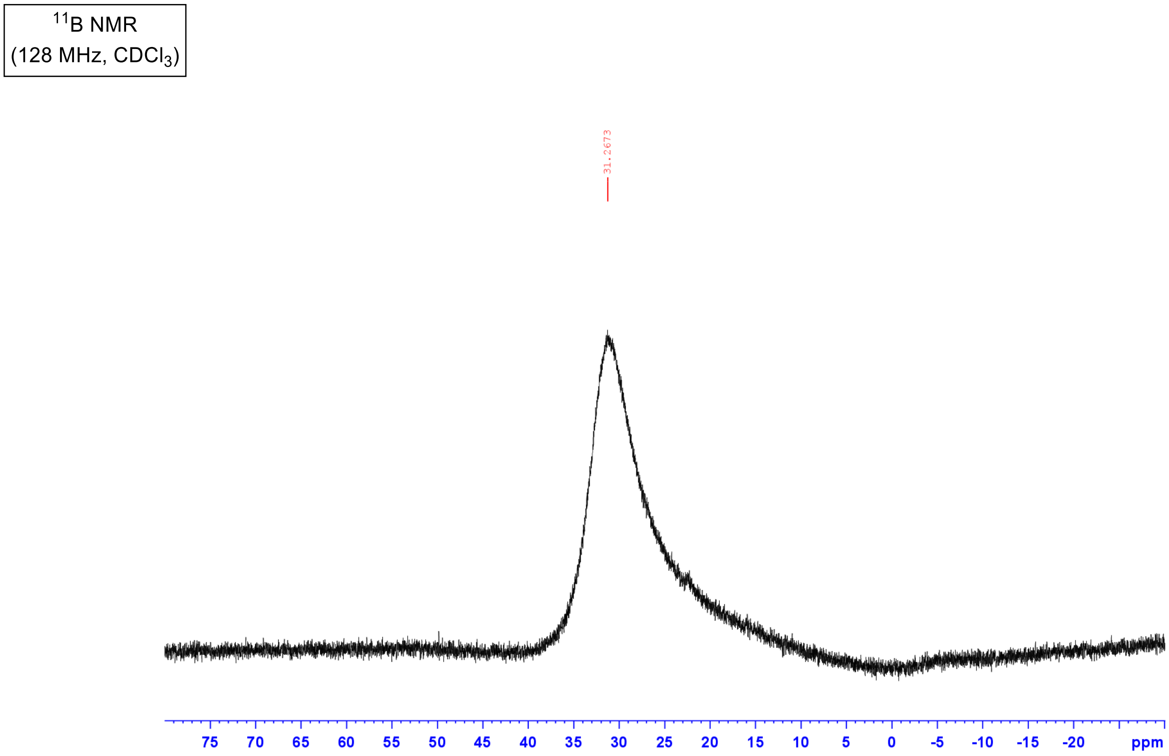

$^{11}$B NMR
(128 MHz, CDCl$_3$)
31.2673
75 70 65 60 55 50 45 40 35 30 25 20 15 10 5 0 -5 -10 -15 -20 ppm

[$^1$H, $^{13}$C{$^1$H}, and $^{11}$B NMR Spectra of ***anti*-3ta**]

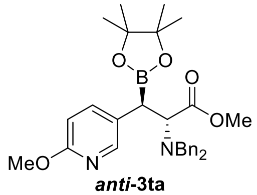


***anti*-3ta**

$^1$H NMR
(400 MHz, $CDCl_3$)

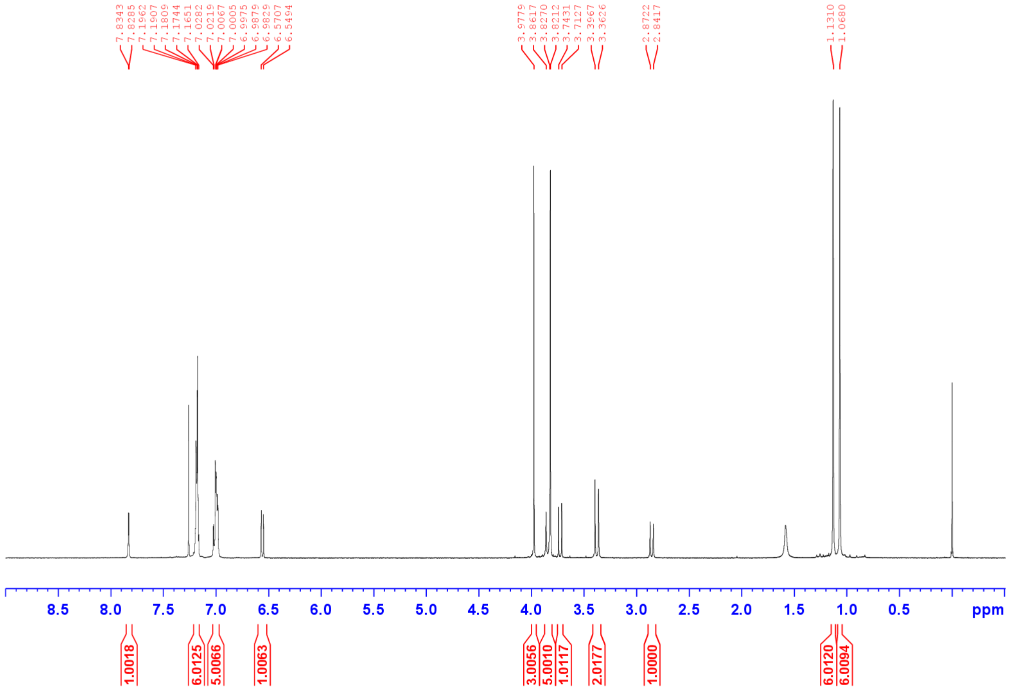


$^{13}$C{$^1$H} NMR
(100 MHz, $CDCl_3$)

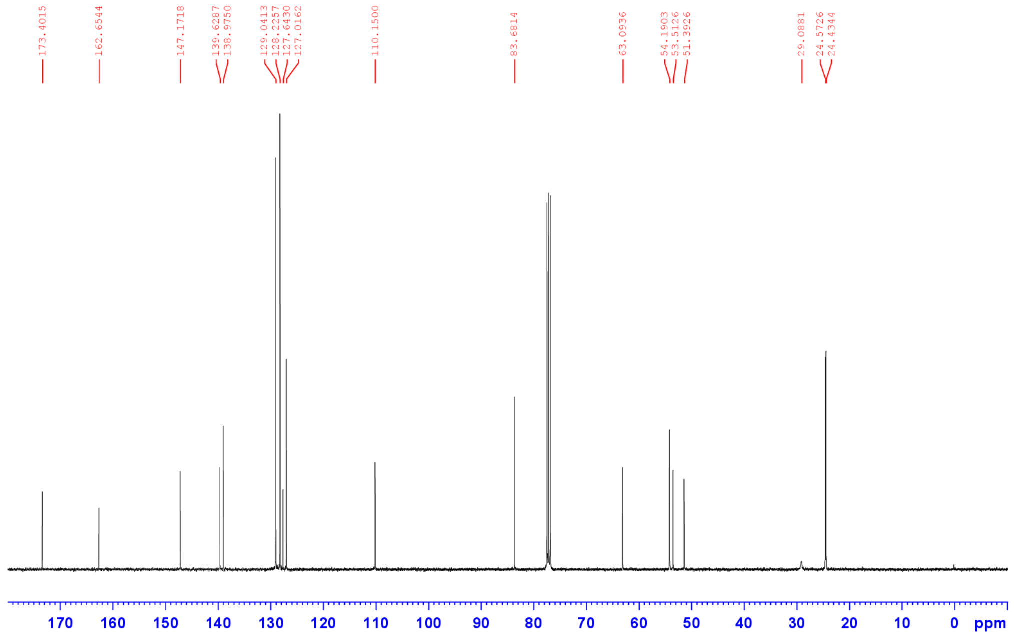

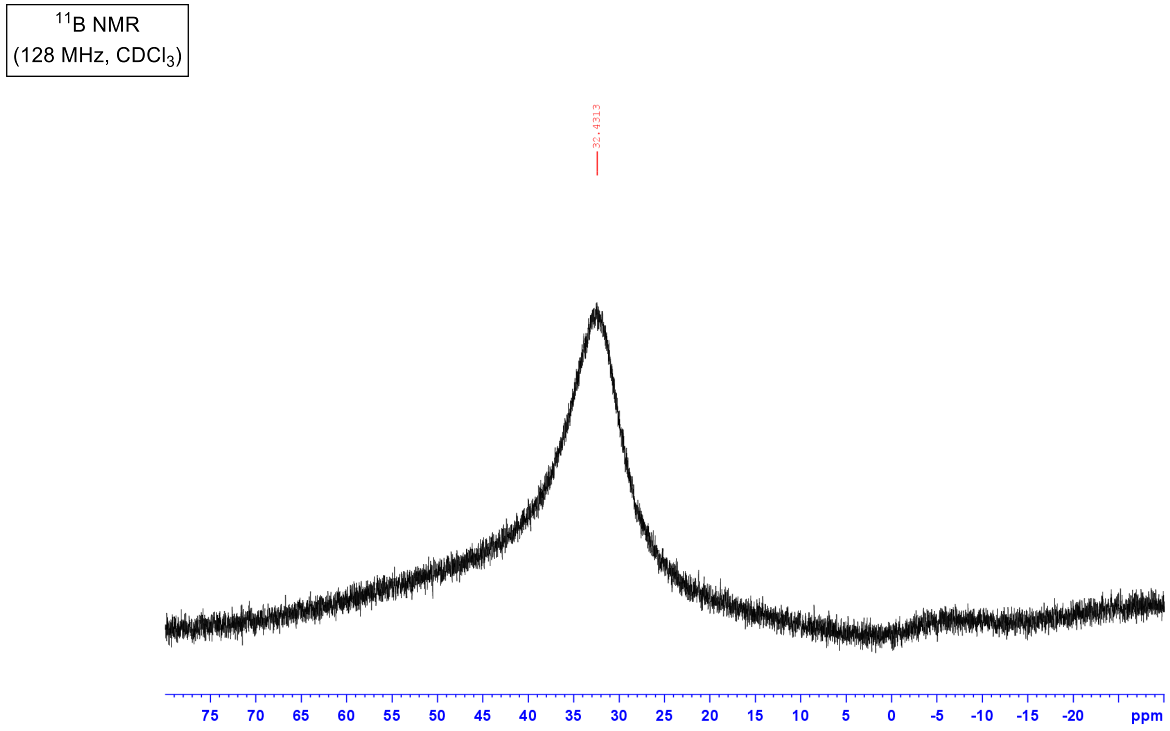
$^{11}B$ NMR
(128 MHz, $CDCl_3$)
32.4313
75
70
65
60
55
50
45
40
35
30
25
20
15
10
5
0
-5
-10
-15
-20
ppm

[$^{1}$H, $^{13}$C{$^{1}$H}, and $^{11}$B NMR Spectra of **3ua**]

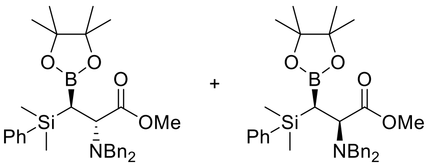


***anti*-3ua** **+** ***syn*-3ua**

$^{1}$H NMR
(400 MHz, $CDCl_3$)

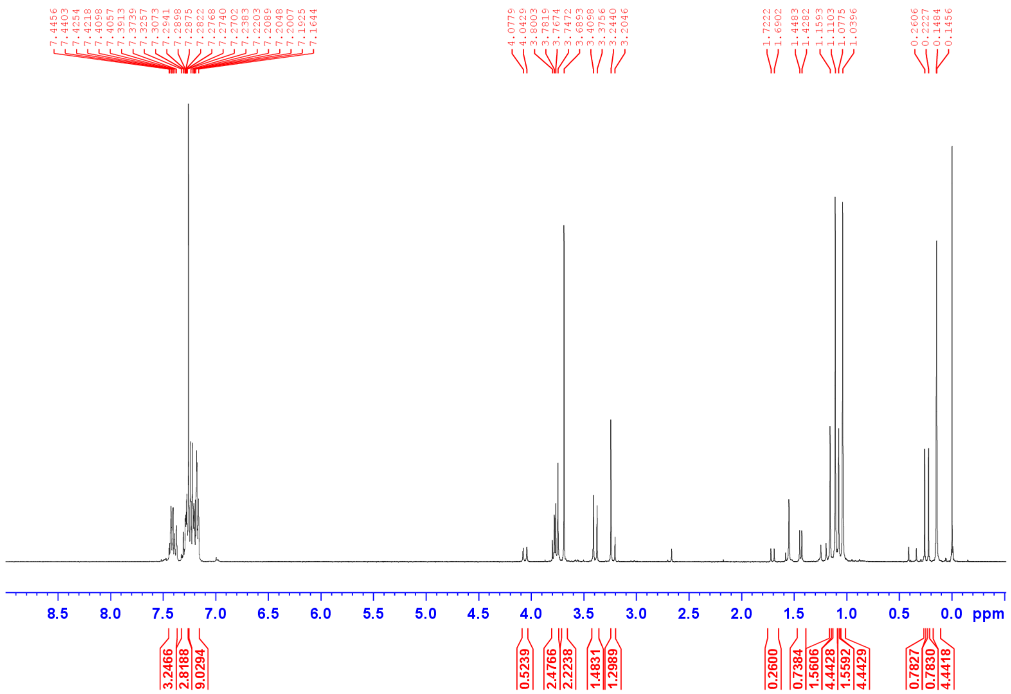


$^{13}$C{$^{1}$H} NMR
(100 MHz, $CDCl_3$)

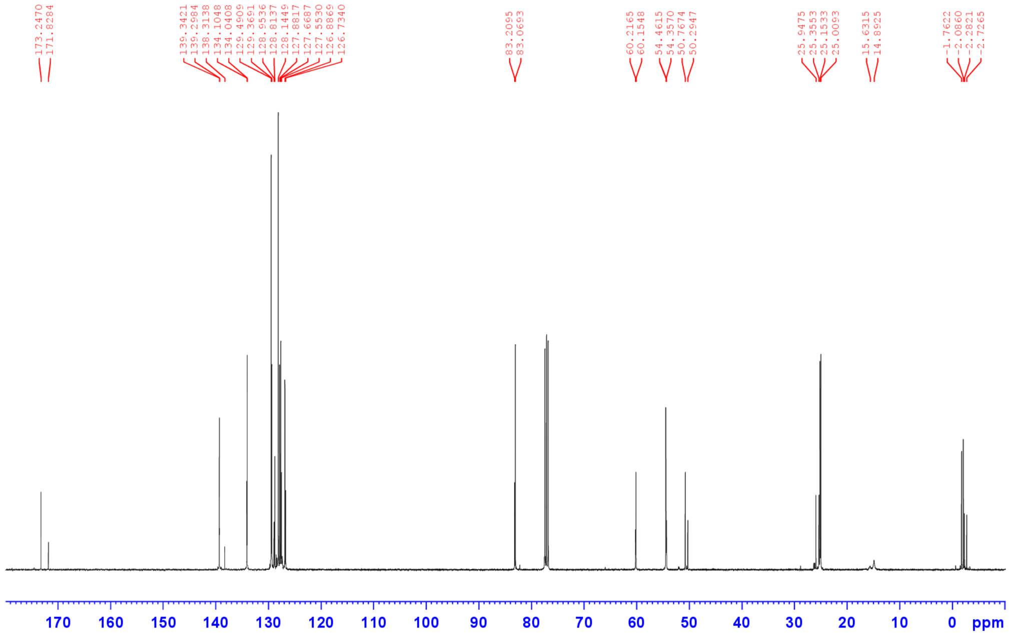

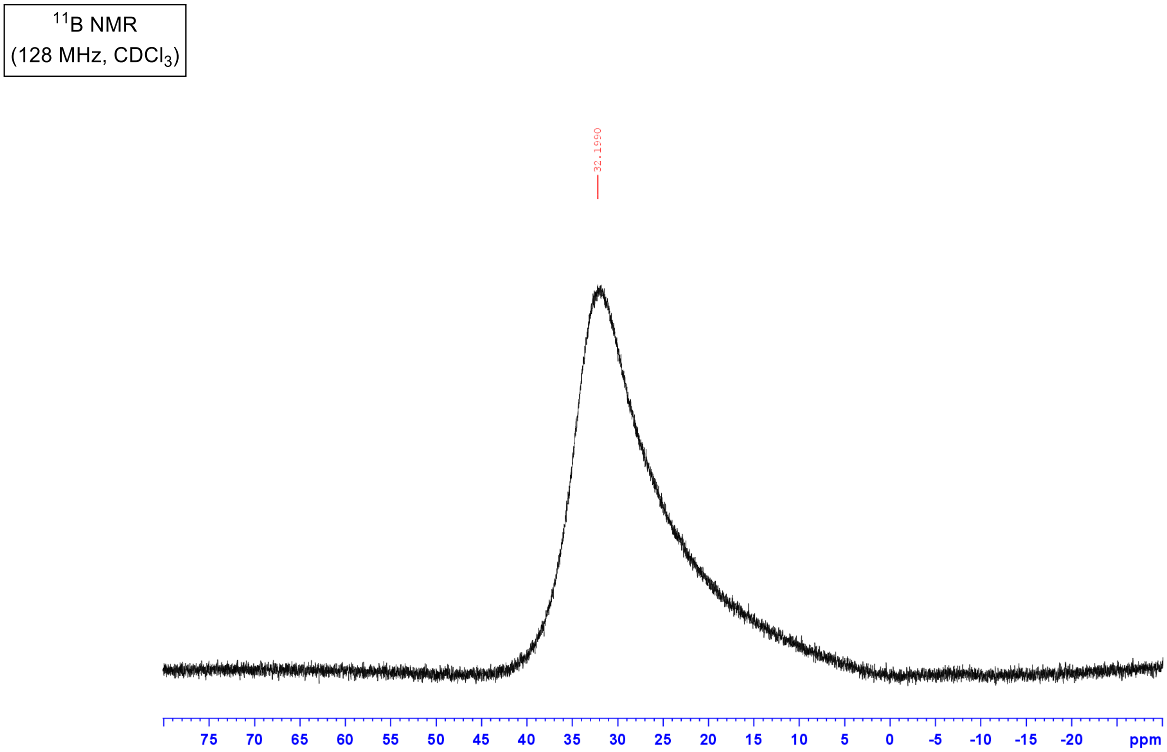

11B NMR
(128 MHz, CDCl3)
32.1990
75 70 65 60 55 50 45 40 35 30 25 20 15 10 5 0 -5 -10 -15 -20 ppm

[$^{1}H$, $^{13}C\{^{1}H\}$, and $^{11}B$ NMR Spectra of ***anti*-3va**]

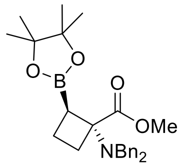


***anti*-3va**

$^{1}H$ NMR
(400 MHz, $CDCl_3$)

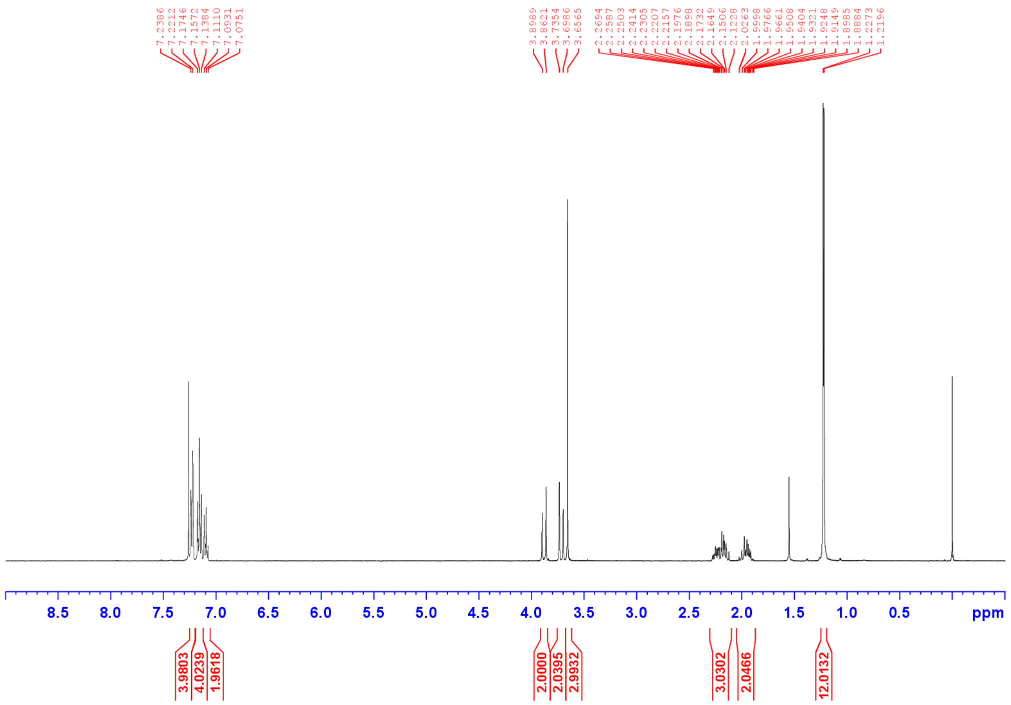


$^{13}C\{^{1}H\}$ NMR
(100 MHz, $CDCl_3$)

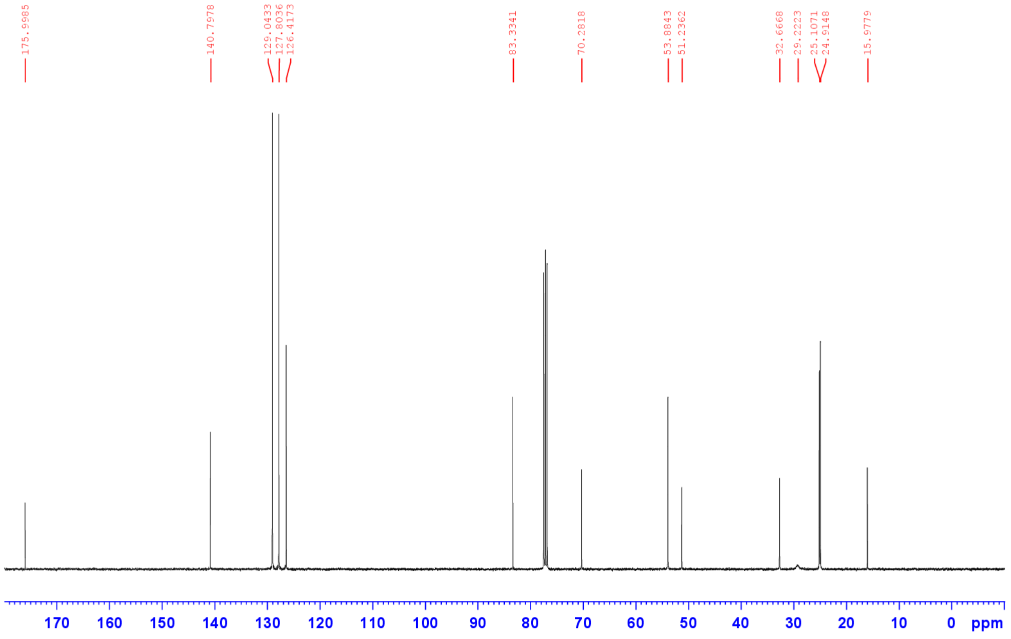

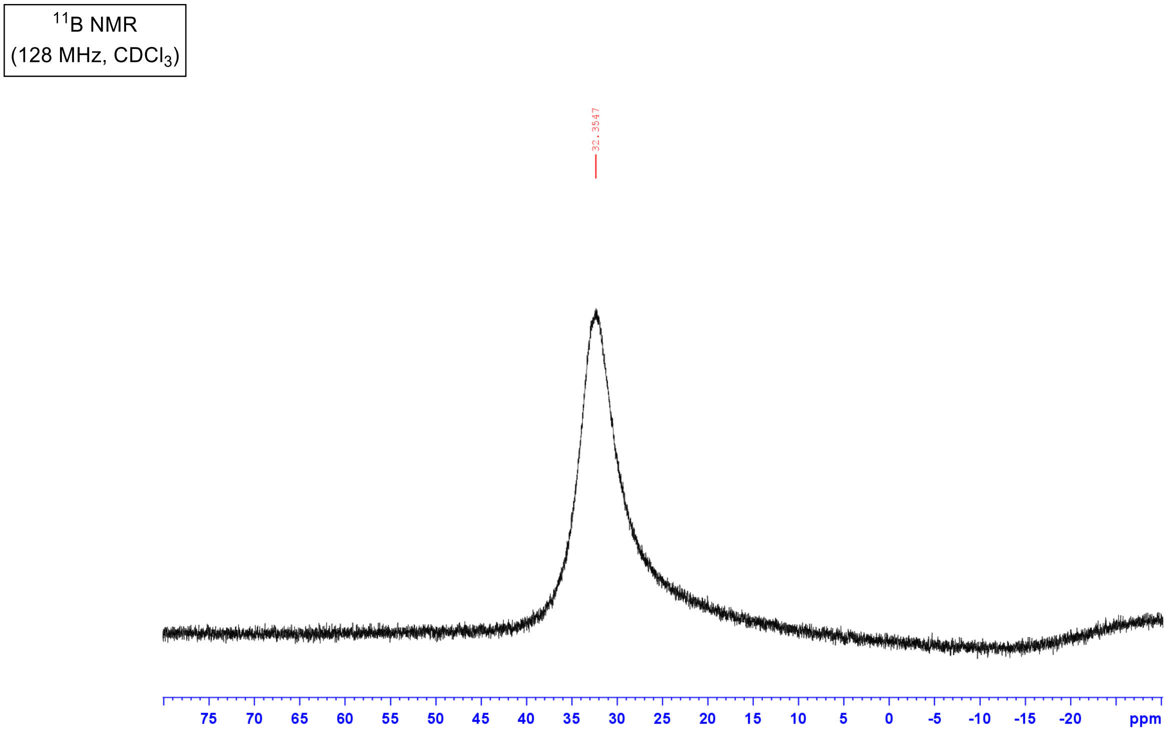

$^{11}$B NMR
(128 MHz, CDCl$_3$)
32.3547
75 70 65 60 55 50 45 40 35 30 25 20 15 10 5 0 -5 -10 -15 -20 ppm

[$^{1}H$, $^{13}C\{^{1}H\}$, and $^{11}B$ NMR Spectra of ***anti*-3wa**]

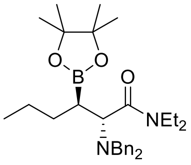


***anti*-3wa**

$^{1}H$ NMR
(400 MHz, $CDCl_3$)

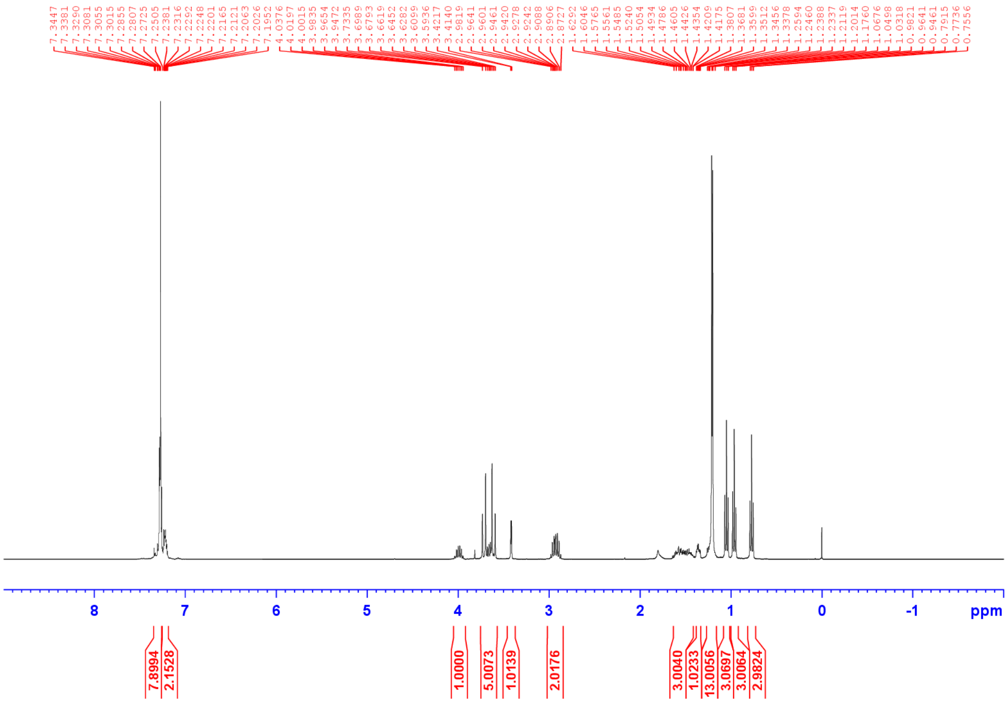


$^{13}C\{^{1}H\}$ NMR
(100 MHz, $CDCl_3$)

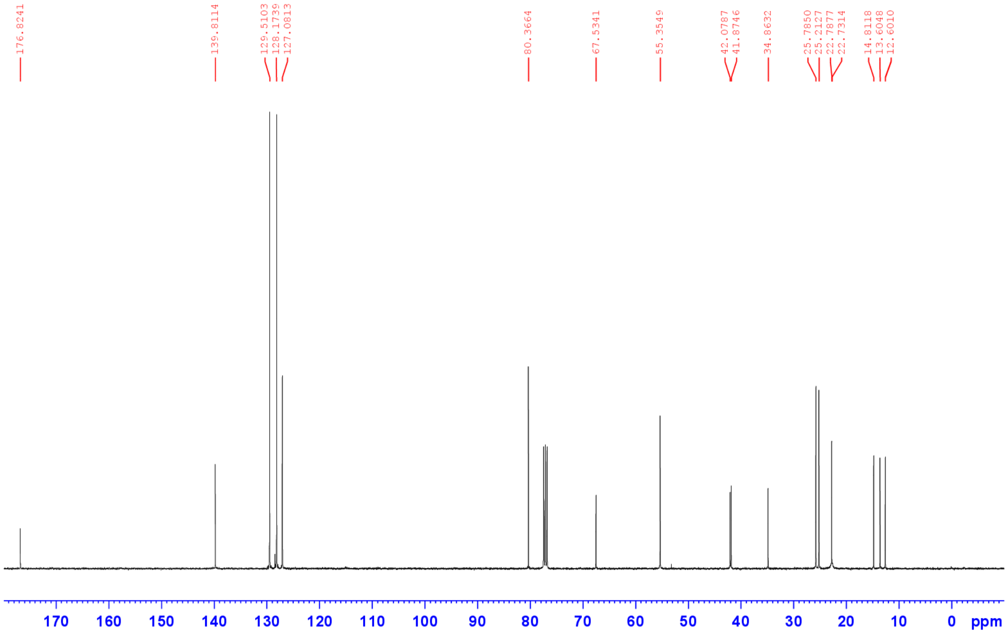

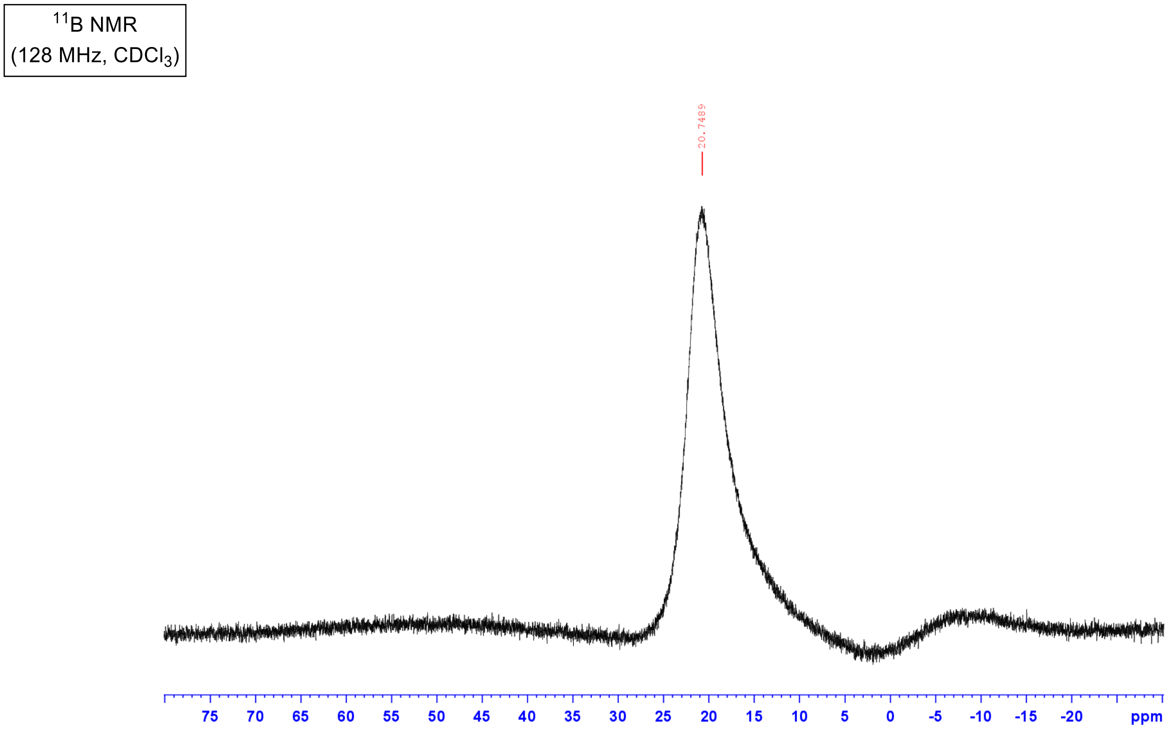
11B NMR
(128 MHz, CDCl3)
20.7489
75 70 65 60 55 50 45 40 35 30 25 20 15 10 5 0 -5 -10 -15 -20 ppm

[$^{1}$H, $^{13}$C{$^{1}$H}, and $^{11}$B NMR Spectra of **3ab**]

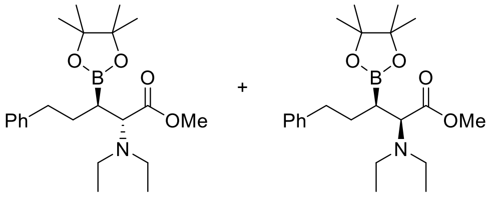


***anti*-3ab** **syn-3ab**

$^{1}$H NMR
(400 MHz, $CDCl_3$)

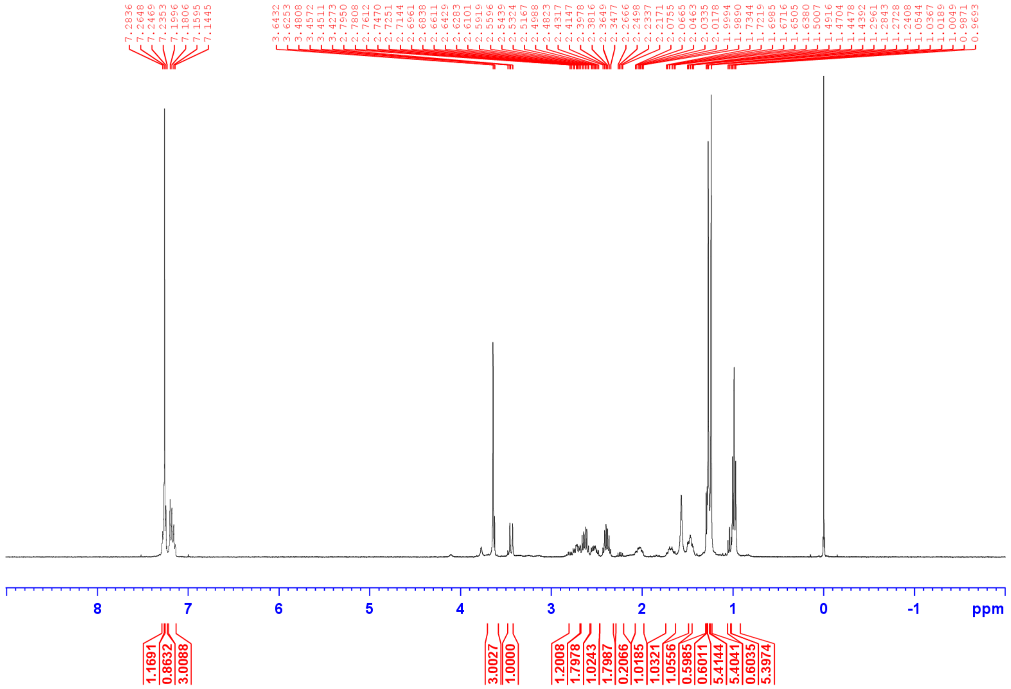


$^{13}$C{$^{1}$H} NMR
(100 MHz, $CDCl_3$)

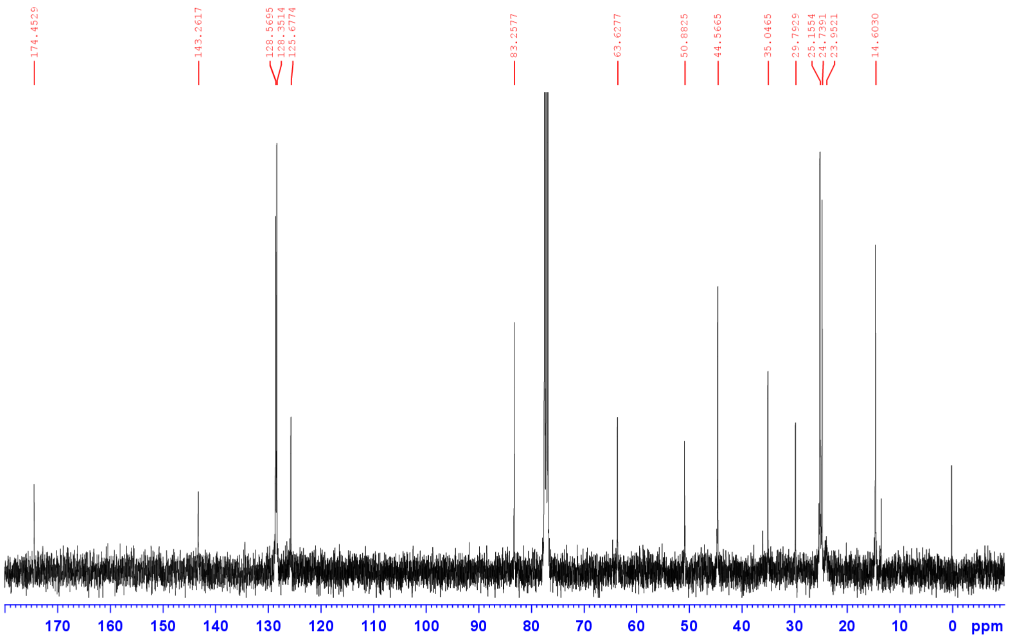

$^{11}B$ NMR
(128 MHz, $CDCl_3$)

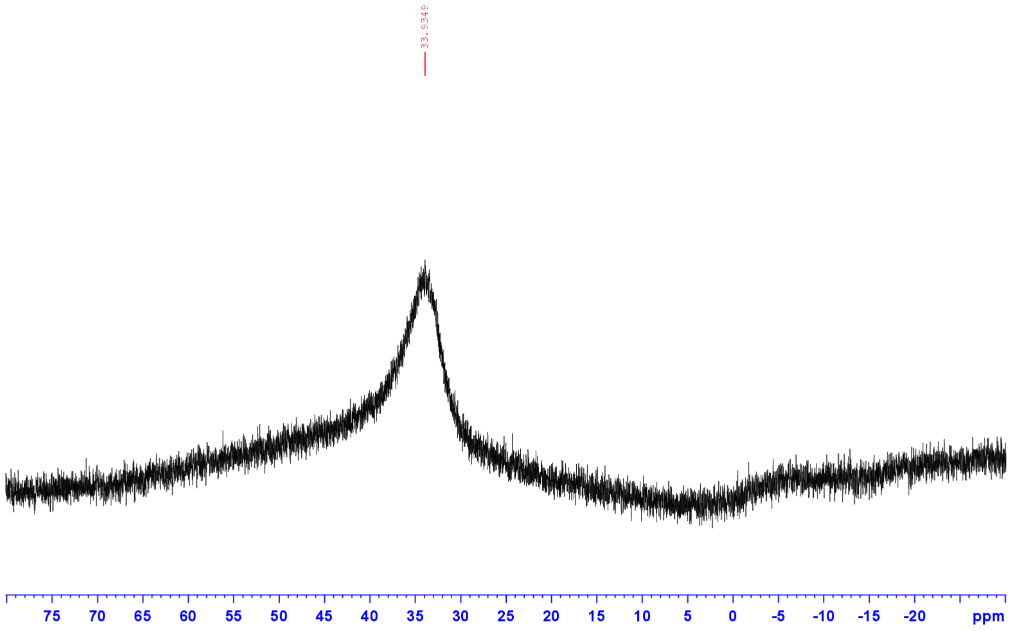

[$^{1}$H, $^{13}$C{$^{1}$H}, and $^{11}$B NMR Spectra of **3ac**]

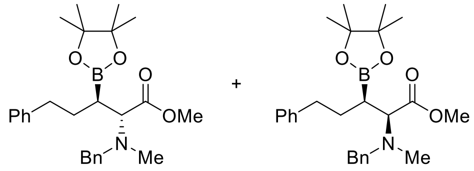


***anti*-3ac** ***syn*-3ac**

$^{1}$H NMR
(400 MHz, $CDCl_3$)

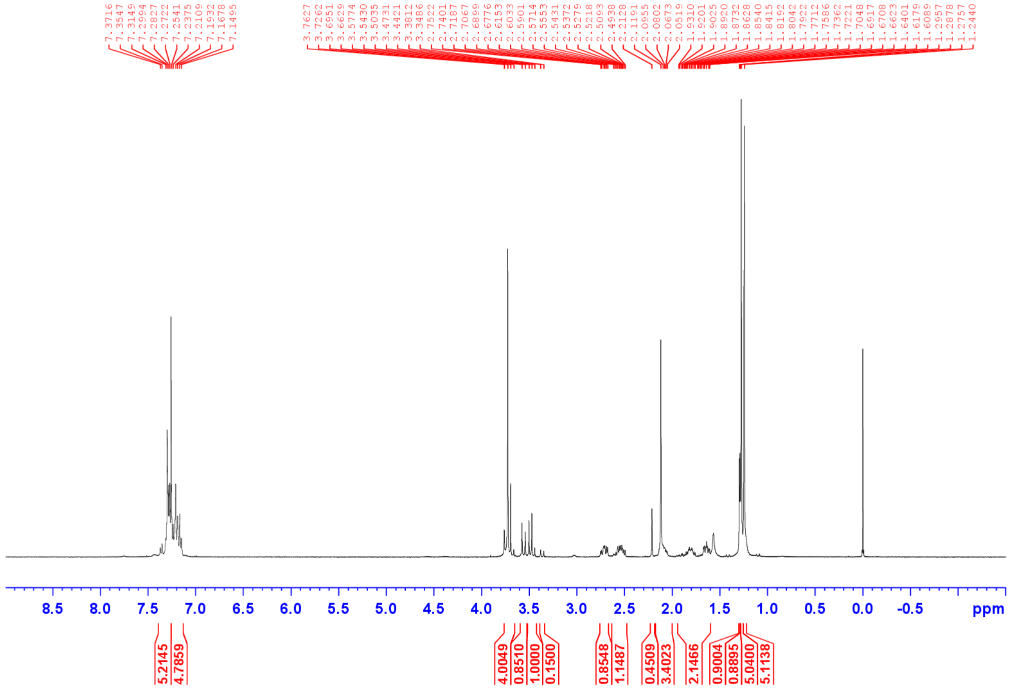


$^{13}$C{$^{1}$H} NMR
(100 MHz, $CDCl_3$)

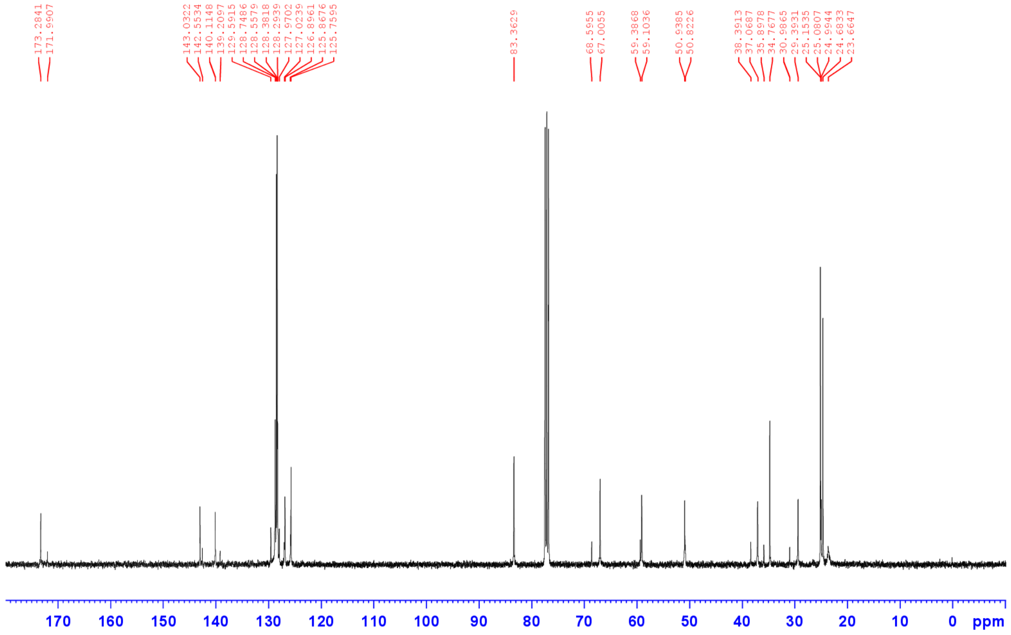

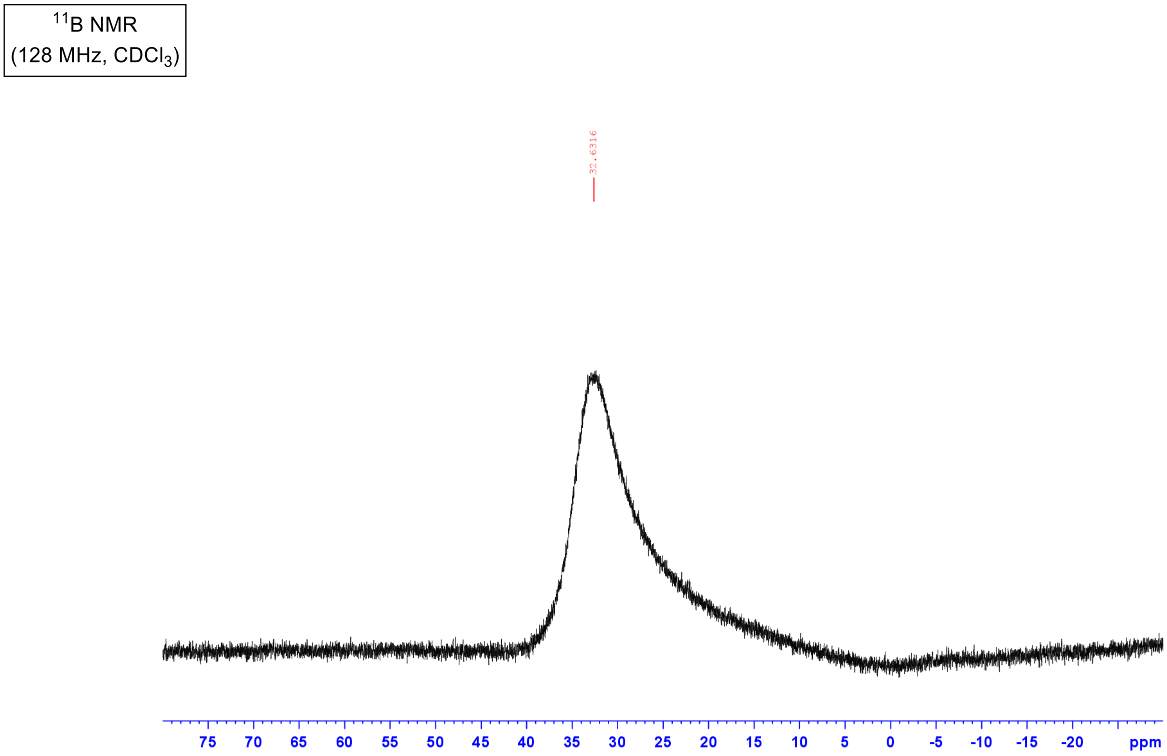

$^{11}B$ NMR
(128 MHz, $CDCl_3$)
32.6316
75 70 65 60 55 50 45 40 35 30 25 20 15 10 5 0 -5 -10 -15 -20 ppm

[$^{1}$H, $^{13}$C{$^{1}$H}, and $^{11}$B NMR Spectra of ***anti*-3ad**]

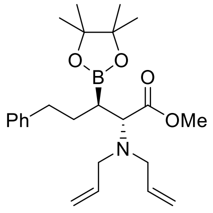


***anti*-3ad**

$^{1}$H NMR
(400 MHz, $CDCl_3$)

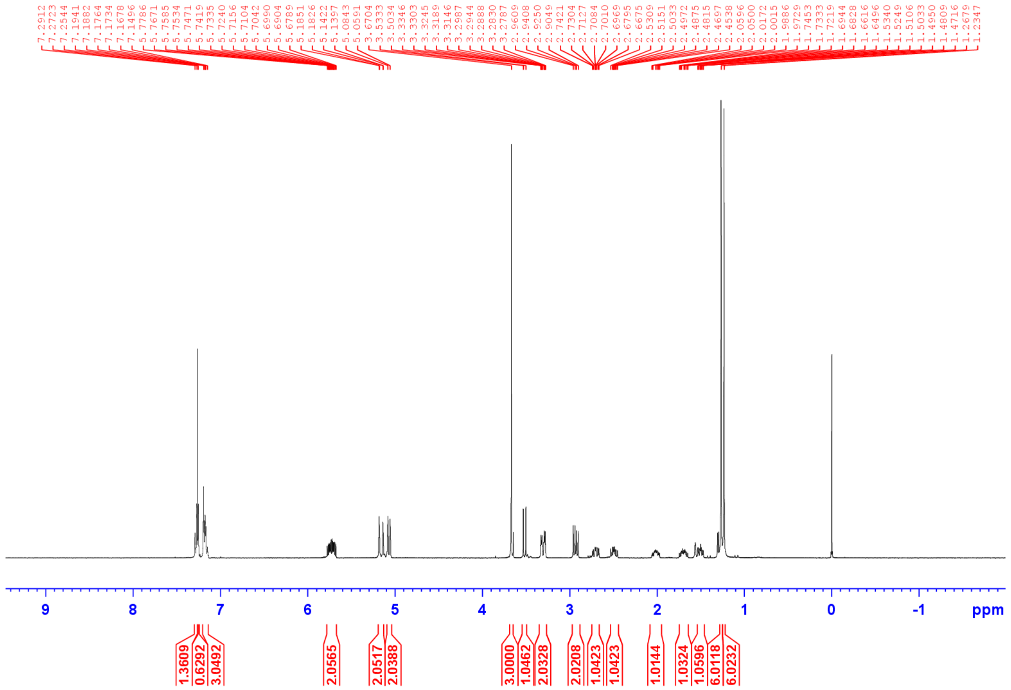


$^{13}$C{$^{1}$H} NMR
(100 MHz, $CDCl_3$)

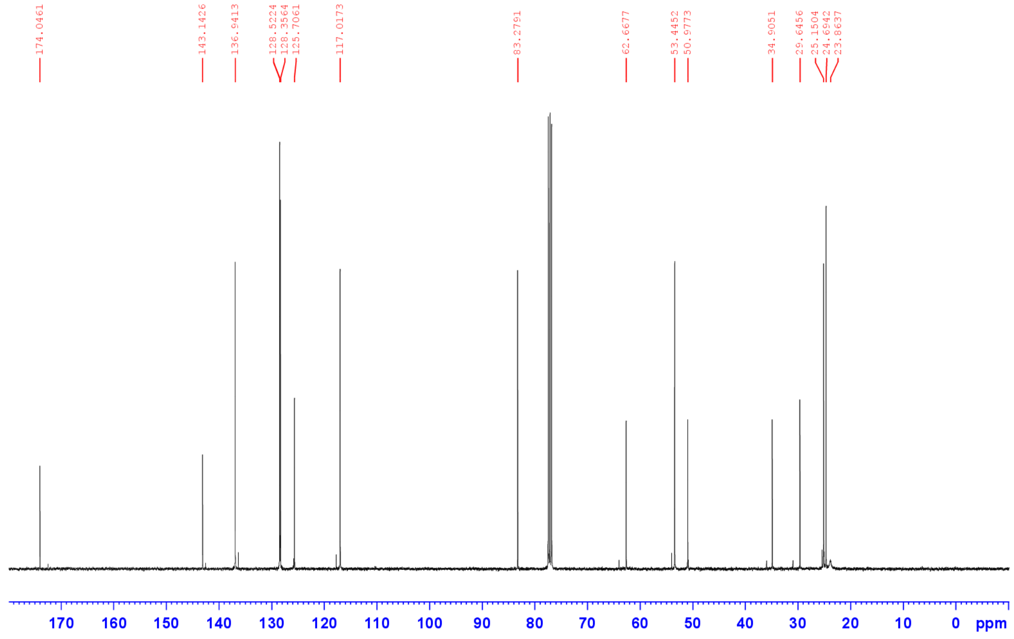

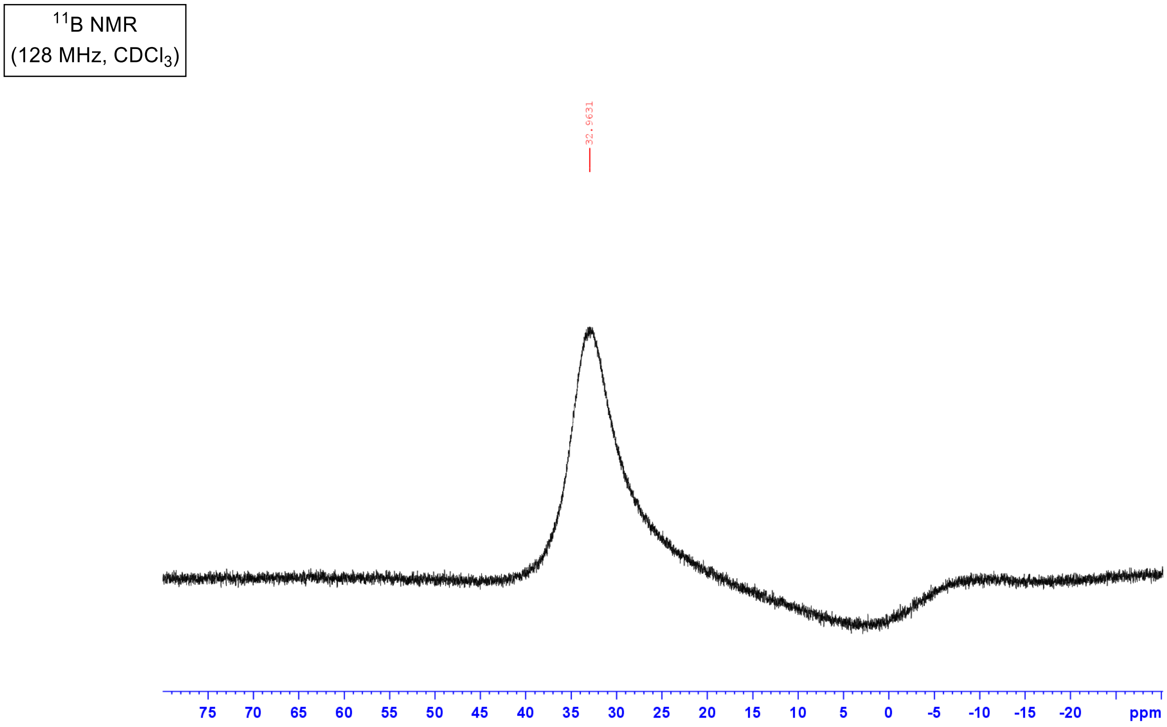

$^{11}$B NMR
(128 MHz, CDCl$_3$)
32.9631
75
70
65
60
55
50
45
40
35
30
25
20
15
10
5
0
-5
-10
-15
-20
ppm

[$^{1}$H, $^{13}$C{$^{1}$H}, and $^{11}$B NMR Spectra of **3ae**]

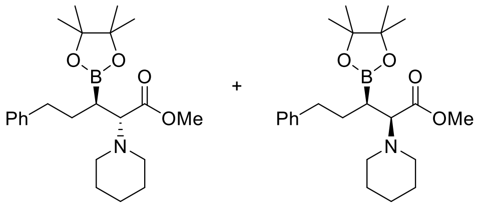

***anti*-3ae** + ***syn*-3ae**

$^{1}$H NMR (400 MHz, $CDCl_3$)

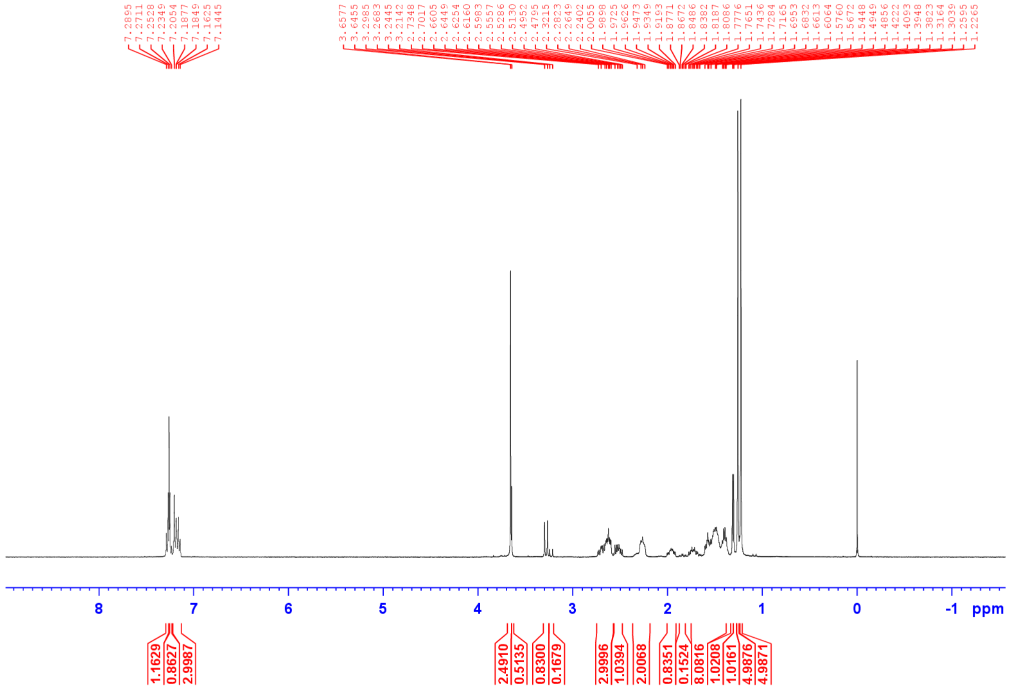

$^{13}$C{$^{1}$H} NMR (100 MHz, $CDCl_3$)

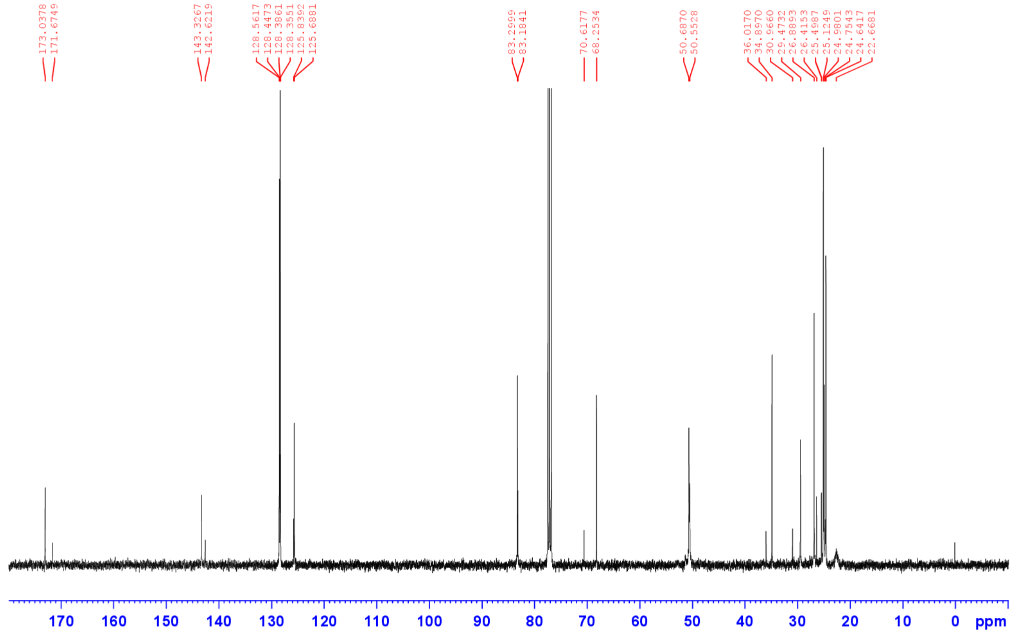

$^{11}B$ NMR
(128 MHz, $CDCl_3$)

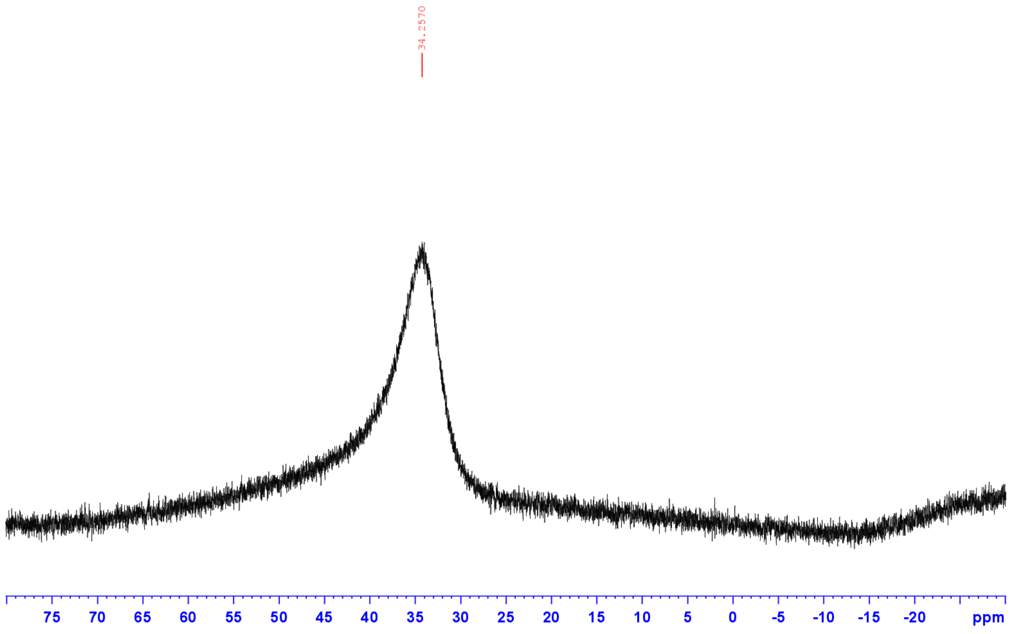

[$^{1}$H, $^{13}$C{$^{1}$H}, and $^{11}$B NMR Spectra of **3af**]

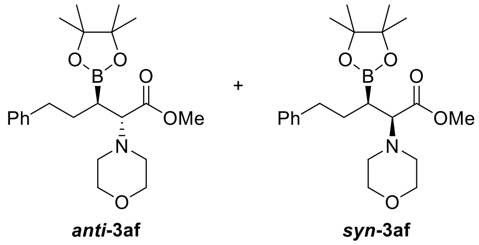


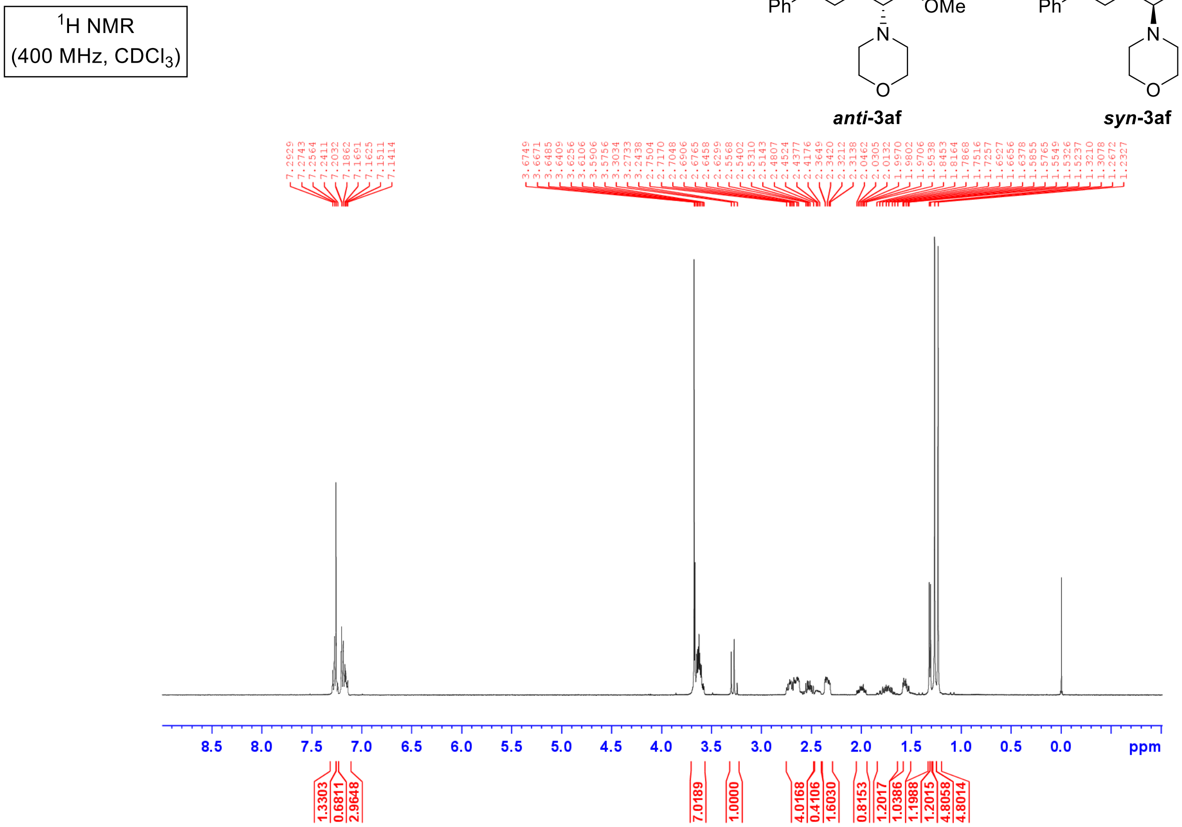


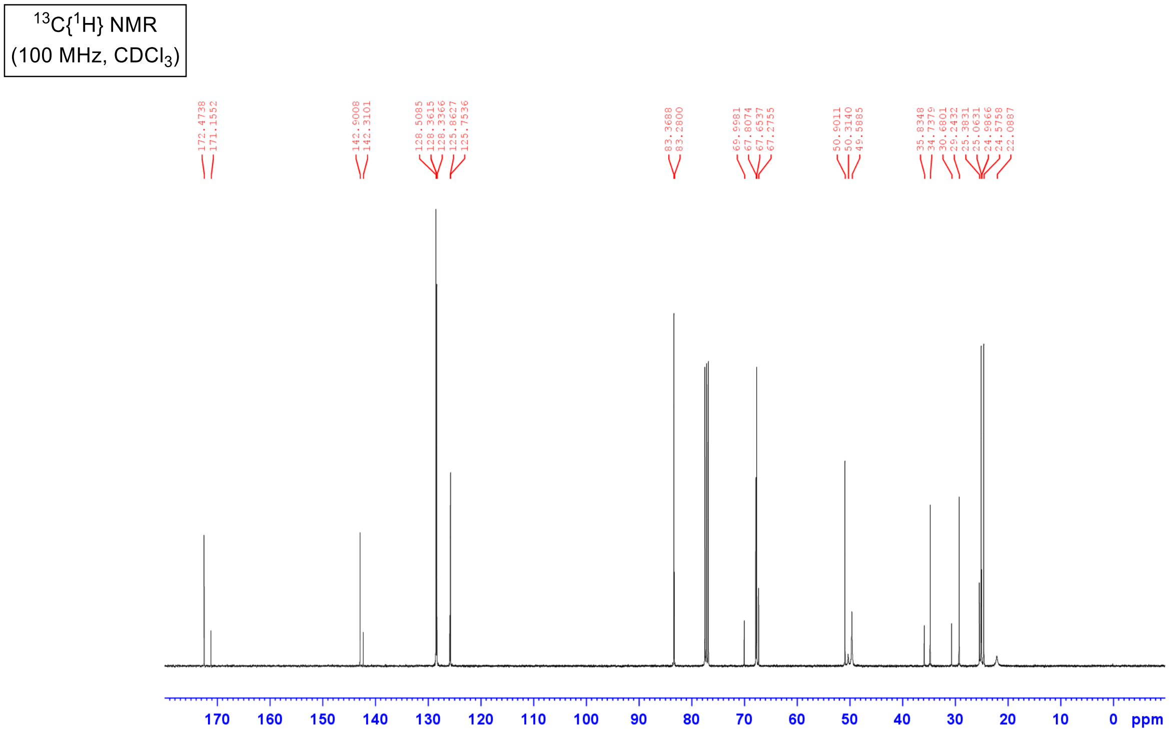

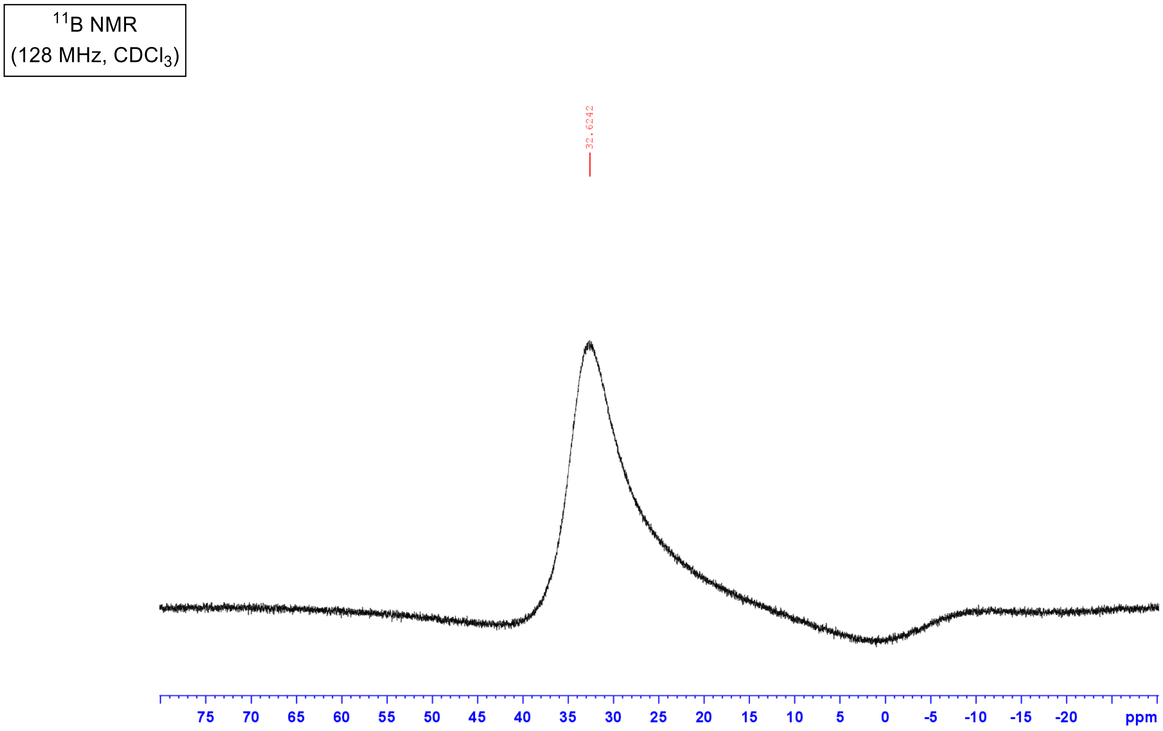

$^{11}$B NMR
(128 MHz, $CDCl_3$)
32.6242
75 70 65 60 55 50 45 40 35 30 25 20 15 10 5 0 -5 -10 -15 -20 ppm

[$^{1}H$, $^{13}C\{^{1}H\}$, and $^{11}B$ NMR Spectra of **3ag**]

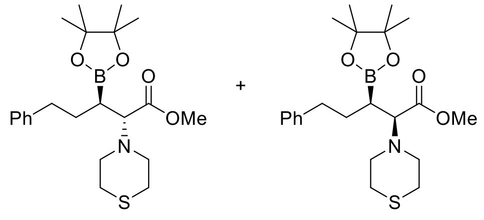

***anti*-3ag** **_syn_-3ag**

$^{1}H$ NMR
(400 MHz, $CDCl_3$)

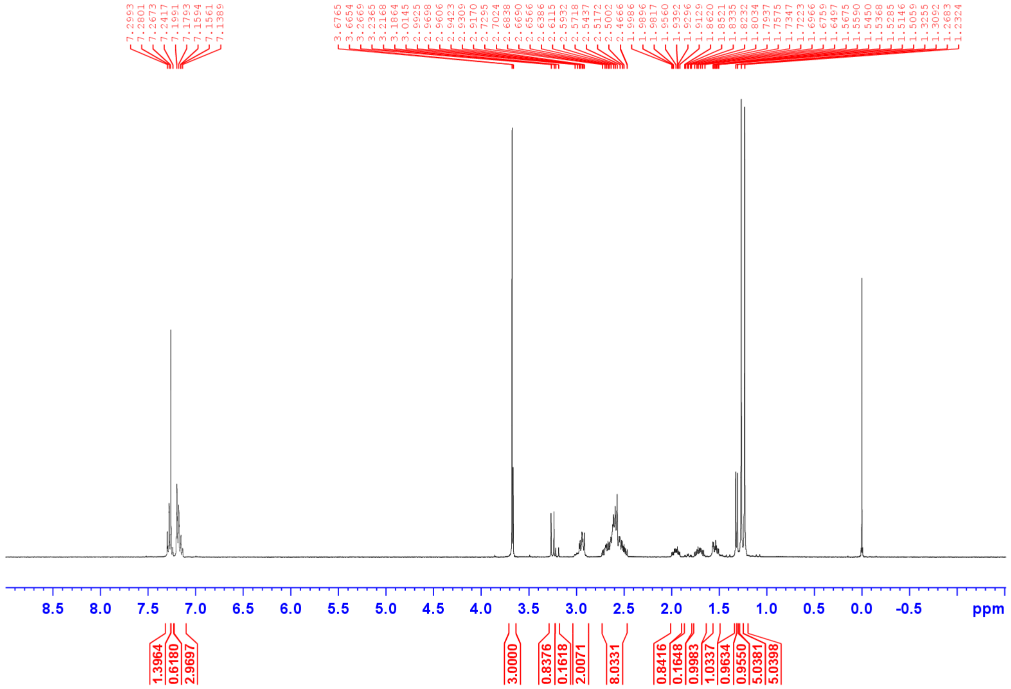


$^{13}C\{^{1}H\}$ NMR
(100 MHz, $CDCl_3$)

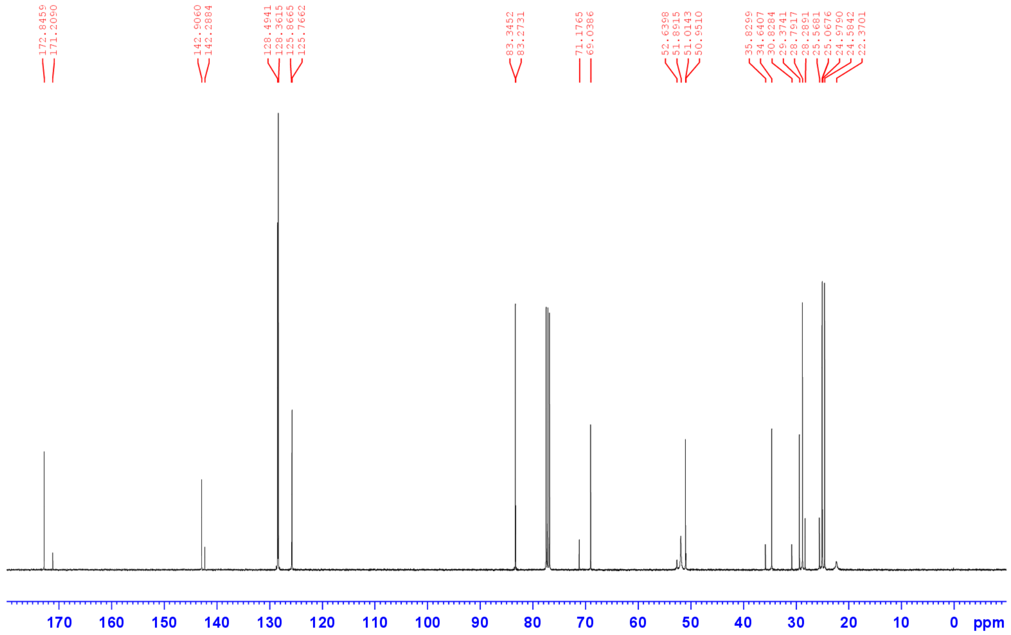

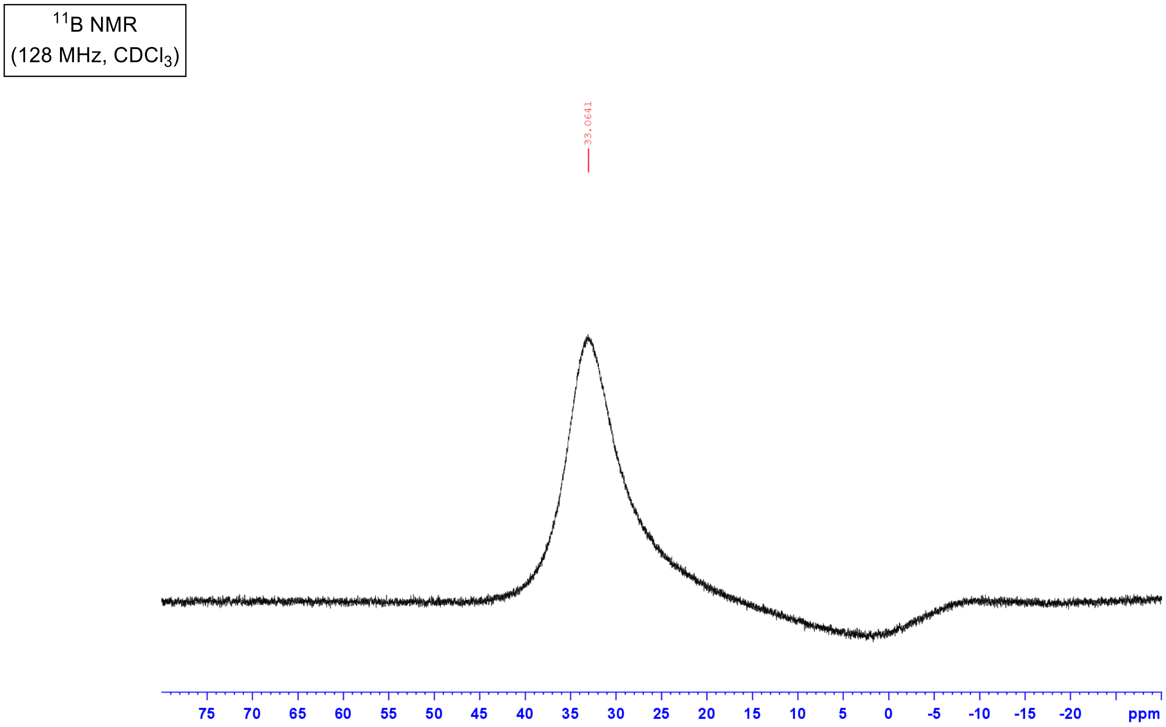

$^{11}B$ NMR
(128 MHz, $CDCl_3$)
33.0641
75 70 65 60 55 50 45 40 35 30 25 20 15 10 5 0 -5 -10 -15 -20 ppm

[$^{1}H$, $^{13}C\{^{1}H\}$, and $^{11}B$ NMR Spectra of **3Ae**]

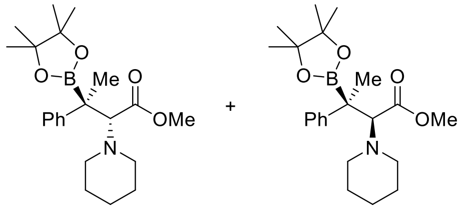


*anti*-3Ae   *syn*-3Ae

$^{1}H$ NMR
(400 MHz, $CDCl_3$)

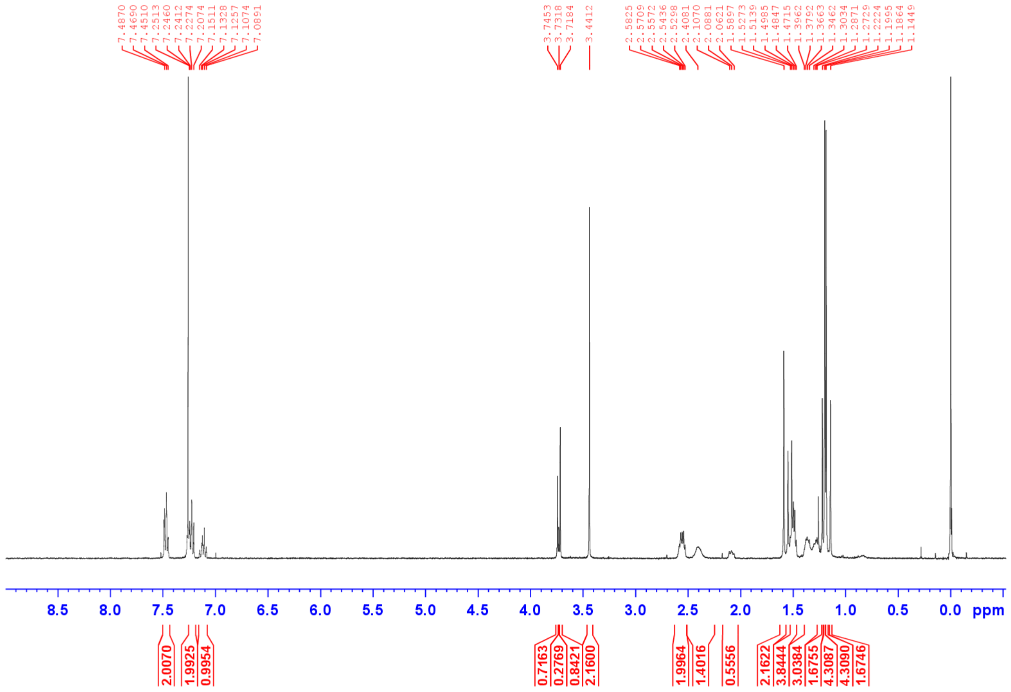

$^{13}C\{^{1}H\}$ NMR
(100 MHz, $CDCl_3$)

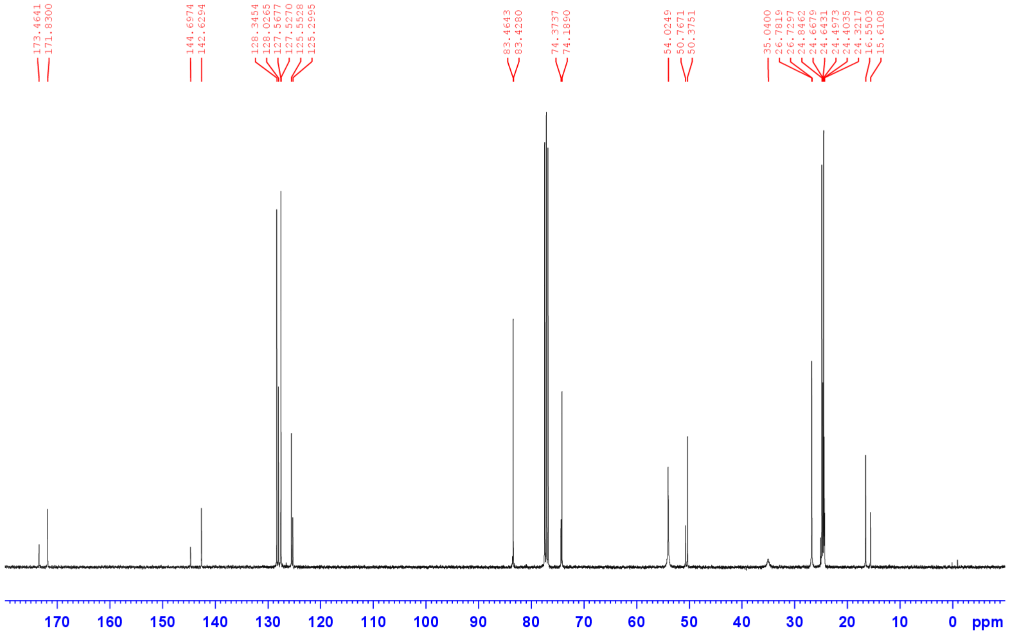

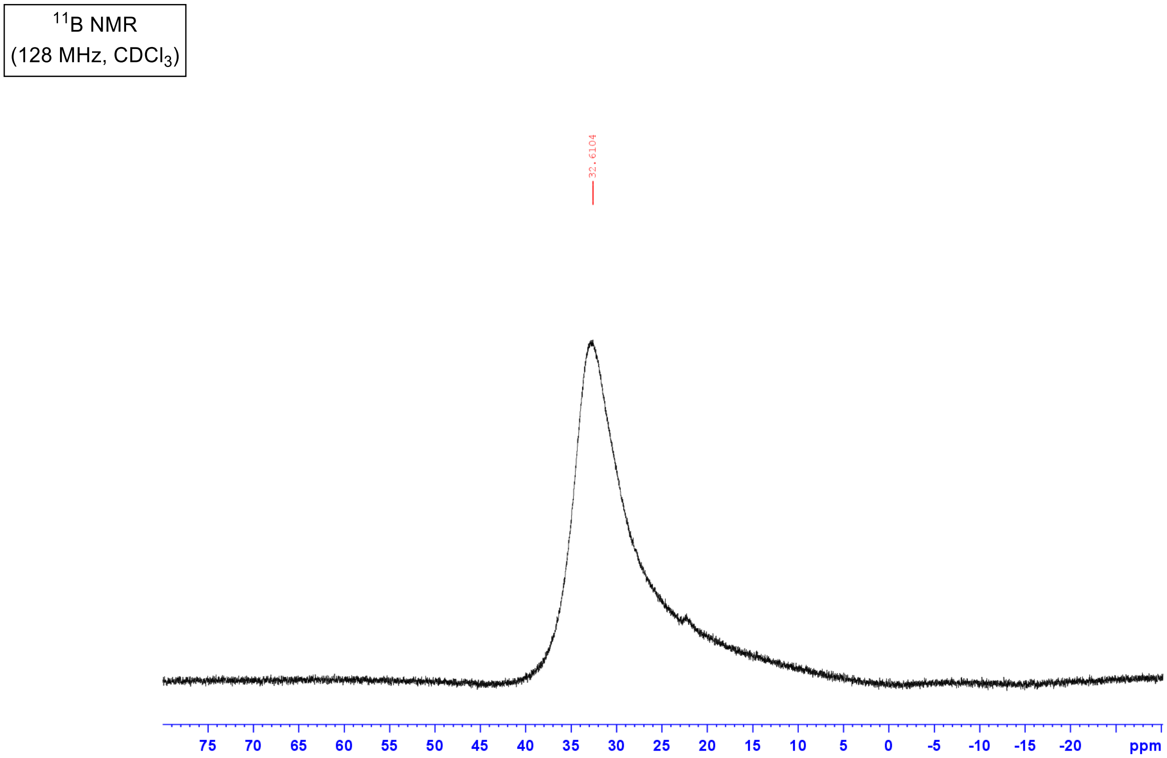
$^{11}$B NMR
(128 MHz, CDCl$_3$)
32.6104
75 70 65 60 55 50 45 40 35 30 25 20 15 10 5 0 -5 -10 -15 -20 ppm

[$^1$H, $^{13}$C{$^1$H}, and $^{11}$B NMR Spectra of **3Be**]

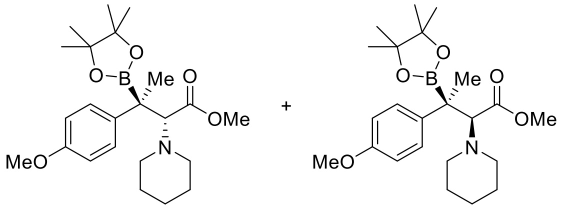

*anti*-3Be + *syn*-3Be

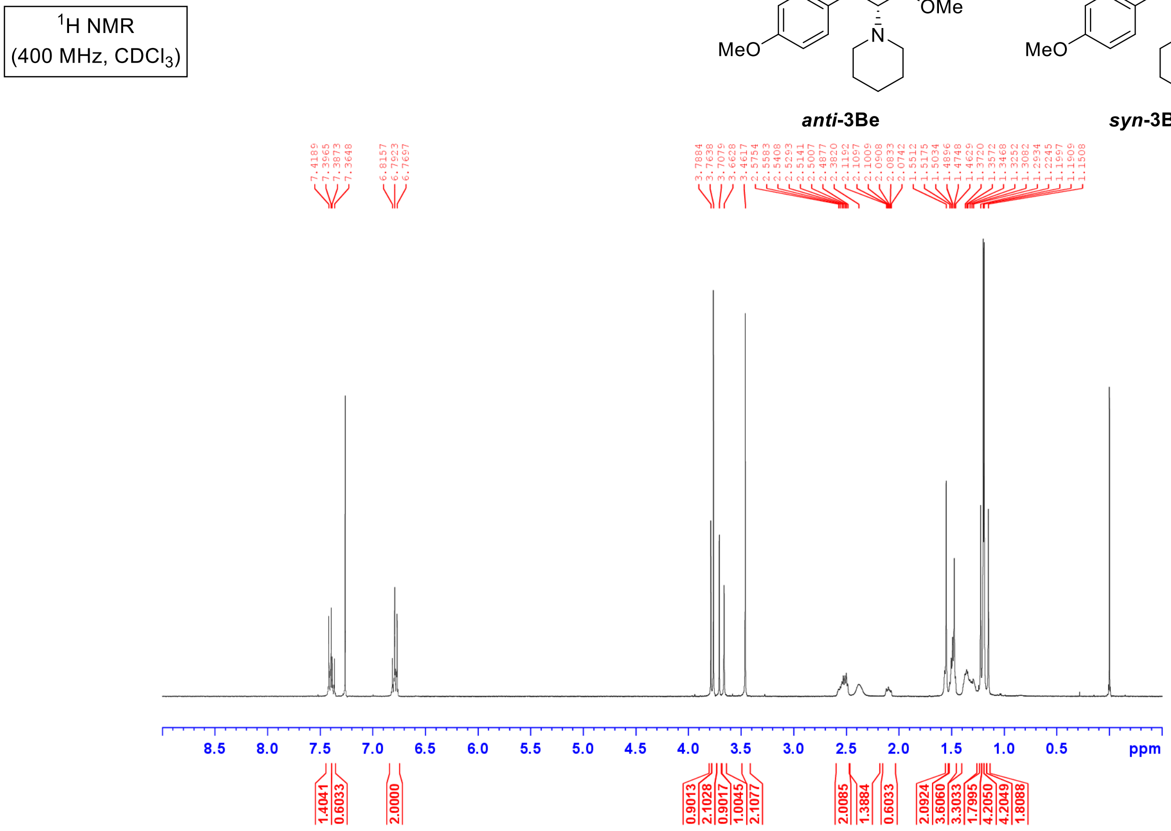


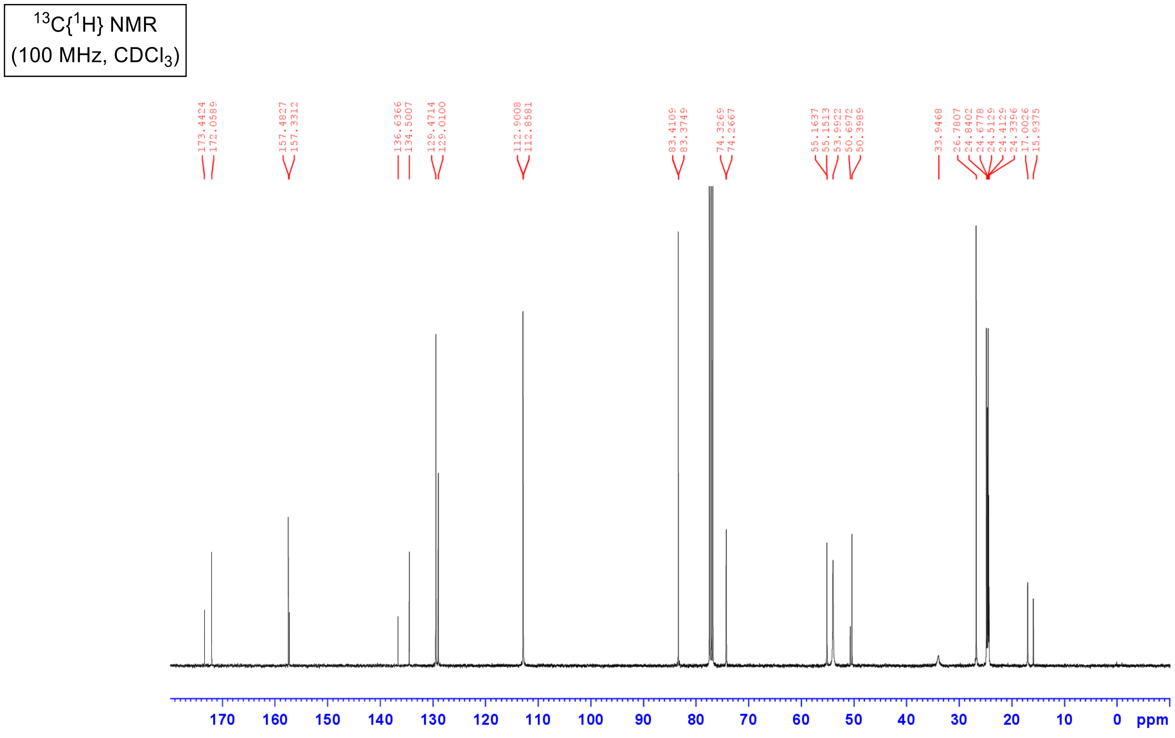

$^{11}$B NMR
(128 MHz, $CDCl_3$)

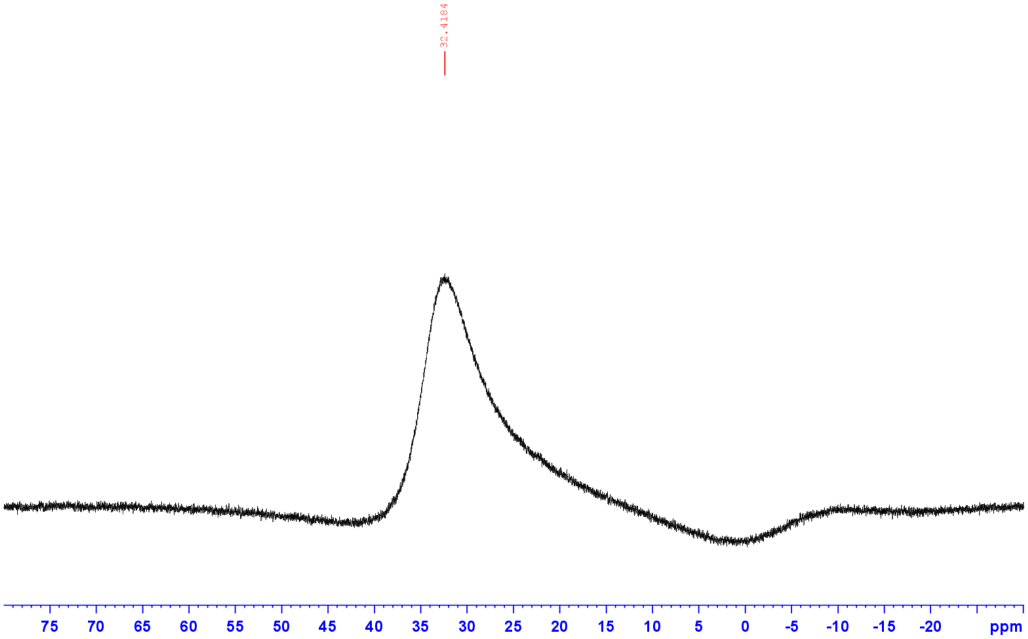

[$^{1}H$, $^{13}C\{^{1}H\}$, and $^{11}B$ NMR Spectra of **3Ce**]

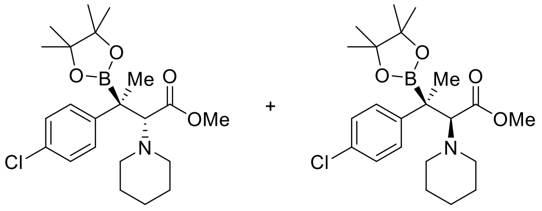


***anti*-3Ce** ***syn*-3Ce**

$^{1}H$ NMR
(400 MHz, $CDCl_3$)

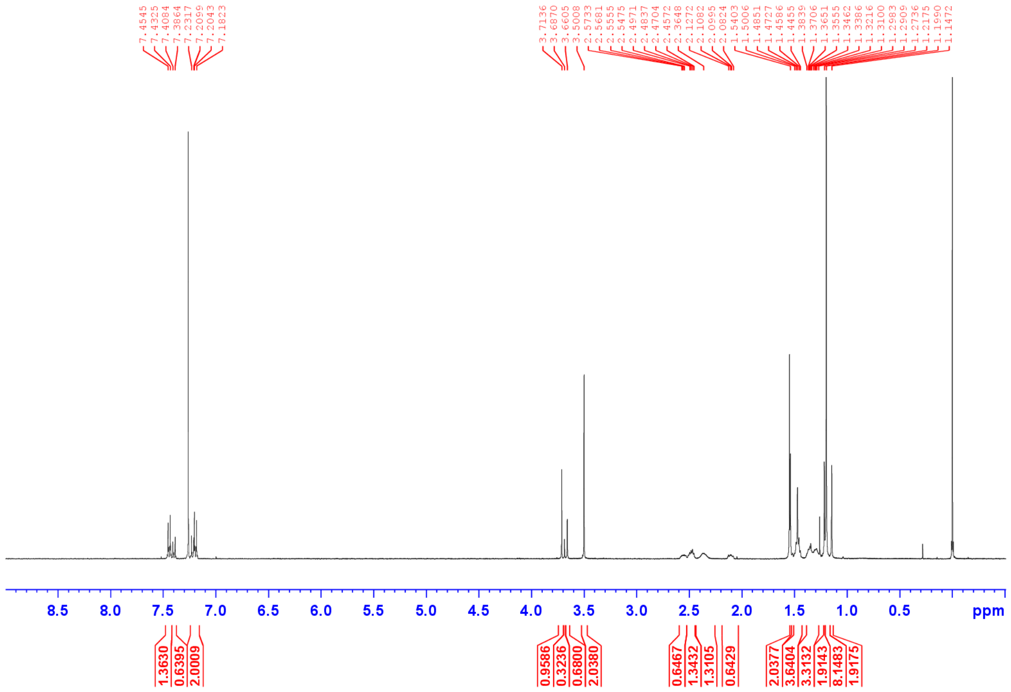


$^{13}C\{^{1}H\}$ NMR
(100 MHz, $CDCl_3$)

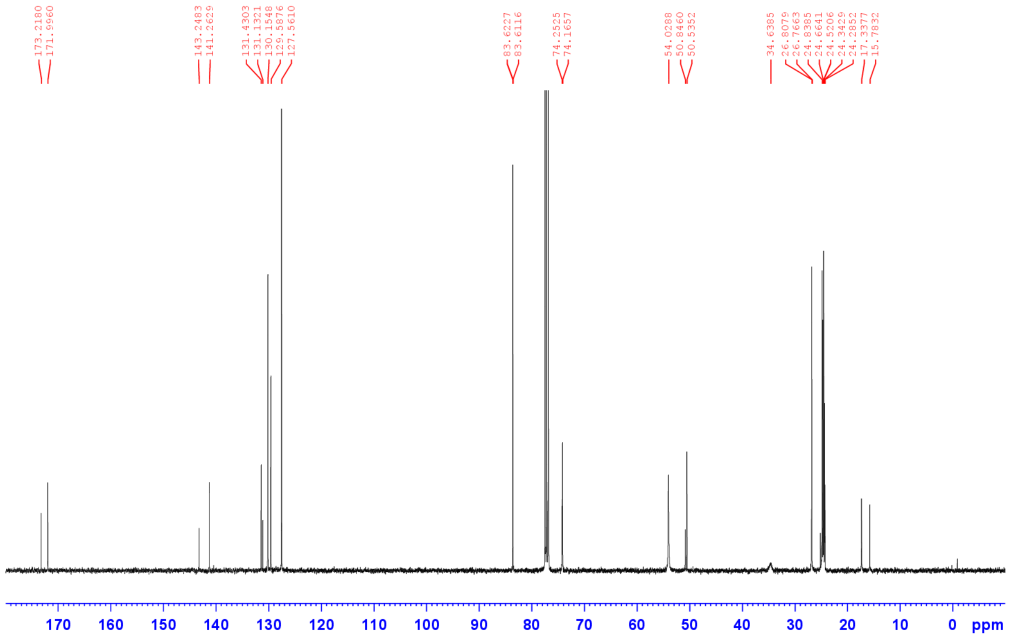

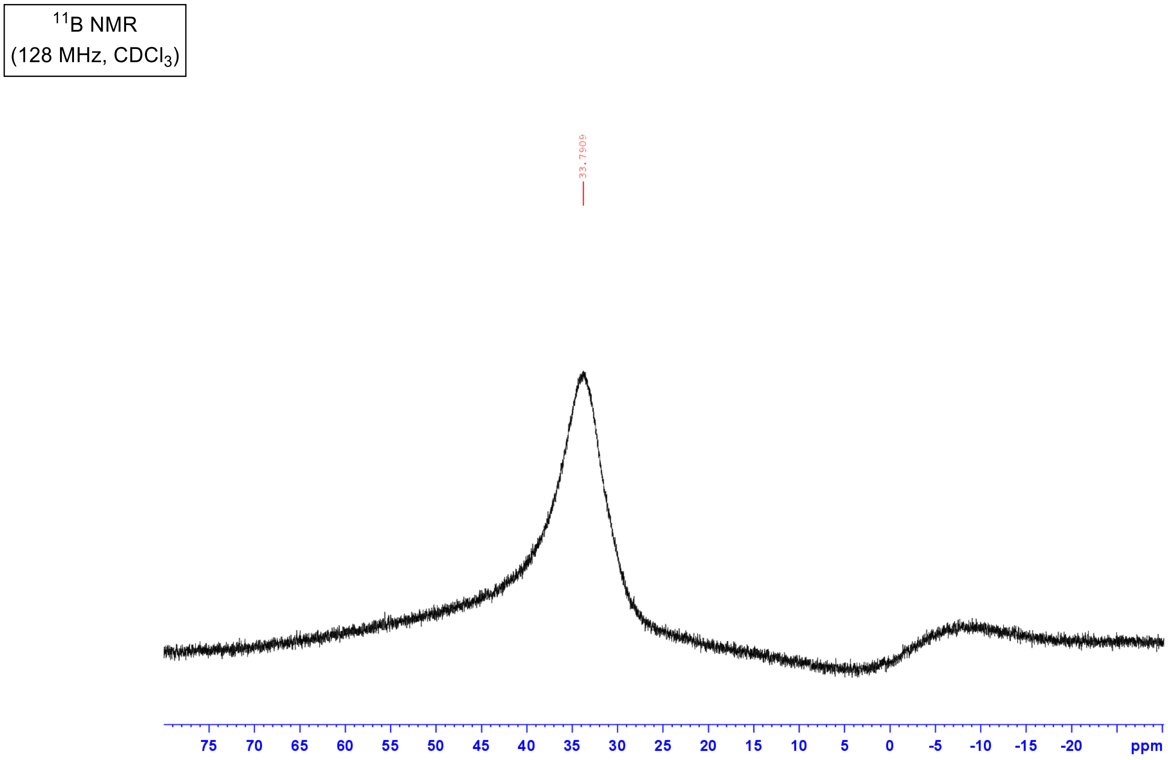

$^{11}$B NMR
(128 MHz, $CDCl_3$)
33.7909
75 70 65 60 55 50 45 40 35 30 25 20 15 10 5 0 -5 -10 -15 -20 ppm

[$^{1}H$, $^{13}C\{^{1}H\}$, and $^{11}B$ NMR Spectra of **3De**]

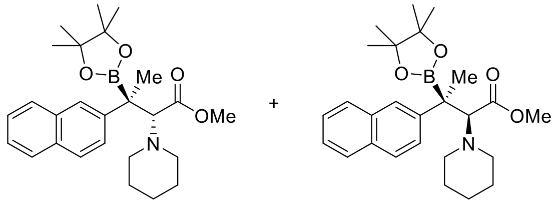

***anti*-3De** **syn-3De**

$^{1}H$ NMR
(400 MHz, $CDCl_3$)

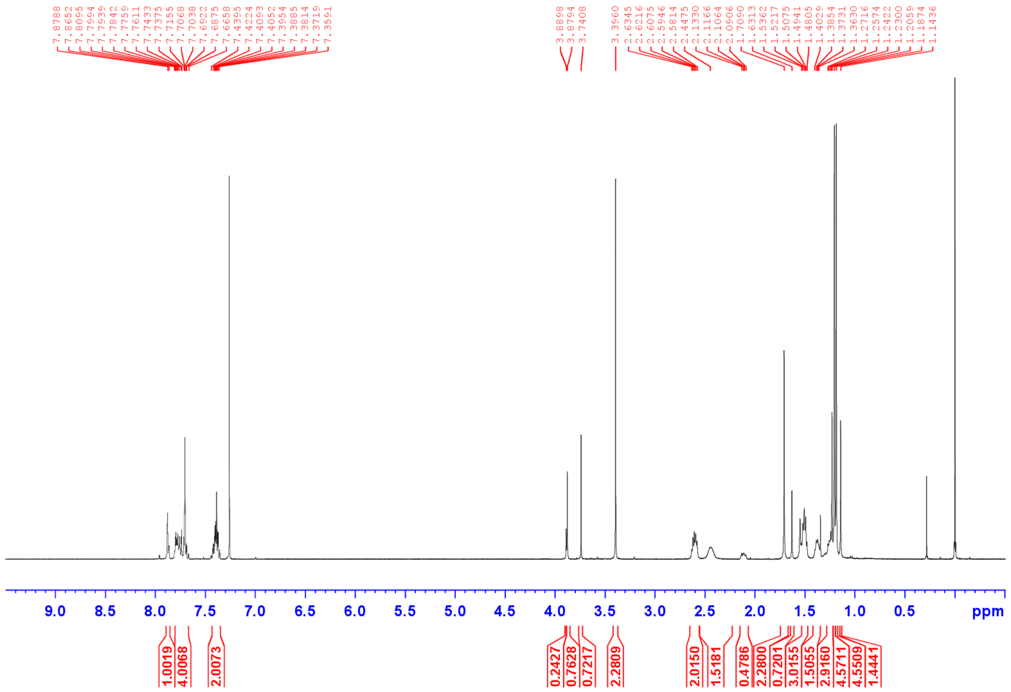

$^{13}C\{^{1}H\}$ NMR
(100 MHz, $CDCl_3$)

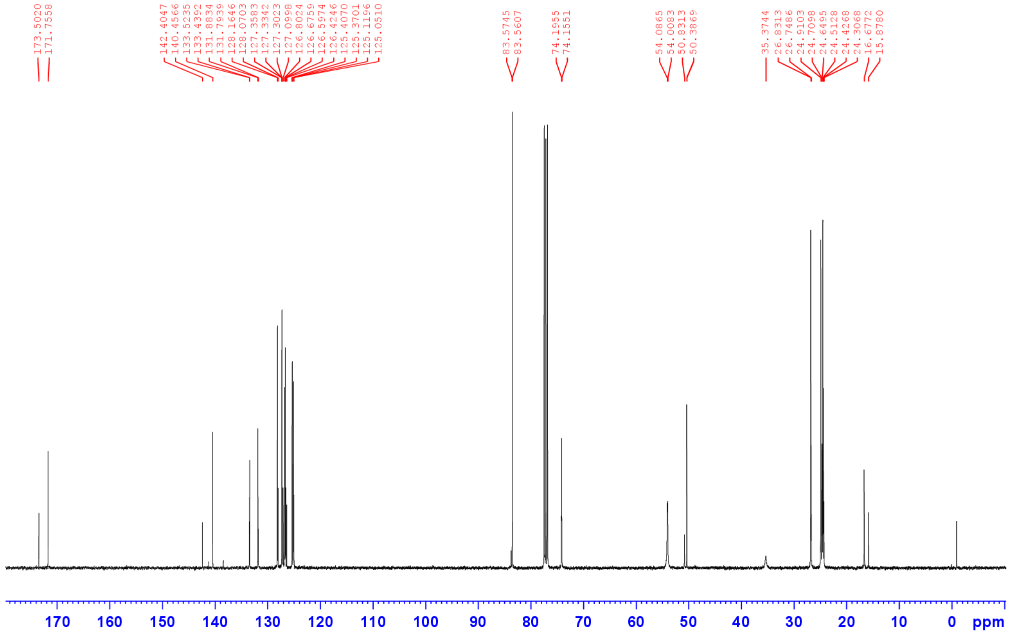

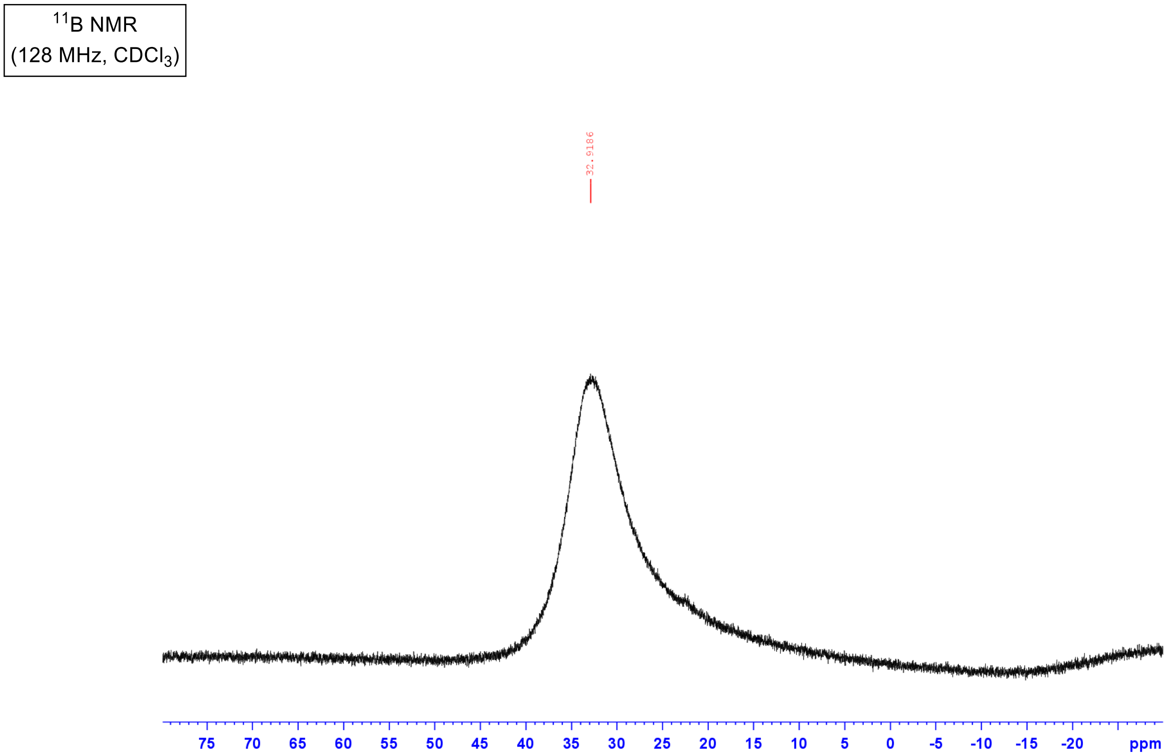

$^{11}$B NMR
(128 MHz, $CDCl_3$)
32.9186
75 70 65 60 55 50 45 40 35 30 25 20 15 10 5 0 -5 -10 -15 -20 ppm

[$^1$H, $^{13}$C{$^1$H}, and $^{11}$B NMR Spectra of **3Ee**]

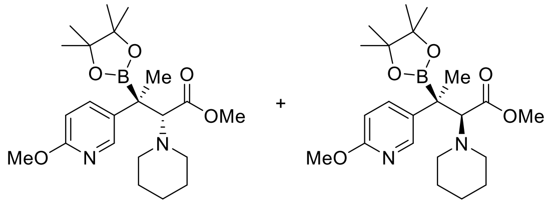


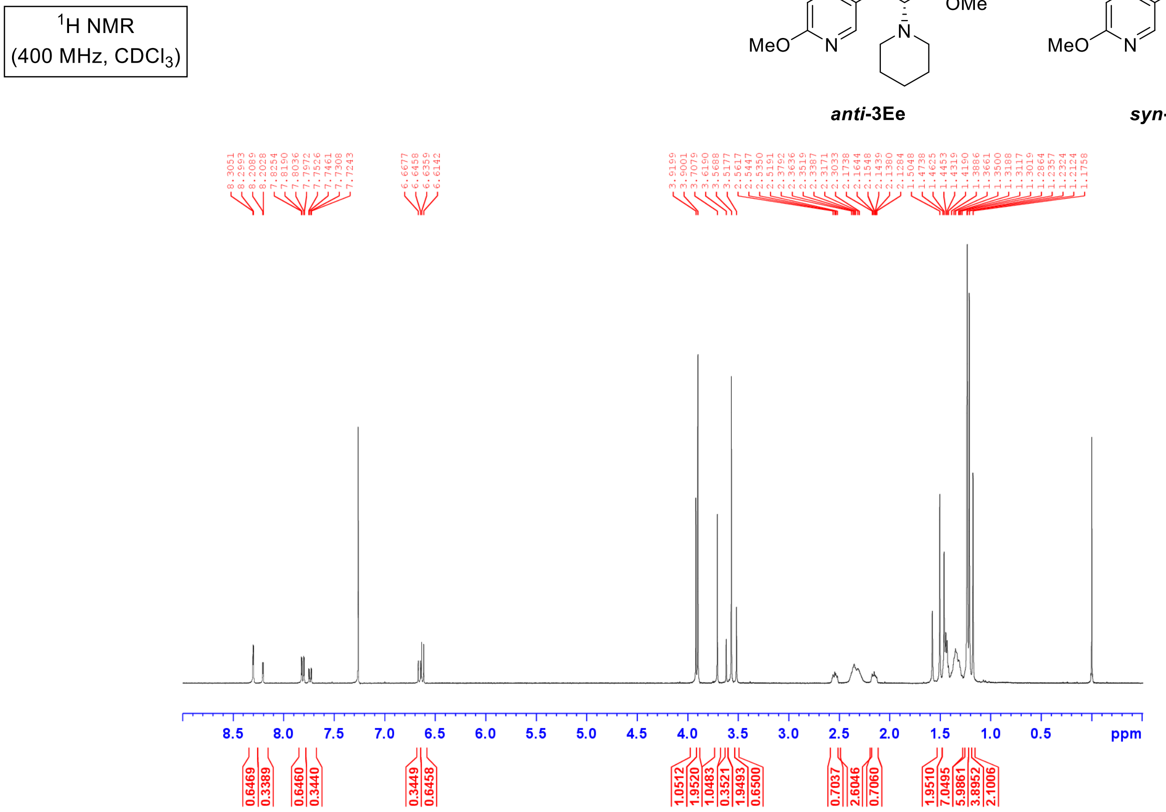


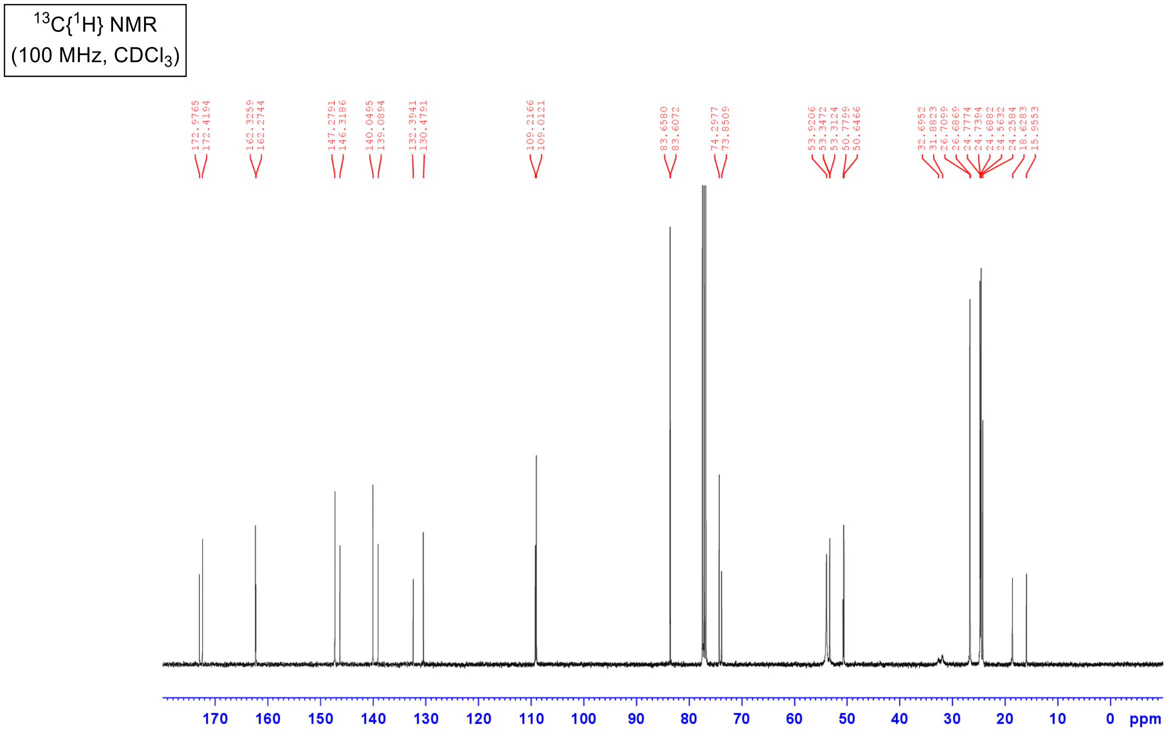

$^{11}B$ NMR
(128 MHz, $CDCl_3$)

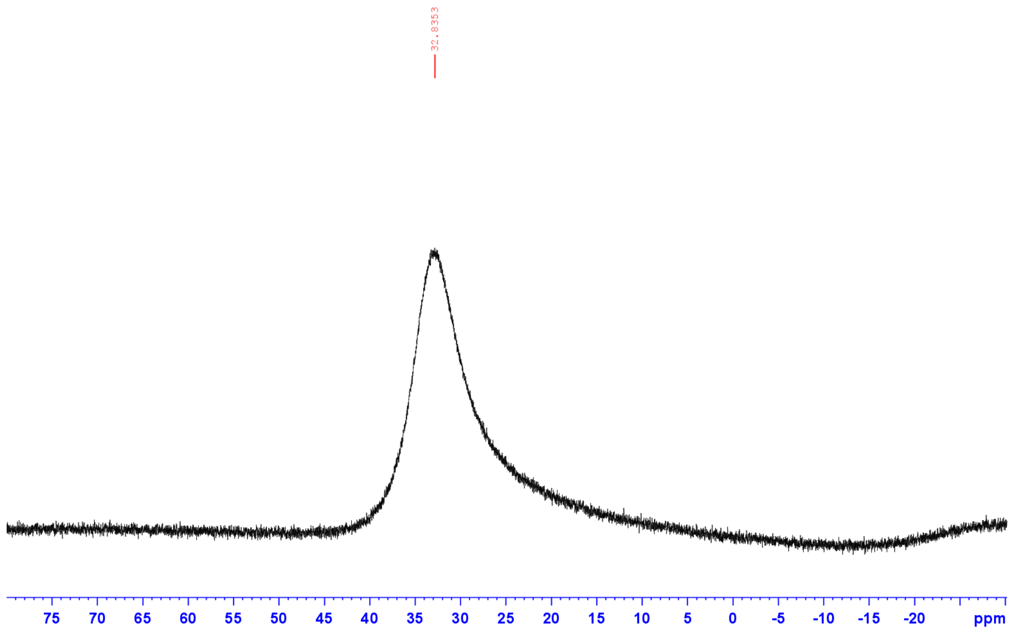

[$^{1}$H, $^{13}$C{$^{1}$H}, and $^{11}$B NMR Spectra of **3Fe**]

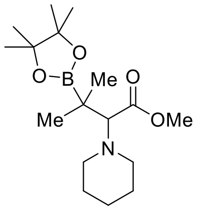


**3Fe**

$^{1}$H NMR
(400 MHz, $CDCl_3$)

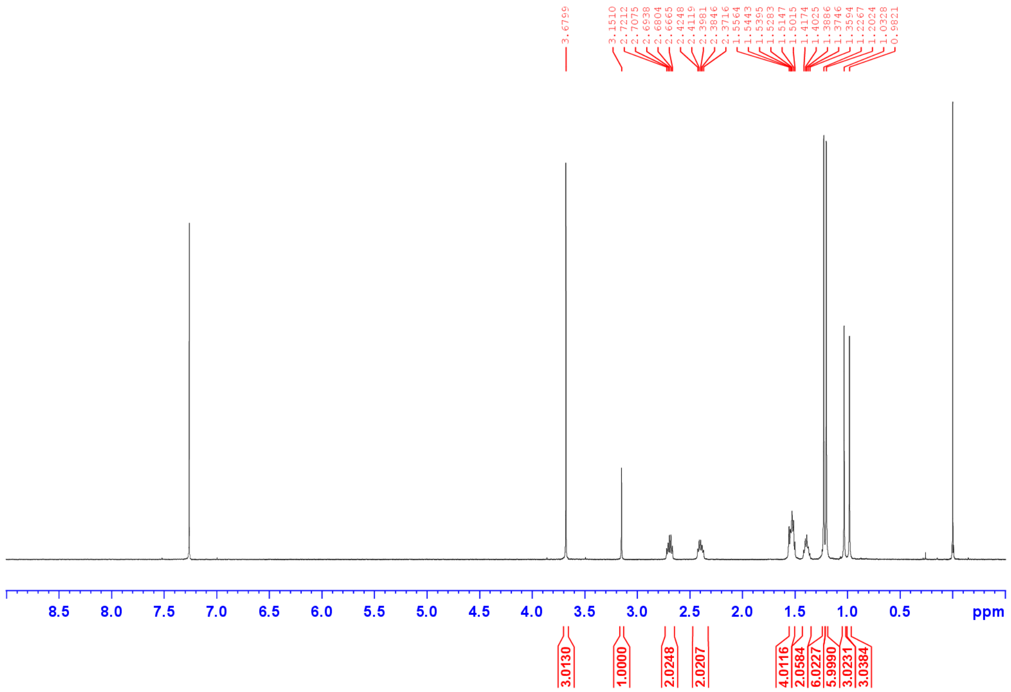

$^{13}$C{$^{1}$H} NMR
(100 MHz, $CDCl_3$)

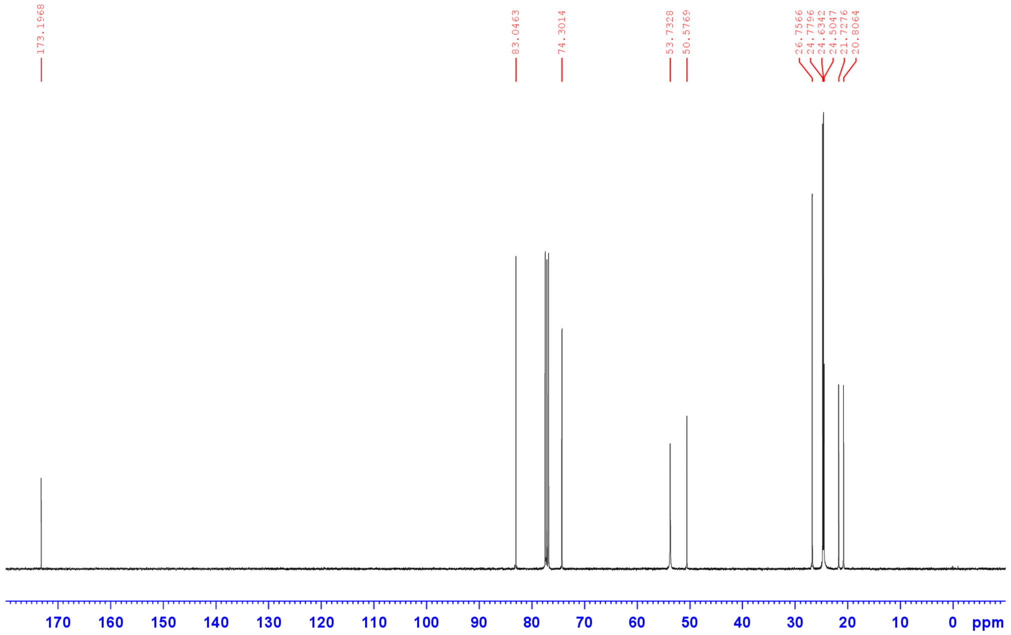

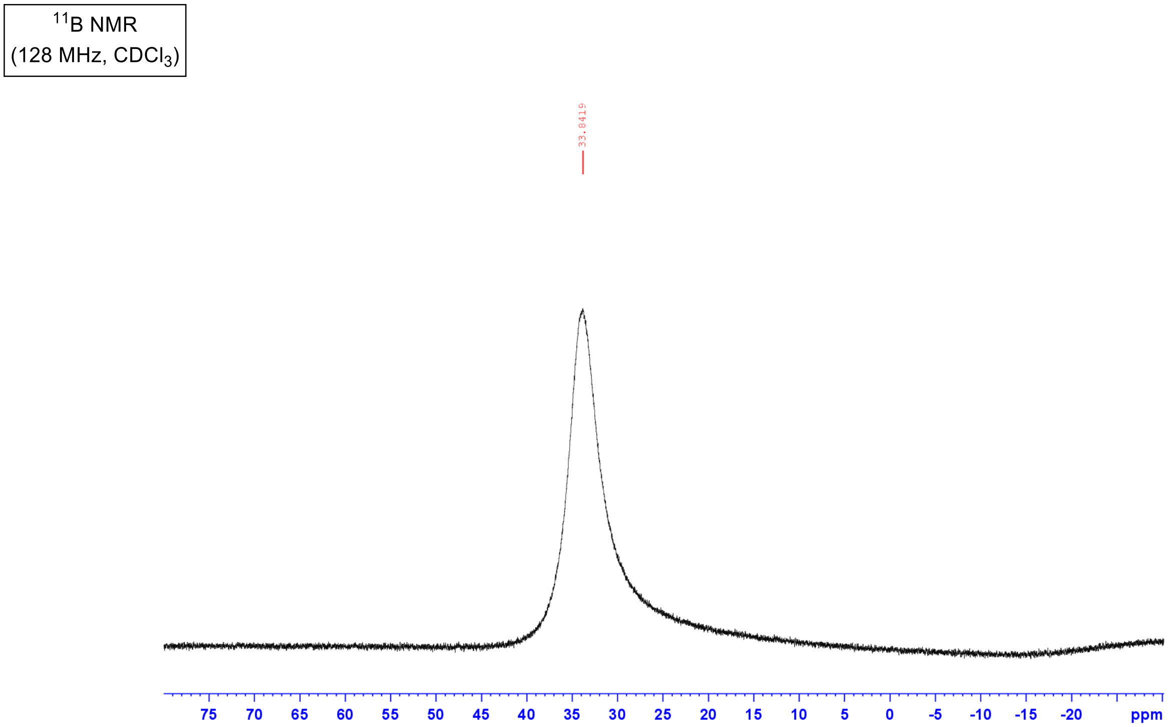
$^{11}$B NMR
(128 MHz, $CDCl_3$)
33.8419
75 70 65 60 55 50 45 40 35 30 25 20 15 10 5 0 -5 -10 -15 -20 ppm

[$^{1}$H, $^{13}$C{$^{1}$H}, and $^{11}$B NMR Spectra of **3Ge**]

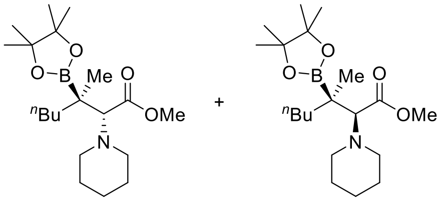


***anti*-3Ge** ***syn*-3Ge**

$^{1}$H NMR
(400 MHz, $CDCl_3$)

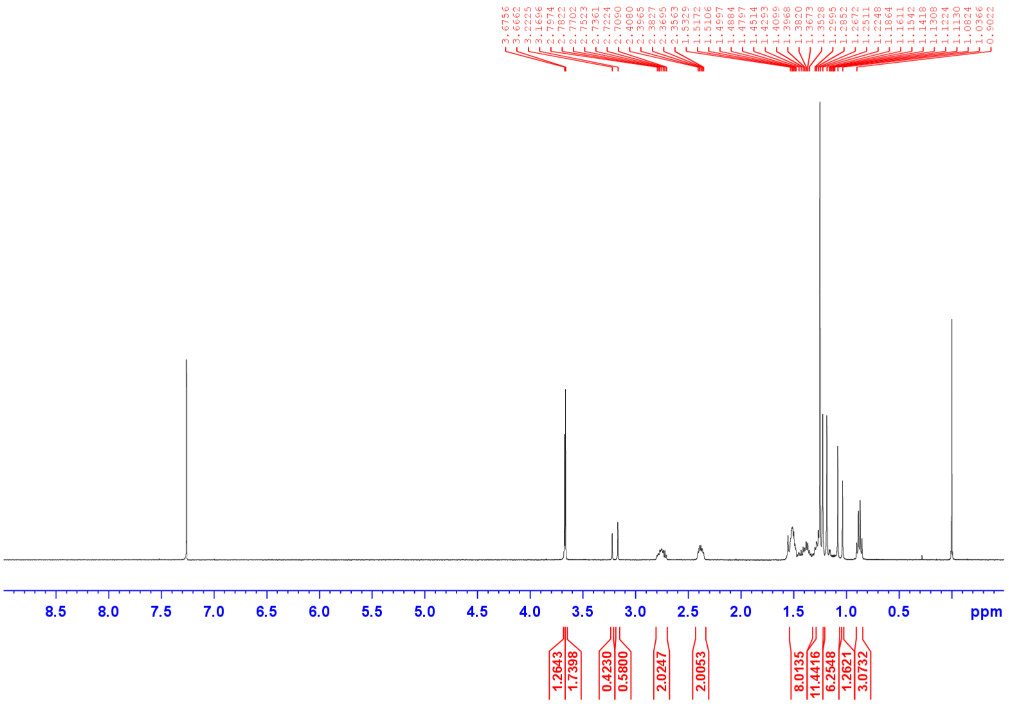


$^{13}$C{$^{1}$H} NMR
(100 MHz, $CDCl_3$)

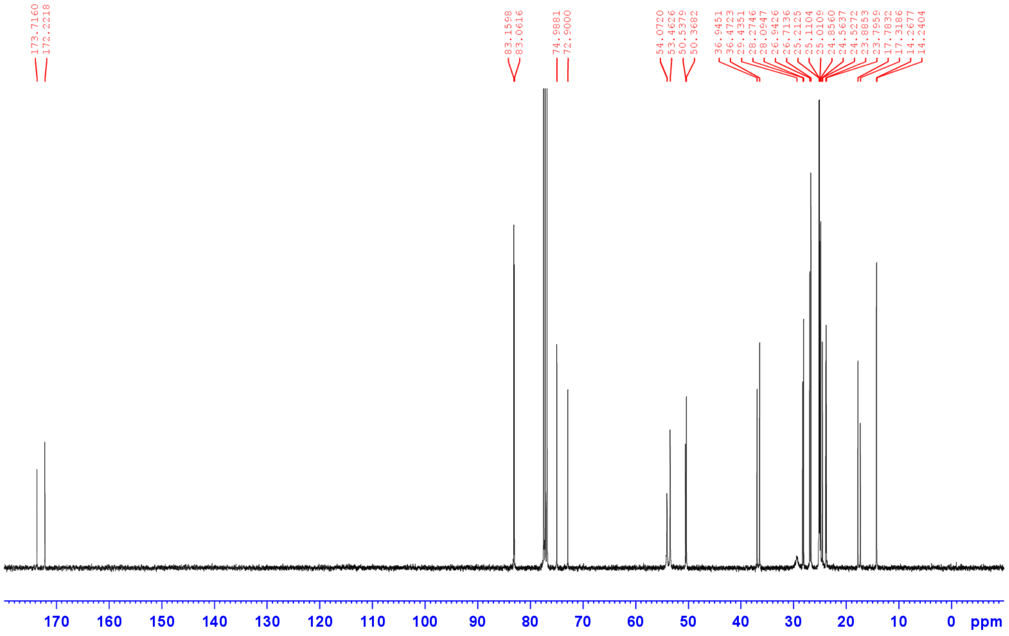

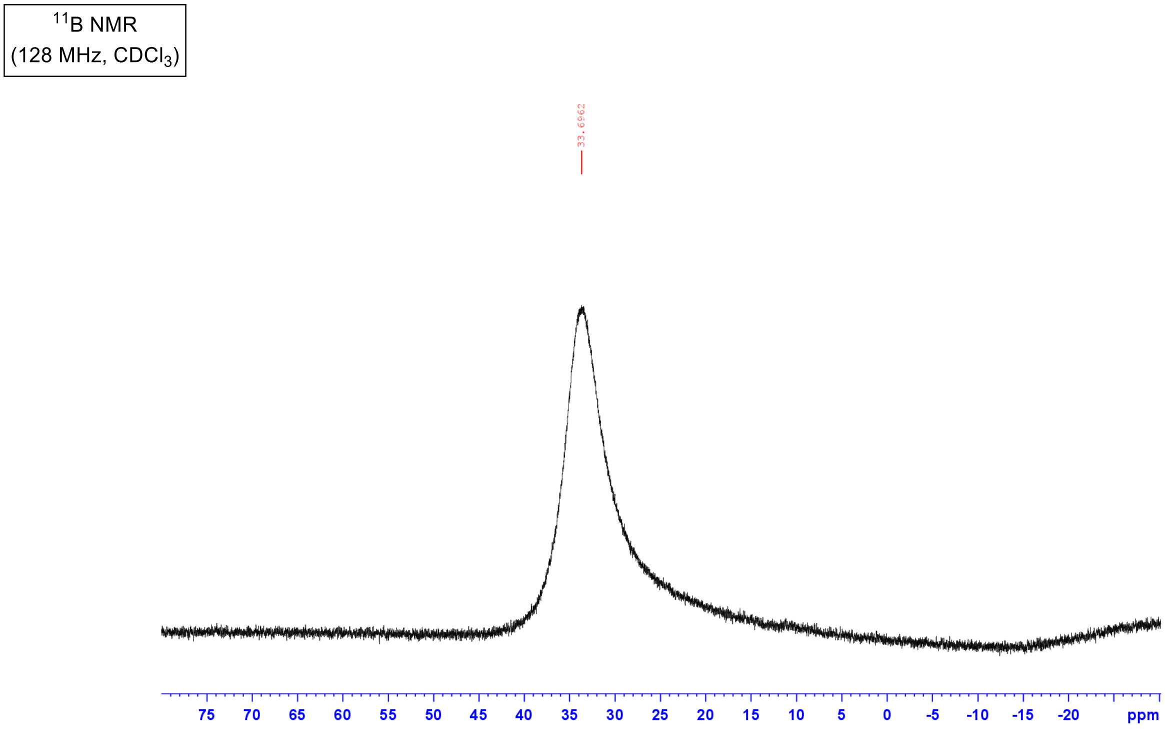

$^{11}$B NMR
(128 MHz, $CDCl_3$)
33.6962
75 70 65 60 55 50 45 40 35 30 25 20 15 10 5 0 -5 -10 -15 -20 ppm

[$^{1}H$, $^{13}C\{^{1}H\}$, and $^{11}B$ NMR Spectra of **3He**]

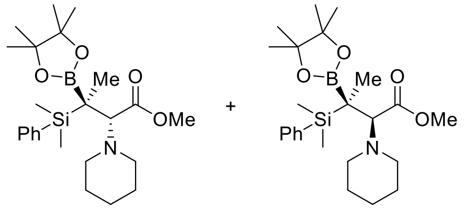


***anti*-3He** **syn-3He**

$^{1}H$ NMR
(400 MHz, $CDCl_3$)

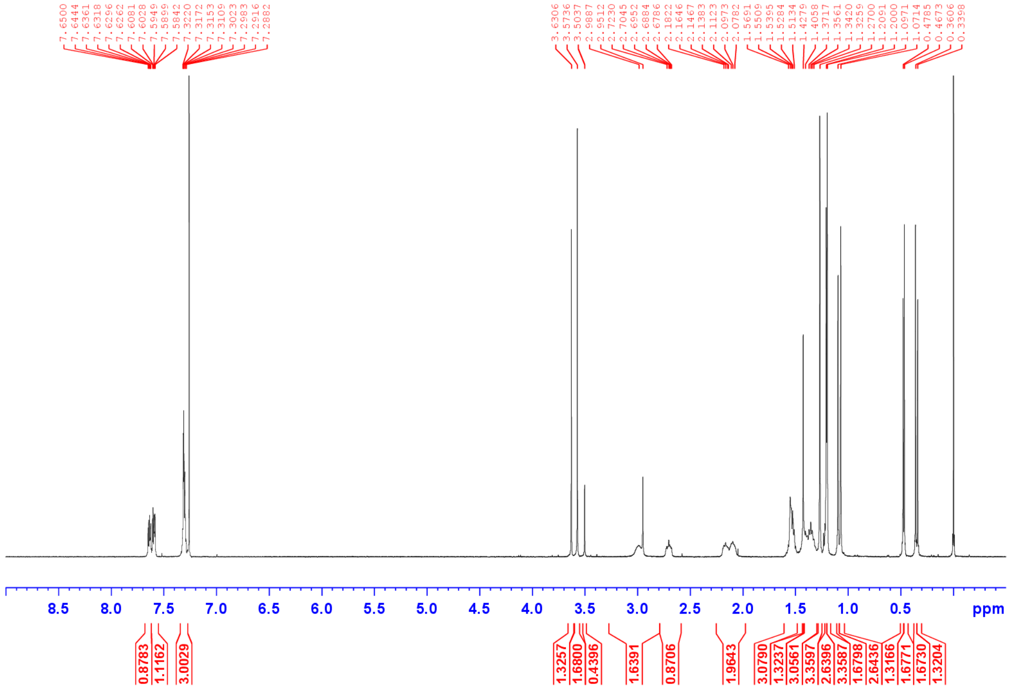


$^{13}C\{^{1}H\}$ NMR
(100 MHz, $CDCl_3$)

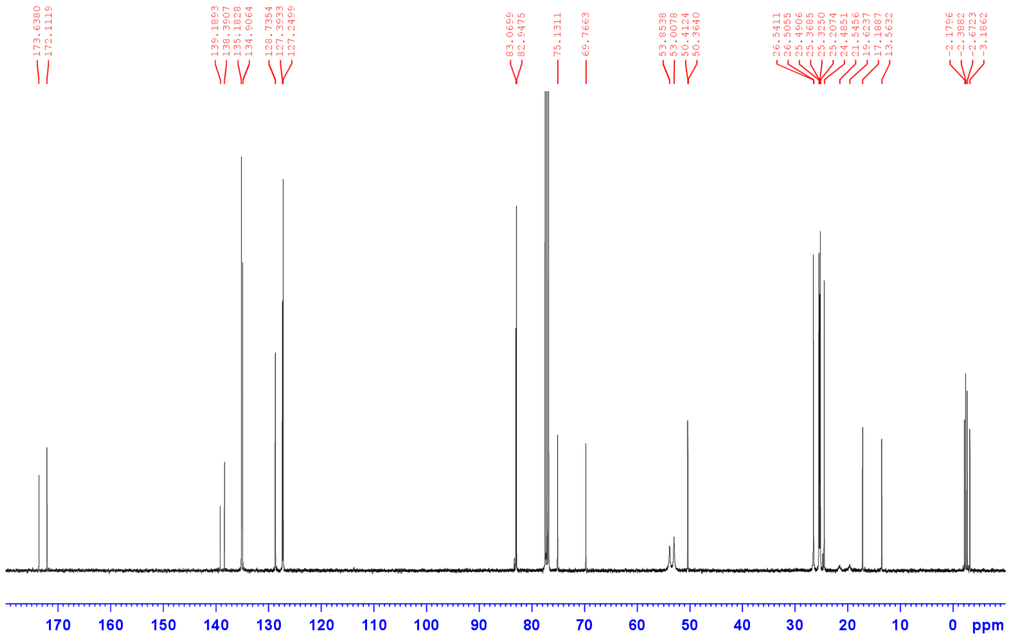

$^{11}B$ NMR
(128 MHz, $CDCl_3$)

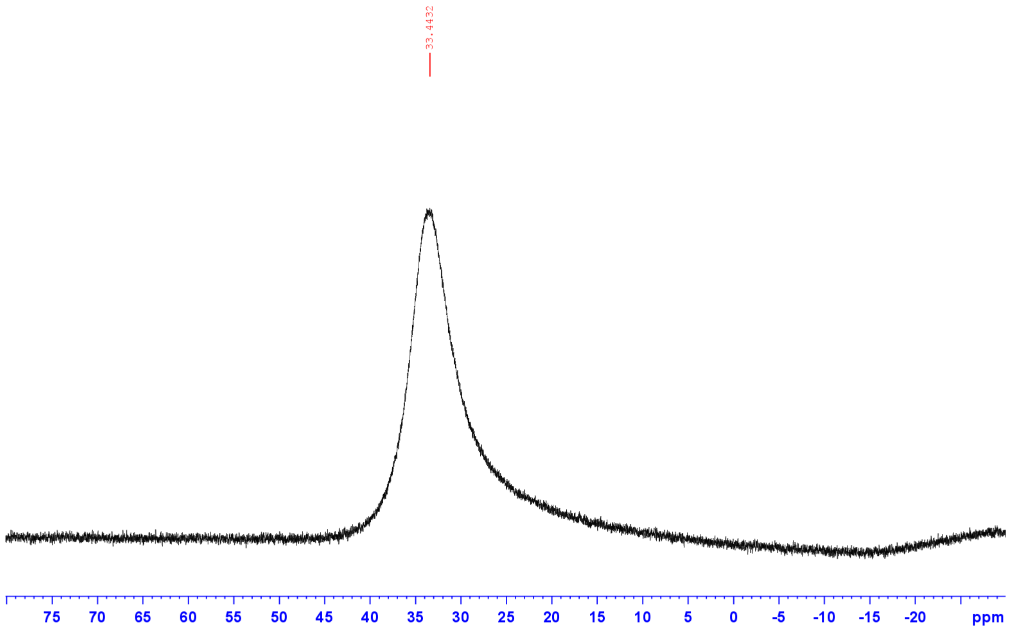

[$^{1}$H, $^{13}$C{$^{1}$H}, and $^{11}$B NMR Spectra of **3Af**]

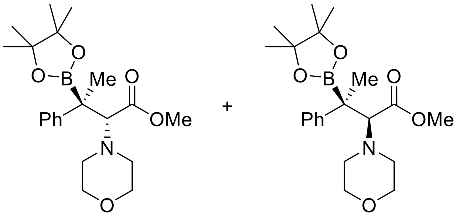


***anti*-3Af** ***syn*-3Af**

$^{1}$H NMR
(400 MHz, $CDCl_3$)

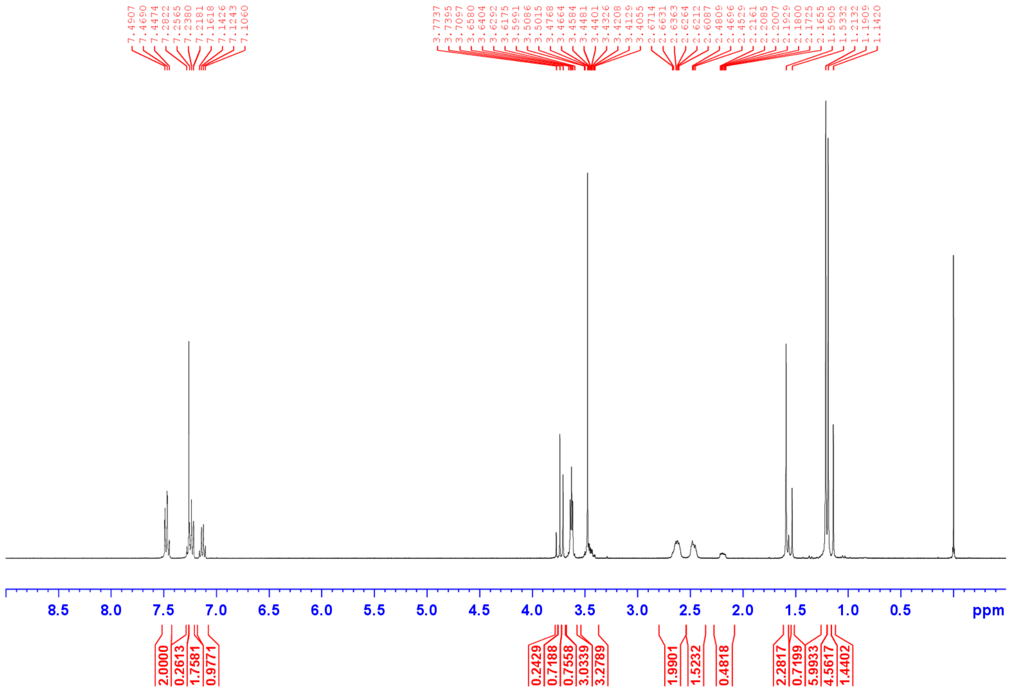


$^{13}$C{$^{1}$H} NMR
(100 MHz, $CDCl_3$)

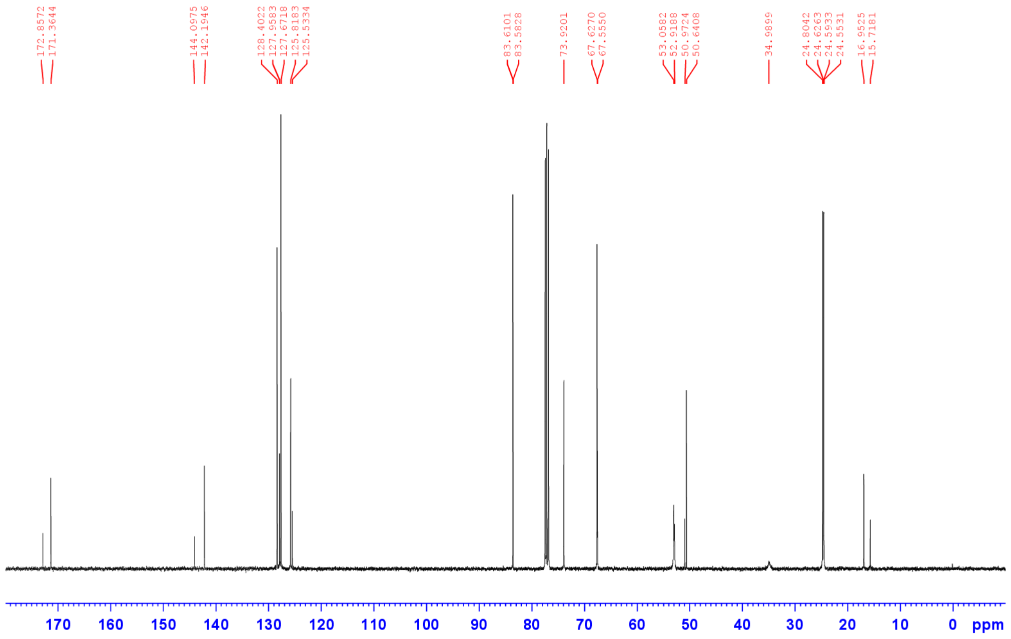

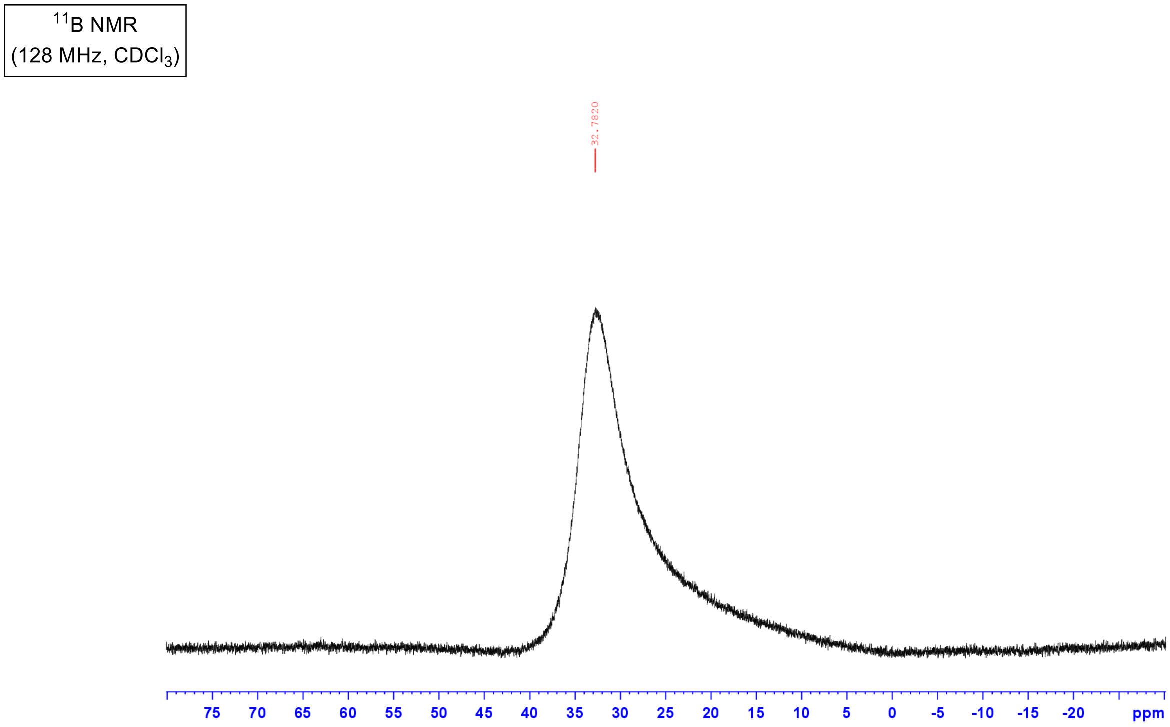

$^{11}$B NMR
(128 MHz, $CDCl_3$)
32.7820
75 70 65 60 55 50 45 40 35 30 25 20 15 10 5 0 -5 -10 -15 -20 ppm

[$^{1}H$, $^{13}C\{^{1}H\}$, and $^{11}B$ NMR Spectra of **3Ac**]

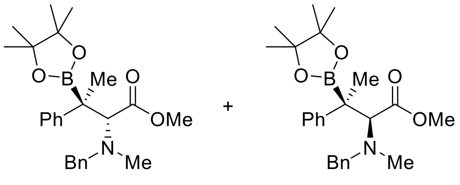


***anti*-3Ac** ***syn*-3Ac**

$^{1}H$ NMR
(400 MHz, $CDCl_3$)

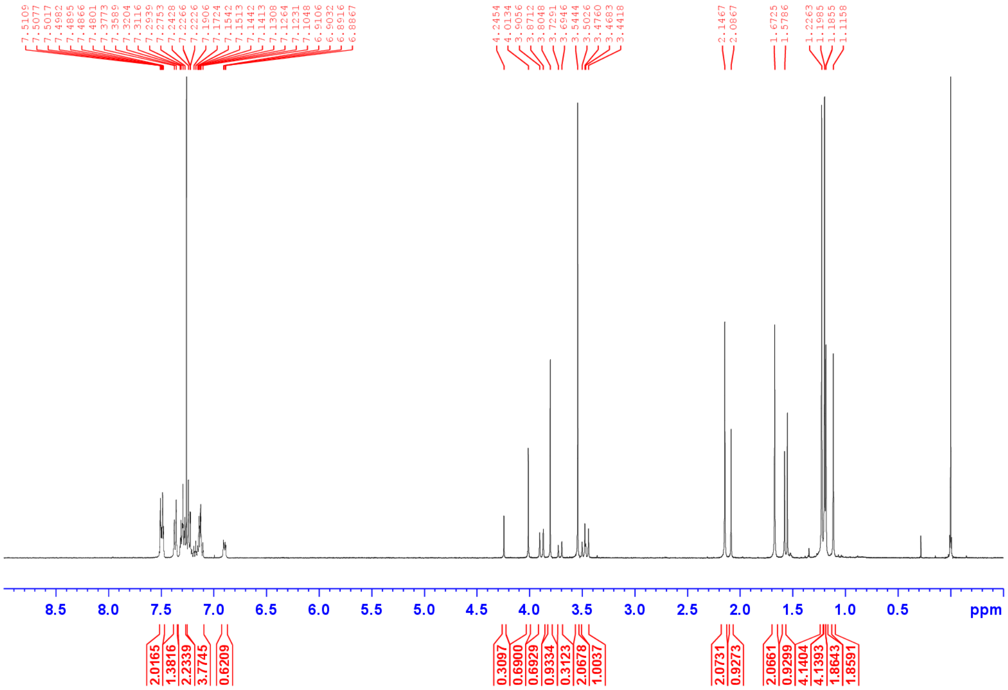


$^{13}C\{^{1}H\}$ NMR
(100 MHz, $CDCl_3$)

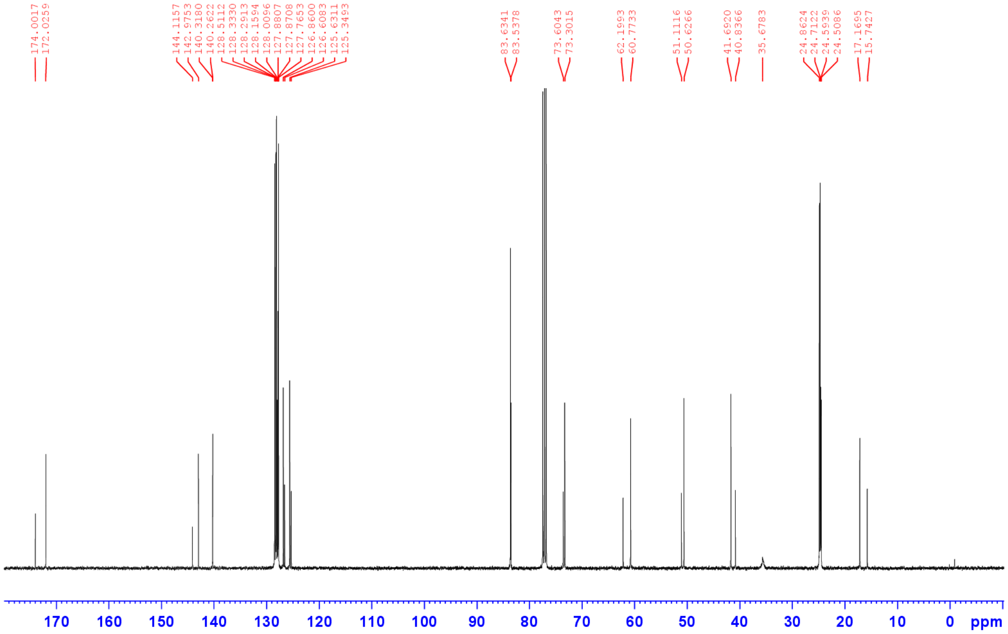

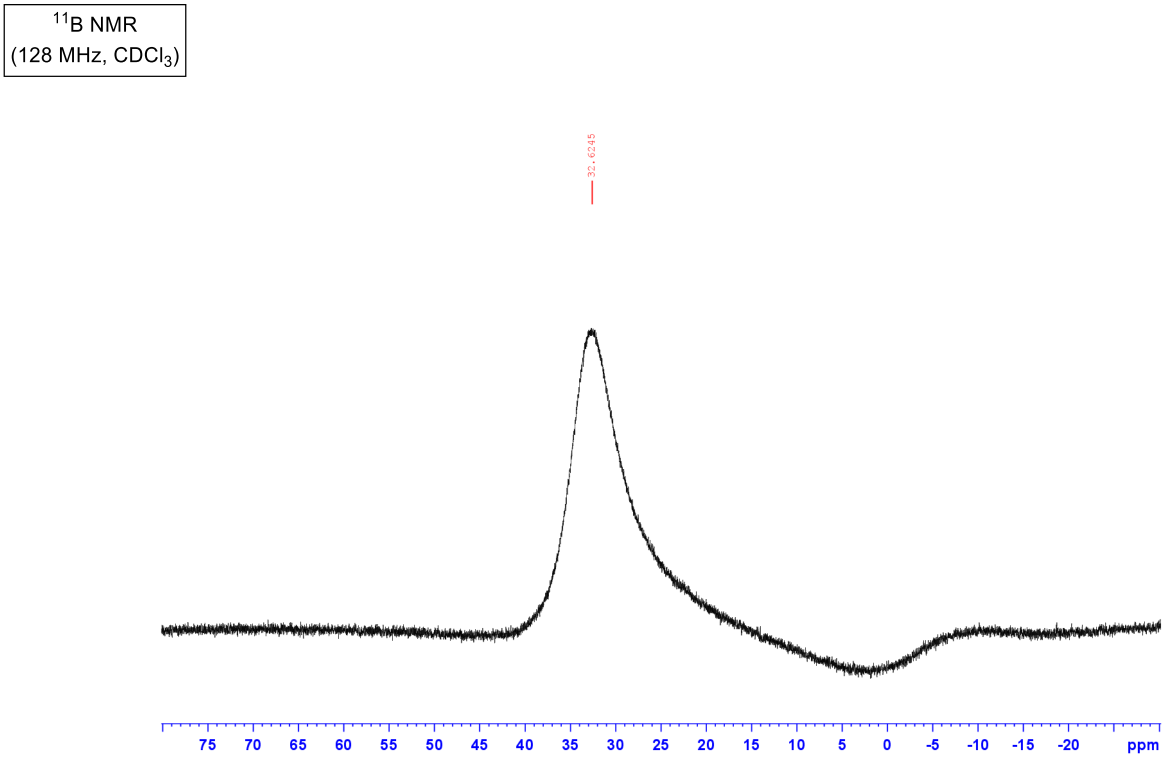

11B NMR
(128 MHz, CDCl3)
32.6245
75 70 65 60 55 50 45 40 35 30 25 20 15 10 5 0 -5 -10 -15 -20 ppm

[$^{1}$H, $^{13}$C{$^{1}$H}, and $^{11}$B NMR Spectra of **3Ab**]

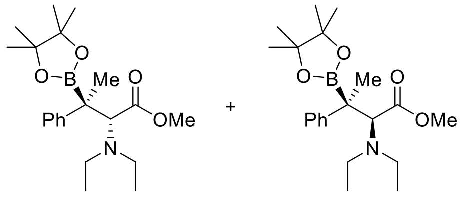


***anti*-3Ab** + ***syn*-3Ab**

$^{1}$H NMR
(400 MHz, $CDCl_3$)

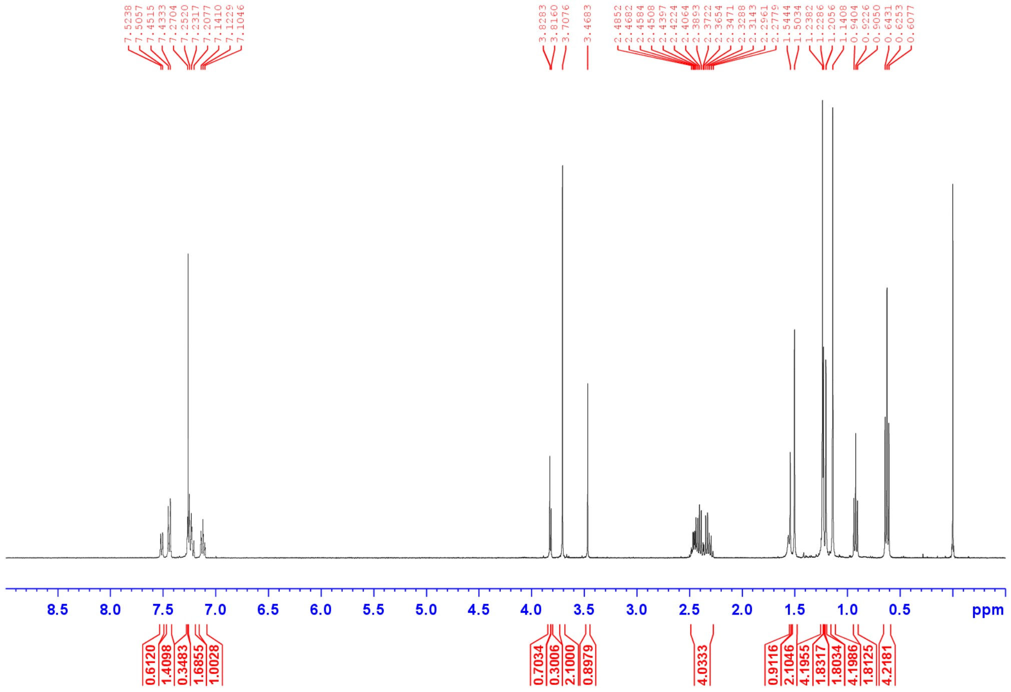


$^{13}$C{$^{1}$H} NMR
(100 MHz, $CDCl_3$)

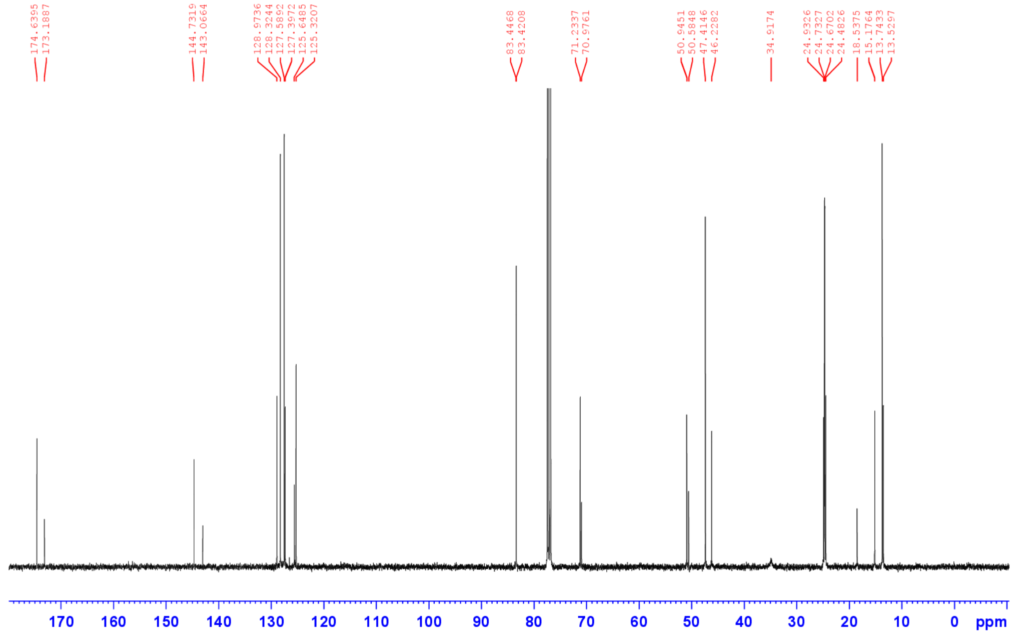

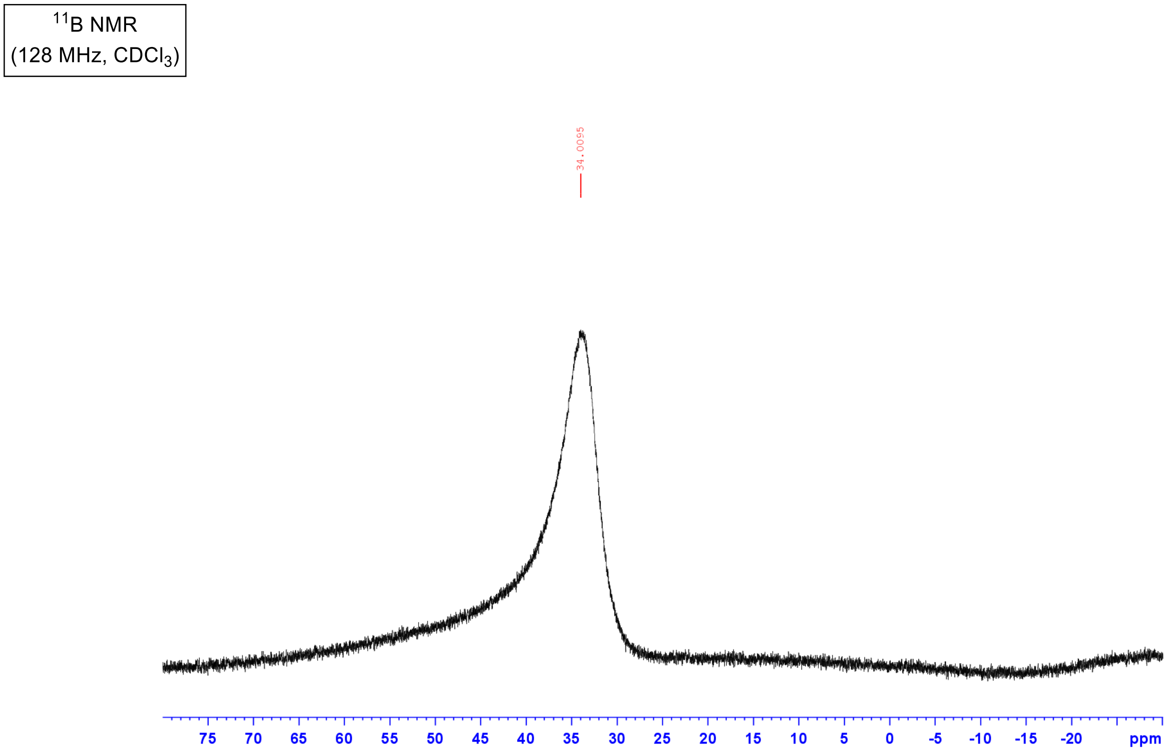

$^{11}$B NMR
(128 MHz, $CDCl_3$)
34.0095
75 70 65 60 55 50 45 40 35 30 25 20 15 10 5 0 -5 -10 -15 -20 ppm

[$^1$H, $^{13}$C{$^1$H}, and $^{11}$B NMR Spectra of ***anti*-3aa-O$^t$Bu**]

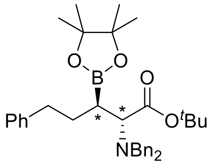


***anti*-3aa-O$^t$Bu**

$^1$H NMR
(400 MHz, $CDCl_3$)

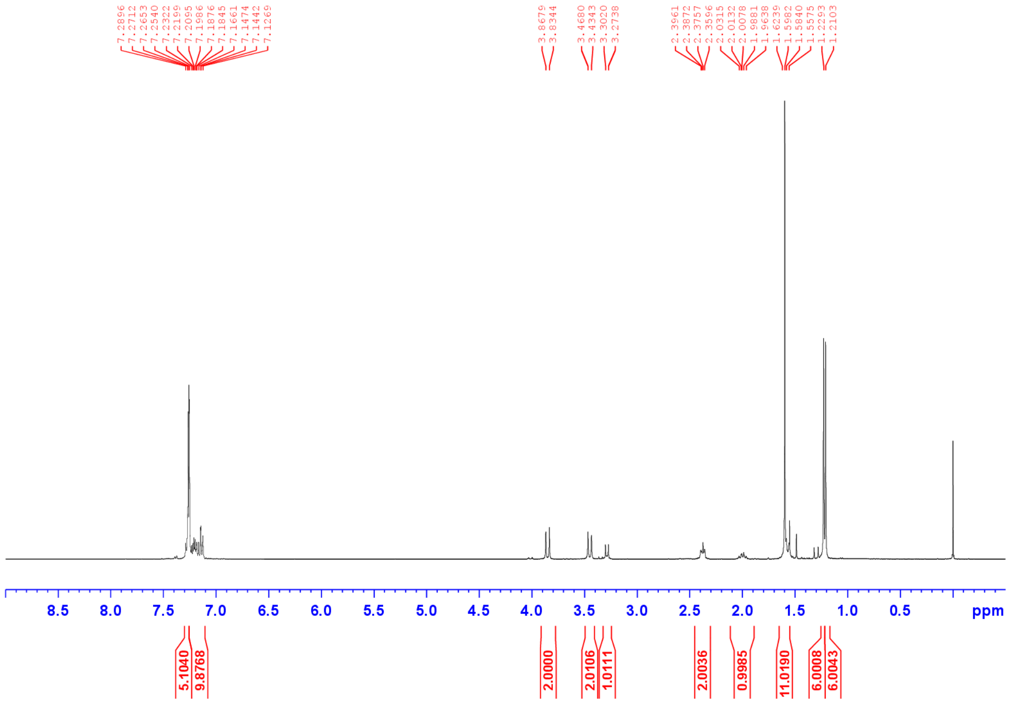


$^{13}$C{$^1$H} NMR
(100 MHz, $CDCl_3$)

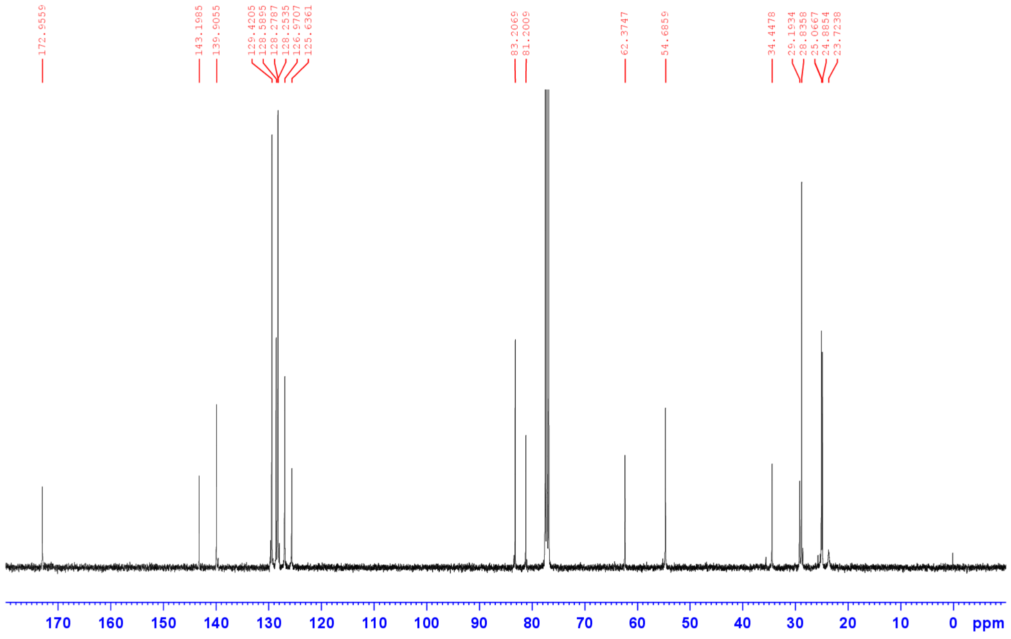

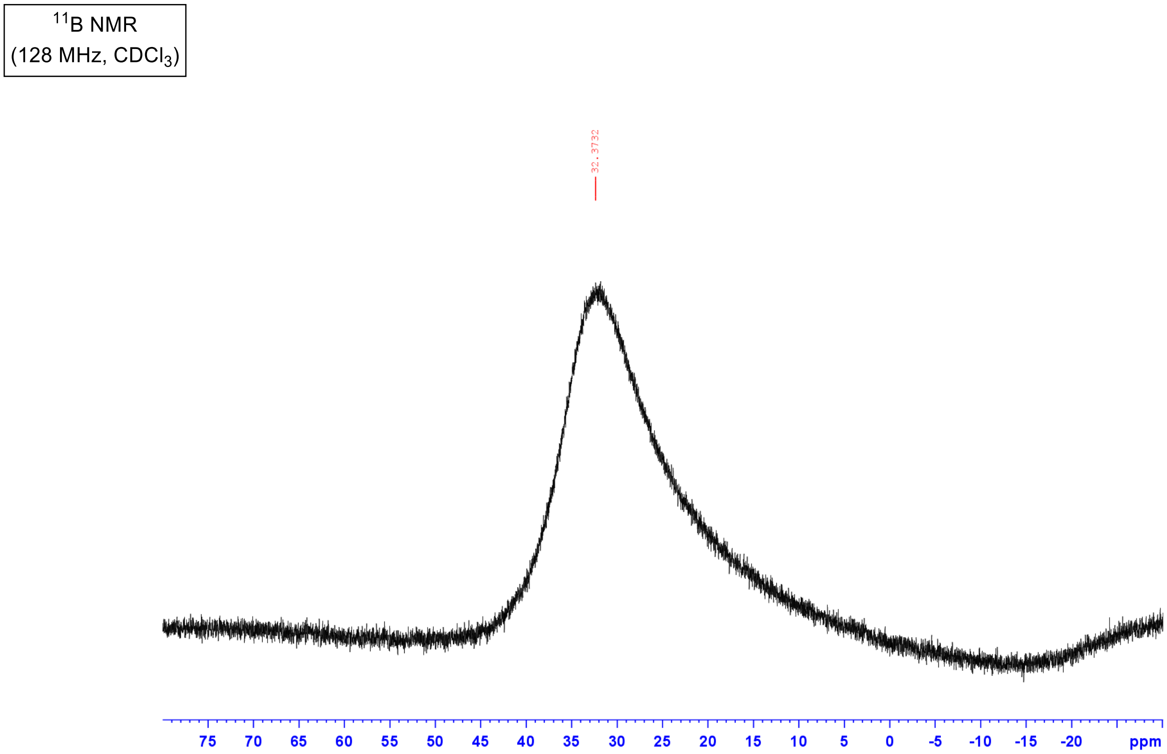

$^{11}$B NMR
(128 MHz, $CDCl_3$)
32.3732
75
70
65
60
55
50
45
40
35
30
25
20
15
10
5
0
-5
-10
-15
-20
ppm

[$^{1}$H, $^{13}$C{$^{1}$H}, and $^{11}$B NMR Spectra of **3ba-O$^{t}$Bu**]

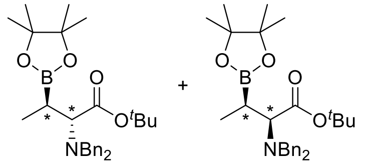

***anti*-3ba-O$^{t}$Bu** + ***syn*-3ba-O$^{t}$Bu**

$^{1}$H NMR
(400 MHz, $CDCl_3$)

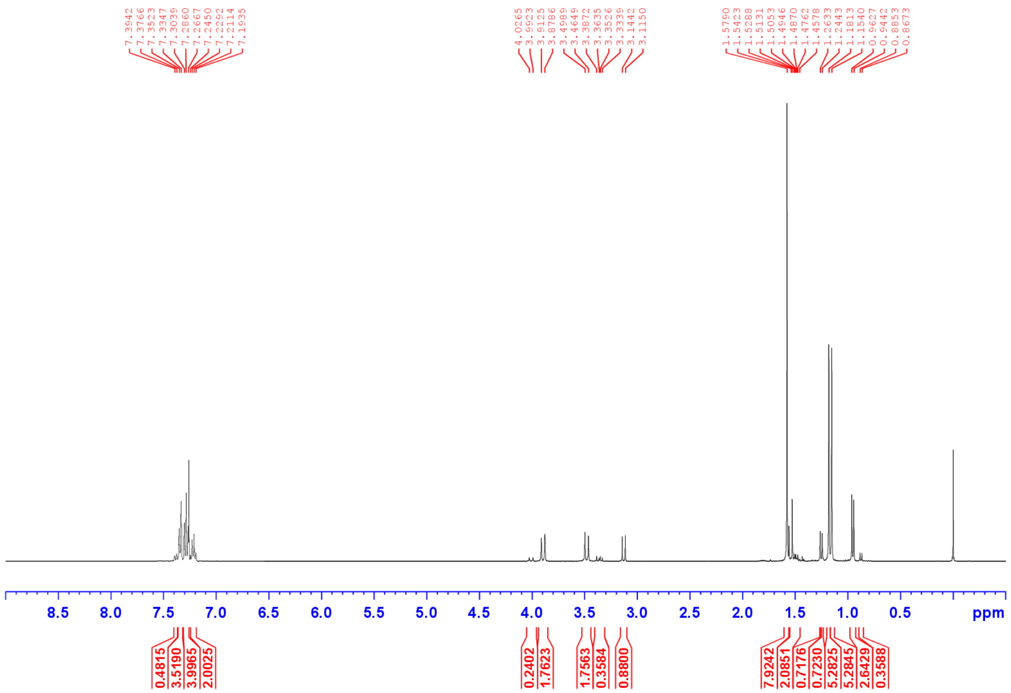

$^{13}$C{$^{1}$H} NMR
(100 MHz, $CDCl_3$)

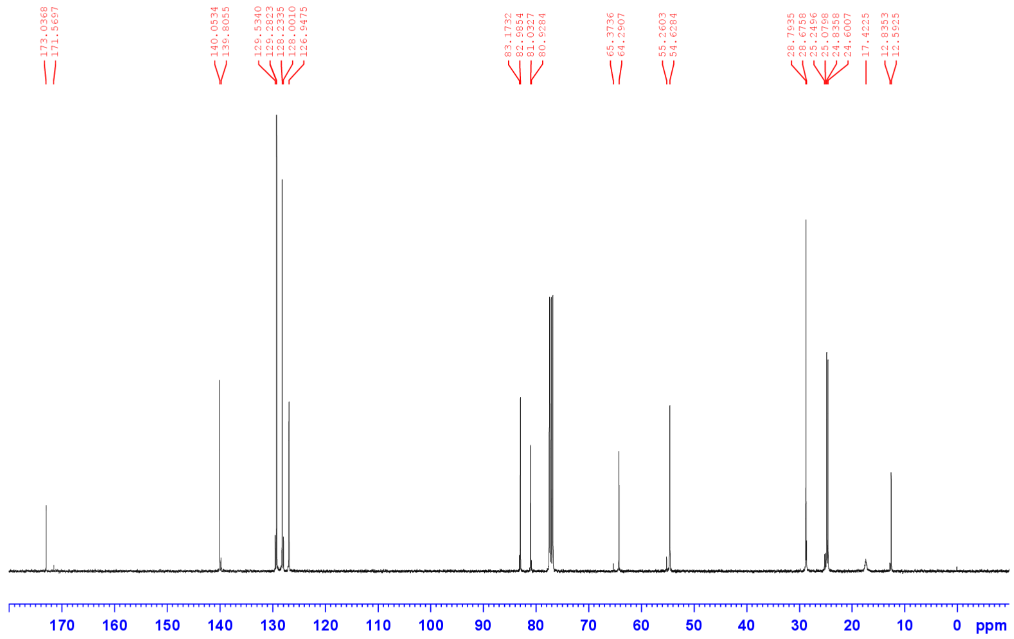

$^{11}$B NMR
(128 MHz, $CDCl_3$)

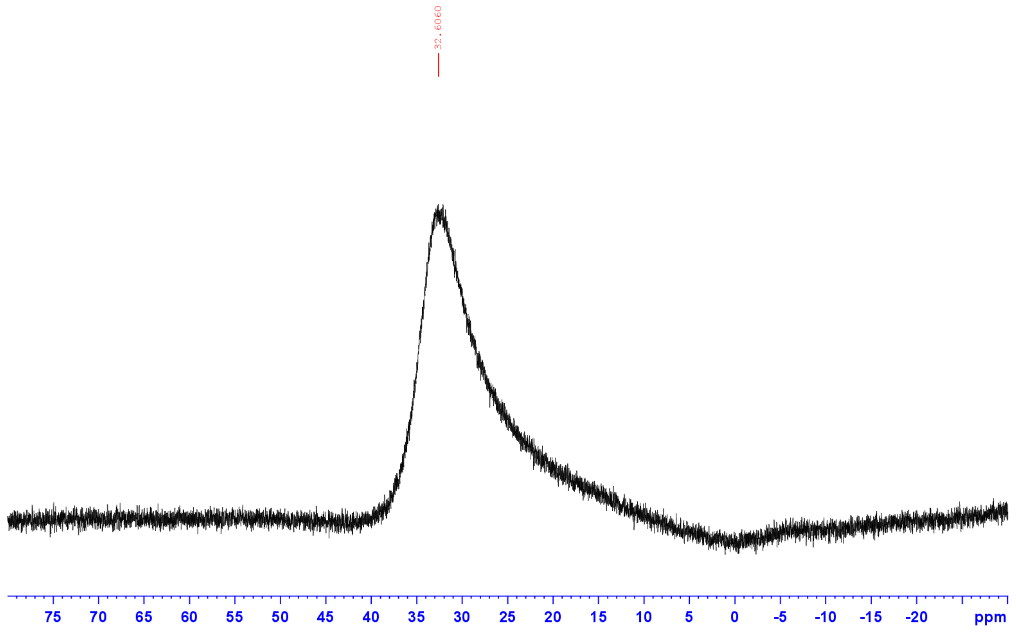

[$^1$H, $^{13}$C{$^1$H}, and $^{11}$B NMR Spectra of ***anti*-3ea-O$^t$Bu**]

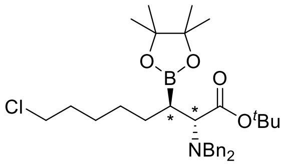


***anti*-3ea-O$^t$Bu**

$^1$H NMR
(400 MHz, $CDCl_3$)

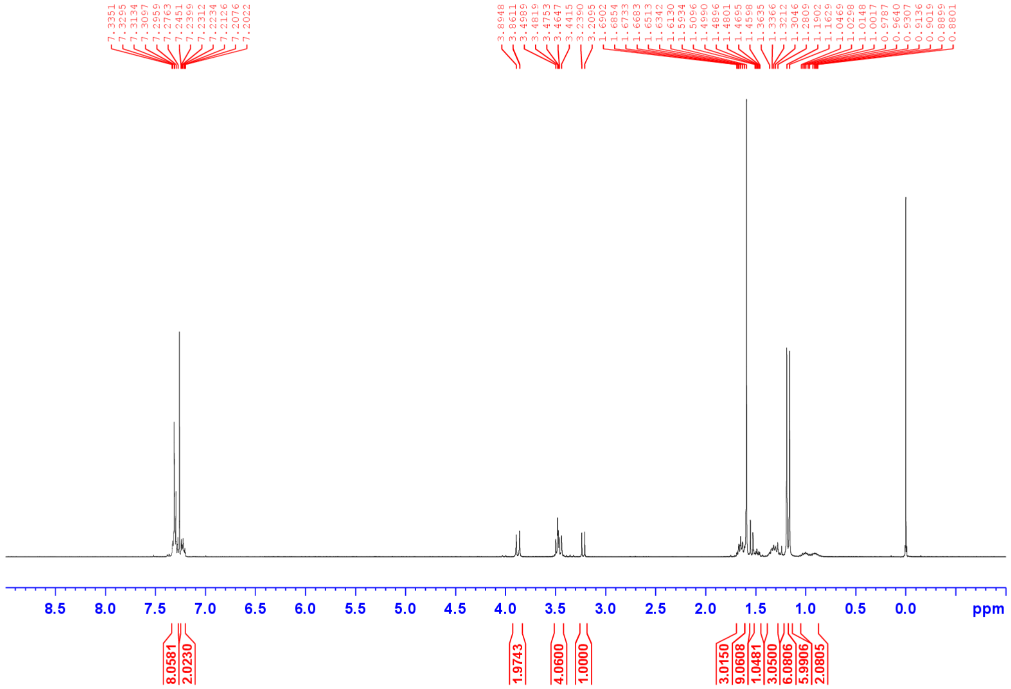

$^{13}$C{$^1$H} NMR
(100 MHz, $CDCl_3$)

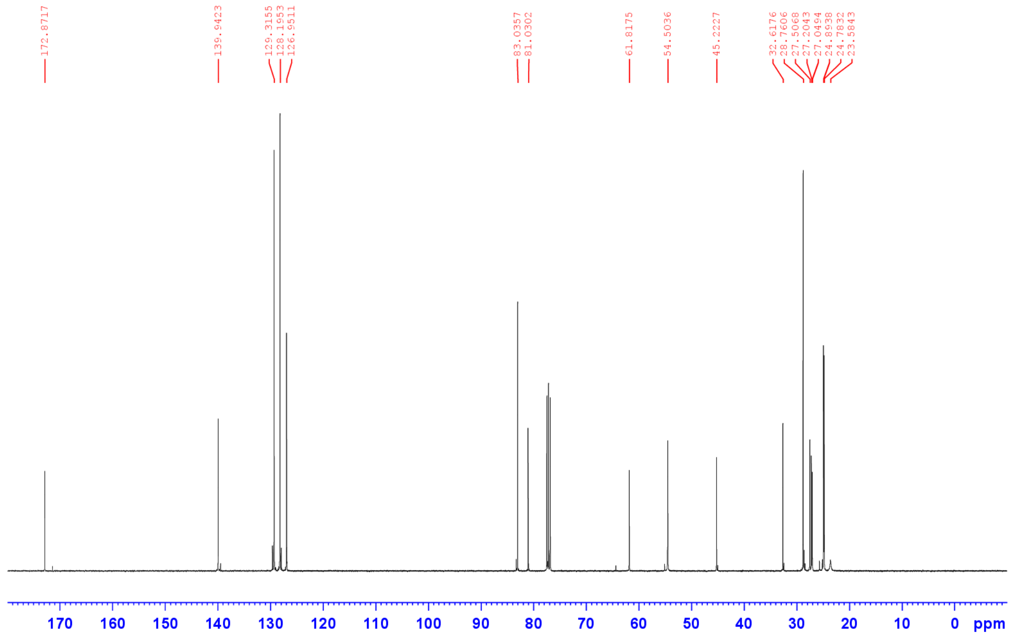

$^{11}$B NMR
(128 MHz, $CDCl_3$)

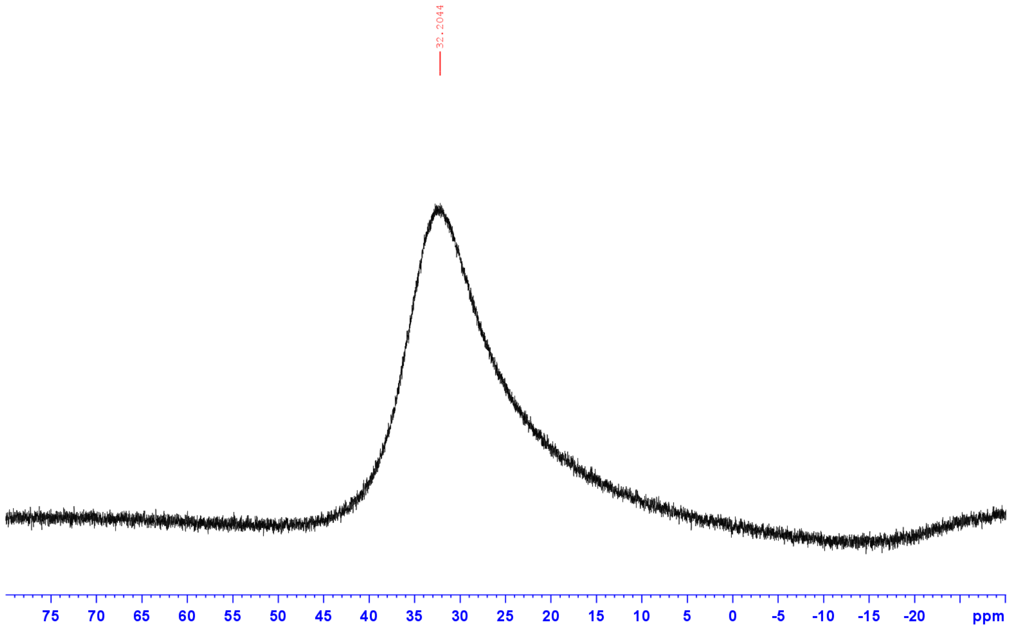

[$^{1}$H, $^{13}$C{$^{1}$H}, and $^{11}$B NMR Spectra of ***anti*-3fa-O*ᵗ*Bu**]

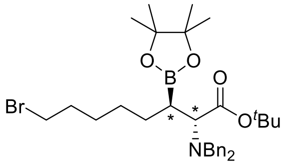


***anti*-3fa-O*ᵗ*Bu**

$^{1}$H NMR
(400 MHz, $CDCl_3$)

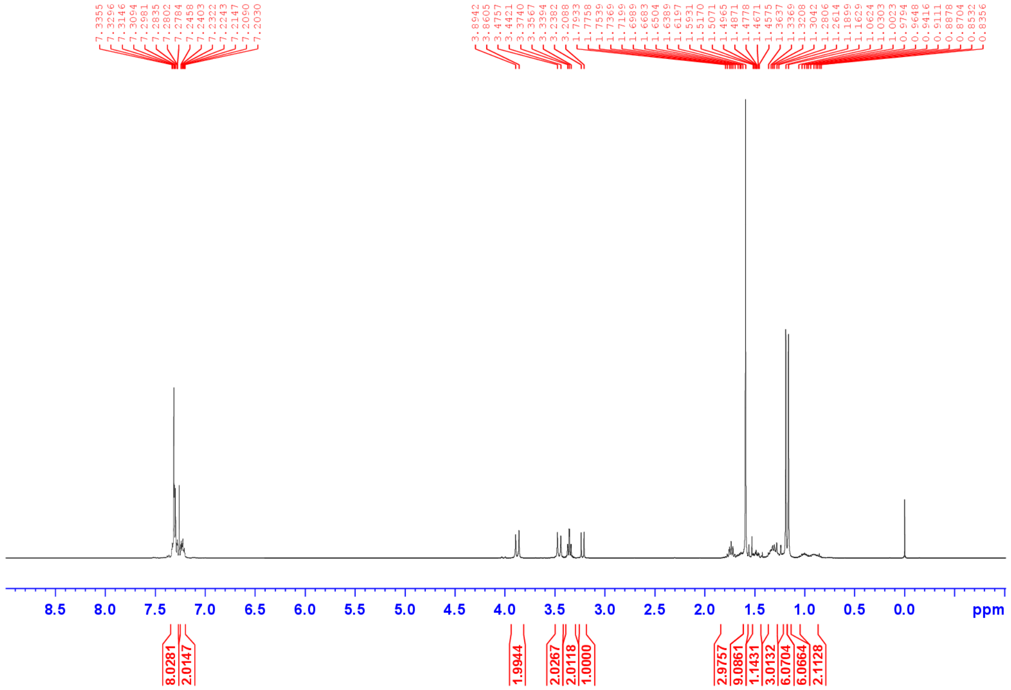


$^{13}$C{$^{1}$H} NMR
(100 MHz, $CDCl_3$)

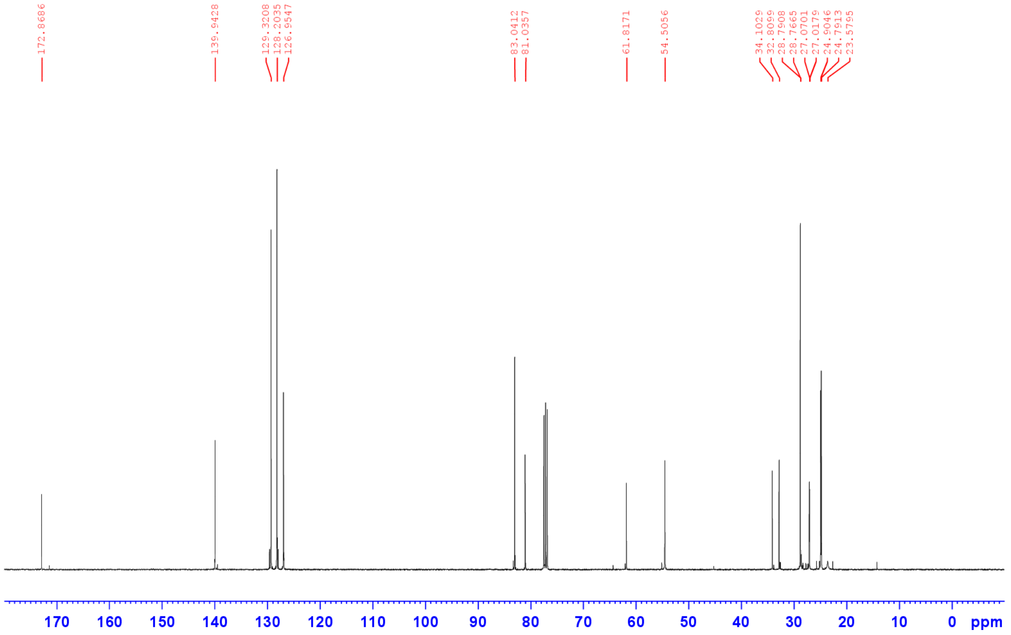

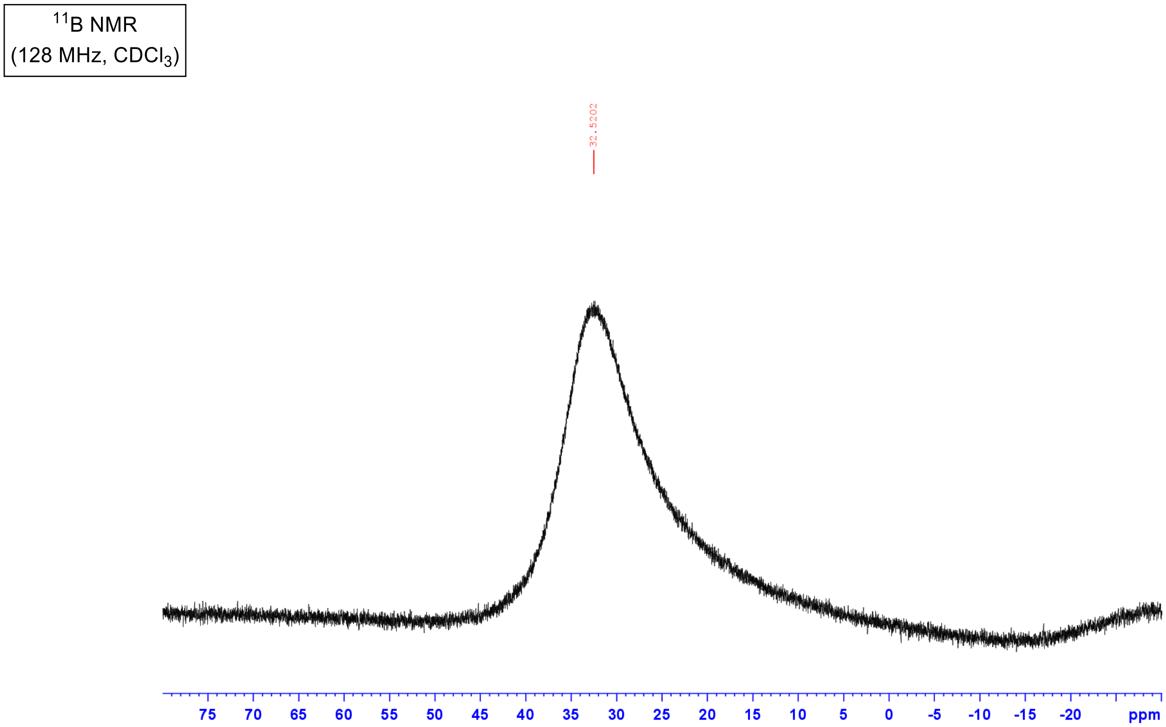

$^{11}$B NMR
(128 MHz, CDCl$_3$)
32.5202
75 70 65 60 55 50 45 40 35 30 25 20 15 10 5 0 -5 -10 -15 -20 ppm

## [$^{1}H$, $^{13}C\{^{1}H\}$, and $^{11}B$ NMR Spectra of ***anti*-3ha-O$^t$Bu**]

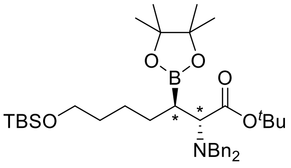


***anti*-3ha-O$^t$Bu**

$^{1}H$ NMR
(400 MHz, $CDCl_3$)

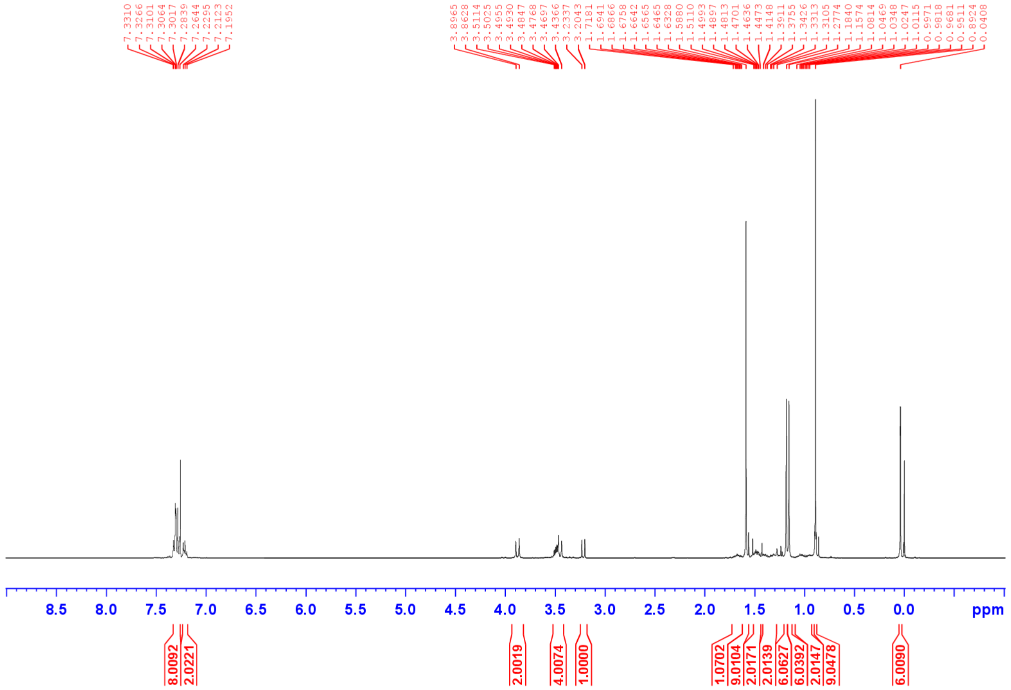


$^{13}C\{^{1}H\}$ NMR
(100 MHz, $CDCl_3$)

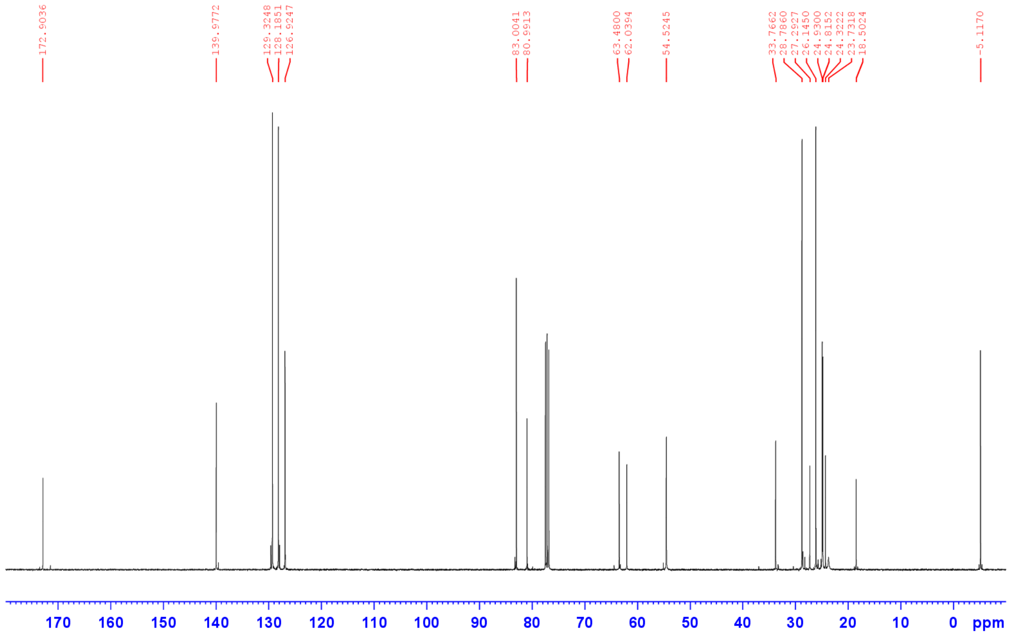

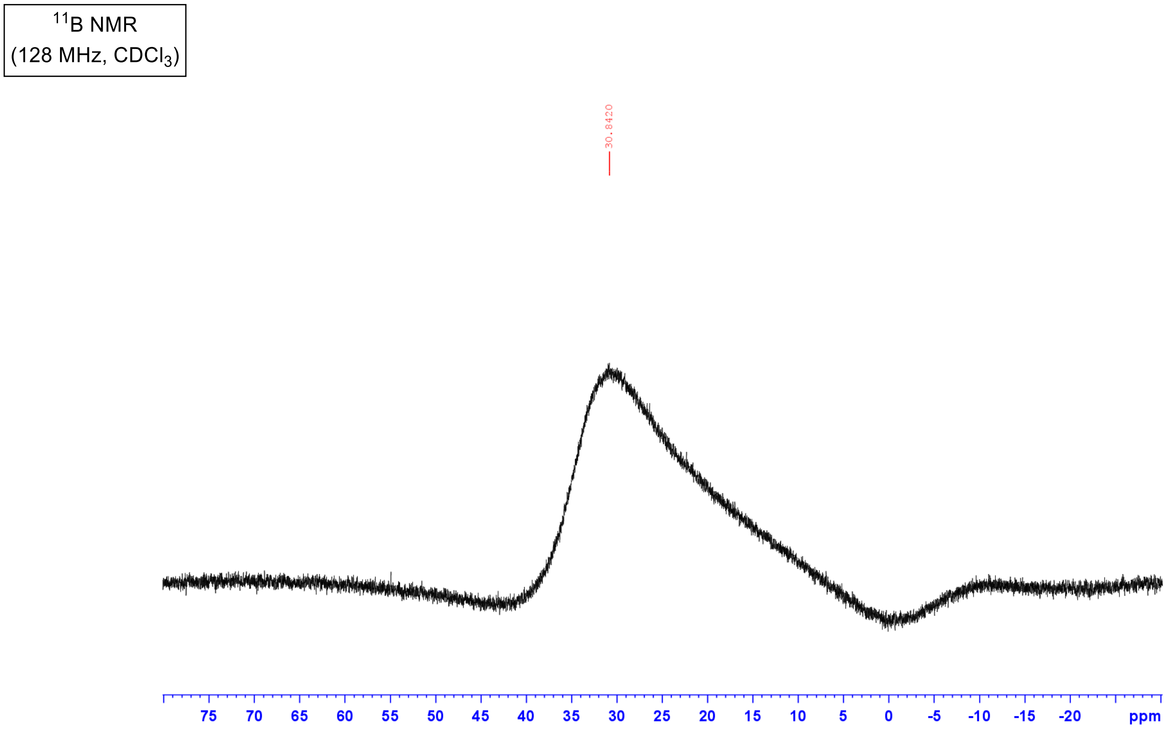

$^{11}$B NMR
(128 MHz, CDCl$_3$)
30.8420
75 70 65 60 55 50 45 40 35 30 25 20 15 10 5 0 -5 -10 -15 -20 ppm

[$^{1}H$, $^{13}C\{^{1}H\}$, and $^{11}B$ NMR Spectra of ***anti*-3ia-O$^t$Bu**]

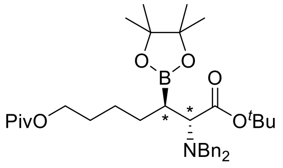


***anti*-3ia-O$^t$Bu**

$^{1}H$ NMR
(400 MHz, $CDCl_3$)

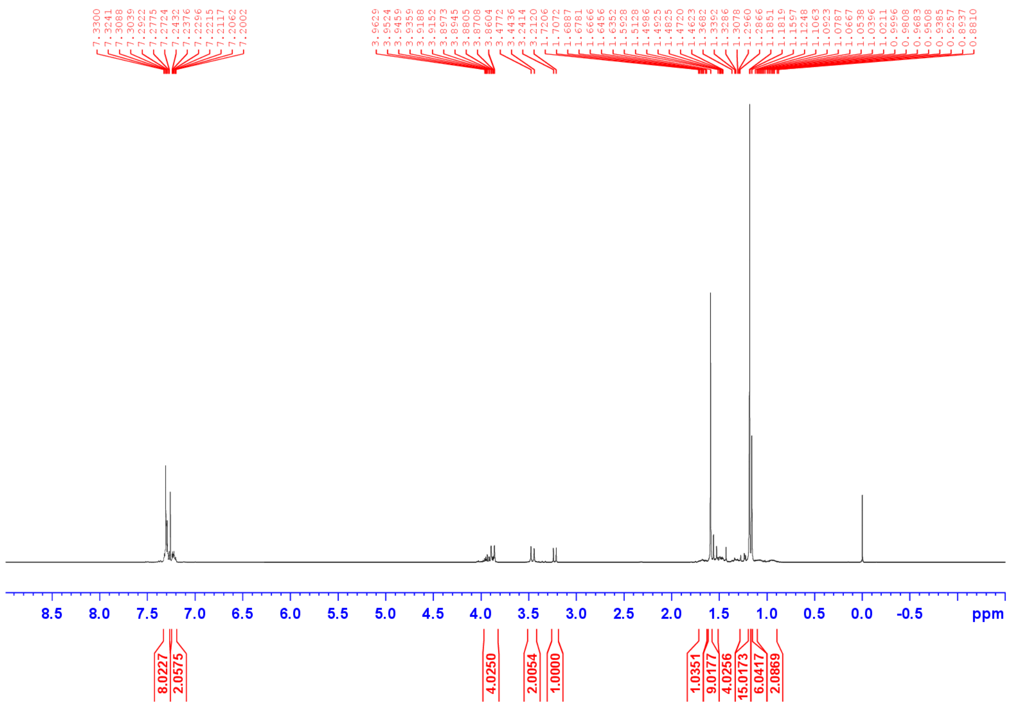

$^{13}C\{^{1}H\}$ NMR
(100 MHz, $CDCl_3$)

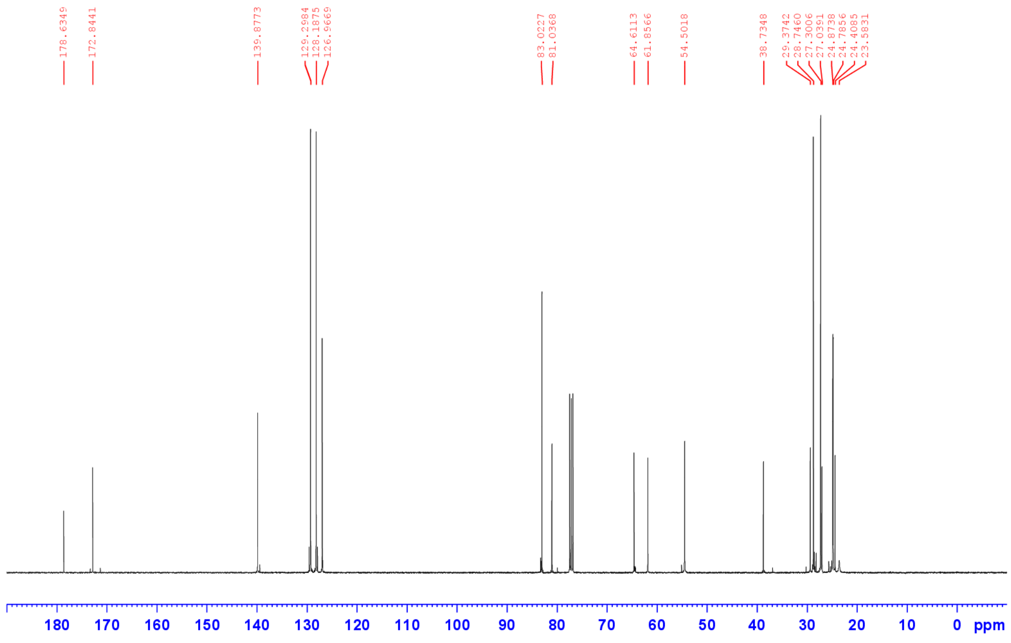

$^{11}B$ NMR
(128 MHz, $CDCl_3$)

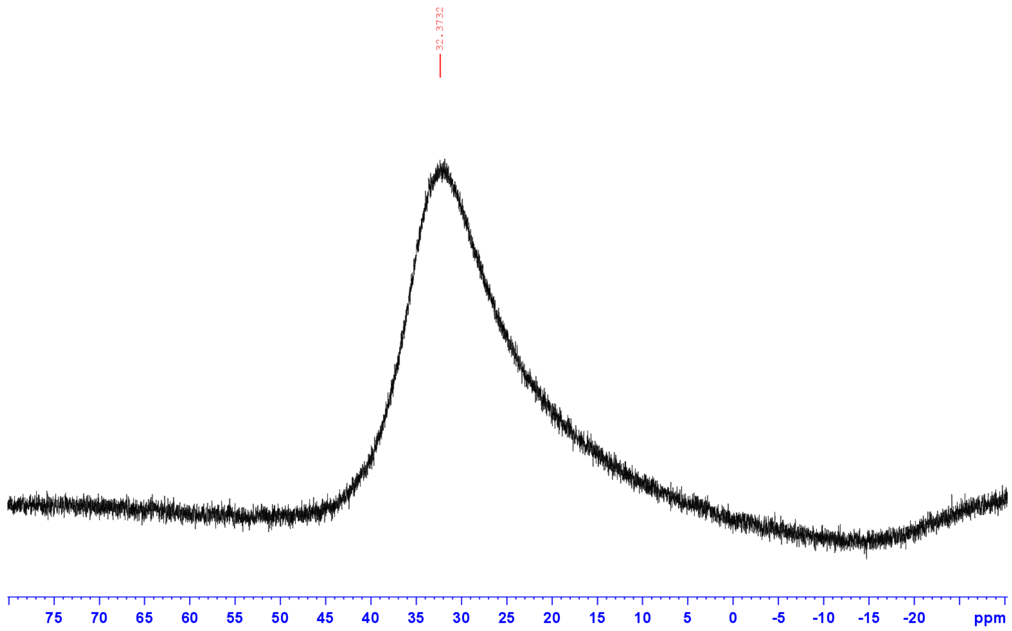

[$^{1}$H, $^{13}$C{$^{1}$H}, and $^{11}$B NMR Spectra of ***anti*-3ja-O$^{t}$Bu**]

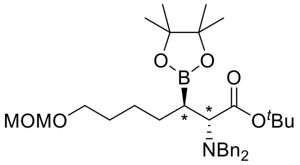


***anti*-3ja-O$^{t}$Bu**

$^{1}$H NMR
(400 MHz, $CDCl_3$)

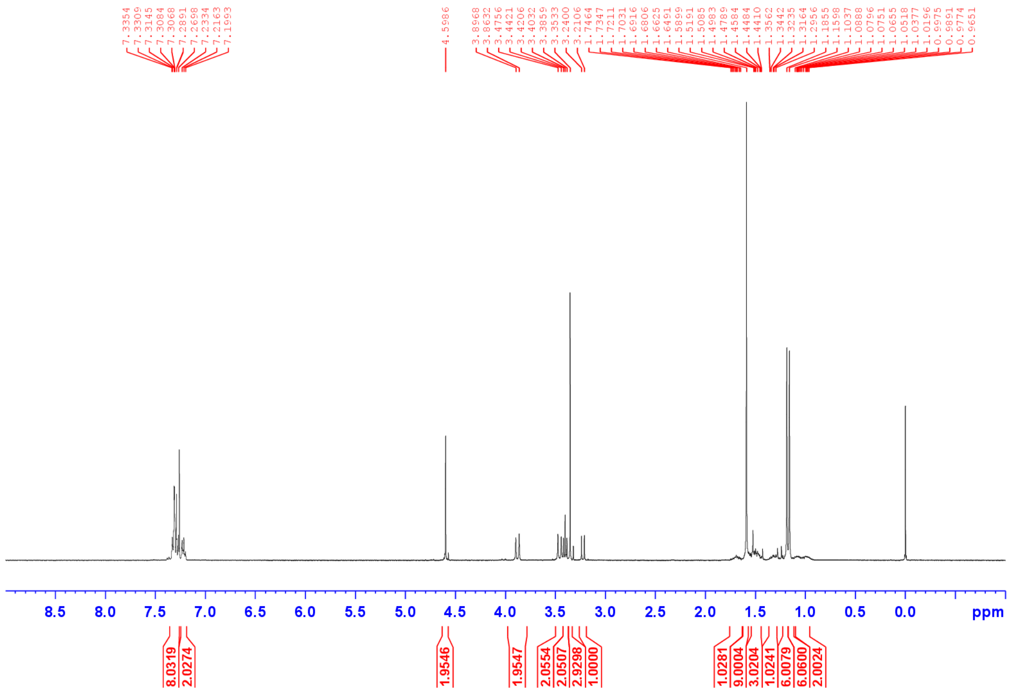

$^{13}$C{$^{1}$H} NMR
(100 MHz, $CDCl_3$)

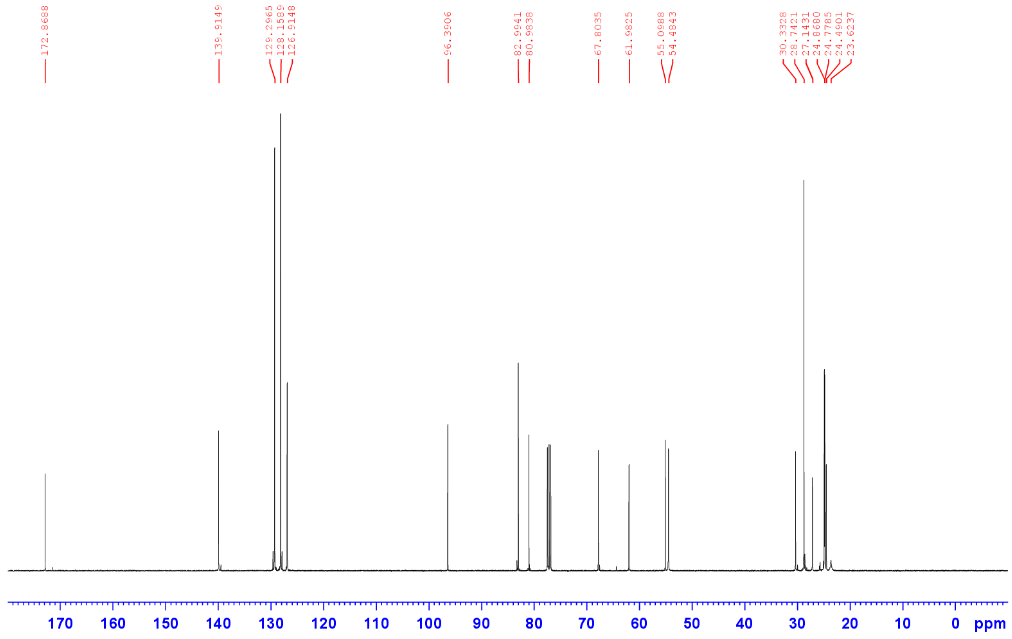

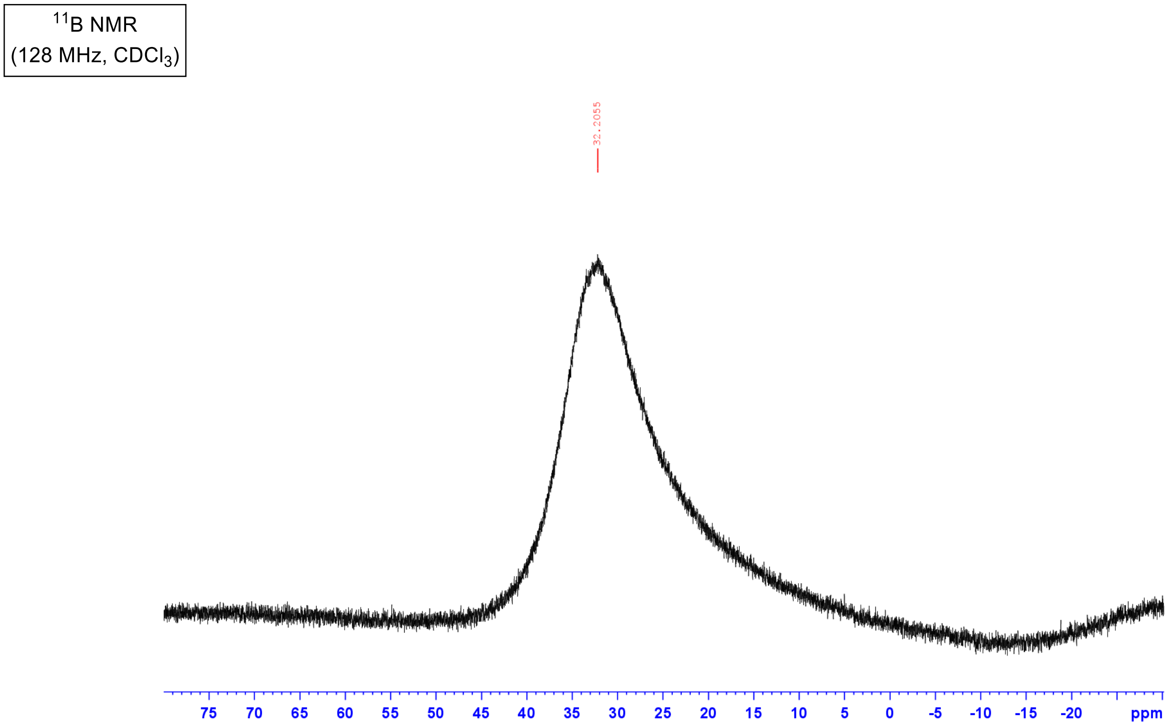
11B NMR
(128 MHz, CDCl3)
32.2055
75
70
65
60
55
50
45
40
35
30
25
20
15
10
5
0
-5
-10
-15
-20
ppm

[$^{1}$H, $^{13}$C{$^{1}$H}, and $^{11}$B NMR Spectra of ***anti*-3ka-O$^{t}$Bu**]

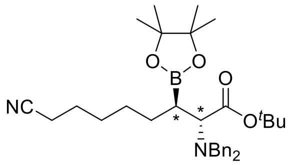


***anti*-3ka-O$^{t}$Bu**

$^{1}$H NMR
(400 MHz, $CDCl_3$)

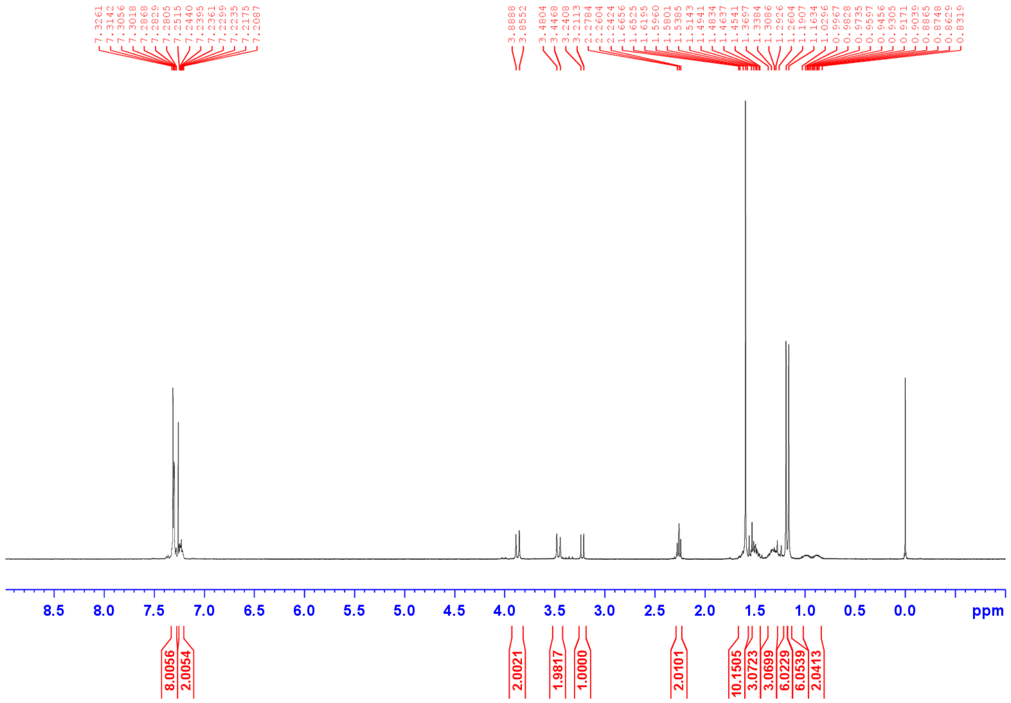


$^{13}$C{$^{1}$H} NMR
(100 MHz, $CDCl_3$)

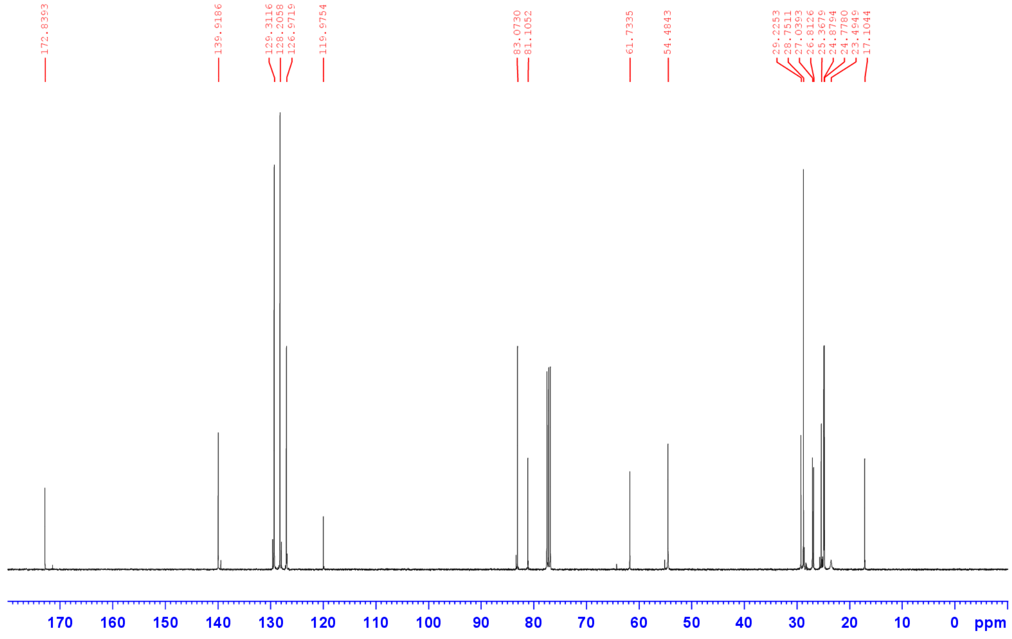

$^{11}B$ NMR
(128 MHz, $CDCl_3$)

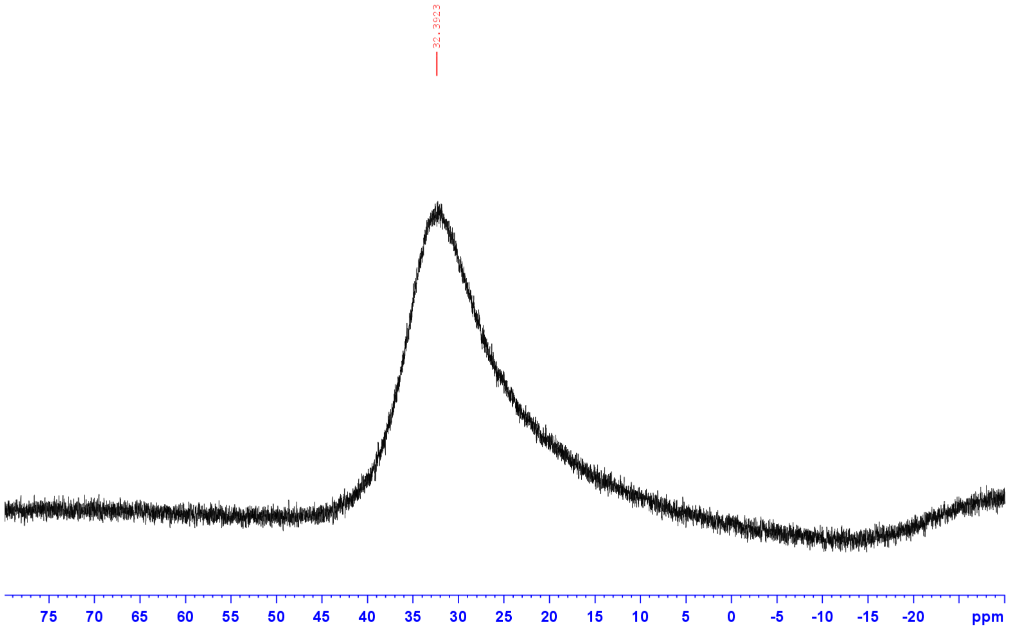

[$^{1}H$, $^{13}C\{^{1}H\}$, and $^{11}B$ NMR Spectra of ***anti*-3ma-O$^{t}$Bu**]

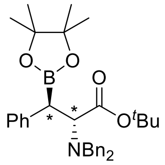


***anti*-3ma-O$^{t}$Bu**

$^{1}H$ NMR
(400 MHz, $CDCl_3$)

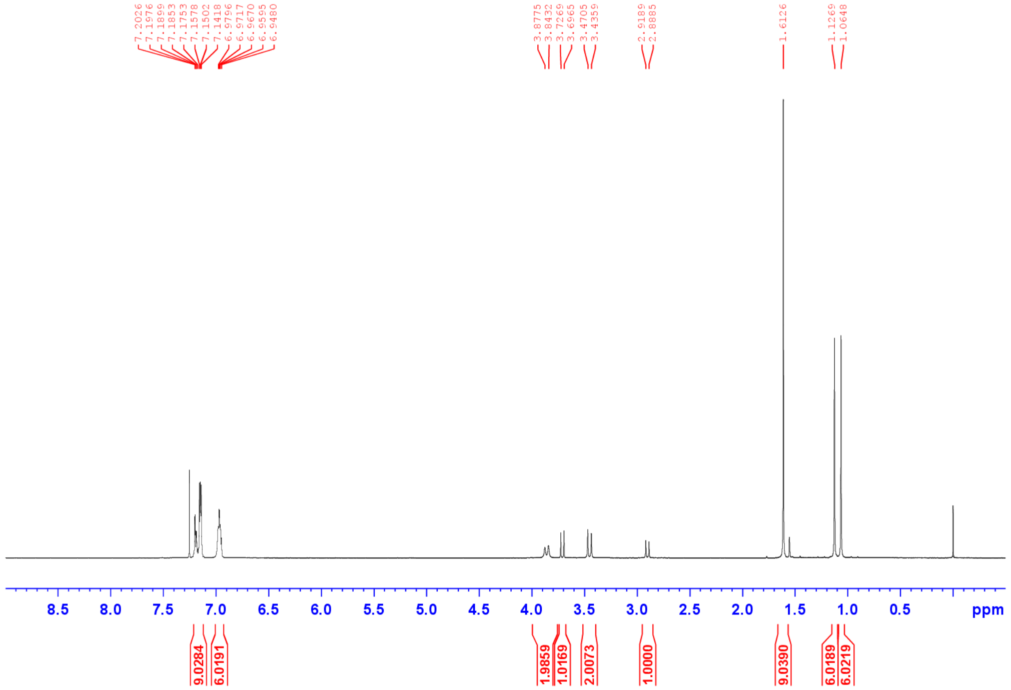


$^{13}C\{^{1}H\}$ NMR
(100 MHz, $CDCl_3$)

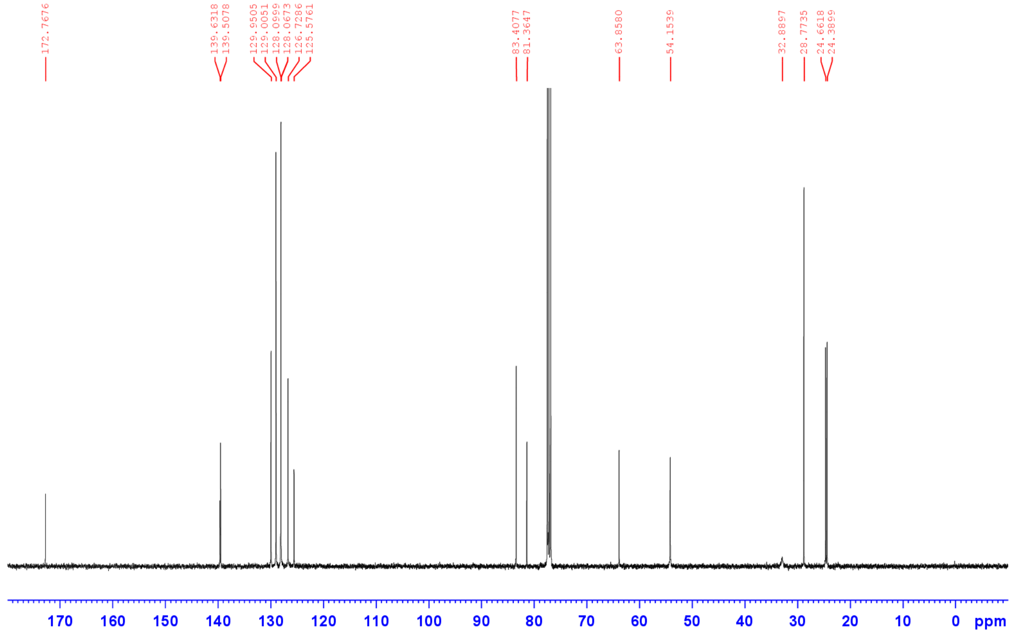

$^{11}$B NMR
(128 MHz, $CDCl_3$)

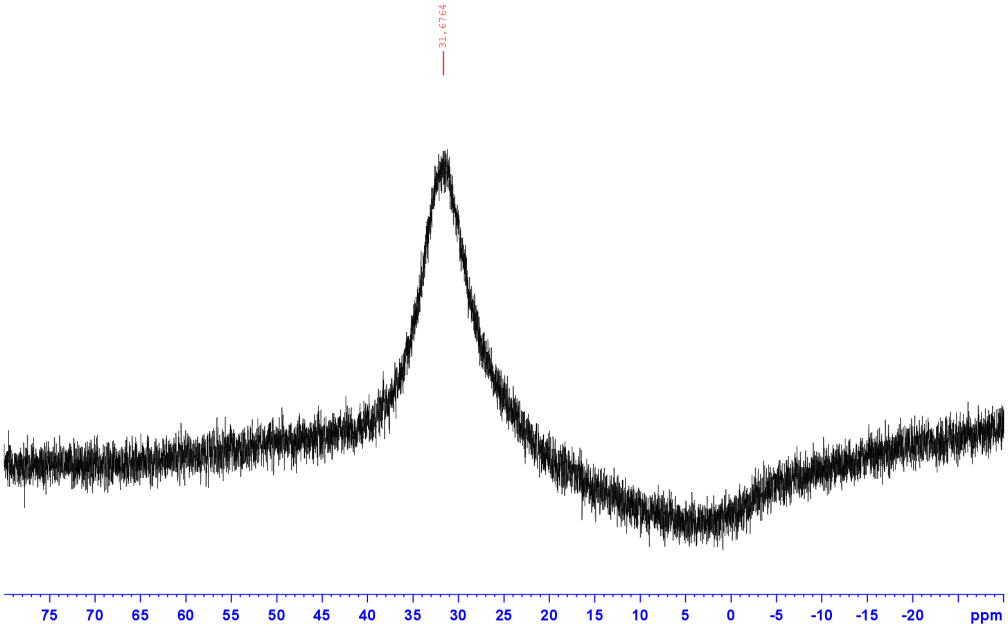

[$^{1}$H, $^{13}$C{$^{1}$H}, and $^{11}$B NMR Spectra of **3ac-O$^{t}$Bu**]

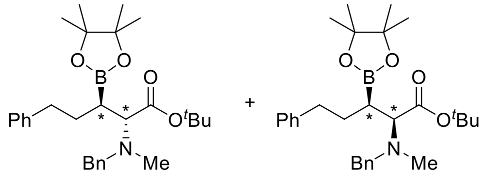


***anti*-3ac-O$^{t}$Bu** + ***syn*-3ac-O$^{t}$Bu**

$^{1}$H NMR
(400 MHz, $CDCl_3$)

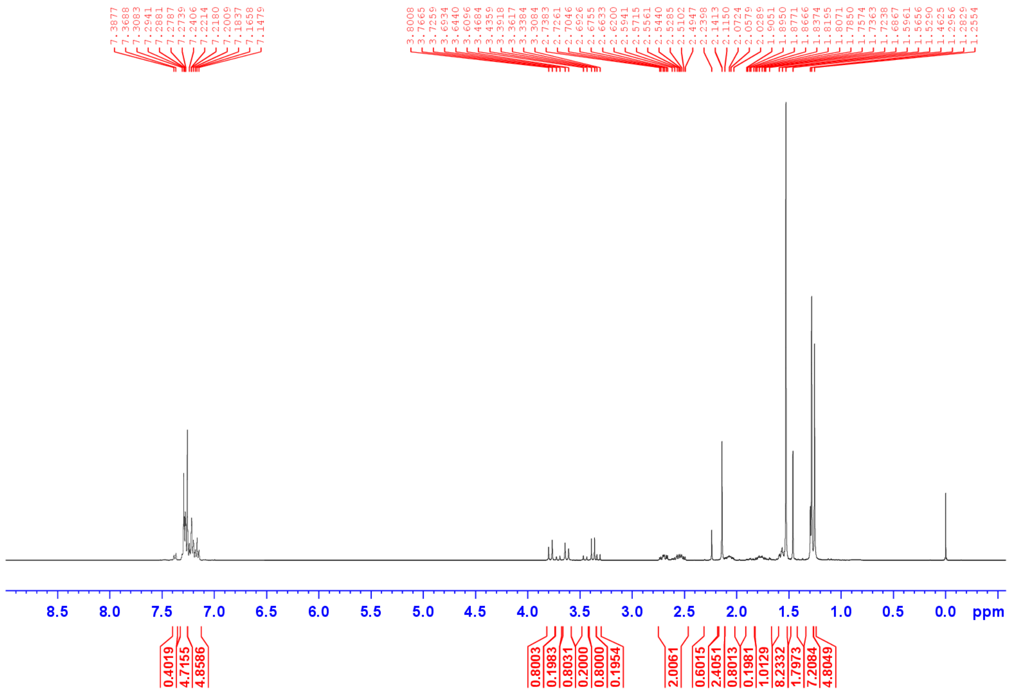


$^{13}$C{$^{1}$H} NMR
(100 MHz, $CDCl_3$)

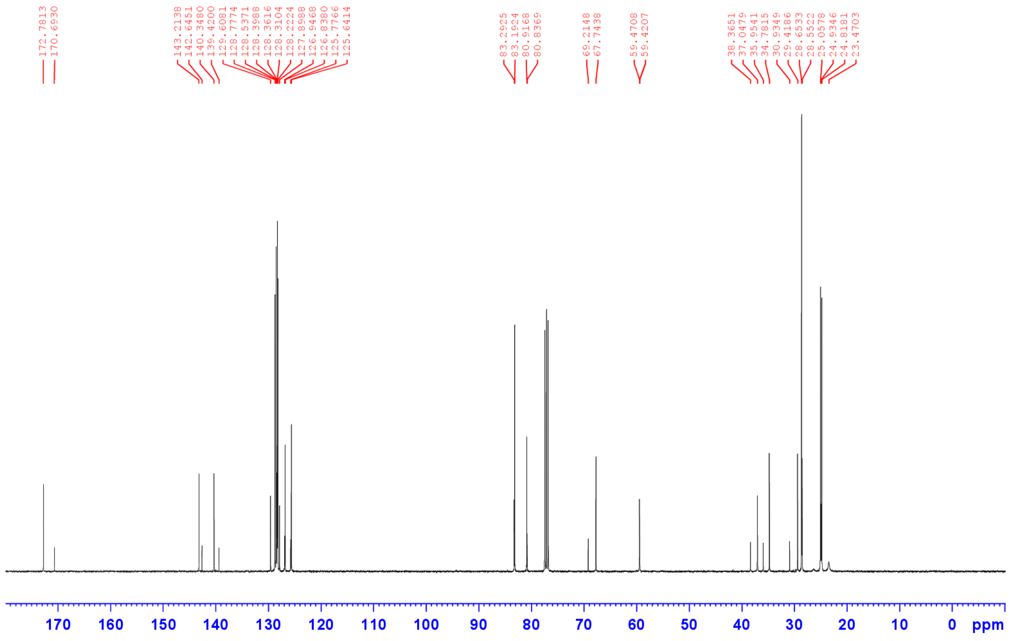

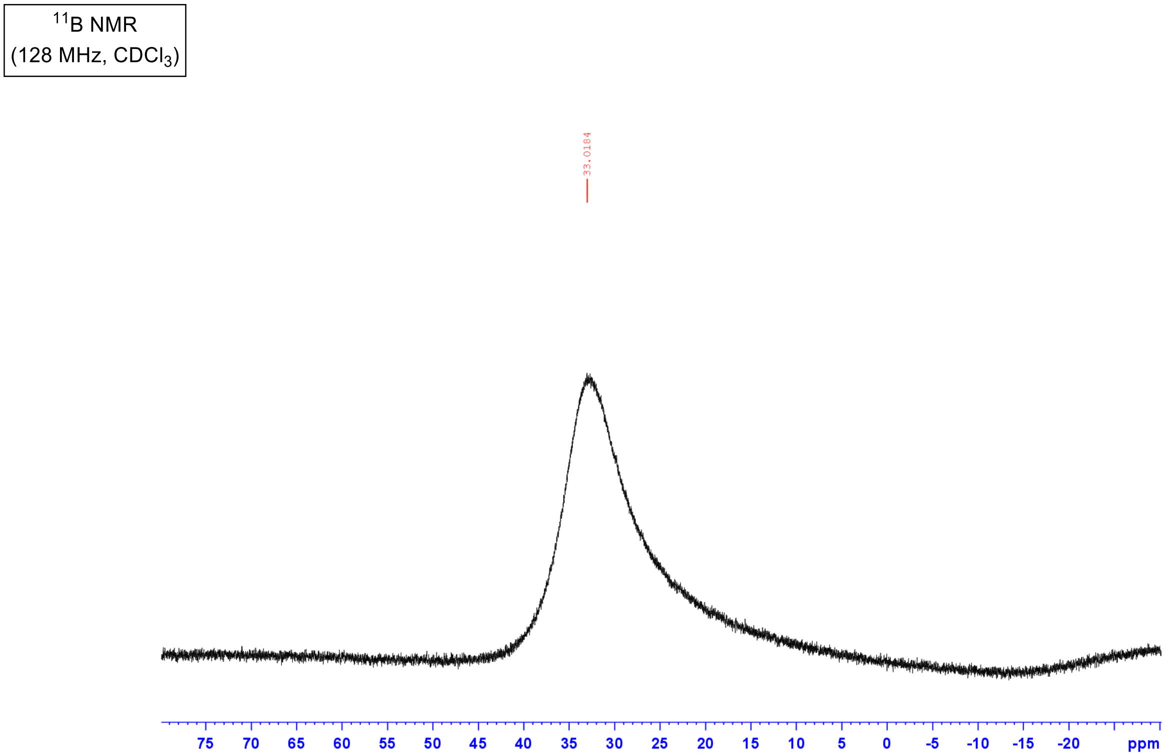

$^{11}$B NMR
(128 MHz, $CDCl_3$)
33.0184
75 70 65 60 55 50 45 40 35 30 25 20 15 10 5 0 -5 -10 -15 -20 ppm

[$^{1}$H, $^{13}$C{$^{1}$H}, and $^{11}$B NMR Spectra of **3af-O$^{t}$Bu**]

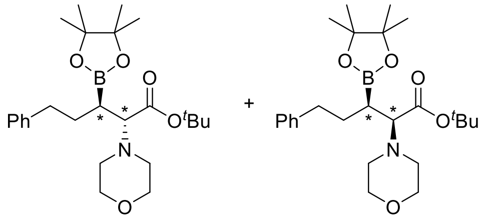


*anti*-3af-O$^{t}$Bu *syn*-3af-O$^{t}$Bu

$^{1}$H NMR
(400 MHz, $CDCl_3$)

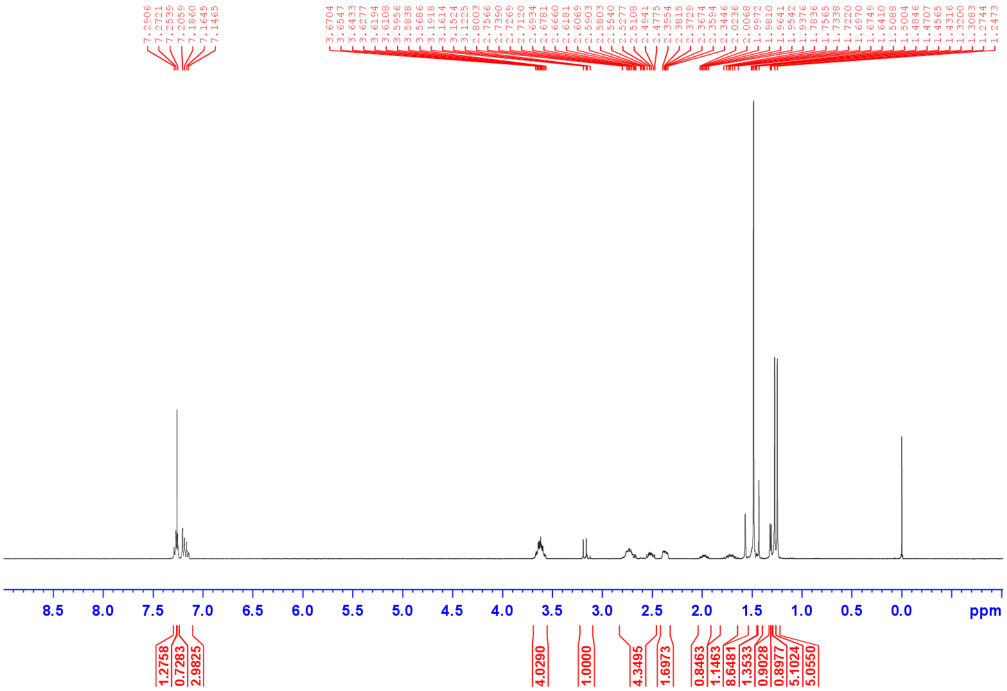


$^{13}$C{$^{1}$H} NMR
(100 MHz, $CDCl_3$)

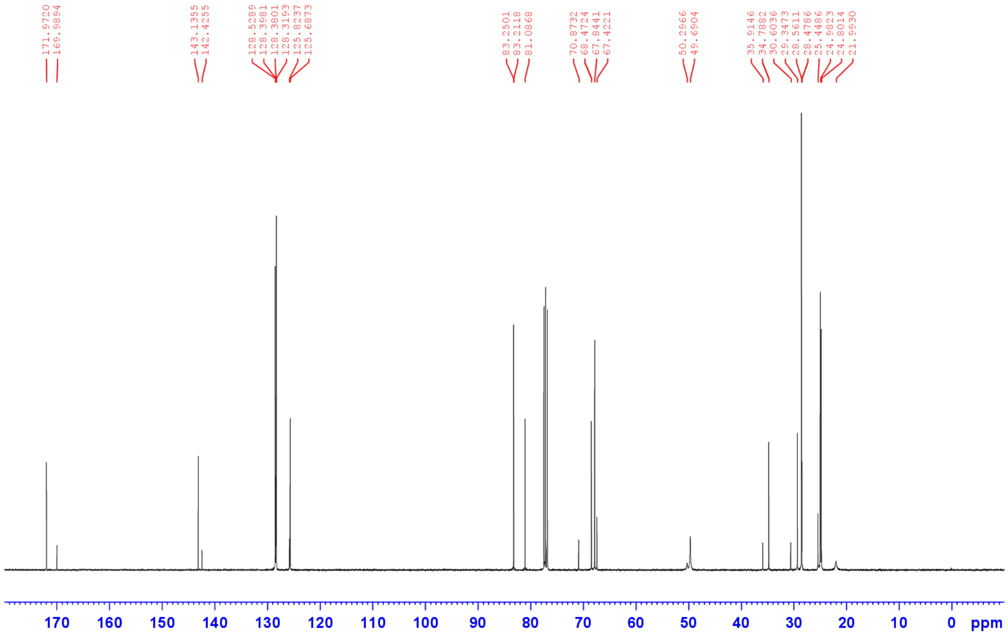

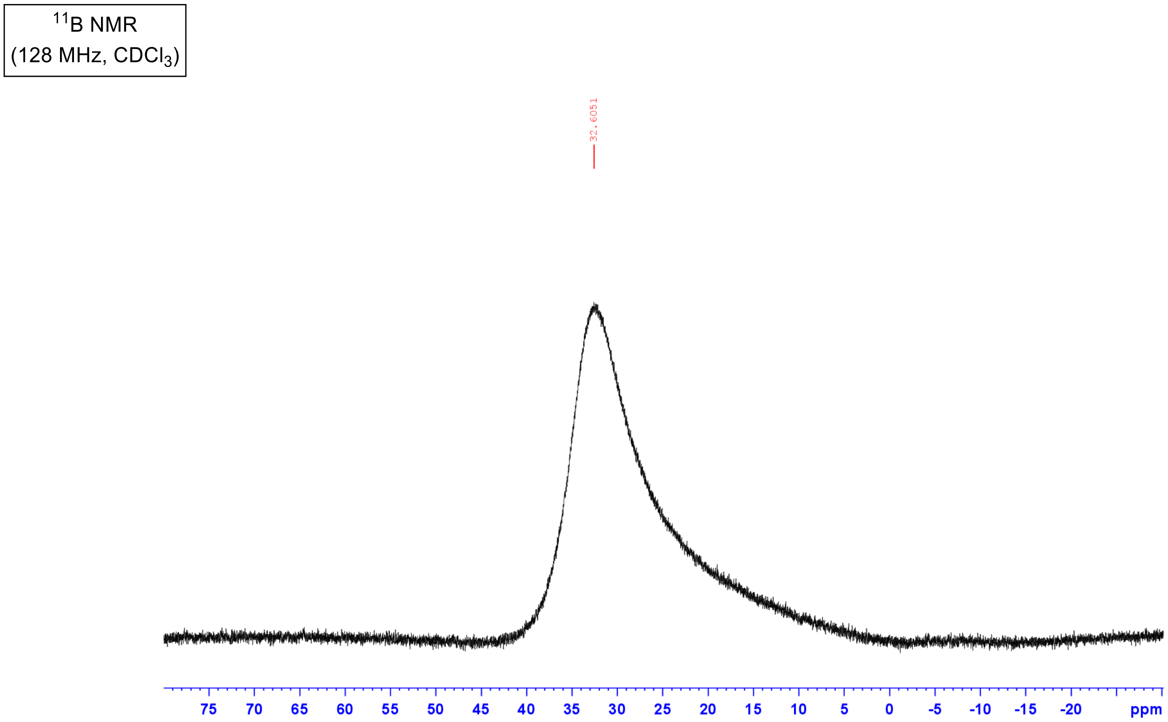

$^{11}$B NMR
(128 MHz, $CDCl_3$)
32.6051
75 70 65 60 55 50 45 40 35 30 25 20 15 10 5 0 -5 -10 -15 -20 ppm

[$^1$H and $^{13}$C{$^1$H} NMR Spectra of ***anti*-5**]

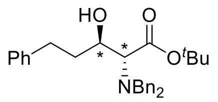

***anti*-5**

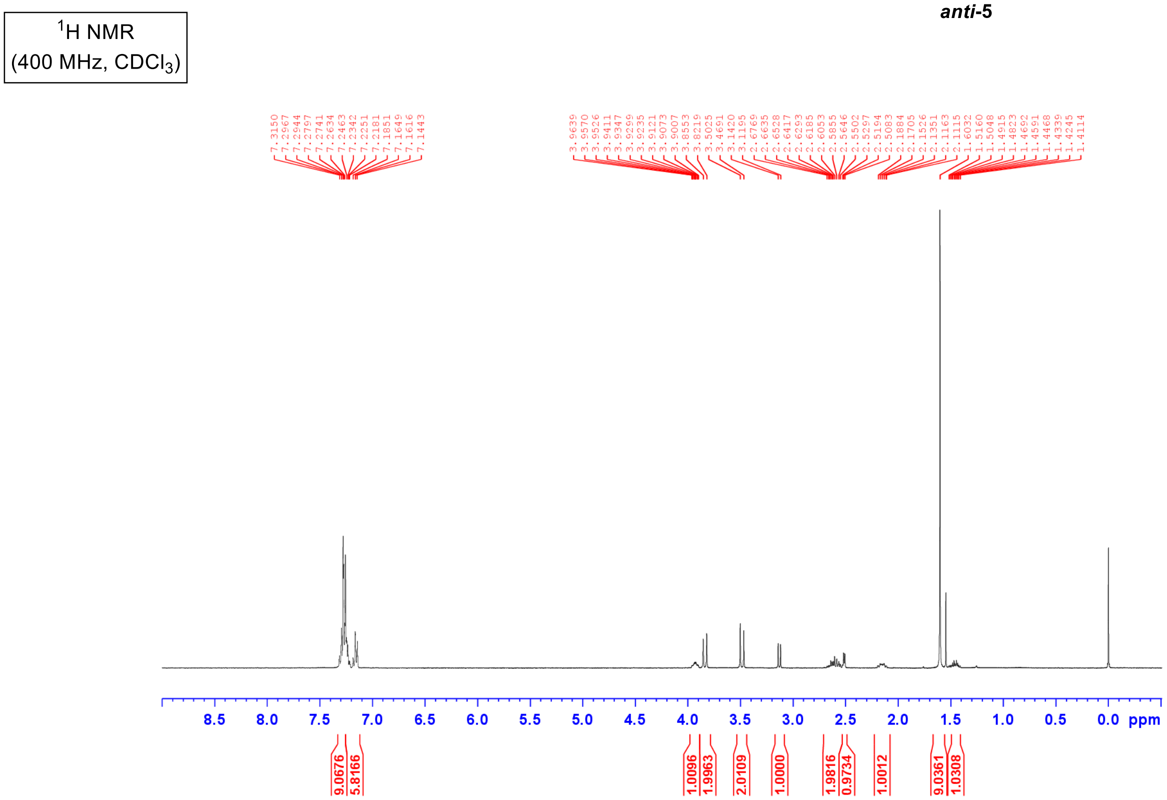


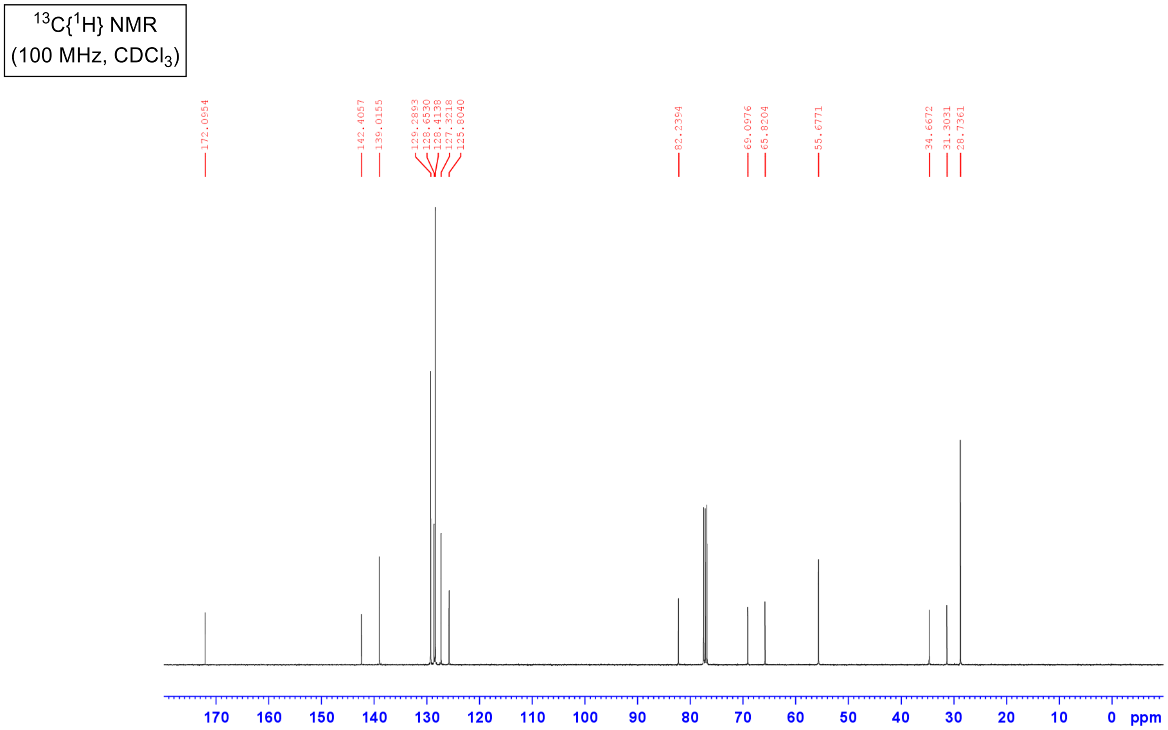

[$^{1}H$, $^{13}C\{^{1}H\}$, and $^{11}B$ NMR Spectra of ***anti*-6**]

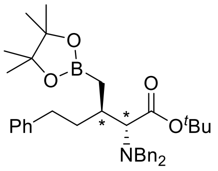


***anti*-6**

$^{1}H$ NMR
(400 MHz, $CDCl_3$)

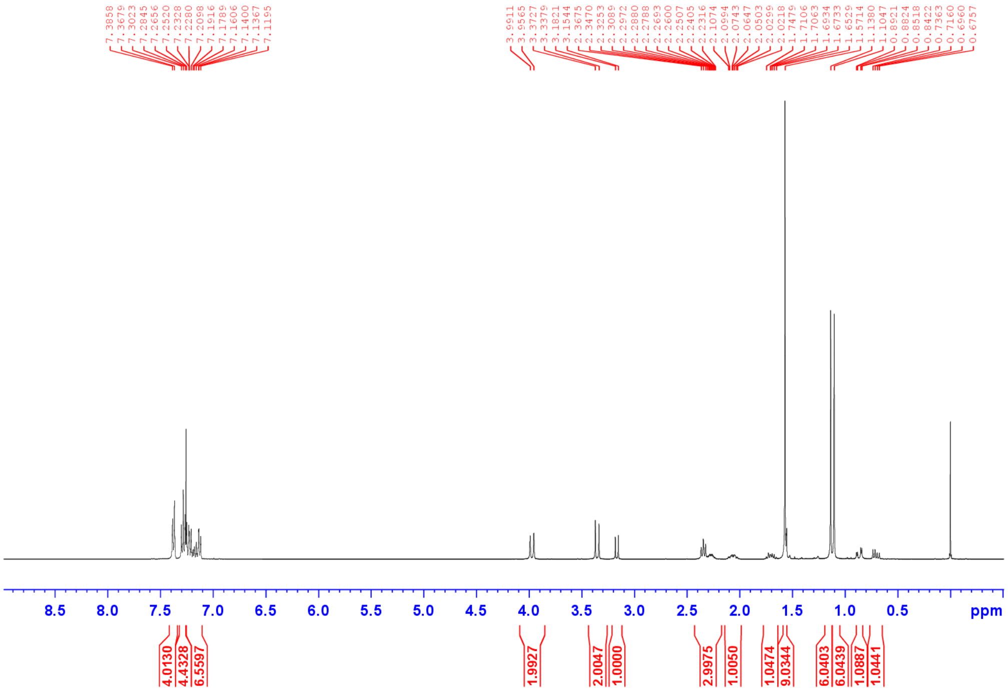


$^{13}C\{^{1}H\}$ NMR
(100 MHz, $CDCl_3$)

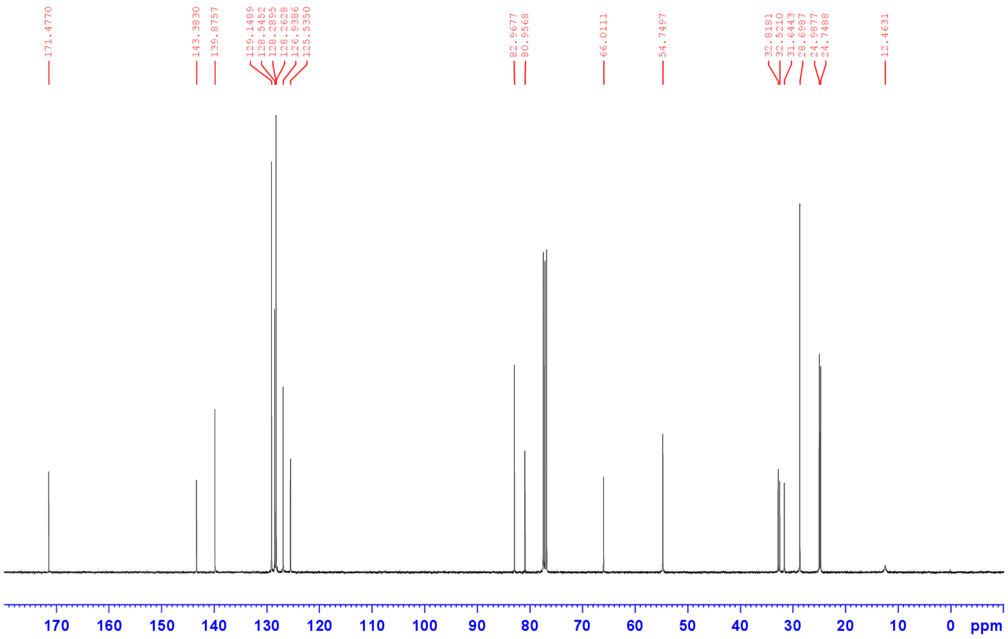

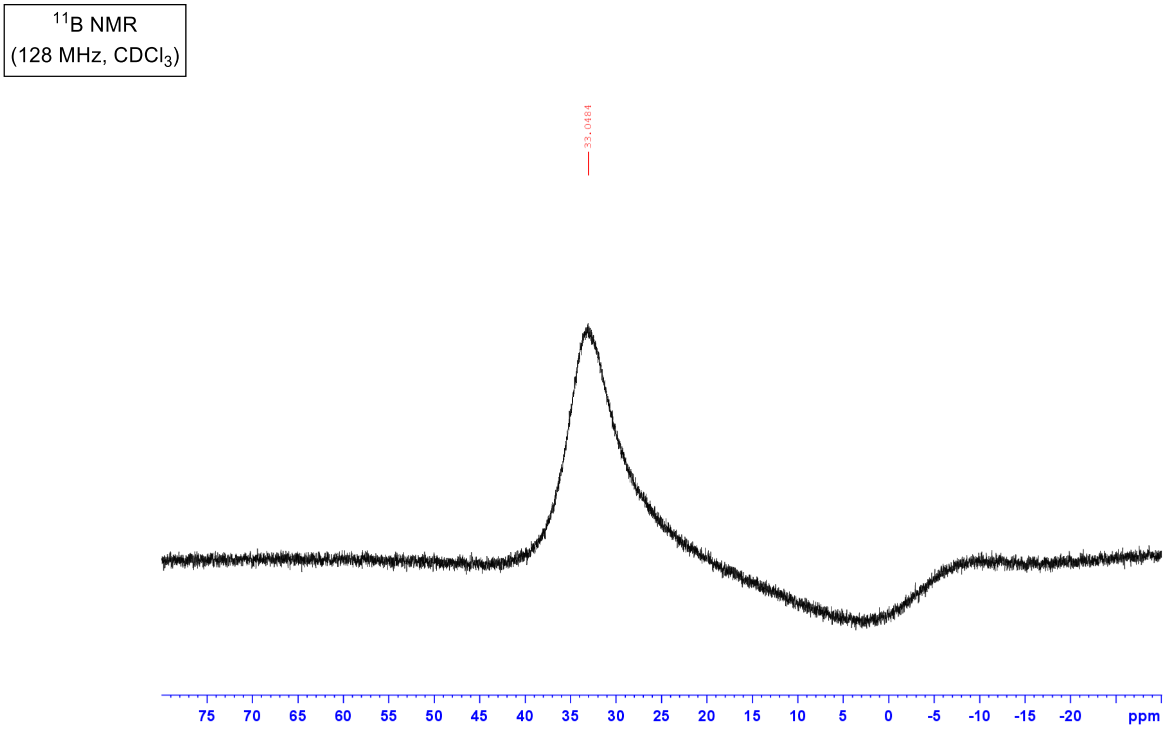

$^{11}$B NMR
(128 MHz, $CDCl_3$)
33.0484
75 70 65 60 55 50 45 40 35 30 25 20 15 10 5 0 -5 -10 -15 -20 ppm

[$^{1}$H and $^{13}$C{$^{1}$H} NMR Spectra of ***anti*-7**]

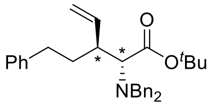


***anti*-7**

$^{1}$H NMR
(400 MHz, $CDCl_3$)

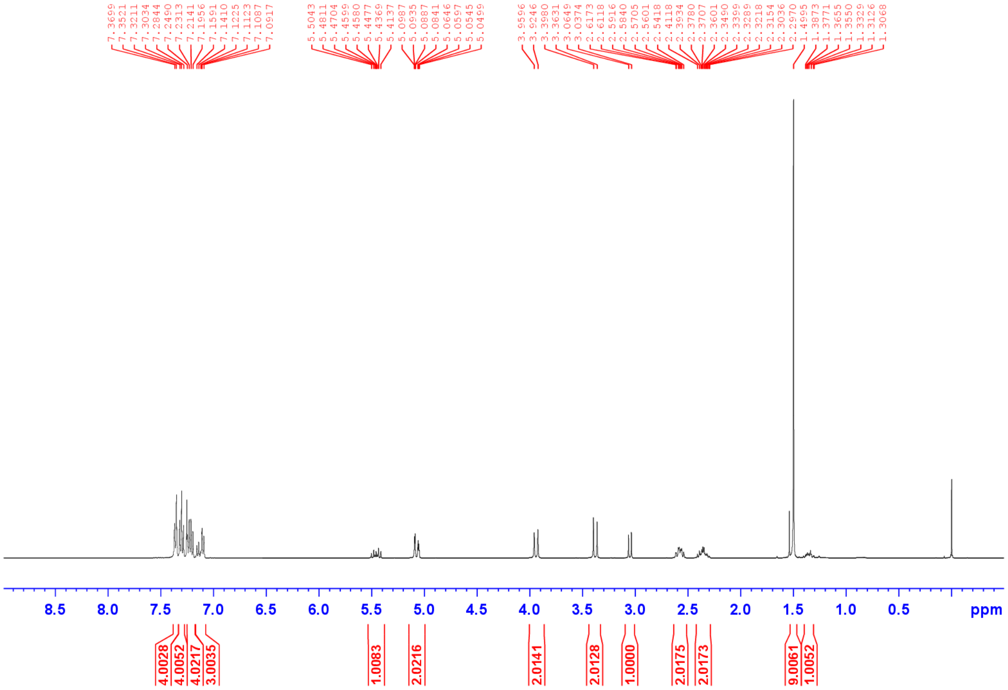


$^{13}$C{$^{1}$H} NMR
(100 MHz, $CDCl_3$)

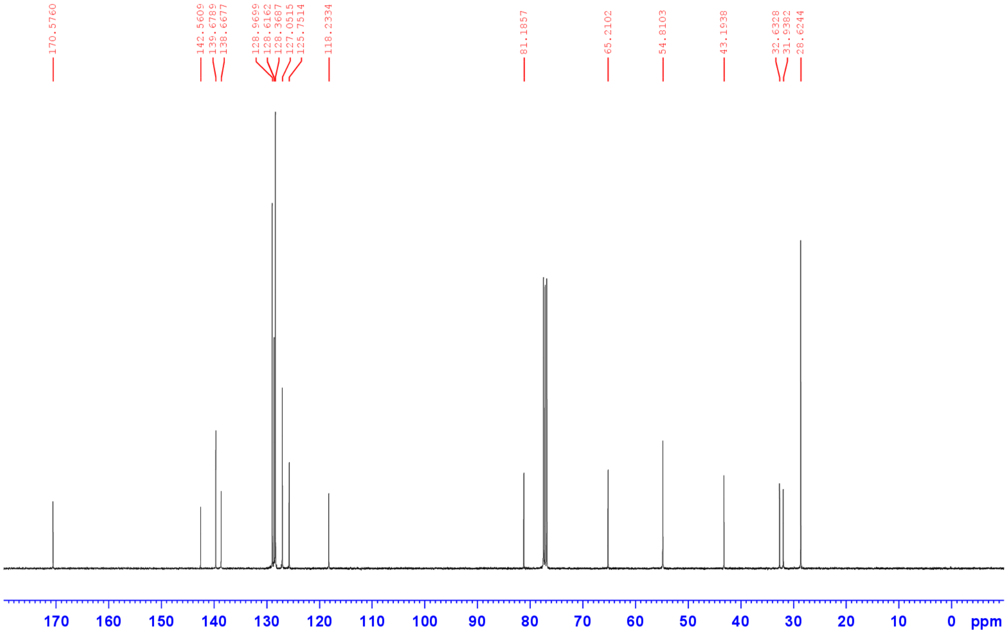

[$^{1}$H and $^{13}$C{$^{1}$H} NMR Spectra of ***anti*-8**]

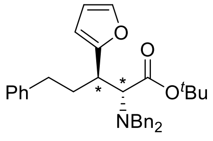


***anti*-8**

$^{1}$H NMR
(400 MHz, $CDCl_3$)

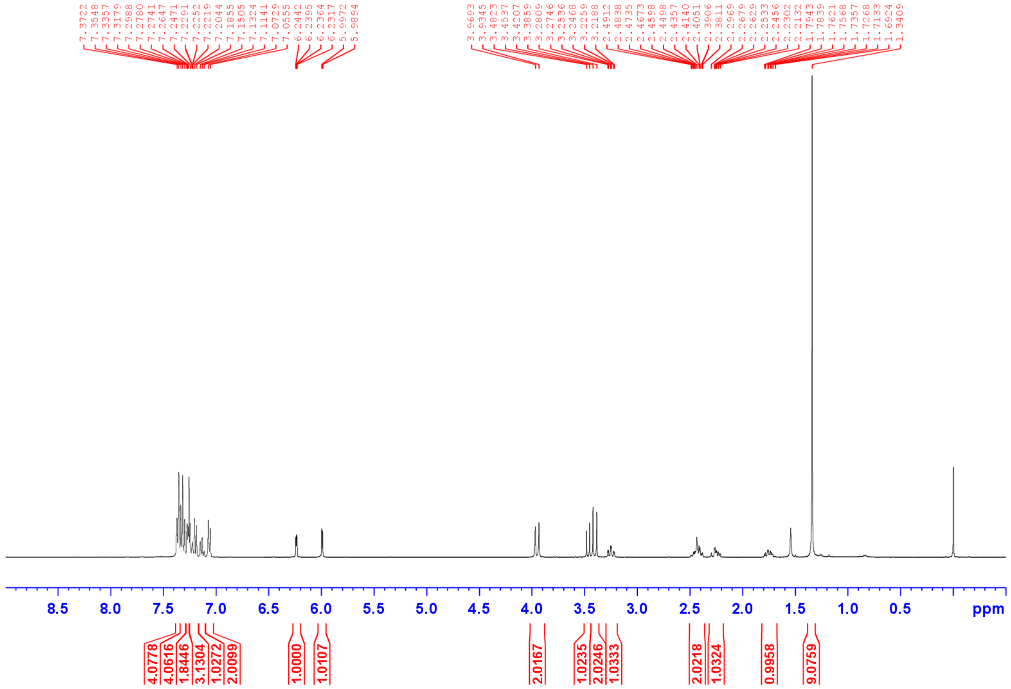


$^{13}$C{$^{1}$H} NMR
(100 MHz, $CDCl_3$)

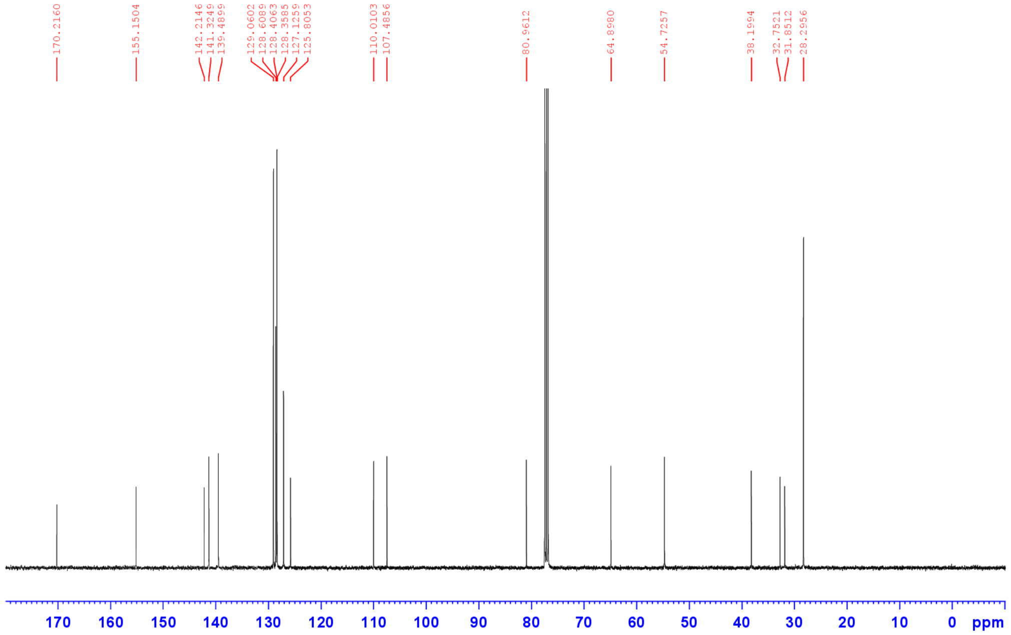

[$^{1}$H, $^{13}$C{$^{1}$H}, and $^{11}$B NMR Spectra of ***anti*-9**]

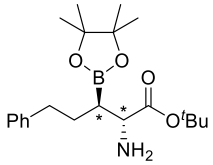


***anti*-9**

$^{1}$H NMR
(400 MHz, $CDCl_3$)

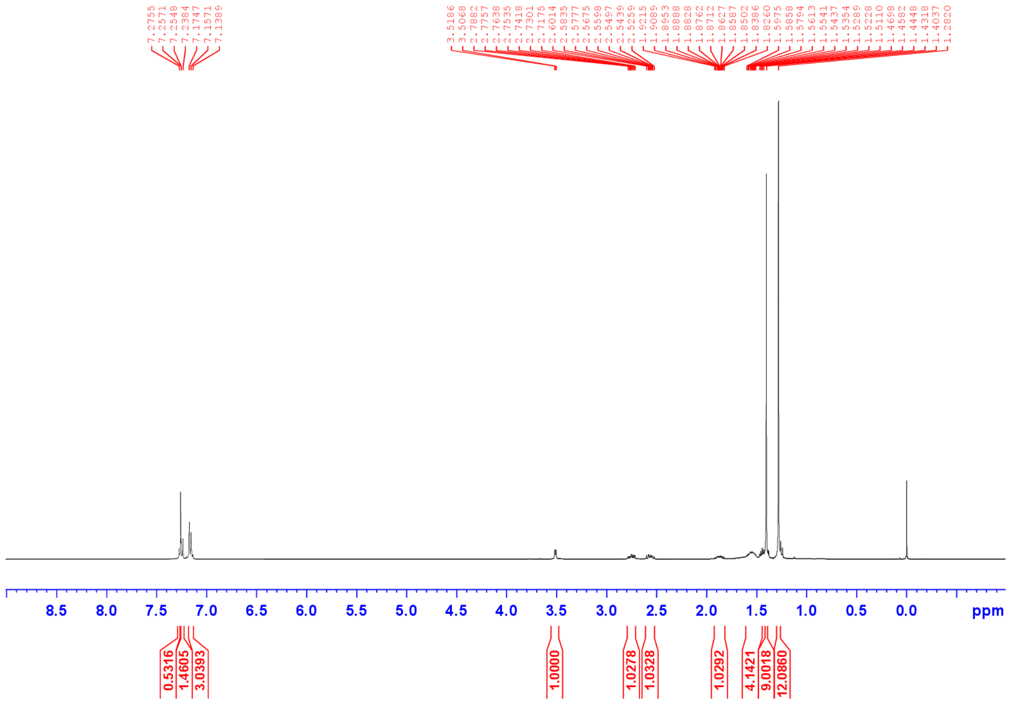


$^{13}$C{$^{1}$H} NMR
(100 MHz, $CDCl_3$)

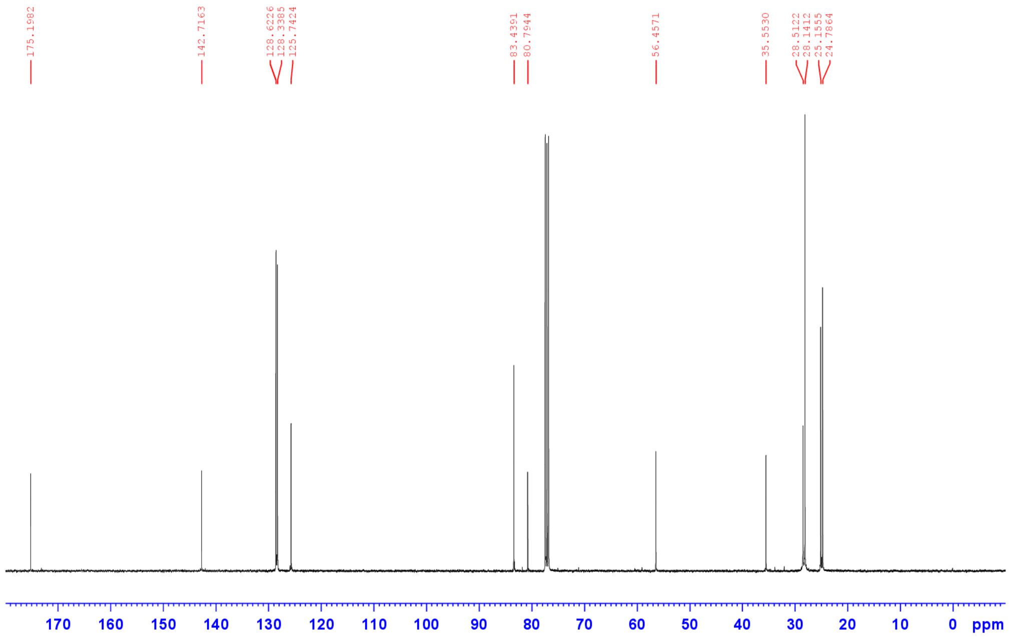

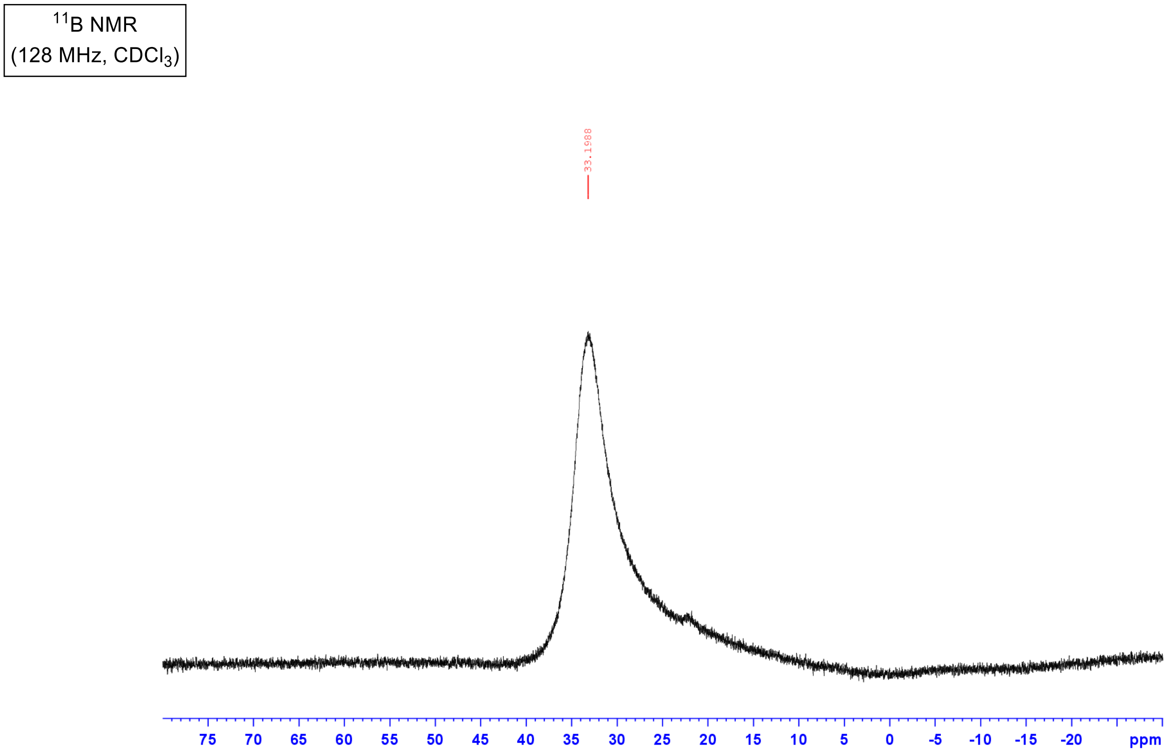

11B NMR
(128 MHz, CDCl3)
33.1988
75 70 65 60 55 50 45 40 35 30 25 20 15 10 5 0 -5 -10 -15 -20 ppm

**References**


(S1) P. W. Miller, M. Nieuwenhuyzen, J. P. H. Charmant and S. L. James, *Inorg. Chem.,* 2008, **47**, 8367.

(S2) R. B. Bedford, P. B. Brenner, E. Carter, J. Clifton, P. M. Cogswell, N. J. Gower, M. F. Haddow, J. N. Harvey, J. A. Kehl, D. M. Murphy, E. C. Neeve, M. L. Neidig, J. Nunn, B. E. R. Snyder and J. Taylor, *Organometallics,* 2014, **33**, 5767.

(S3) R. T. Yu and T. Rovis, *J. Am. Chem. Soc.,* 2006, **128**, 12370.

(S4) (*a*) S. Bovo, A. Scrivanti, M. Bertoldini, V. Beghetto and U. Matteoli, *Synthesis,* 2008, 2547. (*b*) Z.-C. Duan, X.-P. Hu, C. Zhang and Z. Zheng, *J. Org. Chem.,* 2010, **75**, 8319.

(S5) C. T. Nieto, M. M. Salgado, S. H. Dominguez, D. Diez and N. M. Garrido, *Tetrahedron: Asynmmetry,* 2014, **25**, 1046.

(S6) A. Kar, and N. P. Argade, *J. Org. Chem.,* 2002, **67**, 7131.

(S7) Y.-H. Xu, L.-H. Wu, J. Wang and T.-P. Loh, *Chem. Commun*., 2014, **50**, 7195.

(S8) A. Song, K. A. Parker and N. S. Sampson, *J. Am. Chem. Soc.,* 2009, **131**, 3444.

(S9) E. Falk, S. Makai, T. Delcaillau, L. Gürtler and B. Morandi, *Angew. Chem., Int. Ed.,* 2020, **59**, 21064.

(S10) G. Guanti, L. Banfi, E. Narisano and C. Scolastico, *Tetrahedron,* 1988, **44**, 3671.

(S11) T. Kobayashi, S. Nishino, M. Miura and K. Hirano, *Org. Lett.,* 2022, **24**, 1418.
